# Genome-Wide Identification and Characterization of the Potato *IQD* Family During Development and Stress

**DOI:** 10.3389/fgene.2021.693936

**Published:** 2021-07-27

**Authors:** Chao Mei, Yuwei Liu, Xue Dong, Qianna Song, Huijie Wang, Hongwei Shi, Ruiyun Feng

**Affiliations:** ^1^College of Agriculture, Shanxi Agricultural University, Taiyuan, China; ^2^College of Life Sciences, Hebei Agricultural University, Baoding, China; ^3^Center for Agricultural Genetic Resources Research, Shanxi Agricultural University, Taiyuan, China

**Keywords:** potato, *IQD* gene family, phylogenetic analysis, abiotic stress, expression profile

## Abstract

Calmodulin-binding proteins belong to the IQ67 domain (IQD) gene family and play essential roles in plant development and stress responses. However, the role of *IQD* gene family in potato (*Solanum tuberosum* L.) is yet to be known. In the present study, 23 *StIQD*s were identified in the potato genome and named *StIQD*1 to *StIQD*23. They were unevenly distributed on 10 of the 12 chromosomes. Phylogenetic analysis divided the IQDs into four subfamilies (IQD I–IV). *StIQD*s found in three of the four subfamilies. Synteny analysis confirmed that potato and tomato shared a close evolutionary relationship. Besides, RNA-Seq data analysis revealed that the expression of 19 of the 23 *StIQD*s was detected in at least one of the 12 tissues, and some of which showed a tissue-specific pattern. Quantitative reverse transcriptase–polymerase chain reaction results further confirmed that 14 *StIQD*s responded differently to various abiotic stresses, including drought, extreme temperature, and CaCl_2_ treatment, suggesting their significance in stress response. This study presents a comprehensive overview of the potato *IQD* gene family and lays a foundation for further analysis of the *StIQD*s functions in plant development and stress response.

## Introduction

Potato (*Solanum tuberosum* L.) is an annual Solanaceae crop that is widely cultivated worldwide (Xu et al., 2011). It is the fourth most food-cultivated crop worldwide after wheat, rice, and maize (Zhang et al., 2017). Its tubers are an important source of starch, protein, antioxidants, and vitamins and are also used for vegetative propagation (Burlingame et al., 2009).

There are numerous signaling pathways and complex networks in plants participating in plants’ response to stress (Mulligan et al., 1997; Lamers et al., 2020). Among them, calcium (Ca^2+^) signaling plays a major role in many biological functions (Schulz et al., 2013; Aldon et al., 2018). It utilized intracellular Ca^2+^ as a second messenger. The Ca^2+^-binding proteins act as Ca^2+^ sensors, thereby causing plants to respond to various biological processes by regulating the concentration of Ca^2+^ in the cytoplasm instantaneously (Bouché et al., 2005). There are four major classes of Ca^2+^ sensors in plants. They include calmodulins (CaMs), CaM-like proteins, calcineurin B–like proteins, and Ca^2+^-dependent protein kinases (Ranty et al., 2006). Calmodulins are among the most dominant proteins in Ca^2+^ sensors. Their role in Ca^2+^ signal transduction has been widely studied (Bouché et al., 2005). CaMs lack enzymatic activity but can interact and regulate the activity of the target proteins. The CaM-mediated signal can only play its role in regulating the physiological functions of cells through its downstream target protein CaM-binding proteins (CaMBPs) (Perochon et al., 2011; Kudla et al., 2018). Studies postulate that the IQ67 motif contains proteins, known as IQ67 domain (IQD) protein families. They are common representatives of CaMBPs (Abel et al., 2013; Bürstenbinder et al., 2017a). The IQD family proteins have two common features. It contains a conserved domain of 67 amino acid residues (the IQD) and a conserved exon–intron boundary that interrupts codons 16 and 17 via a phase-0 intron (Abel et al., 2005). To date, *IQD* gene families have been comprehensively analyzed in numerous plants. For instance, 33 *IQD*-like genes have been identified in *Arabidopsis*, 29 in rice, 26 in maize, 23 in *Brachypodium*, and 40 in poplar (Abel et al., 2005; Filiz et al., 2013; Ma et al., 2014; Cai et al., 2016).

The functions of *IQD* family genes have also been widely studied. In *Arabidopsis*, they regulate cell function, shape, and growth. Overexpression of *AtIQD1* could enhance the accumulation of glucosinolate and strengthen resistance to herbivores (Levy et al., 2005; Bürstenbinder et al., 2017a). The *IQD12/SUN* of tomato regulates multiple tomato developmental processes, especially the morphological development of the leaves and fruit shape (Xiao et al., 2008; Wu et al., 2011). IQDs have been found to participate in abiotic stress responses. For example, 12 *IQD* members participate in poplar’s response to methyl jasmonate (MeJA) and Polyethylene glycol (PEG) treatment (Ma et al., 2014). In maize, the *ZmIQD* genes play crucial roles in response to drought stress (Cai et al., 2016). These reports indicate that the *IQD* genes can regulate plant development and response to stress or hormonal changes. However, IQD members and functions in potato are yet to be identified. In this study, the *IQD* gene family from the whole potato genome was identified. Detailed information about the *IQDs* regarding their chromosomal locations, synteny, and the phylogenetic relationship was also retrieved. In addition, expression levels of the *IQDs* in different tissues were analyzed using RNA-Seq data. Genes responsible for six abiotic stresses were further identified by quantitative reverse transcriptase–polymerase chain reaction (qRT-PCR). This study provides a theoretical basis for further studies on the function of the *IQD* gene family in potato.

## Materials and Methods

### Identification of *IQD* Genes in Potato

The protein sequences of *Arabidopsis thaliana* IQD family members (AtIQDs) were downloaded from the TAIR database.^1^ The protein sequences of potato (version 4.03) were downloaded from PGSC.^2^ First, the AtIQD sequences were utilized as query, and BLASTP program was used to search against the potato proteins for the identification of the potato *StIQD* family. The sequences were selected as candidate *StIQD* if their *E*-value was < 0.001. Second, the candidate sequences were further confirmed by using the HMMER software^3^ with the Pfam IQD motif (PF00612) and SMART (Simple Modular Architecture Research Tool) online tool (Letunic et al., 2021).

### Analysis of Sequences and Motifs

The physiochemical data [length, molecular weight, and theoretical isoelectric points (pIs)] of StIQD protein sequences were analyzed by the ProtParam server of Expasy.^4^ For the gene structure of *StIQD* analysis, the sequences of DNA and their corresponding coding sequences were analyzed by using GSDS (Hu et al., 2015). The conserved domains of *StIQD* gene family members in potato were analyzed by MEME online tool (Bailey et al., 2015), and the maximum number of motifs to identify was set to 10. In addition, the position of putative CaM-binding sites of each StIQD protein was predicted by the CaM Target Database (Yap et al., 2000).

### Chromosomal Location and Gene Synteny

The chromosomal location of *StIQD* genes was based on genome annotation information in PGSC database and drafted by Mapgene2chrom online tool.^5^ For the synteny analysis of genes, the genome and genome annotation file of potato (version 4.03), tomato (version SL3.0) and *Arabidopsis* (version TAIR 10) were used, and the MCScan of python version^6^ (Python-version) was used to analyze the synteny.

### Phylogenetic Analysis

Protein sequences of the potato, tomato, *Arabidopsis*, maize, and rice were aligned using ClustalX2 (Larkin et al., 2007), and the multiple sequence alignment result was used for phylogenetic analysis. For the phylogenetic analysis, the FastTree 2 software was used by using the maximum likelihood method, and the bootstrap value was set as 1,000 (Price et al., 2010).

### RNA-Seq Data Analysis

The published transcriptome data (NCBI SRA database accession no. SRA030516) of different tissues of potato were used to analyze *StIQD*s expression patterns (Xu et al., 2011). For the expression analysis, normalized gene expression values FPKM (fragments per kilobase of exon per million fragments mapped) were first transformed using log2 (FPKM + 1) and then plotted by using ggplot2 in R (Wickham, 2011). The MeV^7^ was used to conduct the coexpression analysis. The gene expression levels were used and clustered using the k-means method. For the coexpressed genes, the agriGO (Tian et al., 2017) was used for GO enrichment analysis.

### Plant Materials and Stress Treatments

The potato cultivar Desiree sterile tissue cultured seedlings for 15 days were transferred to perlite substrate and were initially irrigated with standard Hoagland culture medium for another 15 days. The culture temperature was set at 25°C, and the light cycle was set at 16 h/8 h (light/dark). Seedlings with consistent growth were selected and washed with 1/4 Hoagland culture medium for three times. The seedlings were treated with 150 mmol/L NaCl, 50 mmol/L CaCl_2_, 20% PEG6000, 4 and 35°C for 24 h, respectively. Each treatment was repeated for three times, and the same volume of double steaming water was used as the control. The leaves and root tissues of each treatment were quickly treated with liquid nitrogen and then stored at −80°C.

### RNA Extraction and qRT-PCR Analysis

Total RNA was isolated from the collected samples using Trizol Reagents (Invitrogen, United States). After digestion with DNase I, the quantified of the RNA was evaluated by using NanoDrop 2000. Approximately 4 μg of total RNA was reverse transcribed by M-MLV reverse transcriptase (Promega, United States). The qRT-PCR was performed using SYBR Green (TaKaRa, Japan) and BioRad Real-Time System CFX96TM C1000 thermal cycler (Bio-Rad, Hercules, CA, United States). The PCR conditions were 95°C for 10 min, followed by 40 cycles of 95°C for 15 s, 55°C for 15 s, and 72°C for 30 s. Three replicates were performed for each sample. The 2^–ΔΔCt^ method was used to calculate the relative gene expression levels, and the potato housekeeping *EF1*α gene was used as reference (Livak and Schmittgen, 2001). The primer sequences used in this study were listed in Supplementary Table 1.

### *cis*-Element Analysis

The putative promoters of *IQDs*, composed of 2-kb upstream of transcriptional start site, were acquired on the base of the annotation of potato genome. The PLANT CARE online software was used to analyze the *cis*-acting elements of promoters (Lescot et al., 2002).

### Protein–Protein Interaction Network Analysis

For the protein–protein interaction (PPI) predicted analysis, all of the StIQD proteins sequences were first searched against the STRING database version 11.0 (Szklarczyk et al., 2019). The “confidence score” of STRING was used, and a confidence score of > 0.7 (high confidence) between proteins was used. The interaction networks of protein-protein generated by STRING were used to show the relationship of proteins with StIQDs.

## Results

### Genome-Wide Identification of *IQD* Gene Family

The IQD motif (PF00612) was used as the query sequence to search for similar potato protein sequence in the HMMER program. The search was performed to identify the IQD family members in potato. The SMART tool was then used to confirm whether the candidates contained the IQ domain. Consequently, 23 *IQD* genes that comprised 33 transcripts were identified and named as *StIQD1* to *StIQD23* according to the position of their corresponding genes on chromosomes 1–12 (Table 1, Supplementary Table 2, and Supplementary Figure 1).

**TABLE 1:** List of 23 IQD genes identified in potato with their protein sequence physiochemical data.

**Gene**	**Chromosome location**	**Gene ID**	**Transcript ID**	**Protein ID**	**Length of protein**	**PI**	**Relative molecular mass (*M*_*r*_)**
*StIQD1*	Chr1:6661315:6666017	PGSC0003DMG400014723	PGSC0003DMT400038175	PGSC0003DMP400025941	517	10.36	58,480.9
*StIQD2*	Chr1:67189254:67202002	PGSC0003DMG400031124	PGSC0003DMT400079917	PGSC0003DMP400054216	1,570	7.47	178,326.4
			PGSC0003DMT400079918	PGSC0003DMP400054217	1,568	7.55	178,083.2
			PGSC0003DMT400079919	PGSC0003DMP400054218	1,565	7.55	177,728.7
			PGSC0003DMT400079921	PGSC0003DMP400054220	1,571	7.47	178,454.5
*StIQD3*	Chr1:75187029:75188561	PGSC0003DMG400019447	PGSC0003DMT400050070	PGSC0003DMP400033806	410	11.34	46,581.0
*StIQD4*	Chr2:34786809:34789703	PGSC0003DMG400022412	PGSC0003DMT400057729	PGSC0003DMP400038858	424	10.20	49,448.3
*StIQD5*	Chr2:36219816:36222212	PGSC0003DMG400016486	PGSC0003DMT400042507	PGSC0003DMP400028833	448	11.49	51,583.6
*StIQD6*	Chr2:38915299:38916613	PGSC0003DMG400003665	PGSC0003DMT400009431	PGSC0003DMP400006554	131	12.52	15,087.6
*StIQD7*	Chr2:46382552:46388752	PGSC0003DMG400001315	PGSC0003DMT400003322	PGSC0003DMP400002357	558	10.25	61,349.6
			PGSC0003DMT400003323	PGSC0003DMP400002358	553	10.27	60,668.9
*StIQD8*	Chr3:48239179:48242195	PGSC0003DMG400009086	PGSC0003DMT400023467	PGSC0003DMP400016001	469	10.37	52,173.9
*StIQD9*	Chr3:61021938:61024716	PGSC0003DMG400002492	PGSC0003DMT400006376	PGSC0003DMP400004403	427	10.56	47,575.2
*StIQD10*	Chr4:11174528:11178268	PGSC0003DMG400012720	PGSC0003DMT400033127	PGSC0003DMP400022534	405	10.94	46,948.8
			PGSC0003DMT400033128	PGSC0003DMP400022535	405	10.94	46,948.8
*StIQD11*	Chr4:70582358:70588266	PGSC0003DMG400003722	PGSC0003DMT400009544	PGSC0003DMP400006640	865	5.50	94,510.8
			PGSC0003DMT400009545	PGSC0003DMP400006641	865	5.50	94,510.8
*StIQD12*	Chr5:5870362:5877158	PGSC0003DMG400017629	PGSC0003DMT400045444	PGSC0003DMP400030791	556	10.36	61,294.5
*StIQD13*	Chr5:14418005:14425294	PGSC0003DMG400034313	PGSC0003DMT400084738	PGSC0003DMP400056415	684	5.58	76,458.2
*StIQD14*	Chr6:1450489:1458495	PGSC0003DMG400010897	PGSC0003DMT400028274	PGSC0003DMP400019257	745	9.56	84,711.1
*StIQD15*	Chr6:1945555:1957734	PGSC0003DMG400014709	PGSC0003DMT400038143	PGSC0003DMP400025918	852	9.50	97,404.1
			PGSC0003DMT400038144	PGSC0003DMP400025919	689	10.23	78,634.2
*StIQD16*	Chr6:38372854:38376570	PGSC0003DMG400019610	PGSC0003DMT400050471	PGSC0003DMP400034070	407	9.89	46,356.6
*StIQD17*	Chr7:37444050:37462795	PGSC0003DMG400020947	PGSC0003DMT400053990	PGSC0003DMP400036362	849	8.03	96,377.9
			PGSC0003DMT400053991	PGSC0003DMP400036363	1,529	8.41	172,953.7
*StIQD18*	Chr7:53728011:53733726	PGSC0003DMG400019284	PGSC0003DMT400049643	PGSC0003DMP400033506	592	9.47	67,915.2
*StIQD19*	Chr8:4751904:4754698	PGSC0003DMG400005774	PGSC0003DMT400014796	PGSC0003DMP400010241	360	10.55	40,755.4
*StIQD20*	Chr8:7262943:7268234	PGSC0003DMG400016053	PGSC0003DMT400041427	PGSC0003DMP400028070	535	11.18	60,496.4
*StIQD21*	Chr8:56711796:56714521	PGSC0003DMG400012115	PGSC0003DMT400031575	PGSC0003DMP400021404	207	11.77	23,551.0
			PGSC0003DMT400031576	PGSC0003DMP400021405	496	10.70	56,288.8
*StIQD22*	Chr9:45709284:45730781	PGSC0003DMG400022027	PGSC0003DMT400056647	PGSC0003DMP400038095	1,514	7.97	171,718.6
*StIQD23*	Chr12:60065036:60071133	PGSC0003DMG400004701	PGSC0003DMT400011979	PGSC0003DMP400008334	702	6.52	79,561.1
			PGSC0003DMT400011981	PGSC0003DMP400008336	462	9.64	53,019.1

The physical parameters of each IQD protein were subsequently calculated using the ExPASy server. As shown in Table 1, the full-length protein sequences of the *StIQD* ranged between 131 (StIQD6) and 1,571 amino acids (StIQD2). Their molecular weights ranged between 15,087.6 and 178,454.5 Da. Most of the StIQDs had relatively high pIs (9.38 ± 1.89), with only two having less than 7. This finding was in agreement with that of IQDs identified in other plants, such as *Arabidopsis* and maize (Levy et al., 2005; Cai et al., 2016).

### Conserved Motifs and Structural Analysis of Potato *IQD* Genes

**FIGURE 1:**
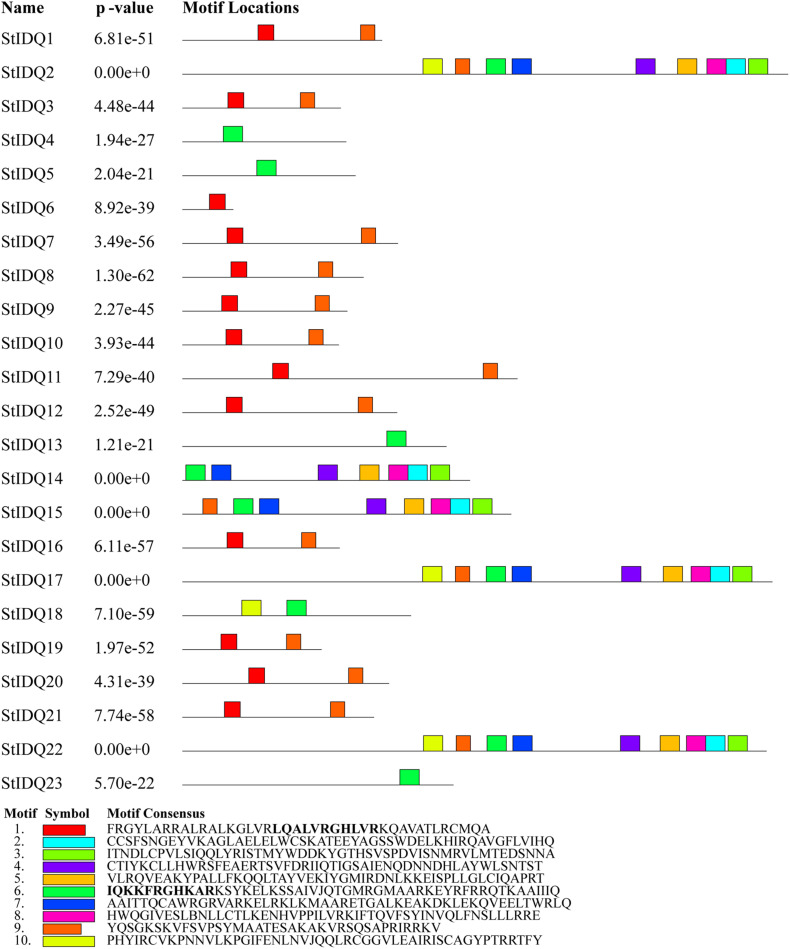
The motifs in IQD proteins of *Solanum tuberosum* L. (StIQD). Each motif was represented by different colored box. The sequence of each motif is listed below with the same color.

**FIGURE 2:**
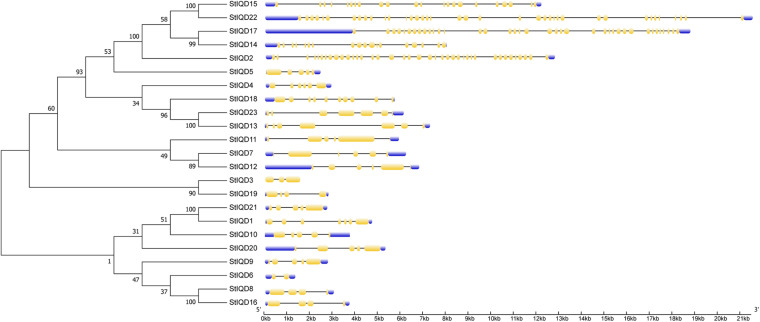
Phylogenetic analysis and exon–intron organization of *StIQD*. Maximum likelihood trees of full-length amino acid sequences encoded by StIQD (left) and exon–intron organization of *StIQD* (right). Exons: yellow boxes; introns: black lines; Untranslated regions: blue box.

The MEME suite was used to identify the conserved domains of the 23 StIQD protein sequences. Motifs I and VI were the most common in StIQD protein sequences, occurring 13 and 10 times, respectively. In addition, we found that the 23 StIQD sequences contained either Motif I or Motif VI. However, the motifs did not occur together in a single StIQD sequence. The motif I contained the IQ67 motif (LQXXXRXXXXR), whereas motif VI contained the relaxed version of the IQ67 motif (IQXXXRGXXXR) (Figure 1). Prediction of the CaM-binding sites of each StIQD revealed that all StIQD proteins contained at least one string of high-scoring regions that indicated the location of the putative CaM interaction sites (Table 2). Evolutionary relationships of the StIQDs based on the 23 StIQD protein sequences revealed that they could be roughly categorized into two subfamilies: subfamilies I and II (Figure 2). The 23 StIQDs formed seven sister pairs with high bootstrap support (≥ 89%). The structural diversity of *StIQD* genes analyzed using their cDNA and genome sequences revealed that they had between 2 (*StIQD*6) and 38 (*StIQD*2, *StIQD*17, and *StIQD*22) exons. However, exons in subfamily I were more than those in subfamily II. Genes with the maximum number of exon cluster together. These results demonstrated the conservative nature of the motifs and the structural diversity of the genes.

**TABLE 2:** Predicted calmodulin-binding sites in StIQD protein sequences.

**Gene**	**Predicted calmodulin-binding sequence**	
StIQD1	307-KA**KVAAKKRENTL**AHA	
StIQD2	526-**QTYRAHKR**	
StIQD3	103-TRR**VKDAAATKI**QAV	
StIQD4	134-RGEIER**KYYQGL**TRRLA	
StIQD5	18-QA**LELKILLQAVKLQRWW**R CKLL	48-AAVVIQSHALGW **IAR**QRASRNK ERL**LQA**VLKLQ**RWWR** SKLL**HEQR**T
	130-WRGK**LLHKQRTKAA**VVIQ SHA	166-TLLAVLKLQRWWR GKLLHKRRT KSAVV
StIQD6	71-RGHLARRAFKA**LK**SLVR**LQA** VVRGAYVRRQA	
StIQD7	8-IKAVLFGKKSSKSHL	155-QAI**VRIQALARGRR**IR
StIQD8	123-QSYYRGYLARRA**LRAL**KGL	
StIQD9	29-RK**WKLWRSASGGI**AMA	117-VR**LQAIFRGRQV**RKQ
StIQD10	106-**AIRIQTAYRAHLARKALS AL**	
StIQD11	248-ARR**AQLKQ**KHIT	
StIQD12	437-**ESNKVNRR**	
StIQD13	562-VRKQY**KVCWAVGILEKV**VL RWR	
StIQD14	75-IASI**VTQC**RWRGRVA	
StIQD15	163-RQTKAAIIIQSHSRAFLARLK YKKLK**KAAITTQCAWRAR VARGELRKLKMA**ARETG	
StIQD16	113-QSYYRGYLARRALRALRGL VKLQALVRGHSVRKQ	164-VRA**RRLQL**LQSKVE
StIQD17	IIQR**QIRT**YIMR	YSYYRSLQRAAII
StIQD18	292-IILQS**FIRGEIERRL**YNT	
StIQD19	139-QA**LIRAQSRARLGRSMVF**E	
StIQD20	1-**MGKKGSWFSAI**	
StIQD21	147-QA**LLRVQARVREQRA**RL	
StIQD22	844-RKFLAYSKFKKLK**KAAITTQ** CAWRA	
StIQD23	352-RGFQVRRQY**RKITWSVGVL EKAIFRWRL**KRKG	

### Synteny Analysis of Potato With *Arabidopsis* and Tomato

**FIGURE 3:**
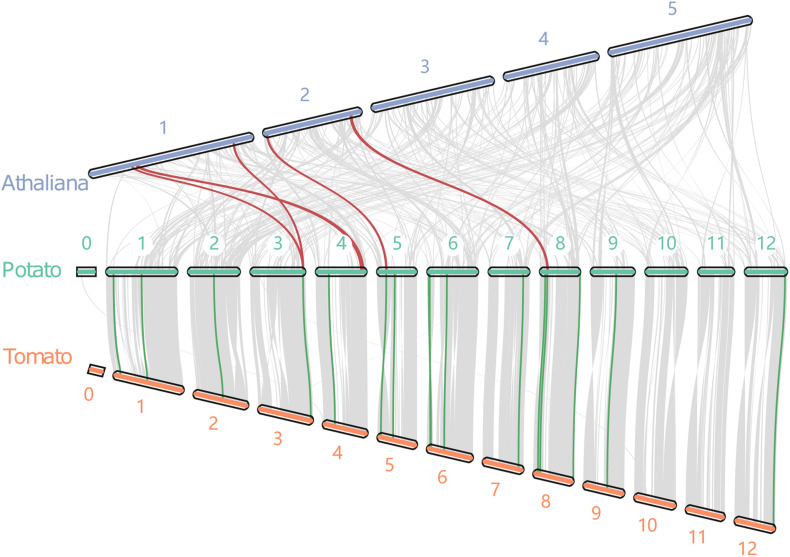
The collinear relationship between potato, tomato and *Arabidopsis*. Red and blue lines indicate the collinear gene pairs of potato and *Arabidopsis*, and potato and tomato, respectively.

The synteny blocks of potato with tomato and potato with *Arabidopsis* were analyzed further based on the total number of genes in potato, tomato, and *Arabidopsis*. There were 105 synteny blocks containing 18,988 pairs of synteny genes between the potato and tomato (Figure 3). The blocks contained nearly 50% of the total genes of the two genomes. Moreover, potato and *Arabidopsis* had 377 synteny blocks containing 4,872 pairs of syntenic genes. The blocks contained 10.6 and 15.6% of the total genes of potato and *Arabidopsis*, respectively. These results confirmed the evolutionary relationship results that found potato to be more closely related to the tomato (Xu et al., 2011). Further analysis of 5 pairs of synteny genes between potato and tomato and 16 pairs between potato and *Arabidopsis* revealed that three *StIQD*s (PGSC0003DMT400006376, PGSC0003DMT400041427, and PGSC0003DMT400045444) of potato showed synteny with those of *Arabidopsis* and tomato. These findings demonstrated the evolutionary conservatism and the important function of these genes. Interestingly, we found PGSC0003DMT400006376 showed synteny with AT1G17480.1 and AT1G72670.1, respectively, suggesting the possibility of a gene replication event.

### Phylogenetic Analysis of Potato *IQD* Genes

**FIGURE 4:**
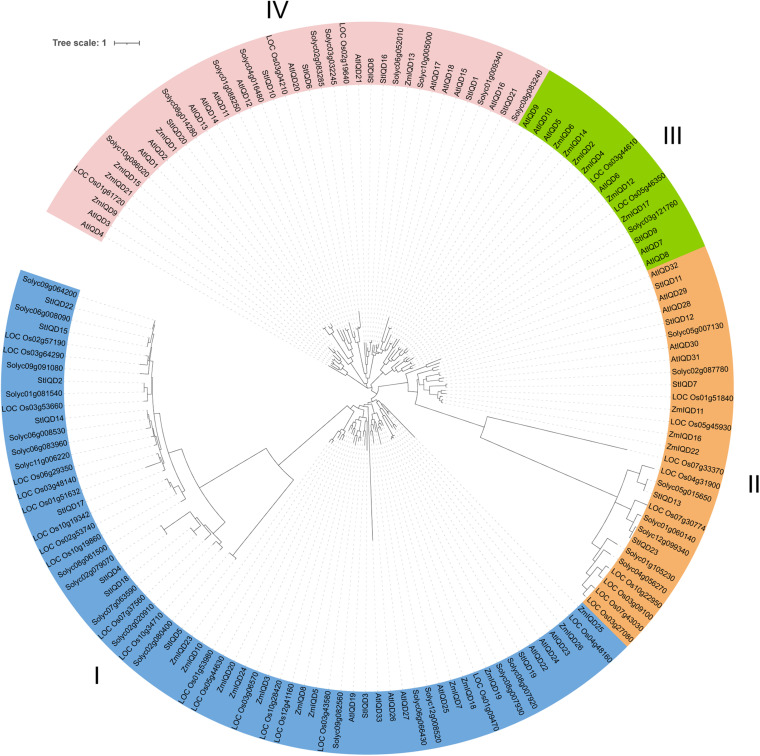
Phylogenetic relationships of IQDs in potato, *Arabidopsis*, tomato, rice, and maize.

The protein sequences of the IQDs from *Arabidopsis*, tomato, maize, and rice were analyzed using the maximum likelihood (bootstrap = 1,000) method to understand the phylogenetic relationship of StIQD in plants. The analyzed *IQD*s clustered into four distinct classes (I–IV), with the potato StIQDs clustering in all four classes (Figure 4). Class I had most of the IQDs (66), whereas class III had the least IQD members (16). All the IQD family genes of dicotyledons were more closely conserved than monocots. While, in the combined phylogenetic trees, classes I and IV contained one ortholog pair, respectively, formed by *Arabidopsis* and rice orthologs AtIQD20/LOC_Os03g04210, and potato and rice orthologs Solyc02g020910/LOC_Os10g34710. The *IQD* genes of potato were more closely related to these of tomato. These results were consistent with the evolutionary relationships among the five species.

### Expression Profiling of *StIQD* in Different Tissues

**FIGURE 5:**
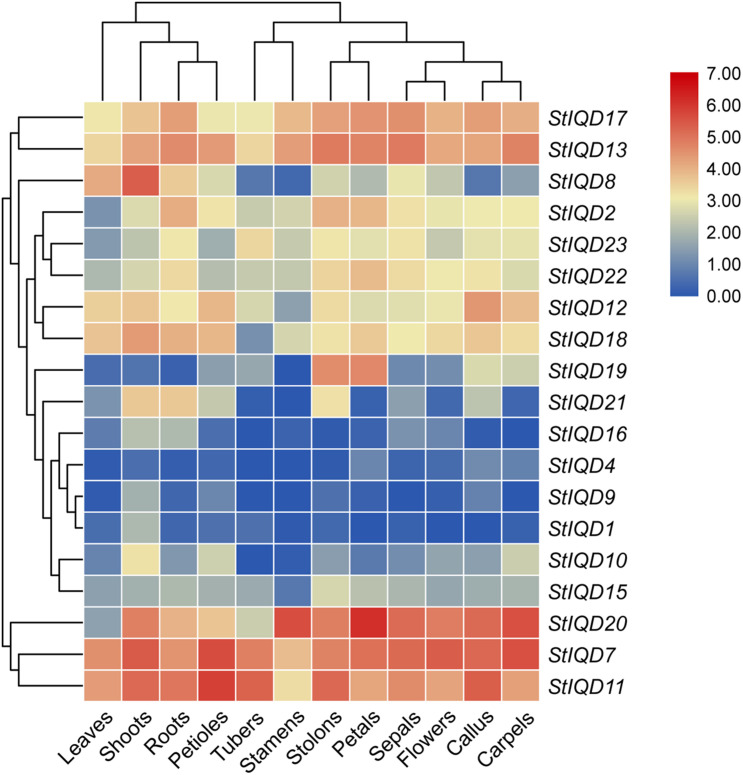
Hierarchical clustering of expression values of *StIQD* in 12 tissues. Red indicates high expression level; blue, low expression level.

The expression levels of the *StIQD*s in different tissues were analyzed using the publicly available RNA-Seq data to reveal the roles of *StIQD*s in potato (Xu et al., 2011). Among the 23 *StIQD* genes, 19 expressed at least one tissue (FPKM > 1). Nineteen were divided into two major groups (Figure 5). The first group was composed of *StIQD*7, *StIQD11*, and *StIQD20*. The three exhibited relatively high expression levels in most tissues. The remaining 16 *StIQD* genes formed the second group. The group contained eight high-expression genes (*StIQD*2, *StIQD*8, *StIQD*12, *StIQD*13, *StIQD*17, *StIQD*18, *StIQD*22, and *StIQD*23) and eight low-expression genes (*StIQD1*, *StIQD4*, *StIQD9*, *StIQD10*, *StIQD15*, *StIQD16*, *StIQD19*, and *StIQD21*). In addition, we found that the tissues could also be divided into two clusters: the tissues of vegetative tissues (leaves, shoots, roots, and petioles) and the tissues of reproductive tissues (tubers, stamens, stolons, petals, sepals, flowers, callus, carpels). In addition, we found some of the *StIQD* genes exhibited a tissue-specific expression. For instance, the average expression level of *StIQD20* in reproductive tissues is nearly three times (2.75-fold change) that in vegetative tissues. While the expression level of *StIQD8* in vegetative tissue was higher, more than six times (6.47-fold change) of that in reproductive tissue. These results indicated that the *StIQD* genes were tissue-specific. Furthermore, in order to reveal the molecular network of IQD family in cellular metabolism, we classified the detected genes into 15 coexpression modules for 12 different tissues, and 19 expressed IQDs belong to 7 modules (Supplementary Figure 2). Then, the coexpression genes were annotated using GO enrichment method. We found the signal and response to stress-related terms were enriched in the coexpressed modules, such as “signal transduction,” “single organism signaling,” “cellular response to stress,” “cellular response to stimulus,” “response to oxidative stress,” and “response to hormone.” These results suggest that the IQDs and their coexpressed genes may play an important role in the signal transduction and stress response.

### Identification of the *StIQD* Genes Related to Abiotic Stresses

**FIGURE 6:**
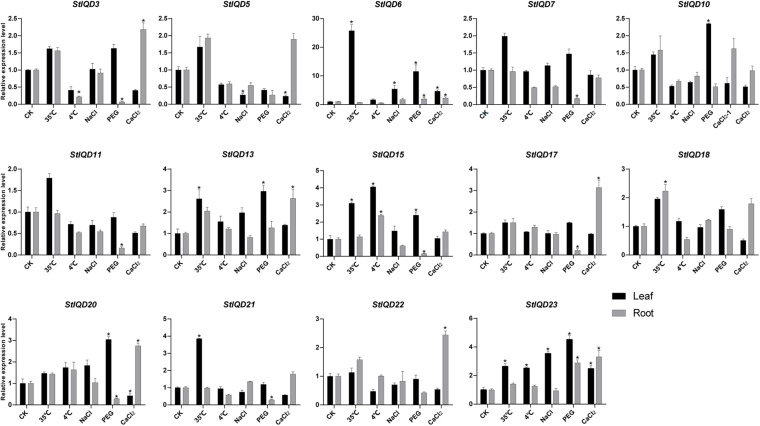
qRT-PCR analysis of *StIQD*s genes in response to heat, cold, NaCl, CaCl_2_, and PEG. Error bars represent standard error of the mean (*n* = 3). *P < 0.05.

Heat, cold, PEG, NaCl, and CaCl_2_ stresses were induced to explore the role of *StIQD* genes in abiotic stresses. Fifteen *StIQD* genes were cloned and analyzed by qRT-PCR. The expression of 14 of the 15 genes was either induced or suppressed under a specific stressor in specific tissues (Figure 6). Most of the genes were up-regulated in leaves but down-regulated in the roots. *StIQD6* and *StIQD23* participated in almost all stress responses. Notably, the expression of *StIQD6* increased nearly 30 times under heat stress (35°C). These findings suggested that *StIQD*6 and *StIQD23* played crucial roles in response to various stresses. Seven *StIQD* genes (*StIQD3*, *StIQD6*, *StIQD7*, *StIQD11*, *StIQD15*, *StIQD17*, *StIQD20*, and *StIQD21*) were down-regulated in the roots under PEG stress. Among them, *StIQD7*, *StIQD15*, *StIQD17*, and *StIQD21* responded only to PEG stress, indicating they played a negative role in response to PEG. *StIQD15* was up-regulated (2.41-fold change) in the leaf but down-regulated (0.15-fold change) in the root under PEG stress, indicating the diverse role of *StIQD15* in response to PEG stress. Besides, eight *StIQD* genes (*StIQD3*, *StIQD5*, *StIQD6*, *StIQD13*, *StIQD17*, *StIQD20*, *StIQD22*, and *StIQD23*) responded to calcium stimulation. The majority of the eight were up-regulated. *StIQD20* was up-regulated (2.75-fold change) in the root, whereas it was down-regulated (0.42-fold change) in the leaf. These results demonstrated that *StIQD* genes play crucial roles in multiple stresses and exhibit different responses in various organizations.

### Analysis of *cis*-Acting Elements of SOD Gene Promoters

**FIGURE 7:**
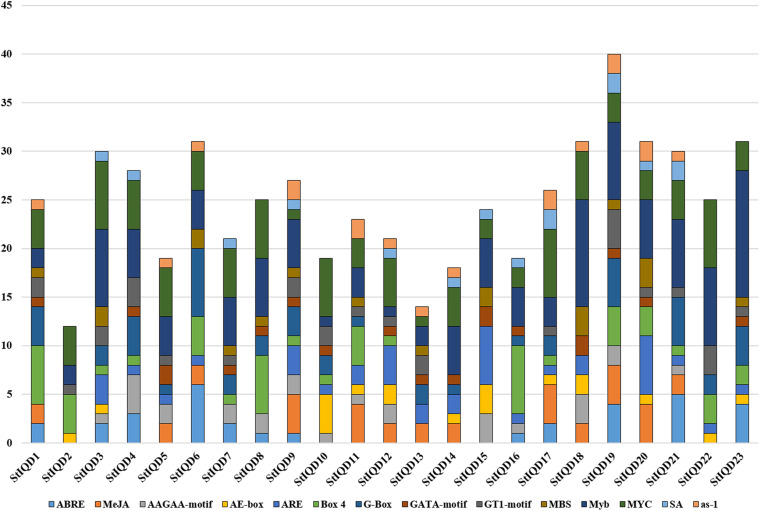
The *cis*-acting elements of putative IQD promoters predicted by PlantCARE. The different *cis*-elements are present with different colors.

The *cis*-acting elements play an important role in cell regulatory network. To explore the potential roles of the potato IQD genes under environmental stresses, the IQD gene promoters coupled with 2-kb genomic sequences upstream of the transcriptional start site were analyzed by PlantCARE online tool. The IQD family genes possessed a variety of elements, including the hormone-related responsive elements: ABA (ABRE), MeJA, and salicylic acid; the abiotic stress-related responsive elements: MBS (Drought) and ARE (anaerobic induction); and the light response elements: AE-box, Box 4, G-box, GATA motif, and GT1 motif (Figure 7). Among them, we found 13 of 23 IQD family genes contained MBS elements. For instance, *StIQD6*, which contained two MBS elements could be up-regulated in four abiotic stresses (heat, NaCl, PEG, and CaCl_2_), suggesting the two MBS elements may contribute to the stress response. Further, 10 of the above 13 IQDs were analyzed by qRT-PCR and found that 8 (*StIQD3*, *StIQD6*, *StIQD7*, *StIQD11*, *StIQD13*, *StIQD15*, *StIQD20*, *StIQD23*) of which could be responsive to the PEG stress (Figure 6). These results suggested that IQD family genes play important roles in abiotic stresses and might be related to hormone stimuli.

### Protein–Protein Interaction Analysis

**FIGURE 8:**
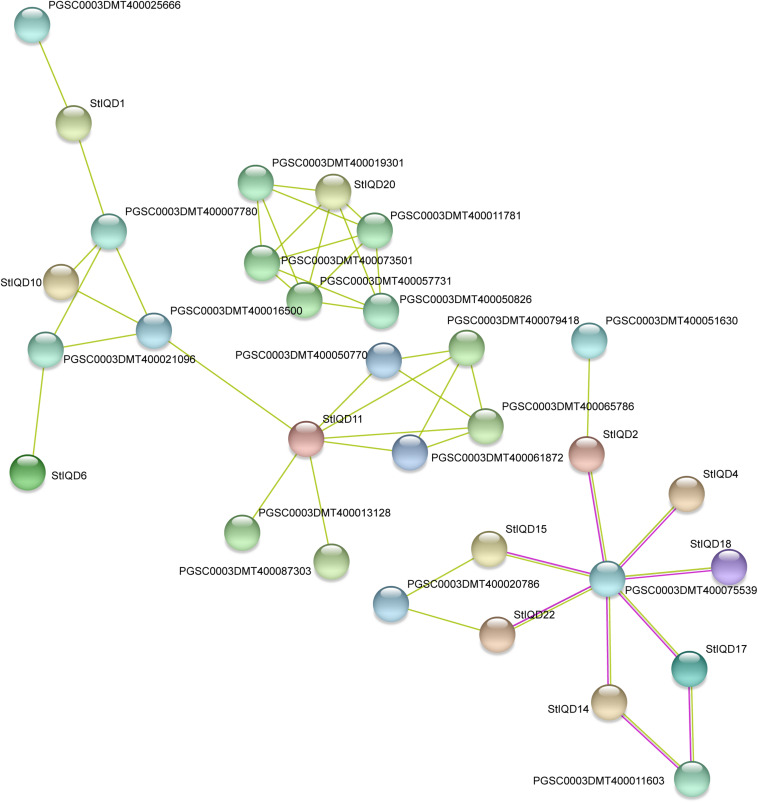
The protein-protein interaction network of StIQDs detected by STRING.

In order to identify the function of IQD genes, the PPI networks of IQD protein were built by using the STRING database. The results showed that the PPI networks can be divided into three groups and contained 12 StIQDs (Figure 8). StIQD11 was predicted to interact with most proteins (six proteins), in which two encoded the amine oxidase, which play important roles in plant development and stress response. StIQD20 was predicted to interact with the five proteins, in which two belong to the NB-LRR family proteins (PGSC0003DMT400019301 and PGSC0003DMT400050826). This indicates that StIQDs may be involved in abiotic stress responses. In addition, we found the gene PGSC0003DMT400075539, which encodes an exocyst complex component and implicated in tethering secretory vesicles to the plasma membrane (Heider and Munson, 2012), was predicted to interact with seven StIQDs, and these IQDs contained the myosin-related motif, such as the “Myosin_head” and “Myosin_N,” indicating these StIQDs may participate in the vesicle transport. These results suggest that the StIQDs may interact with these proteins to take part in potato development.

## Discussion

Plants evolve many specific gene families to adapt to environmental changes because of the specificity of plant growth. *IQD* genes are among the specific gene families that are widespread in photosynthetic plants (Abel et al., 2013). Previous studies postulate that *IQD* participate in various crucial biological processes in plants (Bürstenbinder et al., 2017a,b). Herein, bioinformatics analyses were employed to analyze the *IQDs* gene family of potato at the whole-genome level.

There were 23 non-redundant *IQD* genes identified in the potato genome. This number was not significantly different from that of *Arabidopsis*, which has 33 *IQD* genes (Abel et al., 2005). Although the proteins all belong to the StIQDs, the length, molecular weight, and pI of proteins showed a significant difference. For instance, the StIQD6 contained only 131 amino acid residues and contained only 1 IQD domain, whereas the StIDQ2 contained 1,571 amino acid residues and contained not only IQD domains but also the myosin-related domains (Myosin_N, MYSc, and DIL). The diversity of gene sequence length leads to the diversity of gene function. For an amino acid, the pI is the average of *pKa* values for the amine and the carboxyl group, which are the pH value at which the molecule carries no electrical charge (Moldoveanu and David, 2017). We found the lowest pI of StIQD was 5.5 (StIQD11), whereas the highest pI was 12.52 (StIQD6), indicating the complex properties of StIQDs.

The *IQDs* genes of potato were divided into two classes: the conventional IQ67 motif (LQXXXRXXXXR) and the more relaxed version IQ67 motif (IQXXXRGXXXR). In *Arabidopsis*, four IQ67 motifs have been identified. They include [IQXXXRGXXXR or (ILV) QXXXRXXXX (R, K)], the 1-5-10 motif [(FILVW)X3(FILV)X4(FILVW)], and the 1-8-14 motif [(FILVW)X6(FAILVW)X5(FILVW)] (Abel et al., 2005). The conventional IQ67 mediates calmodulin retention in a Ca^2+^-independent manner. Besides, the 1-5-10 motif and the 1-8-14 motif function in a Ca^2+^-dependent manner (Rhoads and Friedberg, 1997; Choi et al., 2002). Nonetheless, the difference between the conventional IQ67 motif and the relaxed version IQ67 motif has not been reported. Among of the 33 StIQD proteins encoded by 23 *StIQD*s, only one transcript of each gene predicting the calmodulin-binding site motif was consistent with the IQ67. These findings suggest that the alternative splicing events of genes lead to the diversity pattern of calmodulin-binding sites, which may be related to the functional diversity of the *StIQD*.

A comparative genomic analysis of plant IQD members from two dicotyledonous (*Arabidopsis* and tomato) and two monocotyledonous (maize and rice) plants was performed to explore the phylogenetic relationship of the IQDs. The phylogenetic analysis categorized the IQDs from the five plants into four distinct classes. IQD proteins in the same class were closer to each other than proteins from the same species in different classes. Nonetheless, the closeness was more between members of one family in the same class than others. Class IV did not contain IQD gene from *Arabidopsis* and maize. This finding suggests that the IQDs in this class evolve after species differentiation and may play a significant role in species generation. Two orthologous IQD pairs between rice and tomato (three) and rice and potato (one) were identified. These results indicated that the IQDs possibly exist before the dicotyledon–monocotyledon division. Synteny analysis is an important method for identity phylogenetic character (Tang et al., 2008). In this study, we found nearly 50% potato and tomato genes showed synteny. However, only 10.6% of potato genes and 15.6% of *Arabidopsis* genes showed synteny. These findings suggest that potatoes are more closely related to tomatoes.

Previous studies postulated that the *IQD* genes play an important role in plant development and stress response (Tang et al., 2008; Sugiyama et al., 2017; Yang et al., 2019). In this study, we found the expression of the *StIQD* had a close relationship with the type of organization that vegetative tissue and reproductive tissue were clearly divided into two classes. The tissue-specific expression patterns of *IQD* genes have also been reported in other plants such as poplar, tomato, and soybean (Huang et al., 2013; Feng et al., 2014; Ma et al., 2014). *StIQD20* was highly expressed in the reproductive tissues but low in the vegetative tissues. In contrast, *StIQD5* was higher in vegetative tissues but lower in reproductive tissues. The homolog gene of *StIQD20* in *Arabidopsis*, *AtIQD13*, regulates xylem cell-wall deposition (Sugiyama et al., 2017). The differential function of this homolog pair is attributed to species specificity. In addition, 9 of 14 *StIQD*s could respond to at least two abiotic stresses among the five tested, indicating the *StIQD*s play important roles in plant response to stress. In *Populus* leaves, many *IQD* genes were up-regulated by PEG stress. Similarly, the *BrIQD5* of Chinese cabbage is also significantly induced by PEG (Ma et al., 2014; Yuan et al., 2019). Herein, most of the studied *StIQD*s responded to PEG treatment. However, the regulation pattern was different in the leaf and roots; some were up-regulated in the leaf but down-regulated in the root. The differential expression was attributed to the different ways roots and leaves respond to drought stress. Hence, the specific mechanism should be studied further. Most *StIQD*s responded to heat stress than cold stress. Notably, *StIQD6* was up-regulated by > 25-fold after heat treatment, suggesting the crucial roles of *StIQD*s in heat stress than in freezing stress. Ca^2+^ is a pivotal cytosolic second messenger, which plays a prominent role in plant growth and development, and the CaM has been extensively studied (Ng et al., 2001). When exposed to CaCl_2_, we found eight *StIQD*s were differently expressed, and most of which were up-regulated in leaves and roots. This result suggests that these StIQDs interact with CaM to transmit calcium signals at the protein level and respond to Ca^2+^ stress at the transcription level.

In conclusion, 23 *IQD* genes were identified in potato. These genes had diverse exon/intron structures, motif distribution, and phylogenetic relationships that potentially contributed to their diverse functions. RNA-Seq data analyses revealed that some of the *StIQD*s were expressed in a tissue-specific pattern and thus were strongly related to tissue development. qRT-PCR results further confirmed that *StIQD*s responded differently to various abiotic stresses, including drought, extreme temperature, and CaCl_2_ treatment. This study provides baseline information for further studies on IQD proteins in potato.

## Data Availability Statement

The original contributions presented in the study are included in the article/Supplementary Material, further inquiries can be directed to the corresponding author/s.

## Author Contributions

CM, YL, and XD performed the experiments, analyzed the data, and wrote the manuscript. CM and RF conceived the research and revised the manuscript. All the authors read and approved the final manuscript.

## Conflict of Interest

The authors declare that the research was conducted in the absence of any commercial or financial relationships that could be construed as a potential conflict of interest.

## Publisher’s Note

All claims expressed in this article are solely those of the authors and do not necessarily represent those of their affiliated organizations, or those of the publisher, the editors and the reviewers. Any product that may be evaluated in this article, or claim that may be made by its manufacturer, is not guaranteed or endorsed by the publisher.

## Footnotes

1
http://www.arabidopsis.org

2
http://solanaceae.plantbiology.msu.edu

3
http://hmmer.org/

4
https://web.expasy.org/protparam/

5
http://mg2c.iask.in/mg2c_v2.1/

6
https://github.com/tanghaibao/jcvi/wiki/MCscan

7
http://mev.tm4.org/

## Supplementary Material

Supplementary Figure 1 The chromosome location of the *IQD* gene family in potato.

Supplementary Figure 2 The coexpression gene module of expressed genes in 12 tissues.

Supplementary Table 1 Primers used in this study.

Supplementary Table 2 The sequences (gene, corresponding coding sequence, proteins) of *StIQD*s.
