# Supplementary figures and images for: Genome-Wide Identification and Characterization of the Potato IQD Family During Development and Stress
Source: Front Genet. 2021 Jul 27;12:693936. doi: 10.3389/fgene.2021.693936 (PMC8354571; doi:10.3389/fgene.2021.693936)

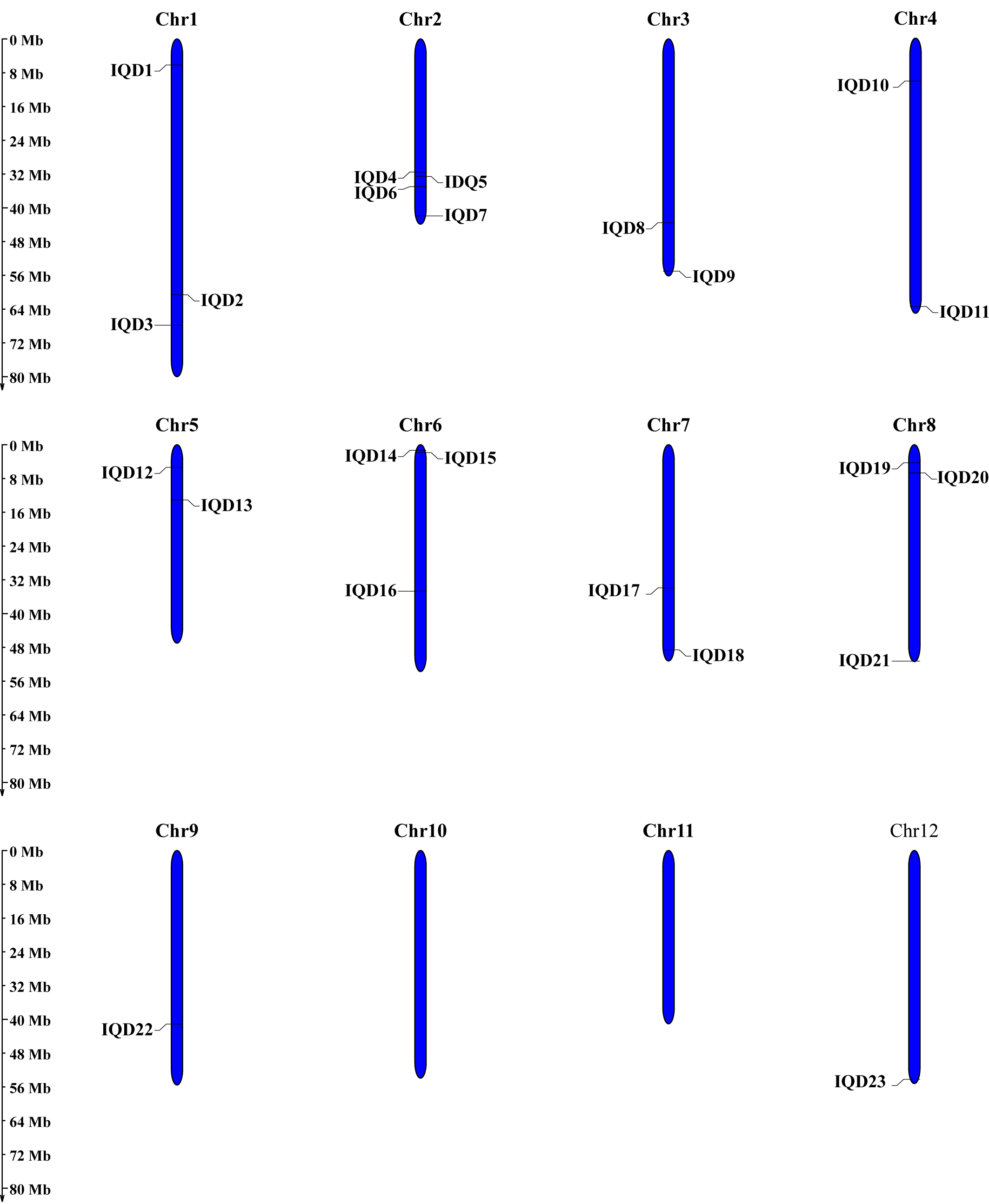

Supplement: Supplementary Figure 1 — The chromosome location of the IQD gene family in potato. [file Image_1.TIF]

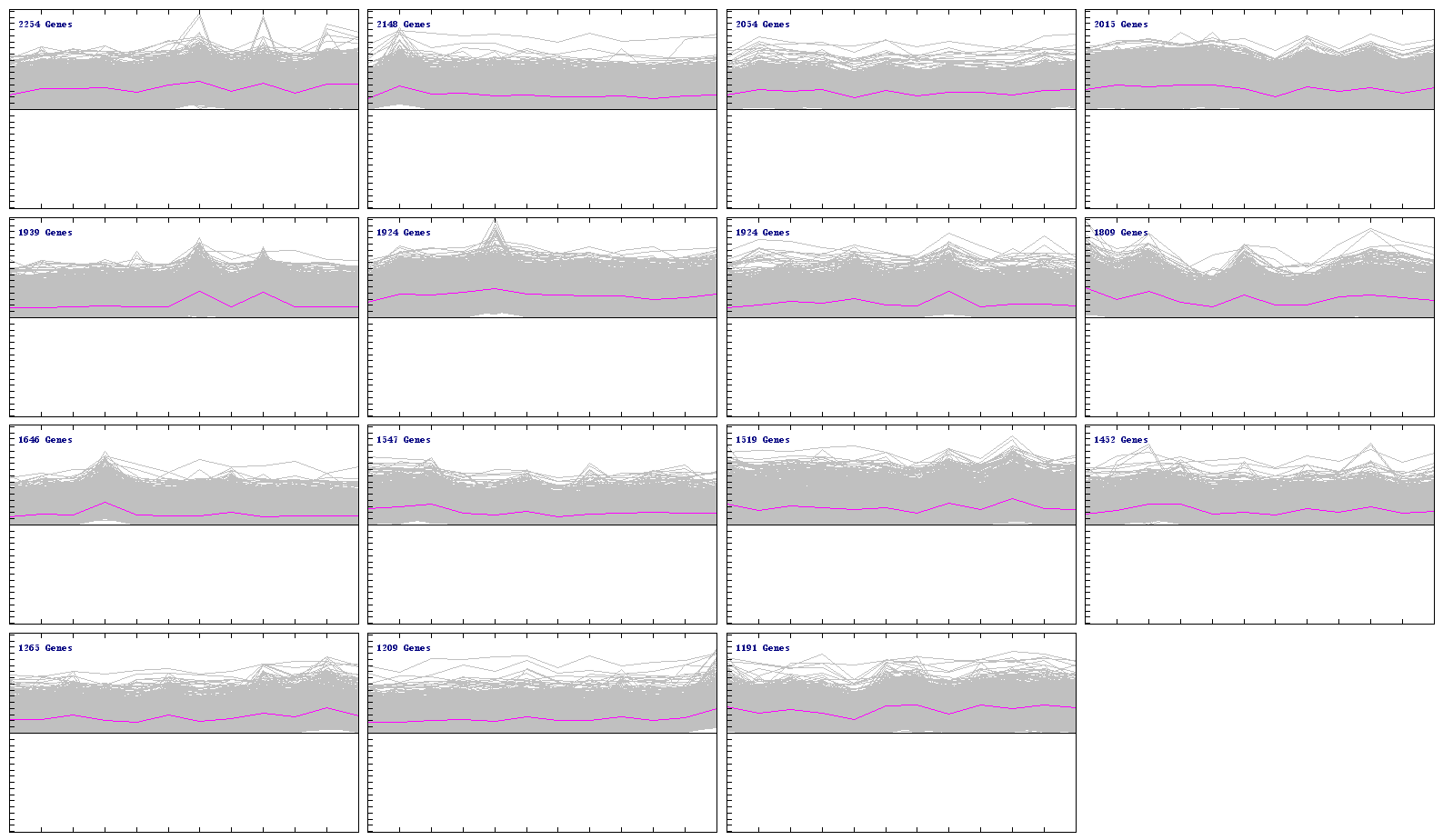

Supplement: Supplementary Figure 2 — The coexpression gene module of expressed genes in 12 tissues. [file Image_2.TIFF]
