# Supplementary material for: Genome-Wide Identification and Characterization of the Potato IQD Family During Development and Stress
Source: Front Genet. 2021 Jul 27;12:693936. doi: 10.3389/fgene.2021.693936 (PMC8354571; doi:10.3389/fgene.2021.693936)
Supplement: Supplementary Table 2 — The sequences (gene, corresponding coding sequence, proteins) of StIQDs. [file Data_Sheet_1.PDF]

####Gene
>PGSC0003DMG400009086
CTTAATCCCAAACAACATTACACTTATCATATATTACATCCATTGCAAAAACTCAAACTTCATACATATTTAACCA
TCCAAATTAACTTCAAAATAGGATACAGAAACAAAAAACAATAAGCTATTAGCACACAGCAACAGCATTTTTT
ATCAGTACTTTATTTACCACCAAAAAAAAAAAAAATCAACTAAAATTATATCTCCAGGGATGTGGTCCTAATGGT
GAAGCCCGATCATCGCCACTTGATTCCGGACTGTAAGTTGCCATCCATTTATTAGCATGTCCACTACTGCGATT
CGCGGTAGTTTTTGGGCTTGGGCTTCTTGAGACTTGATAGATTGCTGTTGTACTTGCCCCTGAACTTGATTCA
AGGCCCAAACCATAGTGTGCTCCCTTCCTTTTCGATGCATTCCATTGGGCCGATGGTGGGCTACGATGCTTAA
CAGGGCCTTGATTTCTCACCTTGGCTTTTGCAGACTTAGTTGGGGTCATGTAGCTAGGGACATTATCAAATTT
TGAGTGACCAACCTTCCGGGCTGAGTATGGGCTTGTTTCAGCTTGATCAAAAGTGTGACGGGCTGAGTTGA
TGGTCTCCGAGTCCAATGGCCCGGTGATTAGGTCCATTTCAACTGTCTTTTCAGACATGTCACTTGTTGTGGA
AGGTGTCACATAGGAGCTGTCTCTTGGCACAATGTGTCTTGAGTATTGGTATGGTTGTGAAGCCATCCATCGC
TCCAGCCAACTCCAACCCCATTGTGACTTTTCGCGCTCATTGCCAAAAAATTCAGCGTCATCACCATGTGGAT
CAATGTGCAATAGTTGTTGTTGCTTTTGCTGTCAACACCAATGACAATTACCACAACATTAGAACTTATCAATT
GACTAAAATTTATAATTCATTTTAGTAAGGAGTTGATTTCTCAGATGGTCGTCACTCAACTAATAGTTATTCCA
GAAAAGTGACAACCATCTGAGAAATCAACGATTTTAGTAAGTTAGGAAATAGCTAACCTGATAGGCAAAGG
CATAAGCAAGAGCTCTTTCCCTTTTCATTTCAGCATCGAATTTTCTCCTACTGCTTTCTTGGATTTTGTCCATGC
TTTGGTTCCTGTTATCCCAACCTTCTGCCCCTGACTTCTTGATGGGGCTGGTGTGCTTGTGTTGTTGTTCTTCA
GCAGCGTGTCTTCGTGCTTCTTCTAGCTTGCTCTCGAGCCTCTCCTGGACTAACTGGAGCCTCCTTGCTCGTA
CTCTGCCCTGAACACGTACTAATGCTTGCATACATCTCATGGTCATTTGTGCTTGTTTTCGCACATTATGTCCTC
TCACTAGTGCTTGCAGCCTTACCAATCCTTTCAGAGCACGCAAAGCTCGTCTTGCCTACAAAATCAGTAAAAT
TAAAGATTATTGAGATAAAATTGAGTAACATTACTAACAGATTACATAAACAAAATTTTGTCTAGGATTTTATTT
TACCAGATATCCCCTGTAGTATGACTGAATAAGCGTGGCAGCCCTTTCCTCTGTAGACTGCAGGAGGCCATAG
CCACCAGCTAATCTGACGACTTTGGCAGCCGCGTGAGCAGCTGCAACAGCTGCCTCAGCGGCTGCAGCAGT
GGCAGCTGCAACAGCAATGGCATGGTTATGATCCTCAGCTACAGTAGACGATGAATCAACGTTGCTCCCTCC
ATTACTAATAAGATCTGGAGAACTCCCTGCTGGAAAATGTTCAAGTGACACAACTTCAGGAGGTTCAGGTTG
CTGCCATTTGCTGTCTAATGTTTCCTTCTGAAAAACATGATACAACAATTAAACCACCTCAATCATCATGCCATT
AATTATCGCTTAATTAACATTAAAGACTCGAGATATGATATTTTTTTGACAAGAGATTGTAACATCAGAGTGAT
GGTAGTGAGTCATGATGGAGAAGTAACGTAAGCAGCACATGTAATTATGAATATAGGTGGGGAAGATATAGG
TGGAGTGAAAGCTAAAGGTCATGAACTCATTTGGTGCAAGGGGGGGGACCAGTAGTAACCAAAATTGAGT
AAATAACTATGCAATTTATATCTTGAAGAGGAAAAAGCCTATGGATTGCAGCTTTTGATGTACGCGATATGGG
ATAGACTTTGTCGTAACAATAATAACTATGCCTCAACACCAAACAAATTAATGTCCGCTCTATGAATTCTCACA
GATCATGTATCTCTAAATAAAAGGACAGATTTTATCAAAAGATAAAATGACACAGAGCAAGAGAAGCTAGTG
TAACTATAATATTGCTATCATTGGCATTTGTCTTATTAATAGTAAATAAATCCTCTTTTAGGGAACAACAATTATC
ATGGATCAACATAAGAGAAGAACACATTATTAGCAAATCAATGGTAATTTGGATCATAATGTTGAAAATCAAC
TAGGAACTAACTAAACATGTATAAACCAAAGAATATCTTGCTCTAAAATATACTATAGTGGTATCAAAATTCTGT
TTTTGTTCTTCTTTTTCTCTGTATTGATCTTGAAGATCATAAGACGAGTAATAGAGTCAGAATTTTCACTAATGA
TGTTTAAGATATGAAAAACGAAGAAGTCAAGTAGATTAAACATATATACGAGGTGTTCGAATGAACCCCTTAC
CCCTGCAAAAGATGTGAAAGAAAAACACTAGAGATTGAGCTAACCTTCTTGTCTGGTGAATCTTTAGGAGAC
TGTTTAAAAACTTTCTTCACAGATGAAAACCAACCACTTCCTTTCTTGCCCATATTCAGCCAAAAGAATTGAC
CTTTCTGTCACAAAAAACATACACATTCAGCAGATCATAACCATCAAGACAACAACAAAAAAAACAGAACTT
TCCCAAGTAAGTTGCTGAAGAAGAAAGAAAGAAAAAGTGATTGGAGAAAAATAGTTTGGTGATAATAAAA
AGAGTAGTAAGTGCAAACCTGCTTATTTTGCCGTTAGTATTTATCATTCTCACGGGGTCAGATCAAGAAGAGT
ACCTTTCCAATTGTTTA

>PGSC0003DMG400019610
CAAGAAGAAAATGAAGATTTATTTCTAGTGTACTGAACATATATAATTATGTCATAACATTTGCATATTTAAAAT
AACCATTTATTTGAACTTGTGCAAGAATATCTAATTATAATTATATCTGCAAGCAGGGGCTCCCATAGGTGAGG
CCCTGTCGTGTACACGTGTTTCTGGGCTATAGGTTGCCATCCATTTAGCAGAAGTTTTTGGGTTTGGGCTTCT
CGCAGATGAATCATAGCTCCACCTACGGGCTGTCACCTTCTTCGGTGTTGCATTCCATTGGGCCACTCCTGGT
GGGCTTTTAGGTTTTATTGGGCCTGGGCTTCTTATTTTTGCTTTTGCAGATTTAGTTGGAGCCATGTAGCTAG
GGACAGAATCCATGTTGGACTGAGTGGATGAATATGGGCTTCTTTCAACTGGACTGAGAATGTGATGGGCTA
AGTTGACATCTTCTGAGCCCAATGGGTCCAATTCTACTGTCTTCTCTGATATGTCATCTGTGATGGACAATGAC
ACATAGGAGCCTTCTCGTCGCACAGCATGTTGAGAAGCCATCCATCTATCAAGCCAATTCCAACTTTGTTGTG
GATTTTCATGCTCATTGCAAAAACATTCTTCATCATCATCTGGATCAGAGTGCAACCATTTCTGTTGTTGCTAA
CATATAATTAGCCAATACAAATTAAGAGCATGGAGTTTAAGGGGGAAAATGACATGTTTTAAACTTCTAAGCT
TTAGCGGCAATTAATTAACAACAAATGTTTAATTATTGTTAAATATGTTTTTTTAGATGCGATTACTAATGACAA
TTACATTTTAGCACTCTTTGTAAATGCTGTAAAAATATATAATGACATTGAATTTAATGACAATTAACTAATGTG
GTAAATAGTAACACTACTCTTTGTCAGTGTGCATATTATTGTCACTCTAAGCTGTATTTATTGTAGTGATATGTCA
AAATATTTGTGGTGGTTTATAGAACTTTTGAGATTCAACATGTCATAGTATTTGTGCAAGTATAAAAACTCCCT
GTCAAGGATTAAATGAGAAGTGCTATGTTAATTATTTCTTATATACACCATTAATTAGCTATATATCAATATTATTA
ATCTTTGTATAACAATATTTGCGTCATACTTAGTCCTGCATGAACCAAACGACCCCGAGTGATTAGTGCATCTA
ATGTGAAGGTAGTTGCACTACATTAGTACTCCCTCTGTAACTCATTTTCCTTTTTAGTAGTTCAAAAGAAAATG
ATACATTTCTATATTAAATAATAATTTAATTTTAAAATGTTTATTTTACCCTTATATGAAATGATTTATAGCCAAAC
AATCATCTATCATTTATTTTAGATCATAAATTTCAAAAGTTTTTCTTTTTTAAACTTTGTCAAATCAAACAAGCTC
ATATAAAATGGAACGGAGGGAGTAAATATATATGACAACTTTTGCTATGTAAAGGCTAACCTGATAAGCGTGT
TGTTTTCTTCTAGTGTTTTGTAGGATCTTTTCAATGCTTTGGTTCCTGTTATCCCAGCCTTCTGTCTCTTGTTTG
TTAGACAGGCTCTTGTTCTTGTGCTGGTCTTCTCTAATAATCGAGTGCAATTTTACTTCTTCCACTTTGCTCTG
CAGCAACTGGAGTCTTCGTGCACGTACTTTTGACTGTACCCGTACTAATGCTTGCATACATCTCATTGTTATTT
GCGCTTGTTTCCGCACACTATGCCCCCTCACTAGCGCTTGCAGCTTCACCAATCCCCTCAGGGCACGCAAAG
CACGTCTTGCCTGCAAAATTAGGCAAAATGTATATTTGAACACTACTTATATATAAGATGGATGATTGGCTTTT
AATTTACCAGATATCCCCTGTAATATGATTGAATAAGGGTGGCAGCAATTTCCTCTTTGTTGTATCCATCTAATT
TCACAATTTTTGGGGCCGTATAAGTTGCTACCGCAACACTGGCTTCGATGACATGAATATCATGATTTCGATCT
TCAGCCAACGAAGATGATGAATTGTCATTGCTCTCTCGATTATTGGTAAGATCTGAAGAACTCTCTGCTGGAA
ATTGTTCAACTTCTGGAGCTTCATCTTGCCATTGGTTCTCTAATTTTTCCTTCTGATAACCATACAAAATCACTT
CAATTGAAGGCTCTGAATTCTTAAACTTGTCCAGTAAATTACATTTACACACTTGAACTGGTGTTTATTTTGAT
GAATAATTAAAGACATGAGAGTATTAAATTTTTGGGAAGATTCTAGACGCGTTCTCAAACATCCATTATATAAG
TAAGTCAACCATTTAAGTATGCCATCTTTTTTAATCATATGCAATAGTCTCAATAAACCGTACGTAAAACTTCAT
ACATGTTTCTGATATGTATAATTATAGGAGACATATTTTATGTGATTAATTTATTTACTTAATGGTGTTTGAGAAT
ACACGCAAAATATAAGCTAGGAGTGTCTAATTATTATTTTTTATCATGTGTTATAATGCTACTATATACACAAAAT
TATCATATATTATCAACGCAATCACATTGGAAGCCTTGCGATATTGAAGAGAGATATACTTGACTTCTTACGAG
TATATTTTAAATAAATGATGTCATATTACCTAAAAAAAGTTATCACTTCATCTATTTGTTTAATAATGTCGAGAAC
TAGTAGTAGAAAAGGAAGGGAAGCGTGTAGTTATGTATGGTTAAAAAACGAACAATCGAATCAATAAATGTG
TGTTATTATAAGGTATATAAATAAAAAATCAAACAATTAAACCAAAATTGCTAACAATCAAATCAATAAATATTT
ATTATTTGATTTGAATAACTTAAATATCGATTAAATTAATTTATTTTAATTGAAATCCAAACCAAATCGAACCGT
GAGTACGTTTGATAGAAGAGGTGGATGAAAGCAACAAGTCATAAATTCTACGTTATTGCCTTTCGACAGGGG
CCCTATTTTAGAGGGGACCAATATGTGTTCAAGATTATTAGTTAATGCCGTTTAGTGAGGGGGAAAAAATGG
CTAACCAGTAGAAACTACGCAGGTCGATTTACCACAGATCCAGAAAAAGAGATGATCATATACAAAAATATGT
TGCAATTTTTTTGATCATATAATTATATACCAATCTAAATTTTAATGCGCGGATTATGTATACAATATTTTTATAATT

AGATCACTTAAAAGGTAATTGCAAAACTATAGTAAACTATTGCTATTATACTGTTAATGTGTATAACTTAGATCT
GAGTAGTATTGCTTCCATTTGTATAATCTGATACTAACAATGCCAATCTATCAAATTAACTATCCTTTTGGAAGG
AATAGAAGCAAACTAAACTAAAAATCATCGATAATTTCGTTTTAATCCTATGCAATATTTATCGATGAGAAGAG
TGAATAACTAACCTTTTTATGATCTGCTGGTGAATAATCCTTAGAAGAAGGTTTAATAAAGACTTTCTTCACAG
TGGAAAACCAACTACCACTTCCTTTCTTCCCCATTTTCAGCCAAATAAATTTATTTTCTGCCTGGAAACATTCA
ATTCAGAATATTGGAAAAAGAAAAAAAATAGCAATGAATTAATCAAAAGGAAAAAAAAACATGGGAGTATG
ATCTCTGTGTACCATAATATATTATGAATGAGAAAAATGAAGAATTAATAAGTGGGGTATTCTGCAGAAAGAAT
GTACCAG
>PGSC0003DMG400003665
CAAAAATCAAACCTATAATAAGATCTAAAAGAAAATACGATATGCAAGTACAACACTGGAAACATTTGTTAGT
TGCAATACCCAAGTTCTAAACAAGAATTAGACGTACAACATTACAAGTCATGTTCAGGCACTCTGACTCAGTA
ATTCCTTCCATCCTATGATCTGATTCCCCTATAATTAATTTTCGTTGAATTGCATCTGAAAATTGGTCAAGAATTT
AACTTCTATACGTACTTTGACGGACAGATCACCTAAAAAAGATACTTTATCAATTCAATGGTCATTGCACTTGC
TGAGGAGTTGCCTAGCTCGGACAGTAACTTGTAACCGTGCAAGGGCGTGCATGCAATGCAGAGCTATCCTT
GCTTGTCTTCTCACATATGCCCCTCTTACCACTGCTTGAAGCCTCACAAGGCTTTTCAGTGCCTTAAACGCCC
GTCTCGCCTAAAATATTGAATCAACAACACATGTATAAACAAATGGTCACACTACTAAAAGAAACACTATTTTT
CCACCGAAATTTCCCATTGAAAAATGTTCAACTAACTATTTCCACAGATATTTTGATCGAATCGATGTGAAAGA
AAAATAGTAATGTAATTATTGTTTCATATGGAAAAGTAAGGAAGCTTCCCAATATTTTAGTGAGAATATGTTTC
CCACCATGCATTTTTCCAGTGAGCTGGTTTGGTGGGAAATGAATGGAAAAATCATGTTCTATTAGCACAATTT
TCACTGATAGTGAGTAATATCAAATTAAAAGAAATTAAAGAAAAATATGATGATTGATCAAAGTTGGAAAAAC
TTACAAGATGACCCCTAAAATGAGATTGGATAGTAATAGCAGCCATATCCTCTTTAGACATAAAATAGATGTGC
CCATTATTTTTCTTTGTAGATGACCGTCCATTGGCGCTGCTGGTCCTATTGGTAATTGTGTTGTTATGAAGAAC
AATGATATTTTTATGTGGAGGAGATGACCTGAATAGTTTCTTTCTGATATTACCAAACCAATTCTTCTTAGACAT
CCTCATCTTTGATACTACAAATTAATACTGTTAGCCTTTTTTTGTCAAACTCAATTCTGGCAGAGATACTGAATT
GGACAGATTTTATGTGTGTTATACTAACATTGTGGAAATAAGGAAGCGTGGGCAACATGTTTGTTAGTAAAG
TAAGAGAATGGAACAATAGGTCTACTAGAACGTGGCAAGATGAAATTGATTTTGTGTAACTTTTAAGTATTTC
AAAGGACCATTCCACCACCTGGGCAGGGGCCACTAATGCTTGAACATATACCAACTAATACTTG
>PGSC0003DMG400002492
CAAATCCCAACATTTATCTTTAAGTTCCTTTAAGTTTTCAGACAGCATTTTCTAAAATTGTTCTGTCTGTTCTGT
TTTTCCTCAAAACAAAAACCATTTATCAAGCTGCAGAAACTGTATCAAGACCATATTTTTCAAGAATCGCTTA
AAAATGGGTGGTTCGGGGAAGTGGATTAAATCTTTAATCGGTTTAAAAAAGAATCAATCAAATGATTCTGTA
AGTTGAATTGGCTAATTCTAGTATACTCTGTTTCTGTTTTATTTTTTGTTGATGTTTTTTGATAATTTGAATTGTT
TTATTTGGGGGATTTTGCAGGAGAAGGGTAGTGGAAAGAATAGAAAATGGAAGCTGTGGAGGAGTGCATC
GGGTGGAATTGCTATGGCGTTTTCGAAAGGTGTAAAAGGGGGTGGGAATTTAGGAGATTCGGATGAATCTG
AATCTTCATTCCTGTCTGACAGTGCTTTGGCTGCTGCTATGGCTACTGTGATCAGAGCTCCGCACAAGGATTT
TGTGGTTGTGAAACAAGAATGGGCTGCTCTTCGAATTCAGGCTGCGTTTCGTGGTTTTCTGGTAATTTGAAT
GATTTGGGACCCAAAGTTGTTAACTTTTTTGTTTTACTTGGTTTAGCTTATTAATCCGTGTAGAAGATTAGTAG
ATAGCTAAGCATTGTCTTGCTTTTAGTTTTTTTTTTCATAAGTACTTGAAGCCTCTCTACCTCAACGAGGTAAG
GGTGAAGTCTGAGTACACTCTACCTTCCCCAGGCTCCATTCGTGGGATTTCACCGGGTATTATGTTCTTGTTG
TTGCTCTGTTTGTAATACTTAGATGTGACTATGAAAGAAATTGAATTTCTTGCATTTGGAGAAATTTTGTTGAA
AATTGAATCAGGAATAATTGCAATTCAAGAGGAACAGATCTAGATGAGTGTATAATTTGGAGGTAACTGCTT
GATTGTATGTTGAGTGACAAAAACTAGACCTATAACACCTTAAATCCTTTTTCTTTTTTTTTCTCTTGTCTGAG
ATGAGCTTAAATGGAGCGACAATATTAGTGTTGACTCGAGATTGAGGCATAGTTCTTTCTCTAAATGTAGAGA
TGTTGCCTTGTTATATGTCCTTGTAATATTTTCGCTCCCACTTGATGTTTTGCAAATGATTTAAATAGCTAATGA

GAAATTTTTATTGTTATGTCAAAAGGCAAGGCGGGCGTTAAGGGCACTCAAAGCAGTGGTTAGGCTACAAG
CTATATTTCGTGGGCGACAGGTGAGAAAGCAAGCTGATGTAACCCTCAAGTGTATGCAGACTCTTGTCAAGC
TGCAGTCTCGAGTTAGAGCCCGATGCCATCAAACATCCGTTGATGCTACACAAGGGTCTCTTGTTGATAGCC
AAGCTGATCCAATTAAGCAAGCTGAGGTATGTAGTTTCTCTATAACAGTACTGTTTGACCTGATACATATTTTT
TAGCTGCTATAGCGAAATATTGTTATAGAGAATATATTCATTGGTTCCAAAATCTGGTTGTTATAGTGAAATTTT
ATTACATTCCTGACTGCCATCGAGAAGTCTGACTGTATTATTCGTTGCTTCAAACTATCCAATTTTTCGCTTTCT
CAGGGTGGATGGTGTGATAGCCCTGGCACAGTGGATGAAGTGAGGTGTAAGTTAAAAATGAGACAAGTTG
GAGCAATTAAGAGGGAGAGGGCCATTGCATATGCCCAACAGGTATCTTGGTTAACTCCTTAAACATGTAATAT
GACACAAAATTTCTTTATACATGTAACTAACTGGCCTGAAAACACATTTAACTGATCTATTTCAGAAACTGAG
AACAAACCCCAGCCCGAATTCAAGAACAAGGAAAGTTGAAACACCGAATAAGTTCAAGGCTAATGGGGAT
TCAGTTTGGTTAGAACGTTGGATGGCGAGTAAGCCTTGGGAAAACAGACTGGTGGAAGATTTCCATACTGA
TGCATCAGGGATGACTCCGAGTTCTAGGAAGTATGAAGATTATGATGCTGGATCTTTCACTGACCGTAGTTCA
GTGAACATTAGGCGGAATAACATGTCTACCAGGATATCTACAAGAGGACCAATGAGTTGTCAAATTGCTAATT
CATCTTCTGAGCCTTATACTGATTATTATCAATATGATGATAGTACGACTTCTCATTCTTCCATATCGACTTCTGA
AACTCTTGGATCGAATCAGACACCTCCGGAAGAGGGTCATAGCAAGAAACCAAACTACATGAATCTCACAA
AGTCTATTAAGGCGAAGGTTAAGCAGAGGAATTCAAATTACTTGTCTCACAGTATGCAGAGGAATTCAACTG
ATAACTTACATGTCCATAGGAAGTCAAGTCCACTTTCGAGGACGATAGCAAGGAGAAGTGCTGATTGTGATC
TTTATTCAGTTGATCTGTGTAAGGATCTCTACCCTCCCTCAAATGCATTTTGATCATCTAATCTGGAAGACGAA
AGGCGATTGGTGTTATGAAAGGGGAGTTTTAGTGAGTTTTATTTGTCCATTTAGCTCAACAACTTGTTGTCTT
GATAGTAAAAATGAGTTTCTTTTGTTTACATAAATTTAGACTTAACCAATACATAATGCATTAGATGAGATTTTC
TCTAGAAAGCTGATTTACGAGTTGATTGTTGTGATGGTCACTCAACTAGTAGTAATTATCTCGGAAAGTGTGT
ATCATACTGTGTGCTTTTTGTTGATTGTGTAAGAGTTTCTGTTTGTTTTCGGACAACTTATTGGATGGATAATG
TTTTTG
>PGSC0003DMG400005774
TTCTAAAACAAACGAAAAATGCAGATTCAAGCGTTCAGGACATAACTTAGAACTTGAATCATCTCAATATATG
TGACAAAAACCACGTCCAAATTCATCCAGATCGCCACTAATAGGCACTCCAATCTTATCTAGTCTATCAGAACC
CGAACAAACACCTTTGCTAGTGAAATTGGCATAACAATTAGAACCCTTTTGCATGTTACTTCTTGAGTACCTC
TTATCATACTGAGACCTTAGTCTTGGTGCACTCATAGATCGCGTTTTTGCCTTAGCTGCCTCGGTGTAAGACAT
GTAATTTGGATGATTATTCGAGTATTCATCACTCGTGTAGCTTCGTGTACTACTCTTTGTTGGCGTAAATGGTCC
TGTCCTCGAACGAGTACATTTCGATGATGCTGTTGAGTGAAGTTGTGGGCTATTATCAATAGAGGAGTAACAT
GTTTCATCATAGCTAAAACAGAATTGCTCTGAATTTAAGCTAAATTCTGAAGAATGAAATAAATTCCTCTGTTT
GGCATTGGCATTGCCTATATGGTTTGAAGAATTCCTCTGCATTAATACAAACTATATGTTAGATAGATATAAGG
GGGGAAATGTACAAATTTACCTTTCTGCTATACGAAATATCTTAACTTTATCCTCTACTATACTTTAGGGTCATT
CATACCTTGAGCATAAACATTTGATCATTTTTCATGCTCCTTGCACGAATAATTTGCTCGAATTTATCAGGAGTT
GTAGGACCCTATATATCAAGATTCAAAATGATCAACTGACATGATCAGTCAAACAACATCATATATAAAATGTA
AAATGAAATGAAATGAGGAAGATGATGTGTTACTTACATGAGGAATAAATTGAGTAGACTTCGCGCTAAAAG
GAGGTGATTCAAAAACCATAGATCGTCCTAAACGAGCTCTCGATTGAGCCCTTATCAACGCTTGCATACGCCT
TAGCATATCCGCGGTTTGTTTCCTTACAATGTGTCCTCTTACTAGGGCTTGAAGCTTCACAAGTCCCTTCAATG
CGCGTAAAGCTCTCCTTGACTTCAAATTTAAACACAAAATGGAAAATTAGTACGAGTGTATGATCAAGTGTCA
TTCGATTTTTTGCAACCAAATAATGCAGAGACGAATACAAGAACTAGAGTTAGTGAATTTTAAAAAATAAATA
AATTGTGATGTTGAAGTTACTCATAGTGTGAATGTATAGACAAATTTCATAGAATTTTAAAGTACACTTAGGAC
CACAATTTTTTCTAGTTTTAGACAATCTGGTCCACACTGGTCCAAAATAGTCCACTTTCCTTTTTCCTATATACA
TTTGACCATGCATGTGCTAACCACCCACCCACCCCCCTGCCGACCTCAACAACCCATGCATTCCAAAAAAAAA
TGTTTAAAAAAAGTACATATAAATTTGCATCGATTGTCAATCAATTTATGCGCAACTCAACTATTTTATCGGACC

TGCTTCCACTAAACATCACAAGTAACTCAACTAATCAAAACTTAAGCAGAGGAAAAGAAAACCATCTAGCTA
ATTTGTTTGTTTTAGTTTTTTTCCTTTTTTCTAAATTTGAACCCTAATTTCACATATATCATATGATCCTAGTATAA
TATAGAGTATATCTCATTCTTTATTCAATGTAACTTTGTATAAAAAAAAACAGGCCAGGGTGGCTCAATTTCTCA
ACTAGAGTATATATTGGGAATCGTTTCTTTTCCCGTCCACCCCTACCCCCTCCCCCCAAAAAAAAATTCCAACC
CTTATTAGTTCTGATTATCTCCCCCTTCTTGGTTTTCATTATATTTCATTGTGTTAAATCTTCTAATTGTAAAAACA
TTATTGTTTTGTTTCAATTTATTTGATACTATCTTTTTGTTAGTTCATTAAAAAAAGGTTGAAACATTTGTATATT
ACCTTAGTGTGAAACTTTTATAGCTATACACAAATATTATATGTAATATATTTAAGTTTACAAGTTTAGAAAAAA
ATTATAATTAACCACGCGCACATAAATTTTGCAACATATTTAGGATCATAATTCTTTAAAAATATATATTTCTTAA
ATTTTCATACTAAGTGAAATTGGTTAATTTTATAGATAAAAAAAACATGTTTGTCTCTCACTATCTAACATTCTCT
AAAATTAGAAACTATTTTGATGAACTATAGAAGAAAAAACCCAAGGAAAACAAAATTCATGTTTTCCCTTCTA
ATACATTTTTTAATGTTTGATTGCCATAAAACAACTTATTTTTCCGGAAAACTCATACTAAACACAATTCAAAAT
TCTCAAATTATTTTTTTCGTGCGAGTAAATACTTTCACATAAATATAAATTAAAACAGAAAGAGGGTACGTATA
ATATAACTAACCAAATAAGCTCGAAAATGTGATTGTATAACAACGGCAGCCCACTCCTCACCACTCCACGGCG
TCACGGACGTTGTTGTACGGATCACACTACTAGTCAAGCTGGAGTGGACCTCACAGATTGTGGGGTCCACTC
CAGCACGACTATTCACGTAGTGTGATTTGTCATGGTGTTGGTGGTGGTGTTGAAAGTCTTTATCTTTGTAGGA
TTTAACATCACTCCACTTCTTCTTATTAGTGGAAGAAGTAGAGGAAGAAGAAGAGTCATTTTTCTTGAAGCC
AAGAAGTGCTTTGAACCATTTGGATGCTTTGCCCATAGTATTGTTATAATAAATTGTGAGATTAGAAAAAAGA
GCATTTGTTTTTGTCAAGAGAAGAGAGAAGGTGGTTGAAAAGAAAAGTGAGTGAGAA
>PGSC0003DMG400017629
CAGAGACGGTACCAAATTTCCAGGTGTTTTTAAAAAAACAAACTTTACCCTGCTTGTTGTTGAGTGCCGTTT
GTTTCTCTCTACTCTCTTCTTCTTGAAGATGCAGATGAACAAAAAATGGTGAATTCTCTCTCTTCATAACTTCAT
ACTTATCTATTAACTCTTTTTTCAGATCTTTCATTAAATAAAGTTTGATTCTTTAGCTGTTTTTTTTTGGATTTAT
GTACTTTTTCTCTTGACCCTTTTCATTTTTGGACTTCTTTTTGCTAAATTCACTTTTATTTCTTGCAGTTAAACA
AAGGTTTTTGTTAAAGTTACAATCTTTGCTCTGTTTGAGGTAAGTTGTAGATCCAATGTACTTCAGGTTTTGAT
TTATAATGTTGGATTCTTGAGCTTACTCTGAAGCTAATGGAGAATACTGATTGCTTTTTTTTTTTTTTTGTTTGT
AAAATAGTTAATGTTAGATGTTACTGGTAATGTTTGTTAATTATATGTGCAAGGTTTAATCTGTGTATACTCAGC
TGTTTCAACATTGTTGCATTTGGGGCTCTGATTTTTATTGGAAATTGGATGTCCTGAAAGACTATGTAGCTTG
GACTCTACAAAAATGTTGCTTACCCGTGTCGGATTCTCCAAAAATAGTGTATTTTTGGAGTATCTGACATGCA
GCTTTTGATGTTTTTGAAGAGTCTGAGCAACATAGCTGAAAGATGTAGCTGGAGTGCTTTGGTGGTAGGGT
TTTCTGCTGAAGGGGATTGCTACACTTGCTAAATTTTTTTAGGAATTAAATAAAAGTATTGCGTTGTTGGTTTC
TTGTGTATCCATTTCTATTGGGTTTTTCATCCAATGTGTTATAGCCTTATAGGTGATACCAGGGTTAGAGTAAGT
AAAGCTTTCCCTTCTCCATTCCTGAACCACAAAGGAGGAAAGGAACACAACTAGAGGCGAATCCAGAATTT
AAAATCTATGGGCTCAATCTTTAAGATTTTTAGTATCCATCATTGTTTAAAATTATGGGTTCAGATCTACTATTTA
TTACTATTTTAGTAAACTTTTGCATATAAATTTACACTCTGTGTCAAAGTAATGGGTTCAGATGAACCCAGTGT
CTGCATGCTTGCTGCATCCGCCCCTGACACAACGTTAGAGGAAGGAAGTTTCTCTATTACACTCATCAAGTCT
GCGAAGCATCTAATTCTCCTCTTCTGGAAGAGATTGTTTGTAGAGTCCCATTGGATAGGTGTTGAATTCATGA
ATCTTACATGAGAGAAGATCACTTGAGATGGTTTGGTCATGTCCGTGTCGATCTCTAGATGCACCAGTTTGTA
TGTGTGCCTTAGTTAGTGAAGATGTTACATGTGACGAGGTAAACTCAAATCACATGGAAAAAAGTTGTCTGG
AAAACCTGAATCTCTTGGAATCCATGCATGCTTAGAGAAAGATAGGGCACAGTGGATGAAAAGATGTCTATG
TATGTTTTAGTCATGTTACTATGCTTTCTAAGAGTCTTTTAGATAAAATATATAGAGAACTGATACTACTTTTTAT
TTATTTATTTTATTTTGTATATTGTTGGTTTGAGATGAATTTAATTGGAGAAACATAGTAAGTGAGAGTTTGTTG
GGTACTGAGGCGTAGTTGTTGCTTCTCTTCTTTTATTTGTTGGCATGTTGATAAACCAATTAGGCTTTAGGCTC
TAGATCCTTGAAAAGAAAAGGATATATTGACAATCAGTCATCTTTATTTTACCACATTCATTTTGACCATACAAT
CTCTTTTAATCCACTAAATTGATCACTGATGGTAGGCTTTTTGTCGGATCTCTAGACTTTTGTTATGCAACTTCA

CTTTTATATATGCAGAATCACAATTGTGCTTTAGTTTTGGAAAAAAGAACACATAGATAAACATTTTAACCTCA
TCAATGGCTTATATTACATCAATGGCTACTTACTGCAGCAAGTTTGCATGTGCTTATTTGATATAGAGCTCTTTT
GAACCTTCCGAACTTATTATTCACAAGGTTCTTACGTTGTGGGCATCAAACTTATTACTATGGGAAAATCTCCT
GGAAAGTGGCTCAGGTCATTGCTGCCAGGGAAAAAGTCTTCCAAATCTGGCACATCAAAGGTAGGAGATAC
GATCCAATTTTGTATTGAATTGAATTATCCCTCTGGTTCTGCCTTTTGCTTATTATCTGCCTGCTTTATAGTAAAG
TGGATTTGTCCATATTTCACTTTGGTTTGTCAAGAGTTAAAAGCTAGTTTTGTACTTTTGTTAGTGCGAAATTG
CTACTACTTTATTGTTTAGTGGATTCACAAGTTAGTAGCTAACTTTTTGCTCCTTCTGGAATAGAATTGGGTAA
GGTTTATACTGAGAAATGTAAAATTGTTGCCATTAGAATTTTCCTCATCTCATCAAGAACAATAAAAATAAAAA
TTGTTGCCTGTAGAACCTTTGCATGGCGAAGGCCGTAACCCATGGTAGTACTTTTTGGGGAAGTGGACATTC
TTTTGAGGCAAGCCATAAGGCGAGCTGTCTAATTACTTTGGTGAATAACATTCATGAAAATGAACCTTGTCTT
CTGCCCTAATGTCAATACCTATGGTCGCTGGCCACGAAAACAGTATTATCATCACAATGCAACAAACTTGAAC
AGGGCAACTTGTAAAGGATATGTTTCTTTGAGAACAATTTTCCACAAGCATTACTTGTTCATTCTGCTTATCCT
TCTTTCCTGTGTGGGGTTTATTTTATTCATATTATTTCTAATAAGTTATTTTAATCTCTGTTTCTTTCTTTAAAGAA
ATCTTCAAATGAGAAAGCATCTGTAATTTCCACCAATGCGGCATTGTCCGGTTCGTCTGTCCATCTGCCATTGA
TATCTGAACCAGTTGCTGGTAATTCTGGTGGAATAAAAGAAGACTCGAACTTTGAGAAAGGAGAGGTCACT
GATGAGGTGATTCTCCCTTCTATTGAGCGAGATGGAGACGAACAAAATACTTGTCTTACCTTACCCGAGGATA
CTGAGAAAATGAGGCTTGAGCAAGCTGCTATGAAGGCACAAGCTATTGTTAGGGGTTATCTGGTAATTACTT
TACCATATAGATTTACTGGTGTCTTAAAATATTTTTGGTTTTTTTTGTGTTCTTTATTACAATTTGAGGAATTATG
TTTGTTTTCGGCAAGCTTTGTCTTCTACCTTCCGATGTCTTCCCCTTCCACTTCAATATTGTGGCAGTGAGTTA
CTTTCACAGCTTTTAACTCTACTATGCTCGTTTCTTGTGGTTAATGAATGTACGAGCTGATTTTATTTGTATTGT
TCTTTCAGCAACATATATGAATAGGTCTTGATTTTCTGTTAAAATGGTTAGAGACAACTGACAAGTGTTAGAT
GTTAAGAGACTGGAAGTTGTAGCATCTGCTAGTTTTTCTCATAGTTTAACCTAAACAAGACATGCAATTGTCC
ATGTTTGTTTAGTAGGGTTTGTCTATATAAACTATTCTTTTGCACAAAGGTGCAGAATGACGGAAGAATTCTG
AAAATTTTACAAGACTATATTGTAAAGATGTAAACAACTTCCTAAGAAGGGAAGTTATGCTACATTAGAATTG
CTAGAAAATGATCTCTTCTGAGACATAAATCTTGGAAAATGACTATCATCAGCAAGCTCTTATACTACCAACAA
AATGGGTTTACCAGTGGCTCCAAAGTCACTTTGAATCTACGTGTTGATAACATTTGGATTCTTGCAACCCTTG
AAGGTCTTAATGGTTTTCTTCATAATGTTCACCAAATGGCTTTGAATAGTGCTTTCCCGATGGATGCATTTAAG
ACTTTATGTGACTGATAGTTTAACCTTTTCTCTGTTCATGTGTTTCCTTAATTTTGCAAATGATTTGAGTGTTAT
GAAAGGACTTGACTACTTACCTCTGACATGATCTTGCTACCTTTGATGTTTAAGAAAATTTTCTATGACAATGG
GCTGATATCTAATTTAACTTGAACATTTAACCTGTACTAATGCCGATATGGTCTCTCTGAAGGCTCGCCGAGCA
TTTCTCAGGCTCAAGGGGACCATAAGGCTACAAGCTGCAGTACGTGGCCATCTGGTCAGAAGGCAGGCTGT
TGCTACATTATACTGTATACATGGCATTGTCAAACTTCAAGCACATATCCGTGGCCAGATTATTAGACGTTCCA
GTATTGGTTGTGAATTGATAACCAAACAAGGACTTGAAAAACAGGTAAACTACCAAATTGACTTTTTAAAAT
TTACTACTCCTTAAGCCCCAATTTATGTGAAGGTGTTTGAAAGAAAGACTCTTGAAATTTGTGTCTAAACGAG
CCTGAGAAATTTCTGTAGCTATAAATCATTTCATCTAGGCTAAAATGTAAATTTTAAAATTATTTTGTCACTTAA
TATAAAAATGTCATTCTTTTCGGGACTCACTGAAAAGGAAGTGCGAGGGGGTACTATCTAAGGTGAAGGAT
GAGGGGCTCACACCTTGTGAGCTACCCTCCAACACAGCCAAATTGACTTAATGGTATCAAGGCTTATTGTAAT
TTGAAATTACAACGTTTAGGAATCCAAGTTTCTTAGACTATATGCTGCCAAGGGTGTTCAATGTACCATTTTTT
ATAACTGTTCTGCTATGAGTCTTGACTTGCTCAATGGCTTATAGTTTTTTATGGTACCTTTCATCATTAGGATGC
TAAACAATTGGATTATCAGAGAACTAATGCATCCAAACTAGCACGGGAGCTATCCAAGAATGAATTCACTACA
AAGGTACACATCTAACTTCTCGCCATTTTTTCTTACTTTTTTTTCCGTACTGTTCCTGCATTGTCTGGACCTAGA
GGGAAGAATATGCCCATAAGACAAGTGGTTATCCCTAAATAACCAAAAGCTGATACCCAGATATTGAAGTTTT
GCATACATGAAAACTCCCCTTACTGCTAAAATTAGTATTTTTTAGATTTTGAGTCATTAAGTGCAAAAGAATGT
CTCATTTGAATTTGAAAGAACCAACTTTTCTCCTTTTTACTTGGTTATTGTTACTATAGTTGACTGATCCACTGA

TTGAACAATCTTTTCATATGGTATATTTCAGCTACTTGCTTCATCACCTACTGTAATGCCTCTACACCTCCATTAT
GGTCCAGAGGAACCAAATTCTAGTCAGGAATGGCTAGTCCGTTGGACTATATCACAGATTTGGCAACCACAA
CCTAAATCGGAAACACTTTCAAGAAAAAAGCATCAAAATGTTGAAGCAGACATTGCTATGTCAAAGCACAGT
GGGAGGAAACTACATTCTAGAAAAATGCAGAACGGTTCAAATCATTCCACTTCTTCAGGGTCAGAAAAGAA
GAAATCAAGTCATCTGGTAAACTCTGTTCTTCAGAATCCTGGAAGTGAGATTAAGAAGGTGAAACATAGTGT
AAAGAAAATGTCCAGCCCTATATTGGAAAAACCAATTCAGTCTGAGGTTGATACTGAGCGAAAAAGGCAAA
GTCATGACAAATTATCAAGCATGACATCTGATGAACCATTGCAAAATTCAGAAGGGATAGTTGAAAATTCTAC
TAATGTGGCCCCATCTCAAGAAACGCTAGGGGTGGATGATACTATCTCTCGTTTGGATATCCTTTCTGTATCTG
ATACTCTTCACAAATCAACTACTGATGCTGCGTATCAAAAACCAATCACTGATAACCAAGAGGATGATACTCCT
GTTGCAAATGAGGATTCTTGTACTAACCATGATAATAATGAGGGGAATGAGAGCAACAAGGTTAACAGAAG
AGTTTCTTTACCTGCAAAGCATGATGTCGATGCAAGTACGCCAACTACGCCAACGACAAGAAAGGTGCCCA
GCTACATGGCTCCAACCAAGTCTGCTAAAGCTAAGCTGAAAGAGCAAGCCTCACCAAGGTTTGGGCAAGAT
GTGGCTGAGAAGAATGCCGTAACCAGACGTCATTCTTTGCCGTCCCCTATGAATGGAAAGCTGAGTTCATCA
CCATCACCACGGGTACAGAGGCTGGTCCAAGCTAGTGCCAAAGAAGGAATCAAGATCGATAGATCTTTATCA
TCTTCAAGGGATGGTACTGGTGAGTATTTAAGTGCCAACTCATTCTCTTCGTCCATTTCAATATGTTTTGTCTTA
GTTTGATTAGACACAGAGTTCTGAAACAGAGAGTAGTTCACTGTGGAGTATGAACTACTCCACAGTGAACTA
CTTGTGCATTAATTTCAAACGATAGAAAAATTGCCTAACCAAAAATATTTGGTCCTAGGTTCTATCAAGAGATT
AATGTATGTCTTGTGAACCCATATACAATGCATCTACTTGACGTTACGCATGTAACTTTTGCCTTTATCATTTCA
GATAAGATGACTCGAGCAGAATGGAAGCGGTAACTCTGCAAGAATCTCAGATGTAGCAGGAAAGTAGTACC
CGGGGATCGATCACTGACAGAAGTCCAGAACAAAACTTCATTATGTTCATATTCCTACCTTTGATGTTAGAAA
GGAACTAAATTATAAGATATAGATGGTGTGCCTTTTGTTGTCTTTTCATTTTGCTTGGGAAATATTGCAAGAGT
TTGTACCTGCTAAACTTTAGATATAGGGTTGTCTTGTTAAGTTCTAGGCATGTTGTCTGTTGAGTTGATATAGA
ATGTGTGTTTTGAGGCAAAATCTATTTGTATGTTCAGTTAATGTTTGCTTTGAAATATTTGATTATGCAAAAATA
CTAATACAAACGAGTTATAGTGTTCTTACTTACCC
>PGSC0003DMG400001315
CTCTGCTACTCAGTAGGTTATGCATAGGGGATATACGCATAAGAATAAAATCTCACTGAGACCAAAAGAGCCG
AATGTTCTTCACTCAAGATACAAGGCATATGGGGCTAATAGAAAAAGGAAAGGAAGATAAAGAATATCAAG
GAAATCCTGTTAAACCAAGTACAGAGTCTCAACTATACAATAACTGCAAACACCCCAACAACACTAATAGAAA
CATGATAAAGATGCTTACGATATCAAACAACGGTTATTTGGAATTTGCAAATTTAACCTAATACACAAAATATT
GATGTCAACAACACATGCGGTACTAAAGTTCCTTGAGTAAGCCGAATGTTGAACTCATCTCCTCCAACCAGG
AAGCACCTTTTCTGGAGGGAGCCAAAAGATAATCATCGAGTCAGTGAAGTGTTCCAAATTGATGAAAGTAA
ATCAGCTTATAACACATCAATTCTGAAATGATCTAAGAGAACCATTTACCAAAAGATAAAGGCATAAGACTATC
ACAATGAAGAATAAAGAATAAATGTCAGACCATTTTACATATGGAGTCTTCAACATTTGAAACAGTGCATGCA
ATTTACTTTACTTTACTTTTGAGAATTCGGTTTTCTTCTTGTTGTCTTAACTTTATGAATGGTCAGAAAGAAAG
AATGACGCACCTCATCCAGACCCAAACATTAATAATTACCATACATCATTTTTATTTTTATTTTAAAAGCAAGTT
ATTCTACAAGTCACTTGAGAAAACAAGCAAGCACATACTTTTCTTGGGTTTGATCAAGATCAAATTTGAAAAT
TCCCAACTATATTACTCATTCAGCTTAAATGAAAAAAGAAAAGAAAAAACATTTCAAAGTAACACAAAATAAC
AAAGGCCCAAACAGAGAACTCACATCAACCTACGACAATTTCATTATGTACTCAGCTATTGGAAACAATCAG
ATAGCTAGAAACATGTAGAGCTACCAACTTGTAACTAAAAGGTCCCTAATCATTTTTTCTTACCGTCTTTCGAG
GAGGGCATCGGTCTGGTCTTGTTTCCACCTTTTCCATTTGCTTGCCCTGGTTTTTGAATCCGAGGTGACAATG
AGTTGAATTTGCCATTGGCAGAGGATGGTAGAGAATGACGCCGAACAAACCCATTTTCAGCTCCATCATCAC
TTACCTTGGGAGATCCTTGAGCTTTAAGCTTTGCCTTAGCAGATTGAGTAGCAGCCATATAACTAGGCACACT
TGGTGTATTCTGTGAAATATTCTCAGAATTGTCCTGCTTAGTGGGGAGAGACTTCCTCCTCCTAATTCTTTCTT
TGGTAGTCTGATCTTCCATCGAGCTAAGTTCCTCATTGACAACTGGGCTGTTTGCGGTGTTGTTAACATCCTC

CGACTGCTGCTGCTCAGTGGTAGGATGATCGTGCAGTACATCACTTGGCTCATCCACTGGTACAGGTAAAGG
AGCTTCACTTTCAGCCAATTTCTCGATTTCAGGAACTGAATCACTCGTTTTCTCATAAGAATTAACCATCATTT
GTTCAACAACATCTGGAGCTGAGGATTTTGATGCTATGGCCTGTGCCTGAGCCTGAGCCAGATTAGGAGTCT
GTTGCGCCTTCTCAATTTCTGTTTCAGACCTCTCTGAAGATGTTGCCAAAGCTGCAGAAACTTTTCTTAAATT
GCGCTTGACTTTTTCAAGTTCATTTTGAGGCTGATCCTGCACTGGCTCTATGTGATGATTCGAGAATTTCCTT
GGGTTGCGTTTAGCCTTTTCTGGTTCAGAGGAACTAACGGCATAAGCATCTCCATTTGATGCTGTAAGAACC
TTCTTGACACTTCGTTTTGGTCGTACAGCTTCTGTTTCAACACTCGGCTTGTTACCCTGCTTCTTCAGCGACTT
TGCTCCAACTACTTTCTTTGGTTGTGGAAGTGGTTCCCAAAAGCGAGATAGTGACCATCTTTCAAGCCACTG
CCAAGCTGAATTAGGTTCGCACTCATCATACTGAAGGCTCAAAGGCATGGCTGTTGGCACTGCCACAAGAA
GCTAAAACAAGCAAGAGATTACCATCAAATACAGAATTGTGTCTACATCAGAAAACTAAAGAAATACTTCAC
GGTCCCAAATAGTTGGCATTATTACTATGAGTCTTAACTGTTTTACAATGATCATTTCAAAAAAGGTGGTTTAA
ATTGATCATATTTTTAGTACTTTGCGTGCAATTTTGAGAAATGCTAATTTTATTCCAAGGAATTACAAAATGTTA
GAATTCATACTGATCATTAGATGAGTTAACTCTCGTACAGTCGTACTTTACCCTCCCCCCACCCCCCAAATTGTT
TAGACTTCCTGACTCTTCAAATCCACTTTAGGTAGTATATGGATTCATTTTTATCAGCAGTATATGGATATCCATT
GGACCTTTGTGATGCACATACTATGGGTCAGGCTTCGATATTCAAAGCAACATTCTCTCTCTTCATAATAAGTA
ACTTCAGCTCAACTTCCAACTCTTCCTTTGCAAAGCCCCAGATCTAGGATGGTCATTCTATTAAGGCATCAAA
GATGGATTTACGACTTGTTCAACTTTCTCAGAAAATAATGGCAGGCAAAAACCTTCTTTAGATTAGCACAAAT
AGCGTTAGTAAAAGACATGCAAAATGTTGCAGATCACATAAGAATCCAAAGAGGCACCCTTCAGTCTGCAGC
CACACTGCAGTAAATGAAAGCAATGATGCAGACAAATCAGTAACACCTTTGCACTCTGGATATACAGAATGTT
GAACCTGTATCAGAAACCAGATTTTCTAACCTTTTAAGATGTGTTACTCCAGTTTTATTTAATCTTTACTCACCC
CCTCCTTTTATTACATTGGGGACTATTTTCAAGGTTCCTCAACCTTATATAAGCAGCGCGGATAACTTCCCATA
CCCATCACCTTATACTTTGTTCTCTGTTTATTGGCACCTCCCTGACCCATCGTCTACCATAAAAGTATAAAAACC
CAAAAGTAAAAGGATGAAGCAACATCATGATTTTCTAACCCCAATCTCCTAGTTAAATACAATATCAGCCACA
CACAGGTCTTCAACACCATCCTAACCTAGGGCCAAAAGGAGAAAAAGAAAAGAAACAACACCTTATAGGAA
AAACCTGTGATGCAACGATAACTTGTCAGTTAAAAAAAGGACAGATAGTTCAGCAATACCTTGCGTGGAAAT
GCATAAGCTGTCAGTTTTGCAGGCCTCTGCTCAGGATCCTGGTTAAAGAAAGAATACATGGAACTCAGAGTC
AATATCTGTACCAACATGAATAAATTTGAAATAGCTACTACAATCTGTCAAAGTTGAAACAAGGGGGAAAGA
AAAGCAGTAGCAGATTCCTACTCATTCTATGTTACAACAAAGTAAAAAAAAATGAAGAAATGATGAAAAGAA
ATCCAAATTTTAGTTTTAGTACTTTGCACTCTTTTTTGGATCGTCCAATTTTATGTGCAGTTCCTTTTGAATTATT
CAAATGTTATGTGCACTTCCGCTTTTGGTTTGTTCCAATTGTTTGACACACTACTACAAACATGAAAATCAGTT
ACTCCACTCTTAACAATTATTAGTTTGAATTGGCTGCCAATTTACATCTAAAATATGGACATGATGTTTTAAAAT
AAAACTAAATCTAAGTTACTTCTTCTATCACTAATAGCGTTACAGGTAAGTCAAGTTCCCTACAAGACTAATAT
TTAAGCCAAACCAACTCTGTATATGTCCCAGTAAATATCAACTCCATCTTGTTTTAATGCCTATGCTTTTACAAT
AACAAAAGGAATGAGAATACAAGCAAGTCAGGGAAAAAGAGAATTTAGTAAGACCCGTATTGCAAGAGTT
ACCTTAAGTTCCTCAAAATTATACTTTCCGAGCAGCTGATGCCCAGGATCTAAGAGTCTAATCCTTCGGCCAC
GAGCTAATGCCTGAATCCTGACAATCGCCTGCATGCAGCGTAAAGTAGAAACTGCCTGTCTCCTTACCAAGT
GCCCACGAATAAGGGCTTGTAGCCTTATGATGCCTTTGAGAGCTCGAAAAGCCCGACGAGCCTGAGGAAAG
TTATAAGTGGATAAGGACACGGTCCTTTCAGAGAAAAGAACAATTGAAAGGCAGATCCTGCTTTGAAATTA
GAGGAGGAAAAGACATCGAAGCTTCTTCAATTTTAAGTCCAACTAATTTGTTCTACATAAAGGCTAAAGCTAT
GGGCTTTCAAGAAAAAGTATGCTGGCATGGTAAAAGTGTTGATTGATTATTTGAGTTAACAGAAAAGGTTTC
TTATCTAATGCAGAACTAATTTACACAAGCAGATAATTTAGAACCCAAGTGCCCCACAAGCAATGACAATGTA
GAGGATCACATAAATTCTACTGTCTCACTTACACAGAAGAAAAAGTTCTAAACTCTTACAGATTTTATGCATTG
CACGCATAAACATTCAAATATTCTACAATCTAACAGAGAAGCTATTCATTAGAGTGAATAACTGAAAACAGAA
TTAAAATCATAATAATCGTACACGTTTTTTTAGAGATTAAAGTTGTAAATTCTGTAATTAAACAGTACCAAGTA

GCCCCTAAAGGCTGCCTGTGCTATGGTGGCAGCATGCTCTTGTCTTTTCAGCTCAGCATCATCTGTGGAGATT
GTCCCATTTACATGCGGTTCTATATCATGTGTTGCAGAGGATAATGAGGCGGTTTCACATGCAAAGTCAGTGC
TTGTACCCTTTTCCAATCCAGCCTGGTCACCACCATTATCAAAATTCTGAACTGGTAGATCCAAACTTGGTGA
ATCAAGAGAGAGATCCCCCACCGGCGCTTTTGCAGAAGATATTTTCTCACCCGAAGCATCCTTCATACAACA
CATAGAAGTAACTTAACAATATAGGATAAAAAATTAAAATAACATGCATTATGTTGAAATCTCTAAAGATCTTG
GTTTACCAAGTCGTTTCTCTACCCACTCTAATTCTAGATTTTTTCATGCCAGAGTTCCTAAATGTTATCAAACAA
TATAGAACGTTCTTACTACTTTATAAATGGAGAGAGACAGCAAGAGAAAGAGAGGGGAAGGTATGGTCTCA
TTTACCAGTGACAATTAATTAAGAAGCCAAATTGCTGAAAACCAGGTCGAACCAAACGAGTAGTAGAATTTT
CATGACAATGTGAGAAACTTATTATCATGGAGAACACAATCAGACATAGTTAGGAAACCACCATTAACTATTA
AAGGAGGAACAAGAGAGAAATGTCTTTTAGAACCCAGACAATGTTGAATTATGACACAATTAGAAATATCCT
CTTACCTTTGATAAATGAGATTTGGATGATTTCTTCCCAAAAAGCACGGCCTTGATCCACTTGGCAGGAGATT
TTTTCCCCATTGCAGACAGCAAGCTGCTGAATTAAAGAGGATTTCAACTGAATCCTGCATTATTCAATATAAAT
TAAGTTCTCCTTAAAACAAGTAAACATGAGGTAATATGCACTAATTCAACTTCAAAACAATCTCTGAAGGATA
ACCAAACTCTTCCCAAATAGAATCAACAGACACACCAGCTAATCAGCTACAGCTGTAAATGGTGAAGATCTAT
AAACTAAAGTTAGGGAGCTGCATATCATAAGTAGGGACACTAGAGCATTTCTCATACTGAAATGAATGCCTCA
ATACCACCAGTCACTCTGAGTCAGCATTACAACAACAGCGCCTACTACTACTACTACTACACCACCAATCAAAC
TAACAGAAATGAAGAAACATAATGTAAATTTGTAAGCAGTGTCCTCTTCCTGTCAATACACTTAATAGCAAATC
CGAAGAAGAAACACAACAATTTCCAGAAAAAACATATGAAATAGGCAAAACAGAGTCTGGTAACTCGGAAT
CAGAATGCTAAAACGAGTATGACATTAAATCAAGACAAGATCTGTAGTAAAAGCACAAAAAATCCAAAATTA
AGGCAAAACAAAAGGTGTAGTAGGGTTTTCGGAGCAAATGTAGCTCAGATCTGAGAAGTACCTGAAGAAC
AGAAACGAAAGCTCCTGTTTTAAGATCCGAAACTTGGCATAGATGAGCTGAAAGAAGAAATTGAAGAAGA
AGAGAGGTACAAATTGTTCAGTAGAATCAGGTAGACTGAGATGAAAGCTAAGAGGAAATAAAAATTGGAGT
TG
>PGSC0003DMG400012720
CAAGCAAGAACCAGAGGAAATTCATTTGCAAAATTGGAAGTAGAAATGTTTACAGCAGTCAACCATCAAAC
AAAACCAACCATCACTGCCTTTAACAGACTCAAACTCTCTGAAGCATACAAAATTAGGCAAAAACAAACAAG
AAAGAAGAGACTTTGAACTATTTACATGTTTATGTGAAGTCTATAAGAGTATAAAACCAGAAAACAAGAAAC
AAAAACACTTCTTTTCCTCAATCCATTTGTATAAATTACACAAGTGGCTCTATATGCAGATTATAGCTTCTTCTT
GATTGAAGTTTCCGTTTTGCCTTGCTGGAAATATCGAGCAACTGAGCAAGCAATGCATGATAAGCTTTTCAAT
CTCGGCAAAACCCTTTTATATTTATAGAAATGTTTGCTGAGAGATTTCACTCTTTTTGGTACTTTTGTTCACTTC
ACCATTATATGAAGTCCAAGAAGTATGGTTGAGCATGAAGGGAGAATGTTGAACTGAATATGTTTCGTTTAAC
ATTAGTCGTTGCTTTGGTGTGCTCATCGATCTCGTCTTTGCTTTTGCAGATTCTGTAGCAGCCATGTAAGTAGG
AAACATGGGAGAACTTGGTAAAGAGCTATCATCACCAATTGATTTCTGTTTCACATGAGAAAATGATCTCCTT
GGTAGCGATGACGGAGAATTAAAATCCTCTGTGACATCTAGTTTACGCGTTTGTCTCATTTGGTTCATGCCAA
CTAGAGGACTACTTGAGTTTGCAAATGACCTCAATTGCTCAAATAGCTCTGCTTTCCTCTGTGCTTCCATTTCT
TCCCATTGATCGAACCTGGAACTTCTTCGATTTTCCTTAATGATTAACGGATCTTGCATAACATGATCATTTCTC
CGCTCCTAAAAACCATTTTGAGTTATTCAACCATAAAACATGACATATTACTCGTTTGTCTGGTTTTGACTTGA
CACGGAATTTAAGAAAGTAAAGAAGACTTTCAAATTTTGTGGTGTTAAACATGTCATGTGGAAAGTTGAAAT
TTTAATAAAAGGAAAGAGATGTTCTTTTTGAAACGGACTAAAAAGGAAAGTAAGACAAAAAATTTGAAACG
GAGGGAGTAGTAAGTATCATTAAAAGAGAACTATGAAATTCTCAATGCAAGTGAAGTTACCCGATGTGAAAA
CGAGTATTTCATCATACGCTCTCTTTTGGCAAAGGCTTCTCGTCTTCTTAAAAACAAGGAGTCAATTTCTTCCT
TTGAAGCCAAACTGAAATCCCAAGTCCTTTGGCTGTTGCATTTTAGCTGCCGATAAGCAAAACAATGTTCGA
CAGACTTCATCATTAGAGTAAAACAAATACAAATTTAAGTTCAATCAGAGGCTCAAACAATGATAAATATTGT
GTGTTATTAATTCTCTTACTTTGAATTCATTAGATTTCACACTTTCCTTCGAGCTATCATCAAGTTTCTTTTCGAT

ACTCTCGTAGTATTCTTCCACAGTAGGAACTCTGATATGATAAACTCTTGGCTTTGGCATTTGAAAGGGCAAC
ATAAACTTCAATTTGGCAACTAATCTTCGTCTAACAAGTTCTCCTCGAATCACTGCTTGAAGCTTCACGAGTCC
CTTCAGGGCACTTAGTGCTTTCCTTGCCTACAGAAAATCCCAGAGAGACACATTATAATTGGACATAAGGGC
ATTTCGACAACGAAGTGGAAAATTTTGTAATAATGCAAAGGAGAATAACAAGATTGACATGAAAGAGTTGTT
TTTGTTCTCTTTTCGTAACATTGATAAATATTGAACTTCAGTTCTTATAATATTCATCATTAGTGGAAACTAAAG
CAAAGGCTGCAATTTCTTTACTGATAATCCCTTTAAGTTTCATGATGATTTACTAATTAAAATATAACATAAAGC
AATCAAATACACATCATATATCCCTAACAAGCCTAATCGTTTTTTTCTCGGAGAAGCACGTGGCAATTCTTTTT
TAAGGTGATACCATGTTGAATGTTTCATCTAAAATCTTAAGTTGTATAAGAGAGAATACTTTTATTTACTCACAA
GGTGTGCACGATAAGCGGTTTGGATTCTGATGGCAGCATTTCTCCGCTTCCTCTCTAGCTCATACGGAGCGAT
AGTGAGGCGTACAACTTCGGCTGCAGCATTGGCAGCAGCTACAGCAGCCTCAGCTGCAGCTGCTGTTGCTA
AAGCAACAGCCACAGCAAGTTTCTTCTGTTGTTCTGTTGCCTCAGTTAGTGTTTTTTGAGGTGCTTCAATTGC
TGGAGGACACTGCTTGAACTTGAACCTTCCTAAAAACCATTTCCATCTCTTTGGTTTCTGAAAATTAAAAAAA
TTCAACATTCATGTTCTCAACAGATAAGGGAAGCCTGATGCACGAAGTATCCTGGAGTTCACGCATAGTCCA
GATAAGGGGCGCACCCCACGAGGGTGTAATGTAAGTAGCCAGTAGCCTATGCTGACGTGCAAGCATCAACG
GCTGATTCCACGGCTCGAACTCGTGACCTATATTTCCACATTCATGTTATAAATCACACGAAGGGGAGCCTTG
GAGCAACGGTAAAGTTGTCTCCATGTGACCTACAGGTTACAGGTTCAAATCATATATAGAATTAGCCACTATT
GTTTGCGTCAGGGTAGACTGTCTAACTCACACTACCTTATGGTGCATCCCTTCCCTGGATCCTGCGTGAACAC
GGGATGCTTCGTACTGCCGTTTATTTCATATTAATTATAGAATCATATCAACCAATTCTCAATAGAAGAAAGGG
AATCAAGATTTCAAGAAAAATAATCAAACCTTTTCAGCTTTTGGTTTTGCCTCAGGAATGAAAAGTAGTCTTT
TCACAAATATAAACCAGCTTCTTCTCTTTCCCATTTTATGAATTCAGCAAACTGATTCATCAAAACAATAACATG
CCATCACCTTCTTTGTGAAAAATCACCTTATTCTTGATAAGAGAAAAAAATAATCTCCTTGAGTTTGCAAGTTC
TATTTTATTTTATCAATAAACAAAGAAGTTCCAAGATAATTTACATTTAGAAAACAGGGAAAAAAAACAAAGA
ATAATGAAACATGACATGCACAACTTTTCACATCACCAAACAGAGAGGGAAAAAAACACAAGAAATTCAATT
TCATTCTCTTGCATAATCATGAGCAACAAGACATTCTTGTGATCAACAAGGTTTGTGGGGTTGTGGTGGAGT
GTAAGGATACCTTTCTCTTTAATAAGAGGTCTCGAAATTCGAGCCTGAATATATATGGAGTCGCATTTGTTAGA
CAACACTTTACTTCTAAGGTTGGAATTTAGTTGAGCTACAATGTCCATGACAAAATTCAGGAATTGAAGTTTA
TAAGTACGAGATTCTAGTACTTCGGAATTAATAATTGTACATAGTAAATGAATTTAGAAAGACAAATATATGATT
TGAACTATAATGTGGATACCAGACACAAGGCATAGGAAGCAAAAAAAAAAAAGACATTATTATTGTAATATAT
ATCAAACCATTCATGAGACTAATCAAGAACTTGAAATCTTAATGGAGCAATTTTCATAATTTTTCCAATTAAAA
CAAGGTAATTATGAGAAACAAAATTAGGATACATACCTAGAGTAGGTGAAAGAAAGATGATTAAATTAAAAC
ACCAAATTTGAAGGACATGTTGAAGATTTGGAAAAATCCAAGTGTGTCTTATGAAGTGATTCTTGAATTATGA
TGTTTTTG
>PGSC0003DMG400012115
CGTATCTCTTTTATTTCCCACTCTCTCTCTCTCTCCTTTATTTATTGTTTCAGGAGCTGAAGGCTTTTGCCAAT
TTAATAAACCACACCACACCACACTCTGTTCTCACCTTCTTCTTTACTTGTCGCTTTAACCAAAGCAATTTCTAC
CTCTCAATGGGAAAGAAAAAGGGAGGTACTTCATGGTTGAGTGCTGTTAAGAGGGCTTTCAGGTCACCAAC
TAAAGATAACAGCTGCGACAAAAAGGCAAAAATAGAGCATGAACTAGACGAAGATGAAGAAAAGGTAATG
TATATATACTTGCTTAATCCTATATATATATATTTTTCCTGAACTAGAATCACATTGTTTTGTATATACAAAATCAAT
TTTTTTTCACTACTATAGAGAACCATTATGATCTTGAGTAATGAATATTTTCTACCCAACTATATAGAATCTTTAA
TTGGCGTTACTTGTACAGAAAAGAGAGAAGAGAAGGTGGTTATTCAGAAAACAGTCACAAAATGAAGGGA
AAGTAATTGTTGATCCAAAGCATGCAACAGCAGCAGCAGCTGTGGCCACTGCTCAAGCAGCAGTTGAGATC
ATAAGGTTGACACGATCTTCCAATAATCCGTCTTCTAACAGGCAACACAATGCTGCTGTACTCATCCAAACAG
CATTCAGAGGCTATCTGGTAACCTAATCATTCCAATCTTATGCCAATTACTTATCAATTCTTCTTTGAGCTTTTG
ACCATTTAATTAGTGTTACTGACAAGATTGTTAAGATACAAGTTACGTAGAAAAATTACTATTTTCACTAGATT

TTGACTTGTGCTAAAGAAAATAAAACCAGAGAAAGCTGTTTTCGGCTATATATACTGCTCTTAAAATGCTGCT
GCACCTGTGTCGGTTTGCACTACTTTTTGGATGATCCATTTTTGAAGAGTCTGAGCAATACGAGTTTTGTGAT
ATTTAACTTCAGTTTCATTTCTATAAAATGGACAATGTTTTGGGACAAGTAAAAAAACTAAGTAGTAATAGCA
ATTTAGAAAGAAGAAAGTAGTGAGTTAGCACTTGCCACAACTAGTTGTTAATTCAGAGCAGACACTAGATAG
CTTTAGATTTGGGACCATTTTCAATTGATCACAAGTCTTGCATTGGAGTGTTAAATATGTACAGATAGCTGTCA
TGTGAAGCAGTTGATTTATTGTTATTGCAGGCAAGGAGGGCACTAATTGCATTGAAGGGGATAGTGAAGCT
TCAAGCATTAATAAGGGGTCAAAATGTACGAAAGCAAGCTAAGATGACACTGAAATGTATGCAAGCTCTGCT
GAGGGTGCAGGCTCGGGTTCGTGAACAACGCGCTCGCCTTTCACATGATGGAGGCCGCAGGTCCATGTTT
GCTGAAACAACCAATTTATGGGACTCTAAATATCTCCGTGACATCCGAGATAGGAAGTCCAGAGTGAGTTCT
ACTTTCCGCGCCTTAGTTACTGTGTGATAAACAGCACTCAGTAGCATCTCTGTCTCTAACACTCGTAATTTTTT
GTACTCCGTGCAGTCTAGAGATGGAAGCTCAATTGCAGACGATTGTCCGAGATCACTTCTAGAGCTGGAATC
AATGTTACAAGCCAGAAAAGAAGCCTCCTTCAAACGGGAAAAATCCCTTGCTCATGCTTTTACTCAACAGGT
ACAACTTTAATTTCTGTATCCCTTTAATTAATTCTATTTACATCCTTTCATGAACTGAAATACTTACTGTCTTTTAC
ACATTCCAAAATCAAGGAATTGGATGAGATGGATGTTTGTAGTGAAGAAAGAAATGAAAGGGAACTAGAA
GAGACAGCGAATTGGCTAGACGAGTGGATGTCATCAAAGCAATGGAACACCAGCAACAGAGGTTCATTTG
ACAGAAGAGACTCTATAAAGACTGTTGAGATGGACACGGCTAAGCCATATTCTAACATGGTTCCAAATGCTC
GAAGATCACAACACTCGAGCCCACTTCACAGACAGGCTAGTAGTCCTCATTATATTGCTAATTCTCCCCATCAC
CAGAGATCATCACATTACAATTACTCGGCAATTCAACCACCAGCCACCCCGCCCCCTTGTCAACCAAAACCTC
TTCAAATGCGCCCAACAAGCCCACGTAAAAGCCAATCAACTGCAAACACTCCATGCCTACGCTCTACGAGCC
GTTCAAACAGTATAATGTCCCGGTATAGCACGTCAGGAAACGATGCATCAGTTCCTAACTATATGGCTGCCAC
TGAGTCTGCAAAAGCTCGGATTCGTTCACAAAGCACACCTAAACAAAGGCCTTCCACACCAGAAAGAGAAA
GAGTTGGATCGGTGAAAAAACGCCTCTCTTACCCTATTCCTGAGCCATACTCGCTAAACGCTGCGTATGGCTA
CAGTCAGAACTTGAGAAGTCCTAGCTTCAAAAGTCTTCAAGCTGCCTATGTTGGGATGGAACAACAATCATG
TTACACCGACAGCCTTGGTGGAGAAATTTCTCCTTGTTCAACCACAGATTTAAGGAGATGGTTAAGATGAGT
AGTAATCAATTGAATAAAGATTACAGGAACCAAGCCATCTTTTGTGTCCTATTTTTCTTTAAGTTTAGCCAGGT
GCTAGGTGAATGCTAGTCATAAATGATGTGTATTTCTTTGTTCATTTTCTCTAATGGTTCATTACCCTTCAGCTC
ATTTATTATGCACCTAGGAATCACATTTTCA
>PGSC0003DMG400014723
TATACATCAAAGTATTTTATGTTTTTAATAAGCTTTCATATTGTAACAAAATTATGAAGAAGAAACATATCCCCT
CATATAGCAAGCAAACACAAGGCCAAGGGACAACAACAACAACAAATGATCATCAACTAAGTTTAGTAACAC
AAGATGAAAGTTTGCTAACAAAATGTTTCATAGGGAAGAGTATAATTGGTGGGCCTTCTTGGTTAGGGTTAA
TGAAGAAGGCTTTTAGATCACCTATCAAAGAGAATGATATCAAAAGTATTAGAAGAAGGGAAGAAGCTCAT
GACCAAGAAGAAGAAGAAAAGGTATACTACTTAATTATAAACATGTTAATTAATTTACACTAGCAATAATCTTT
TTTTTGCAACTAATTCATCGCTAAATAGAATTAGCAGACAAGAGGGGTTGTTCAGATGTAAAGTATTATGTATA
TCCTCCACCTCCAACTCTAAAATTCTAGGTTCAAGTCATCAGGAGAGCAAAAAATTAAATGTGAAATCACAAT
TTTTTTTTCCATTTAGCTACAAAAATTGTCCATTGCTAATTCTTGATAGTAACATTACGAGACATAGAGGGAAG
ATCTAGAGTTATTTTGCTGATTAGTTCACTACTAGAAATATAGAAATTAACGACGGATAAATTCTATCCTTGAAT
AACAATCTAACCATAGACTAGTAACGAAGTTCATAGCTAATTTCAATTTTTTTTTATTTGTAGAAGAGGGGGA
AACGACGATGGATCTTCCGAAAACCTACTGTTCACGAAACAACATTAATTCATCACAATCAAGATCAAGAAA
ATGCAGCTACAACAAGCAAAGGAAATTTGGCTACAATTTTTTTGCCAAAAAATGCTGATTTGAAGCAAAAA
GGAGCAATTGGAGTAGCTATAACTAGGGGAGGATCTAGCTTATCAGGTTGTTCATATTCTGTATTTGTATTTTA
TTTGTCTCAATTTACGTGATTCTTTTCATTTTTCGAGATCCAAATTATTTATAAACTTTTACCAACACAGTTTTTT
ATTCTATAGATATGAGAAAAAAATGTACTTTCTTCGTACCATATTGACTAATTAGAGATGTATTTACTAATCTGA
CTTCATTAATGATTTTCTTGAACTCTAAATTTAATTAGTATACTATTTTACGTAGTTATTTAATACTATGGATAGTA

TGAGAAAAAAAGAACTCTCTTGATTTGATAAAATGAAATATTTATTTTTGATATGGGCCAAGTAAAATGAAAC
AAATCAGGAGTAACTAATAGTACTTTTATATTTATAGCTTTTGAATATATAAATTTTGATTTTGAAATATTAAATT
GATCTAATTCAACTTAGCTTTCATGACTAGTCAAACTAAGCTACTTTTGACCATAGAGTTTTGGAGTAAATTCT
GAAGTCGAATTAAAAAGGCATAATAAATAAATGTGTAGATTAAATATAAACAATTGTAGATCTTATAAAATAAT
CACATTCTTTAATTCAATTATCATTTTTCTTTCTATATTTTTCTATTTATCTACTCATAAATTTCATATTCTAAATTTC
TCAGCCGCTACAACTTCTACAATAGCCACTGATGTCGCAATTGCTAAAACACAAGCAACTATAGATATCACTCA
GCTCACACAACCTCCTATTTTAGTTAAACAACAATGTGCCATCCTTGTCATTCAGACAACATTCAGAGGGTAC
TTGGTATGTATAGATATACCTTTATAATTGTCTCTATATTTTATATCGATGGTGAAAAAAAATATTTTTCGCAATAT
AATGATGTCGATTATAATATTTTGTTCTGATCGCATGTTAAATTATGTTAATAATGTAACATATATAATCGGTATAC
AACTTAATTCATGTATGATTTTTAATAAGTATAAGTTATATTCCATGATTGTTAGTGTATAGTTTTAATATTATTGA
TGCATTTTAATCTATAGTACTTATATTAATTACTATTTTTATATTACTAATTAATTAGCCCGCGATAAAATAATTTAG
TAAGTTTAGTAAGAAAATAAAGGATGGGGTTGGGNNNNNNNNNNNNNNNNNNNNNNNNNN
NNNNNNNNNNNNNNNNNNNNNNNNNNNNNNNNNNNNNNNNNNNNNNNNNNNNNNNNNNNN
NNNNNNNNNNNNNNNNNNNNNNNNNNNNNNNNNNNNNNNNNNNNNNNNNNNNNNNNNNNN
NNNNNNNNNNNNNNNNNNNNNNNNNNNNNNNNNNNNNNNNNNNNNNNNNNNNNNNNNNNN
NNNNNNNNNNNNNNNNNNNNNNNNNNNNNNNNNNNNNNNNNNNNNNNNNNNNNNNNNNNN
NNNNNNNNNNNNNNNNNNNNNNNNNNNNNNNNNNNNNNNNNNNNNNNNNNNNNNNNNNNN
NNNNNNNNNNNNNNNNNNNNNNNNNNNNNNNNNNNNNNNNNNNNNNNNNNNNNNNNNNNN
NNNNNNNNNNNNNNNNNNNNNNNNNNNNNNNNNNNNNNNNNNNNNNNNNNNNNNNNNNNN
NNNNNNNNNNNNNNNNNNNNNNNNNNNNNNNNNNNNNNNNNNNNNNNNNNNNNNNNNNNN
NNNNNNNNNNNNNNNNNNNNNNNNNNNNNNNNNNNNNNNNNNNNNNNNNNNNNNNNNNNN
NNNNNNNNNNNNNNNNNNNNNNNNNNNNNNNNNNNNNNNNNNNNNNNNNNNNNNNNNNNN
NNNNNNNNNNNNNNNNNNNNNNNNNNNNNNNNNNNNNNNNNNNNNGTCGGCAGTGTGTAC
ATTAGTACCAAATATATATATATATATATATAAGACATACCTAAAAACAAAAACAAAAAAGCAAAAAAGGAAAA
ATTATTTATAAATAAGGAAAATTGTTTATATTATAGTATAAATAACTCCTTAATATTATTGGTGTATCTAACTTACA
TTCAGTAATAATAGGTGACATAGTTAGACAGAAATGGAAGGGTAAAAGTGAGTCTATCATATTGGGGGGAAA
ATTACTCATTTCTTTTTAGATAATATATAACGATCTTAGCTTATTATTAAAAATGTCATATCACCTCTTTTATTATAT
GAATCATATAGATATAAATTATGTAAATATTTTTAAGGCGTCATTTCATGATTTAAAAACGGTCAATTATAAAAG
AAAAGAAGAGGAAACAATAAAAAAAATTTCATTTCTTCTTTATTTTCTCATTTTCAATGATTTTTTGTGTGGACA
TATTAGGCAAGAAAAGCACTTGGAGCGCTCAAGGGTGTCGTGAAATTGCAAGCTTTAATAAGAGGCCATAA
TGTTCGAAAACGAGCGCAAATTACACTCCAATGCATGCAATCTCTTGTTCGCGTGCAAAATCAGGTAACTTTG
ACTCAAATCTTATATTTGTATGAAAAAAAATATTGAATATGTACAAATAATTTATTCAGAACTCAAAAAAATGATGA
AATTCTAGTTTAGAACTCTTAAACTAAAAATCATGTCTCTACCGCGCTGCTTAGGTGTGTGATCAACGTAGAA
GGTTATCTTGTGAAGGAATTAGTTGTGATTCAATGTTCAAAGAGCCAAAAAGCATTTTGGAGCTTCATCTCA
ATGATAAGGAGTCCAATGTGAGTGTCTTATTTATATTAAACCCTTTGGGAACGTCCGATAAGATTGGAACGAC
ACAGAAAATATACTTATATGTTATTTTTCATATGTTTATGTAGTCTATAAATCAAGCTAGTATTCCGGACAACGAT
GGTTATGATCATTTACATGCACTAGAAAAAATTGAAGCTTTGTTACACAAAGCAAAGGTAGCTGCTAAAAAG
CGCGAAAATACGCTTGCTCATGCATTCTCTCGAAAGGTATATATATTCACTAATCATTTCGACTCTTTTCTCTAC
TTAGTCAACTTCTCATATTTCCTCGAATACTATTTTTTAGAAGTCATTTACCCAAGCTTTCACCTCTTTAGTTAGT
AGAGAACCTCAAGTACGTCTAATAGCTTCAAATTTTTGTATTATAGATGTGGACCTCGAATAAGGATGAAGAT
TCAAGTAGCAATACAGAACTTGACGAGGATTTGAGAGTTTTTGACGTAATAGATGAAAAGAATCGCAAAAG
TACTAGTAGAGCTTCATGCGATCAACCAAGAGATCGCATAAAGAACATTGAAATTGACACGGCTTGTTCTTAC
TCTAACTCGGACACTGAGTTTTGGCGATTACACCATCAATACTATCACGACCATCAACAAAAGTTCTATTCATC
GTATGTAGTCCCTTCTCCTCTCTCCCATAGAGCGAATATCAACGCCTTGCCCATGACACCACCGTTAAAGATGA

AAAATATCCAAGTTCACTCTGCTAGTCCTCGCTACAGAAGGGAAGAGAACAACCATCACCGAATGGCTCTTC
TCTCCCATAGAGCGAATGTTAACTCTTCACCTACAGCAGTTGATCAGCCAAGTTACATGGCAGCTACAGCATC
AGCTAGGGCGCGAGAACGGTCACAAAGCACTCCAAGACAGATACCGATGACTCCAGAAAGAGAAAAAACA
AGTTCAACAAAGAAACGTCTATCTTTCCCTATTCAATGAGAGACATTGTTAACTTATTATATCGGTTAGCACTA
GATAGAAAACGCGTACAACTGTACATGGATCATTAATCCGATAGACATTATTATTACTATGTAAAATGTATTTTC
AATCTCGTGATCTTACACATATCAGATCACAAGATTGAAA
>PGSC0003DMG400019447
ATGGGGAAGGCAAGCAAATGGATCAGAAACTTCCTAATGGTGATGGGGAAAAAGGAGGAAAGGGAAAAG
AAAGAATACAAATCAATAGAAAGTATGGGCACCCCAACAACTCCAAAGGCGAAAAGGAGATGGAGTTTCAA
GAAATCATCAAGCATGGAGAGAAAAAGCCACAAGAGTAACAGGTCTTTCGACTTAACTTTTGATCACCAAC
TAAACACACAAGGTTCAATGCTGGAGTTTGACATGCTAGAGAAACACCACAAAGCAAGCCTAACAGCAAAA
GGAGCTATAAAACCAAAGGCATACCTTACCAGACGTGTTAAGGATGCAGCTGCCACCAAAATCCAAGCTGTT
TTCCGTGCTTATTTGGTAACCATTCAAACCATGAGTGTGTTCTTGTTTATTTTTAAACTAGGTAAAGCATGTTT
GGTTTCATTTGATCCTCATAACTTGCACTAAGTTACCTATTATGGAGAGGACCTTGTTCTCACCATAAGGTCAA
TGAACATGCACCGCTAAACTATGTAGGGCTAAATAAATAAGGTTTAATGCTAAAAGGTGAAAAATGGTTTGA
AAGCATGACTGATGTTTATGCAGGCTAGGAAAGCATTGCGCGCCCTAAGAAGCCTAGTTAGATTACAGGCAC
TGGTAAGGGGTCACCTAGTGAGGAAACAGACAGCAGCAATGGTGAGACGCATGCACTCTCTTATGGTCATT
CAACTAAGGGCTCGTGTTCAAAGAGTTCAGATGACCAAAGAAGCACATACCCCAAACTCAAGAAAGAGTCA
GAAAGTATCACCTGGGAACAATCAACTTACCAGAGCCTGCAGCATTGTAAGTCTCCTCTTCTCATGCAGAATT
TTGAGTGTACCAACATAATGGAAATGACAAGATTTTAAATGGCAGGAAAAGATGGATGTTAGCATTCAAGAA
AAAGGGAGAGTTCAGAAGAACAATAGCACGAAAAACAGATCTTCTGGAATGGAAAATGGATTGAGCACCT
CTGAGTCTCGTTGCCTTTCACTGTCAAGGAGAAGTCATCAAGTACTTCAGACATGTCCAAGTCCCTCTACACT
GAGTGATATGAGCACAATAAGTTATGACAGGCATTTAGAGGATTTTTCCTTCAAGACGCCAGAAAAAGGTTT
TGAACATTGCTCTAATGTGTCAACAACCACATCTTCCAAAACCCCATTTTCCATTCCACACTCAGAAAAACCG
AACTCCATTTTTTCTAGTGCTACTTTAGCACTAACATACATGTCCAATACAGAATCCTCAAGAGCAAAAGCTAG
GTCACATAGTGAACCCAGACAACGACCCAATTGGAGCATTATAAGAAAAAGCAAGCGCACACCATCAATGG
ATGGGATAACAGGCATACCTGACTCTAGAAGGGAAGAAACACCTACTCACAGCAGGAGACACAATGTCCCA
GAAAGCCATGAAGCCTGGTTACTTAAGCTCTACAAACAAGCAAAGTCTATCAAGCATGTCAAGGTTGATTCC
GCCAGCATAGTGTCCGCCGTTTGA
>PGSC0003DMG400016053
ATAGCAAAAAAAAAAAAAAACTGAAAGTGAAGTAAGAGAGAGAGAGAGAGAATTGTAGAGAGAGAGAAT
TGTGTAGAGGCAAAAAGGGCTAAGTTCTTATTTGTTCTTGATTTGTGATTTGCTTTTGGTTTTTATTAGCAGC
TTTATGATATGGAAGATTCACAACAACTGTTGAGGTACATAATTCCATCTTTCTTCTTTTATTTGAAATGATTTT
TGGTGGGAAATCCATCCTCTGTTTTCGTGTGTTTTGTCTCTGTTTTCGTCTTTTTGGTTTCTCATTTGGTGTTC
GATGTCTTTATTGGGCTTGATTAACTTAGATTTACGAAGAAATACTCTCTTCTCTAGAATGTTTTTTAACATATT
CAAGGTTCGATTTCTTGTTAAGGATGGAAAGATCCTATGAATTTTACCACACCTTTGGTGATTTCCTTTCTTGC
TTTCTCATTTGGTGTTCGGTATGTTTTTCACAATAATTGGATCCTTCTTCTTCTTCTTTTTTAAAATTATTTTTGG
TGGGAAATTCAACTGTTTTTTCTTCCTGTTTGTTGTTTTCTGAACCACAAATTGATTTTTATGGTTTTTTTTTGT
TAGTTTTCTATCTGTTAGTTAGATTTTTTTGGTTCGTCACTCGGTGTCTAATGTCAGTAGTGGCATGCTTGATTC
AGATTTGGCACCACATAAGGTCCACTAAAGAAAAAAACACTTCTTATCACAATTTTTTTCATATCCCATGATTA
AATTCTTATTAAGGATGGAAAGATCCGACACATTTCAACATAACCTTTGGTGATTTCGATTATTGATTTCCCAT
CCGGTGGCTTGTATTTCTCACTTTATCTTTGTGTGCATGTTAATATTTTGAGTCCGCAAAAGGTTTTATAAATTT
TGTCTTGTTTTGGTTGTTGTTGACGTATATAAGTTGAATCCATTTAGTAGCAATTAAGATAATTTCTCTCTCTTC
ATTTTATGTTTTTTCCCATAAACAATTTTAGTACCATGTTACATAAATACTTTTCAATAACGAAATAAATAAGTTT

GACAAATACTTTTTGGTATTATGATTTAGGTTTGAGATCTATATTTTGCACAGGAGAAAATTAATCCTTTTAGG
TTGTATTCAGTATGAATGAAAAATGTTTTATTTGGAAAATAAGTGAATCTTTTACTTATTTTAACGATCGAATAT
GAGAAAATTAGAGAAAATGTTTTCTTCCCTACCAAACACACCCTTAGTATCATTAATCTGCTTCTTCTTCTTTTT
CAGGGTGAAAAAGTAATGCTAGAACTATGGGGAAAAAAGGCAGTTGGTTTTCTGCCATCAAAAGGGTGTTT
ACACCTAGCTCAAAGGAGAAATTACCTAATGTAAGTTTATTTTACACTCTCACAAGGTCTATTTGCTACTCTCT
TCCTTTATTTTTTCTCGTACATGCGAACAAAGTTTCATATTGATAGGGAGGCTACATATAAAGTGTAGGATCTC
TTAATGGTATGAGGTCTTTTGGAGAAAACCGTGCAGGTTTGGCCCAAAGCATACAATATCACTCCTTGTTAAC
AGTATTTAGGGCTTACCATTACTAAAAAAGGCAAAAAAAAAAAACGATAGACAAAAAAGTTACGGAGTGCGT
TGCTCCAAAAAAGCGACGACAAATGGATGTTGTTCGTCGTTTTATATTTGTTTTTTTTACTAGAAAACGACGC
AGTCCGTAGCATTTGGCTATCGTTTTTCCAGGCATTTTTTAGTAGTATGCATAGACCAACACTTGCTTATTTCA
AAATTAGCTTTCTCCTAAAAAATAGAGTTGAAGGGTTAAAATGTAACAAGGTTAAGATGGAGTGTCGAAATG
AAAATTGATGGCAAATTAGAGAGGTTGTTTATGTATTTGGCCACAAATTAATGACAGAAAAATGTAATTGATG
TTTATTATAGTTTACATAAATAAGAATTCAGATGATAATAGAGATCTACAAAACTAATCTATGAACCAATTTCAA
CAAGTAGTGTCTTACTTCCTCTTGACTGAAAAAATATATGCATGTAGCTATTGAAGTTAACTTAAAATCCTCAA
AACTATTTGAAATCGAGTTGTTCGAAAGGGGAGCATTATTCACATAATATGTTTGCTCTGTAGTATGTGCAAG
TTGGCGTTGGCTTTATGAAGTCTCACTGTCCATGTCGCTACATTTAAGCTCATCTTAGTTAGTATTTCAGTGTTA
TATGTGGTGTAACATCTATATGCCTCACATGTTTCTGGAAGGATCATGTAGTACTTACCTGAATCGGACGATTG
TTGCAGGAATCAGAGAAGAAAGGCGCTAAGGAAAAGAAGAGTCGCGGAAAACTGAAGCATGGAGAGACT
AAATCATTTATCCCTCTCTTTAGAGAGCCAAGTAGTATTGAGAAAATACTTGGAGAAGTAGATGAACAAATGC
TACTTTCTCCAAGATTCACTTTACCCGCGGAAGTTGTCTCTCCTCGGATCTCTTCTTATAGGTTTGCCACTCCT
AGCGCTACGTCTCCAAGAGTTGCCTCTCCTAAGGCTTCCTCTCGCCGGATTACTTCCCCTAAGGCTCCTTCAC
AAAGGGTTACTTCTCCAAGAGCTATATCTCCTAAGGCTCATCCTCCAAGGGTACCTTCTCCTAATGTTAGTCGT
AACCGCAAAGAAATCAGCTATGCTTATAGACCAGAACCAACTTTGAGAACTCTCAATCTTTCAGCAACAAAG
ATACAGGCAGCCTATAGAGGTTACATGGTAAGTTCCTATATAATTTCGCCTTGCAAATGTTTGGCTAACTCTCT
TTTGTCAGAAGCTTGTGATTTAGTTTTTGATGCAGCAAGTAGAGGAAAACTGATTGTTAATTGAGTCCAGGC
TCTATGGAAGCAACCTCTCTACCTCACGAAGGTAGGGGTAAGGTCTGTGTACACTCCATCCTCCCCAGACCC
CACTTATGGTACTATACTAGTATGTTGTACTGTGAAAGAGTAAAGCAATTCATTCGATAGAGTGGACTGCAGC
ACACATTGTCTAATCAAATCCAATCCAATTCTCTCTCGATCAATATGTTCCTCAAAAATCCGATTCAGGGCAGA
TTCTTCTCCCAACTAAGCACAGACCCCACATTCAACCCCCCGTTTTACGGAATTTCAACAATTGCTTCATATTA
TGCTCCTTAGTCAATGGGAAGTACGGAGTGTGCATTCATCCATTGAGGAATTCTATCATATTGCTAAGTTTGTT
CTAATTGCCTACTAGGAATAACATCTTATGTCAAAGCGGAGACATGTAACGTTTTGTGACTGTCACATCTACA
GCTCAACGAGAACATGATCCTAGATAGGAAAGCATGGAGGTAGAGGGTTAGTACATAGTCGAGTGATTCCTC
CTTTTCGTGAGGGAGAACATGGTTCTATTGATCAGTATCATGGTTTTACTTGGGTTATTTGTACTAATTTTGTT
GTCGATACTTCCCTTTATTCAGTATTTTTCATTGTAGCTTTTACTCTTGTATTTGTCAAACCTGTTTTTGAAAAC
ACTTTTCTTGAGCGGAGGGTTTATCAGAAACAACATCTTTACCTCTCACAAGGCAGGGGTAAGGCCTGACC
CCACCCTCCCCAGACCCCAGAATTACACTAGACGTTTGTTTATCAATTGTTAATAGACGTTACGTTCTAATCTG
AGTGCTTTCTGTTTTGACAGGCAAGGAGGAGTTTCAGAGCTTTGAGGGGTTTAGTAAGGCTTCAAGGAGT
TGTGAGAAGTAGTAATGTAAAAAAGCAAACAGCAAATGCCATGAAACAGATGCAACTTCTCGTTAGAGTAC
AAACTCAAATTCAGTCGAGGAGGATCCAAATGTTGGAAAACCAAGCACTTCAACACCAAGCATATAGAAAC
GACAAGGAAGTCGAGAGCACCATCAGTAAATGGACTCAACTGGTATGTTTGAACCATCTTCATGAAGTTACA
TTCCTATTTAGTGGTTTATAAAAAGTCGTCATTTCCAAAACGATAGTTGTTGATGTTTGATGAACAATGTTGGT
ATAGTGTGAGGCAGGTAACAATGATAATTGGGATGATAGTTTGCTGACTAAAGAAGAAGTAGAAGGAAGGC
TGAGGAAAAAAGTGGAGGCAGTCATCAAGAGGGAGAGAGCAATGGCATATGCATATTCTCACCGGGTACA
ATAACACCTCTATCTTTGGTGGATTCAACATTTAAAGTTTTTACTATATATATCTATGCTCCATGTTGAAAAAATG

ATAAGTTCATCCAACGTATGGTCCATCGACTCTCTACATTTTGACAGTTAAGTAGCTCTCTTACAAACTTTTCA
AGGCTAATCTTGTTAAATTACCTCTTTTATCAGCTATGGAAAAATGATCCAAAATCGGGTTTGGACATGGGAG
CTAATGGTTTTCCATGGTGGTGGAATTGGTTAGAACGTCAACTACCTTCAAGAAATGCTAACAAAACTCCATC
TGCTGTGAAAGATATCAAACTAACACCACCAAGGGCTATTTCAGAGCACAAACCAAGTCCAACGCCTCTAAA
CAACGTTACTTTCAGACGTATACTCTCTGATTACGATAACAATGAATCATCAGTCACACCAATGTCAACCAAGT
CAGCAATTCCAACGAGGGGAAAACAGATGCATACTCCAATTAGAACACCACCAATGAACAACTCAAGTCTAA
AGAAGCACTCGAGAGCTCGAGCTAGTGCTTCTAACTACCCTTTTGATCTTCCATTAAAAGACGATGATAGCCT
CACGAGTTGTCCTCCGTTTTCAGTACCACATTACATGTCACAAACAGCATCAGCTAAAGCCAGAGCAAATAG
CAATCCTAAGGAGAGAAATCCAGAAAAACAATCCAATGACACAAAGAAAAGATTTCATTTCCTTTAACTCC
AAATATATGGTCATCCAAATGGAGTAAAGGCTCTGGAAAGGATCCAACTTCTCGAAAAGAAGTCGATAAACA
CGAGTCCATGGCTGATCATATAAGTGTGGATTCAACTGTTTCGATGCCCGCGGTTGTTGGTGGGAGGAGACC
ATTTAACAGATTTGTGTGATTGTTCTTTGTATGTTACCCTATTAACTTGTGGAGTTCATTTTGTTTGTTTTTTTTT
GTTATTCTATTTGTTGTTATATGAGTGCATGACGTGTTGTATTGTGTTACATAGAATTGATGTATAATAATGAAG
AAACCATAAGAACATTTTACTATGATTCAATACTTAGGTCCCAACTATTTGGTTTGAGAAAATGGAAGAAATT
GAAATTTGTGAGTGAGA
>PGSC0003DMG400022412
GAAAAAAGCAAATCTTTCTGACAATTTACACATGCAACCTCTTATTATTCTTCAAACATGCTTAAATTTTTGGC
ATCTCAAGTAAAGCCAAACAACAGCAAGAAGAGGTGACAAACTCTCAGTTCTTGTAATATATATTTGTTTGTT
TGTGTCTTTTGTGATGGTGACAACTTATCAGATGAAGAGTCTTAGTACATTAGAGCTTATGTTAGAGGAACTT
CAGCAAGAAGATGAAGGAACAAATGATTTGCCACCACCTTTGCCTGTTAGGCCTGTCATTAAGGCTAGGTTG
CCTAAGGGCAGAAGGAAATTGACATTTGGTAAAGAAAAGAGCAATCTTGAAGATATTAGGGTTCAAGAAAA
TGTATTTGTTGATCAATGGAGTACTTCAGCAGAGAGGGATTCTGCAGCCATGACAGATAAGGTAAATGGGAA
TGAATTAAAGTATTAATTTTTGTTAACTTGCACACGCCTCGACTGTTCCATACTTTCTTCATTGGGTATCTGCTA
CCTCCCGTCAGCATTCGTACTGCTTAACTCTGCCAACTAAGGTAGAATAAAAGAAAATAACCTAGCTAGTTTT
GACTTTCTGATAGAATTTGAACCATTGGATACCTGCTATCTCCCATCAACACTGGTACTACTATTTGTTGACTCT
TTGCTAGAATTTGAACCTTTGTCGATGGTTTATTCTCACTTCGTTGATCATTAGGCCACACCTCTGGATGCATT
AATTTTGTTAAATTGTAGCAAAGGGGTTTCTACTCTTTAGGTTCATGAAATTTACTATGTGATCATTACCTACAT
TAAGTTACAGCAGCAGGACAATGACTCTTGGAAGAAGTAGTACAACTAAAGCTTTTCCAGATACAAAATTTA
TCTTCACCAACTGGCAAATGTGGTTAGTTCCTAGATTAATGAATGAGGGCATCTAAATATTTATTAATTGGATG
ACAGAATGTACAAAGGAAGATTCACATAGCATCTTATTTAGACTGAATCACAGTCATATTTCTCATAATTTCAA
CTGTTGTTAGCTTACAAATTATACTTAATTTTGATGGTAAGTGTGGTTTATTTCACAGATTTGTTTGATGGTTGA
TCAGAGAGAAGGAGCTGAACTAAGTAGAGTACTGCAAATTCAGAGATGCTTTCGCGGCTATCAAGCCCGCC
AGTACTATCATGAGCTTAAAACAGGAGCAGTAGCTCTACAATCATGTAATTCACAATATCCTATTTTTTTTCTCA
AAACTTCATATGCTATAATACTAAGTCCAACCTTCAGAAAGAAAAATCAATCATATGAAACTTAATAGTTCTTA
AAAACATATGCCTCAAGGGAAAAGAACTTCAGCATGTTATTTCGCATTTTCCTTCACAATTTTGATGATCAGA
GCTCAACCATAGTTGTCGATTCTTGCAGTTGTGCGTGGTGAAATTGAAAGAAAGTATTATCAGGGTCTCACTA
GGAGGTTAGCAGCAATTATCTTTATACAGAAACACATAAAGAAGCATCATCATAAAAGAACAGAACGACAAC
GAACTGCAGCCATATGTTTGCAGTCTGGTAAGATTGTAGAATGCTTAATCCAAAATTTTGAATATATAGTTTTG
AAGATAGTCCTCAACTTTTCTCTATGCATTCTGCAGTAATTCGAGGTTGGTTGACTCGAAAGAAGTCCAATCT
CTCGGGTGATGAAAAACGATCTTGTGTTCAGAACATTAGAGAAAAAAATGACCTTGACAATAAGGAACCAG
AAACTAAGGTGTGCTTTATTTCTTTGACAATTGGTTCAATGTGCTGAAAAACACATATCTTAACCACTTTGGTT
GCTTGAATGAAACTCCCTTCAGGTACCGCGTTCAGTTCTACTTGATCTTCAGAGGCATATTTTGAAGACAGA
GGCAGCTCTGGAGAGAAAGAAAGGGGAAAATGCAGCTTTGAGGCTACATATTCAGCATTATGAGATAAAGT
GGAATCAGTATGAATCAAAAATGAAAGCTATGGAGAAGATGTGGCAAGATCAGTTAACGTCTATACAAGTGA

GCATTCCATGTCTTCATTAAAAAAACAAATGTTGTTCAGTGGCATCTCGTATGGATATCACTTCATAATGTGCT
GCAGATTACTTTATTTGTTTCGATAAAATCCCACATATAAATTCTTATATAACATCAGTGAGATTGCTTAGTGTTA
TTTGCTTTAACCAAAAGACATTTGTGTATCTTGCAGATAAGTCTGGCTGCAGAAAGGGAAAAACACGGGGA
CGAAAAGACTAAGGGAAAACTCAGACTACTGATACTTCAAGATCAAGACGAAAATGTGCATAACGGATTCC
CAAGAACTATATCTCTTCGAACAAGTGCATTGAATCATCCTGATGAGCAACCAAGCGGGAGATCAAATCCCA
AGAATAAGTGTAATAACCACCATGTGATGGACATGGTCAATCACTATCAAAATTTTGTTGTCCACGATCAATGT
AATTCAGGGGAGGAGGGTTCCGCCCTCAGGCCTAACGATGAGCTCCAGAAGCTGAAGATTAGATTTGAAG
CATGGAAGAAGGATTACAAGAATAAATTACGCGAGGCCAAAGCAACAATGAAGCAACTAGGACACTCTGAA
AGGGGGAAGGGTTCAAAGATATGGTGTGGAAGATGAAAAAGTTAATGTCTTATGTTAGATCCTCTAATTGGA
GGACATTTTCAGAGGATCTGACACCAGTGCAACAACAACATATTTGGAGAGTCCGAGCAATGTAGCCTCTCA
TTATCATTTATTCTTCATACTTCATATTTACATGTACAAAGCATATAAATTGTCAATGTCATACAACCATTGAAGA
CATATAAGAGAATATGTTGAGGAAAATAGAAAAGACTAGTAACTTCCGC
>PGSC0003DMG400019284
AAAGAGTTTAAGGGAGAAACAAGAATGCTCATAAGCAAAACACAATGATACCATCACATTTGGCCACTCATT
GTGCTGTTGTGAACAATCAAGTAATGACTTTTCTACAATTGATCACCATCTTTCAACAACAACTAATTACAACA
TTTGCTAATGCGAAAAGACGTGTGGACTGAGAAATTCAATCCGTTTTTCGCAAGCTATCTAATATACTTCCAC
AACCTAGCATTGTTTACAACAGGCTATTCACAAACAAGTTCTTAACTATCTAGCTAAGCGATTGCAGAGATAC
ATAATTGAGCACACGTACGTACTATTTCCCAATCTCCACCAACTTTGCTAACTTAGCAACCATAGTTGGGGAA
ATTAGTTCCATCTATTAACACCCTAAAGAAGAAGACGAGAATTATTTTAAGCTAAAATCACTTGCTCTTCTTAC
CCCACCATTTTCTATGACTCTTCCCTGCTTTAGAATGAACAAGCTTGTGTACTTTTGACTTCGTCTCCTTTAACC
GGACCTTGTACTCTTTTTTCCATTCCTCAAACCTGTGTTTAAGTCTTCGATACTCATCTGCAGGATTATTAGTAG
AATGTAACAGCCCTGGCTTCAAGTGAACAATTGCCATGGCTTCGTTGTCAAAATTCTGCTTCCGTTGTTCAAA
TTCCATTGTAAGGTGGCTGATTAAGGATAAACCACCATTAACCTCTCTATTAGTTACAACACCCAAGCTGTTGT
TAGAAAAGTTAATTGGAGTGCGTCCCGCAGGAGTGTCCATAGATGTTGCATCATCAGAATCATAATAGTGAG
GAGATGGGGAACCTTCAAGCTTTCCAGGTTGACCAGTAGTGTCACCAGCGGCAAGAGTGTTCTTGGCTGCA
GCTAGATTAGCCTGCTTTAACAAAGCAATCAATTACCTTGTAAATTCATAAATATGAGTTCACAAGTAGAAACT
CAATTTCCAGCATCAGCAGTATCAGTAGAGCATTCATAGAACTACGGATACCAAGAACTAGAGAAAATGAAC
TATCTAGGATCATATTGCAGCTTACTCACCTGCAATGATGTCATTTGCTTTTGCCACATCTCCTCCATTGACCTC
ATCTTGACCTCATATTCTAAGCATCGGGCCTCGAATTGGTTTGCTTGTTCCTTCAGGGCAGCATTTTCCTTTTC
CTTCTGTTCAATGGTTGCCTCGGCCTTTGCAACTCGTCTTTCGAGGTCTTCTACAACAGATGGTAGGATTTCT
CGAGGCAACTCCTGCTTGCATAAAAAATTCAAACTTACTACTACAGATTCTCAAGCAGTCAGTAAAAATATCA
AAGACTGCATTGCTGCCTCAACTGTTTGCAGCAAGGGGAAGGCATGATAAATGAAGCCATTATCCTGATTTA
TCCATCAGTGATTCTTCACCAACTGTCCTTTCCCTTTCCCCTTTTAGCTTCTACATGAGAGAGGGGAAGGACT
AATGTATAAGCCAAAAAAGGGCCTTACTACTCATTCTCTCACCATATTTTTCTTTCAATTTCAATAATCAAACAC
TCGGCATTTTCGAGATGCCAGAGTATTTCATCTTCCAAAGAAAGAATAAAAAATGAAGAAAGATTCCCCTGA
ATCCTGAAATCAGAAAAGCAGTTGCATACCCAAAAATGGTTTTAACTATCCCCCCCAAAAAAGAAGAAAAAA
ATGAAATACCTAAAATTGGCTTCATGAAACAGGCCACCAAATTCTTAAAGATATTAGTCCGGAAGCAGACTTT
ACAATTCTGAACGAAGAAACACGAAAAGTTACAATAAAATCTATGGCAGAACTTTTAAAATGCTAATTATTCC
ATTATAAGGTCTATTAAGGGAATAAAAAATGAGGCATGAGGACTACTAGTAAGGATAGGTGTGAATCTTTATC
ACAGAGTACCTTGACCTCCGTCTTTCTGCCTGATCTTCGTTTGTCTACATTTAACATTTTTGAATTCTGCAGTTT
ACGAAGATCCCTTCTAGCCAACCAACCACGAATAGCTGCAAAAAAGAAGATGTGTAAATTTATATTTGGTTTC
ATAACCAGCTGCCAAAAGAAGATGTCCAAATCTATATTTGGTTTCATATATTTGATACCATGCAAATAACTTTTA
CCTGATTGTATCTGCACAACAGCCACCAGCTGCTCATCACTTCCTTCACGAGCAACCTTCCCTTTAGACATCA
CTTTAGTATTATACAGCCTTCTTTCTATTTCACCACGAATAACTATATGAACAAATGTTGACATCAAACATCAGG

TACAATTCAGCATTGAAAGCTTATAATTTAATTATATTGATGCATCACCTCAAAATTGACCCGGTTCACATAACT
AGTTACTTTTGGAACTTCCAACAATGGTTCAAAGGCAGTTCATTATAGTTTTTTTAAAAAAATTATGAAGACA
AAATTGTAGTTATCTGTTTTTCTTTTTCTAAAACCACTTGAATGGCATCCCCAAGTGCTCAAAACTTTGAAGAT
ATCTAATACAGTCTTGAAATGGAAAAATACTCATACAACCTGATAGACACAACATTCTTCATGAATATCATCTTC
TATTGTATTATAAGAAAAAAGGGAAGTAAAAAGACGAGTTGCAAAGGAATCTGCTTTACATGATTGAAGTAT
GATTACTCCTCCTTTCAGCTCATGGAAGTGCCGACGAGCACGATGACGACGGAAGCACTTCTGCACCTCAA
GAGTACCTTGCAGAACTTGGTTCCTTACATCCTCCAATGAAGCAATCTACATGTAAGAGGGACAGAAGTTCA
CATATAGGCACACAAGGAAATCCAATCTTCAATAGTATGTGTCCATTATCTTACTAGAAGCCCATATTGGGTATA
TGTTAGAGGATATGTTATATCCATAAGCCATAACTTTTGCCATTTTTATTGTTTGACCACTCAAAACCCTTCTCC
AAACAGACATATGCCTCCAAAAATATGATTTTTCCTCAAAGTGGTGCCACCAGTTTGGAAGACACCACACTTT
TTTCAAGATAACTATGGTGTCTGGGCCAGCTAGCGTGCACATCGACACACCACATGTTTTTCATTTATCATTAT
TTGGTTCAACTATATAGTAGTGTAATGATTAGAAGAAAATTAAAATTTCTGGTGCTTCCTTTAGATGATTTAGT
AAATGACATAAATTCTTTGAGAAGTAAACATATCCACACAAAAGAACAAGAAACCATGAACTCACCTGTCCT
GATCGGAAATATAACTTTGTATACCCAACTTGGTACAGTTCCGGAAGAATACCAAATTGATGAAGAATGGCAA
CTGACATACTTAAAGGATCTTGGCATGCATTATCCTTTGGCAGAAGGAAGCCGTACCTAAAATACCGCAATCA
CATATATAAGTTGACAAACAACCAAAAGAACAAATCAGCAACAAACTAAGGATCGTGTATGTCACCTGCTTGT
GAATTCTTGATGTGTTAACCTAGTAGGATAGCCAGATCTTGATATTCTAACCACTTCAAGAACACCACAGCATC
TGAGCTGTTCTATGACAAGATCTTTGTCAGACATGCCAGGAAGCTGCTTATTATTTGGTTTTATGCAACATATG
AAATGTGGTATGGTATTTTCCAATTGCTGCATCAATTTGAACAAATTATCCTGCCAGAATTTGATAAAGGACAA
CATTGAGAAACAATCATAGCACCTCAAGATACAAACTTTTATTTTCCAAATGTTTGGTTTGTTAGCATGATCAA
GTTCTATAGAAAATAACCACCTTAAACTTAGTTGCAACACTTTGCTTCTTAAAATCTAGCACCCCTGTATCAGT
TGATGGAATTGAGAATTTTTTATCTTCAGGCAGGTGACTATCACTTGATGAGAGTAGCTGAATAATGTCAGGA
TGCAACACATCTCTGTTCTTTGCTAAGAAGCCAGTTGCATCATAAGTTACCTGCAAAACTCATATTCGTAATTA
TAGAGTATGTAAAGAACCAAGGAAGTCAACCATAACTTCTGTCTCTAAAACAAATGATGTGGCTTTGTTGCAC
CAAAAAATAAGCAAAGACATATTTGAATTAAAGGTTAATTATATAGTCCTTCTTGCCTTCAAAAGCTCTAGCGT
TTCTCTCTCCAAATTTACCCACCATACCAATACTGAAATGGACTTCCAGATGTTCCTAGATATTTGGATAAGTCC
GATTCAAGCCATGATAGCAAAAGGTCCATTGATATTTAGGCATGCACCAGTCCCCAAAATATTAAGCAAGAAA
GAATATGTTACTAGAACTACTGAAAGATAACCAAAATAGAAAGCGGTTGTCTGAGATTATTGAAATAGAGAA
CAATTACATGATTCTGGAATTCTTTTCCACTAAAAAGCGCTAGTACTGGCACAAAGAAAGAAAGTTCTTCTTG
CAGCACCCATGGATAAGAATCTATCATCAATCACCAGAAAGAGGTATGATAAAGTAAATCTTCCAGATATTATT
GGTGTAGTGGTGTAGTTTGGATTGAAGATGGAAAATCACTTGTGAGAAGTTATAAGAAGAGTCATCACAAG
ATACAGCTAGAGGGTCACTGGTCAAGAGGCACACCCCCATTGGTGGTAAATGAAAACAAGCATAATGCCAC
CAAAACAGTAATCGAAACAAGGATGGTGCCGCCAAAATGGTCACTGTAATAAAACCATGAAAAGAAAGAAA
AACTCTGCCAACAATTGAGCGTAAGTCTGACCATGGGAATGTGAAAGCTATTAAGTTTCTTAACCAAAATCGT
GAAAATACTACTAATGTGGGTAGAAATTGAACAGGACTAGAAAGGATGCATTTATAAGAGAAACTGACCTCT
CCAGCATAATGACGAATACAAAATTCTTCTCTTTCGCTTTTAAAGCAAGGGCTAGATTTGATGTGCTGCTTAA
GTTTACATGCAAAGGTCAAATCTGTGGCTTTTAGGGAATTTGATTCTTCATTCAACAAAGATATAAGGCCAAT
TGGCTTCTGCAAAATGAAAATGGAAGTTCATCTATCAACTGATATGTCTATTCAAATAAATGTTGAAGATTGCT
CAACTAAAATACAGAGATCTTCTTTATGATTGACATTGGTAATTCGACTCGAGATATCGCAATAGAATTTGTGA
GTTGTGACTACATAAGCATATTCATTTTTATCCTTCTCATACAACTCAACAATTAAGCACGGAAAGAGTAATCA
GAATATGCAAGACTTCACTCGAAAGAAGTAATATGACAATGACAAAATTGGTGTGGAAGTTATATCAAATACA
ACTTTTTTTTGAGATAAAAAAGAAAGTTGTATTTGATAATTTTTACAACTTGTGACAATACAAACAGATAATTT
TTATCCTTCTCATCCAACTTAATAATTAAACATGGAAAGAGTAATCAGACTAAACGAGAAAAAGATGTAAAAA
TCAAACAAACAAACAAAAAAACAAAAAGAGCAACAACACAGTGACATCTAATTAAGTACGATTGTAAACAT

GTAATCCGGGGTAGAAGAAAGTTGTCAGCTTATATAAGCTCCTGACGCCAAATAAATAGGAAAAAGAACCA
AAAGGAAGAAATACCTTTTCAAAAAGATTCAGACACTCTTGGTTGTCTTCAAAATCTACTTTTGACCAATCAA
TTCCGTCCAATTCATATTCCTGAATCATAAAACATCAGTTCCTGCATTGACATCTAAAACTGATACTGCAAGGA
AT
>PGSC0003DMG400004701
GCTAGATTAAAGTCCTGTCTTCTACTGATTGACAGAACCTAACAGGGATATGACCGTCCAAAATCATGAACAG
AGACTACTCGAGATTAACACACTTGAATGGGATGATCTGTTGGCACCTGGAGATCCCAACAAGATAGTTGCT
ACCCAGCAAGGTATATGCTTGATGATATGATGTACCTTATAGACATTATTTATTATTTACATTCATAAAAATAGTT
ATTTATTAAGTTTTTACATTATTTTGCAGCAGGAAGTAAAACTGCTTATGTACAGCACACGTCGTATGAGCAAC
ACAATCTATGTGAATTAAATGGTTACAGCCTCAATGTGAGTAGATATATGCAGCTTGCTTCACTGGTGTAACTG
TCCTCCAATTCACATTTTTCTCTCATCATTTTTATCTTCTCTATGTTGCTGTTATGATCTATTTATATCTACACATAT
CTTATGCTATCAATGAAAAGACAAGTAGTGTTCTTTGATTTAGAAAAAATTGTAGTTCTCCATTGGTGGTTGTA
AAAGTATCTTAGGGAGGAACCTTTCCCCTTGAGCCATTTTAAGGAAATATGGTTCATCAGAGAGCAAGCTCT
ACAAAAGATGTTCTACTTCATTTTCTAAAATAAACTCTCTTCATCTGAGATTAAATAACTTAGTGTATACCTTAA
AATCAGAAGTAGAGAGTTATGTAGAACTACTTGTATGTATTTATGATCAAATAAATTGACAAGTTGATTTTGAT
ACTTAGATTGAAATTCTTAGAGCCCGTTTGGATTGACTTAAAAAAAGCAACTTTTAAAAAGTAACTTTTTAAA
GTGCTGGAACTTATTTTAAAAATAAGTAGTTATGTGTTTGGATAAAAGTGCTGCAGTTGAAAAAAGAGTTGT
TGATGTGTTTGGCAAATAAGTGTTGGTAAACACTTTTTTTAATCAAAATGTCTGAAATACCCTTTTAAGCTGTT
AACATGATAAAAAGTTGATTAATTTAAGGTTTTTATACTTAAAAATAAATTTATATTTTTCATCCATAAGTAATAA
TATTTTCCTATCATTCACATATTTCTTTACCACCACAAATTATTTATAAGAGGAATAATAATAATAATAATAAAAA
AAAAATAAAAAAAAATATAATAATAATAAGAATAATAATAATAATATAATAATAATAAGAATAATAATAATAATATA
ATAATAATAAGAATAATAATAATAATATAATAATAATAAGAATAATAATAATAATATAATAATAATAAGAATAATAAT
AATAATATAATAATAATAAGAATAATAATAATAATATAATAATAATAAGAATAATAATAATATAATAATAACAATAAG
AATAATAGCAATAATAATAATAATAACAATAATAAAAATAATAATAATAATAATAATAATAATAGATGTTAGG
GTTAATTCAATATGGGCAATTTGGGAAAATGTATAAAAGAATTAAGGGCATAAAGGTAATATACTTGGTCAAC
ATAAAAGGGCTTTTAAGTTAAAAAAAAGAAGAAGCTCCCCTCCCCTAGCTTTTAGCTTTTGGCTTAAAATAA
GTCATTTCTGACTTAAAATAAGTCACTTTTGATAATTGTCAAACACTTTAATAAGTCAAAAACTGGCTTTTAAG
CCAGTTTGACCAGCTTAAAAGCCCATCCAAACAGGCTCTTAGTATCTTGCTTTGCACCAACATGCAGGAGAA
ATACTTCCATCTTATTGTTATTACCAAAAAGGAAAAACCCAATAGAAATCCTGTTTTATTGAAGTTTAAAATCA
ACTATATACTATGTGGTTTGCGAGCTATTGCATAGGAGCGGGGGTTTTACCTTGTGCGCACCCAAAGGGTAG
CGGCTGCGGGTTTCCCTTGTCATAAAAATAAAAAATAAAAAAATCAACTATATACTATAAGAAGCTAGAGTAA
TCATCTTGGTGTAGGAGTGTGGTGACTTGGAATGAGATACACCTAATCAAGAGTAGTAAAACAATGTCTTTCT
AGACTTTAGCAACTCACATGTACGATGTACTGTAATTTGCTTACTCCCTTGAACACAACTTTTTTTGAGGTTAT
GGTGTTCTTAATTTCAATTTTGCACACTTGAATAAGATTATCTTCCACCTATTCCTTGCAGTGTTCATTTGTTATT
TAATTTCATGCATTCACACACCATGTGTATATGTGATCTGATCATGTTGTGTATGCTATCTATGTTATCATTTAAG
GATCAAAGTTTTACTCCTGCTTTAAATCCATCCTTAGCAATTGTTCCTCCTGGTTTTATCTTTCTTTCGTTTGAA
CTGAAAGATGCTTGGTAATATCTTCTTTTTTTCCCTTTCTTTTAGTTGGCTACTAATATTTGGTACTTGTAGGGT
GTCTCCTCTTCTCTTGAGAGAATTTCTACAGTCAACAATTCAAATGAGATCATCTTCCAGACAGTGGATGGTC
AAATGACTCCAAGTTTTGAGAAGAATGAGTCTGGAGTAATGACAGTAAGCACAGGTGATTCCTTTGATAGTC
TGAACCAGGATAGACTTCAAACTCAGGATAGCTTTGGAAGGTGGATGAACTACTTTATTACTGATTCTCCAG
AATCCACAGACGATCCAACTCTCGAATCTTCAGTGTCAACAGGTCAATCATATGCAAGGGAGCAGACATTCA
ACATAACAGAAATATCGCCTGCGTGGGCTTCTTCAACTGAAGAAACAAAGGTCCTTTCTTCACACACTTGCT
ATGCTGATACAATGGTTCTCCTTGCAAGAAGAAAAATGTTGTCTGTCATGATATTGGCTTTAAGTAAACTCTAT
AAAACACAAGTGATCAGAAAAAAAGGAAGCTTAATTACTCTTCATGTGTTTGTGTTACTTTTTCCATTTTTAA

AGAAACAAGAAAAATGTGTTCAATATCATTTTGCTTTAGAAGAAAATAAGTGCTTTAAACCTGACCTGACATA
ACATTTTAGGTTGAGAATTAGGACTAGTTAGTTTTTTCAGGAAAAAATGGAGAAAAAGGTAAGCATACTGTG
GCCATTGACTTGTTTTGTGAATGTGATATGTTCAATTATCAAAGTGATTTTTTTTTAACCTCTTTAAACTTTTAG
ATATTATTCTTTTAGTTTGCCCAAGTTCTTTGCCATGGTCAAGAAGGTCAAGATAAACATGTATTTTGAGCTAT
GATCTAGCAACTATACCTTTTGTCTCTATGATTACAGATCATTGTCATTGGGCAATTTCATGGAGAACAATCAC
ATCTGGAAAGTTCCTGCTTGCATTGTGTTTGTGGAGATGCTTGTTTTCCTGCAGAAGTTCTGCAACCTGGGG
TATATCGTTGCATAGTTTCTCCTCAAACCCCTGGACTGGTAAACATATATTTAAGTTTCGATGGTAATAAACCTA
TTAGTCAAGTCATGAGCTTTGAGTTTCGAGCTCCTTCAGTACAAGTTTGGACAGAACCCCCGGAGAGTAAAT
CTGATTGGGATGAATTTAGAAATCAAATGAGGCTTGCTCATCTGCTGTTCTCTACATCTAAGAGCCTTAATATT
CTATCCAGTAAAATACATCAAGATTTGCTGAAAGATGCAAAAACATTTGCTGGAAAATGTTCTCACATCATTG
ATGATTGGGCCTGTCTGATCAAATCAATTGAAGACAAGAAAGTCTCTGTCCCACGTGCAAAAGACTGCTTGT
TTGAGCTCTCTTTGAAAACCAGATTACAGGAATGGCTGTTGGAAAGAGTTGTCGAAGGATGTAAAATCTCA
GAACATGACGAGCAAGGTCAAGGAGTTATCCATTTGTGTGCTATCCTAGGTTACACTTGGGCTGTATATCTGT
TTTCCTGGTCCGGTTTGTCATTGGATTATCGAGACAAATATGGATGGACAGCTCTCCATTGGGCTGCATATTAT
GGAAGGTATCCTTAGTTACAGCTCTGGAATGTTGCAATTATCTATCATTCAATTCTTTCTTTTCTATTTGTGGTT
CTTTTGCAAATGTTAACTGTTTAAAATAGTCGTTACACCCTTGGTTGTGTTGAAGTTGAACTGCTGTCCGCCA
ACCTGTCTGACTCATTTTCACCTCATTTCTTCGCACCAGCGATAAAGAAAGTAGAACTAACATGGAGCATATAT
TTTCGATTGGTTTGGTCATTTCAAGTGTAAGGTTGTTAATAGTTTTGAGCATTTGGACTGTCAGGGAAAAAAT
GGTTGCAACTCTTTTATCTGCTGGAGCAAAACCAAATTTGGTCACAGATCCCACTTCAGAAAACCTTGGTGG
ATGTACTGCTTCCGATCTTGCATCTAAAAATGGTCATGAGGGTTTGGGGGCTTATCTCGCTGAGAAGGCTTTA
GTTGCACAATTTAATGATATGACATTAGCTGGAAATATCAGTGGTTCACTCCAGACGACTACTGAGTCAATAA
ACCCCGGGAACTTCACAGAGGAAGAGCTAAATCTGAAAGACAGTTTAGCAGCTTATCGTACAGCTGCAGAT
GCAGCTGCACGTATCCAGGCTGCTTTCAGGGAGCGTGCACTGAAAGTGCGGACAGAGGCAGTCGAGTCTT
CAAATTCAGAAATGGAAGCACGTAATATAATTGCAGCTATGAAGATTCAGCATGCTTTCCGCAACTATGAGAT
GCAGAAACAGCTGGCAGCTGCTGCACGAATACAGTATAGGTTTCGAACTTGGAAGATGAGGAGAGAGTTT
CTTCACATGCGCCGTCAGGCCATCAAAATTCAAGTAAGTCCCCCTTTATTCTTTGGCTTTCCCATAAAACTAAC
TGGATAACATGATCCATCTCATGCATCAAAGGACATGAAGTTTAAACTAATTTCAAATAATCAGTTAAATTTATT
AGTGTGTTTCAACTTTGCCCACCAAACTGAGGCGGGCAATCATGTTCTGCAATTGGAAGCGTGGACTATAAC
CTTTGTTTTCCTGAAAATAATGGAATACTAGCAGATAACCTGAAAAGGTTCCTCGTTGTGAATATGATTTTTGA
TGCTCCTGGATTTCAAAATTCTTGGAAAAACAACTCTGTTATTATGTGTGTCTACTACTGTAGACAGCAGCCTT
AATTTGGAACCTGCTTCTGGTTTAATGCAGGCTGTGTTCCGGGGTTTCCAAGTCCGAAGGCAGTACAGGAA
GATAACTTGGTCAGTAGGAGTGCTTGAAAAGGCAATATTTCGGTGGCGTTTGAAGAGGAAAGGTCTCCGCG
GGCTTAAACTCCAGTCTAGTCAAGTAGTTAAATCAGATGATGCGGAGGAGGATTTCTTCCAAGCCAGTAGGA
AACAAGCTGAAGAGCGTATTGAAAGATCTGTTGTGCGAGTTCAGGCCATGTTTCGTTCAAAGCAAGCACAA
GAACAATATAGGAGGATGAAGTTGGAACATAATAAAGCAATGGTAATTGTTAGGGGAAAAAACTCAAATATA
CCATCAAACATAAATGAGCCTTTCTCAAAGTTCGATAGTATATTTGAGCCGTTTCCCTATTTTGTGAGATACAA
TCGGATACAACATTTGAGTATTTTTATCACCTACATTTTACCAACTTTTTTTTTTTTTATTGCAGCTGGAATACG
AAGGGACTCTCAATCCTGACACTGAGATGGACTAAGAGGACAGTTTACAAGCATTTCATATATTTTGGCTGCT
TCAAGGGCTAAAGCATTGGGTTTAGGCCAAAGAACCAAAATCCCCCAAATTCAGTTACAAAAACATAACGA
AGATGCACAAAAAAGAAACAAATTCCAGCAAGAGGCAAGATTAAACGAAGAACCAAAAGCTGATGAAGGT
GATATATATATATATATATATATATATATATACATTAAAGAGATAACCAGATCGGCATAATACCTAAGTAGATTATAA
AGAAATTTGTATTTACTGTTATTACTCCGTCCGCTCCTTTTTAATTGTCATGTATAATATAAATACTTATTAAAAA
GGAACGGGGGAGTATTAATTAAAATTTTCTTAATAGTTTGTTGTAGGGTTGTGTATCAGTCGATTTGGTTCGG
TTTTGAAGTTTATCGCTTTGTAAACATGTTAAATCATTAAGAACTATTAAGATATTGGTTTATCAGTTATTT

>PGSC0003DMG400034313
TGATAAGAGTGTGCCTCTGTAGGTGAGAGCAGTGAATCTCATCATCAAGATCTGACCCAGGATGGTCACATG
TGGAAGGATATGCTGGATCACTATGGGGTTTCTGCATCTGCTGAATCACAAACCAAATATTTGCACAAGTTGG
ATGAGAATGTGAGAAACGTATACTTTGTAGTTAGCTCTGTTTCATTAGGACTGTAGATGCATATAACATCAAAA
TATAGTGTGAGATTTGACCTTATTTCAGGGCCTTCTGGCCAAATACAACATCTGCCTCTTCTCATATACTCATAT
CAGCTATACAATTCAATTTTAAACCTAGTCATCTTTTTTCATGTCTTACGTACCTTAGGCAATGCTTCAAACCTC
ATCAGAGAGAAGGGCCATAGAAGCATATGAAAGTTACAAGTGGTGTGATTTCAGTGACAGGGAGGCTCAG
ACGGGTAATAATTTTTTTCACCTCCAGAGGATAATTTATGTCGACTACATATAGAATGTCAGGAATTAGTTAAT
TTCTGGAGAACTTGTATTTCCTTGCAGCTCCTGTACCAGCTTTTAAGCAACTCGAAGATTTCAAGTATACTACA
TATCCTCCTGCTATCACTACTTTTGGATCCAACCCTGACGAATATACAACAATATTTGACCAAGATCAGATTGG
TACTTCTCTTGAAGATGAGATGAGCTTAACTATTGCTCAGACGCAGAAATTTACTATTCGACATATATCCCCTG
ATTGGGGGTATTCATCTGAGGCAACAAAGGTATATGCCATGATTTAGTTGATTGTTGCTGTTTGTACCACAAA
ATTTGTCTGCGCTCCATGGTTTTGGACTTGTGGTGTTCGTTGAACTTTTGTTTTCTGCATGTGCAAGTAATGTT
GAAATGTCTCCAGTGGATTGCTTGTATGTTTACAACAACATACGCAGTATAATCTCACAAGTGAGGTTTGGGG
AAAGTGGGGGTATACAGAGACTTCACCCTACCTTTGTTGGGTAGAGAGGATTGCTTATATGTTTGTTTAGTCA
TTTCAAATTTCAGTATTTTAATTTCTATGCCCCAATATCTTATGATTATTATGGCTTCCAAAGCACACAGATCTCC
ATTATTTTGCAATTGGCTACTTTAAATGGTATTACTTCAATATCTTAGATGGAATGCTTTTATTTGATGGCTTAAT
ATGGCTCATTATTGCTGGATGCTGAAAAATAATATCGCAATTTAAAGGAATAGAAAGGTCAAAAGGAAGAAG
AGCTACTGCTTGCTCCAGCAACCAAGCCATGATGGCTAATTCAGCAGTCTTTTATCATCCTTGAAATGCTCATG
CTCGAAAACCCCTTAAAACTTAGATATCCCTGTAAACTTTGATAGCTTTCCAAGAAAGCTGTTTTAAATCCTTT
TGTATGTACCTTATATGTATGACCATGCTCAGGATATCGACAAGTTAGTTACTATGGTTGTATTTAATATTTTTCA
GCTGATTTGCATCTAGTTTTTTAAAAATTATTATTATTTTGAACGTTCTAGCTGTTCAATGCAGATTGTTATCATT
GGATCTTTTCTTTGTAATCCTTCAGAGTGCACGTGGACTTGCATGTTCGGTGATATTGAAGTTCCTGTTCAGA
TCATTCAAGAAGGTGTCATCTGTTGTCAAGCTCCTCGTCACTTGCCTGGTAAGGTCACCCTCTGTGTTACTAG
CGGCAATCGGGAGTCGTGCAGTGAGGTGAGGGAGTTTGAATACCGGGTAAAGCCTGATGATTGTGCTCGA
AATAATCAGCCTGATGTAGAAGGAGCTTATGGAAGTACAGAGGAACTGTTGTTACTTGTCAGGTTTGTGCAG
CTGCTTTTATCAGACTTATCTGTCCAAAAGGGAGAAAGCTCTGAATTAGGCAATGATTTCTTGGAAAAATCCA
AAGCAAGTGAAGATTCATGGTCCCAGATCATTGAATCTCTTTTGTTTGGATCTTCAATGCCAATGGTAACCAT
TGATTGGCTTCTTCAAGAGCTTTTGAAAGACAAGTTCCAGCAGTGGCTTTCTTGTAAATTGCAACAAAAAGA
TAATCAGATAGGCTGTTCTTTATCTAAGAAAGAGCAAGGAGTAATTCACATGGTTGCTGGATTGGGATTTGA
GTGGGCGCTGCACCCAATTCTAAATGCTGGAGTCAGTGTTAACTTCCGTGATATTAACGGCTGGACGGCCCT
GCACTGGGCTGCACGCTTTGGGAGGTAATCTATGTTTTTTGCTGTTTGGTAAATGACTTCTCTATTCACTGTAC
TTCAGTTCGCATGTTATGTTGGTATCAGTAGCAATAGAAGCTGGATAAAAAACTTTTAGGAAGTCAAACATGT
CTCGCTTCTAGCCCCCAGGGCTTTGGTCTAGTGGTAAGAGATGCAATGCGTGATATGAGGGTTAGGTACGTA
ACCTGTGTTCGAACCCTGACACAAACAAAAGCTTGTCGTTTAAGTGCAGAAAGGCAGAGGGGCATCAGCC
AAGAAGTTTTGTTCCGTGCACCATCGGGCTATGGATTTTTCAGTTATTTTAAAGATTAAAAAACCATATCTCGC
ATCCACATTCTGTGTACAACATAATACCCAGTGTAAACCCATGAGTAGGGTCTGGGGAGGGTAGAGTGCGCG
CACCCTATGTTGCGCGGACTCTTCATTTTTGCAGCCGAACCCGTCTCGACGCGACACTGGTGCGGGCGCGG
GTGCAGGGTGCGTGTCGGATTCGGTCAATCCGATCCGGATACTTTGACCAAAGCCGAGAAAAAAAAATGGG
GAAGAAGAGATCAATTTCATGGCCGGCGAAAGTTGACGACGACGAAGTTGTCACAAGGTCTGGCTGCTCC
ATGGAAAAGAATAATGAAGAGGCAAAGCTTACAAGAATAGAGAGAGAGCAGGCATATCTTTTTTCTTGTAC
AAGAACAGAGAGGAGGCGAAGGGCTCCATACGTTTTACAAGATATTTAATATTATTATTATTATTATTATTATTA
AATTTGTATATCTATAATTGATATGCTTTTAAGTATAGCGTTTTACAAGATATTTAATATTATTATTATTATTATTATT
ATTAAATTTGTATATCTATAATTGATATGCTTTTAAGTATAGCGTTTTACAAGATATTTAATATTATTATTATTATTAT

TATTATTAAATTTGTATATCTATAATTGATATGCTTTTAAGTATAGCGTTTTACAAGATATTTAATATTATTATTATTA
TTATTATTATTAAATTTGTATATCTATAATTGATATGCTTTTAAGTATAGCGTTTTACAAGATATTTAATATTATTATT
ATTATTATTATTATTATAGCTATATTTTATAATATTGAATAATTTTAGCAGAATCCCCGCACCCGTATCCATACTCG
GATTCGCACCCCCGAATCTTAAAATTTAAATTTTACCGAATCCGACTCTCGGATTCGCATCCGTCTCGGATACC
CGCACCCGAGTCCGAGCAACTTAGCGCGCACCTTACCTCTACCTTGGGAGGTATAGAGGTTGTTTTCTATAG
AACCCGACTTAAGGAATAGCATATCAAAGCATATACTAGAAAAATGCATGACAAAGCATTCTGGAAAAAAAA
CAGTAGCTACAACAAATAGTGCAAAATCGAAGTACAAGAGATAGTAGATAGTACTAGAAATCTGAGGACAAG
CACTATTGGAGAAATACTACGACTACTGGAGGGAAAGGATAAGCAAGACTACTAACCTTCTACTTTTTGTGTA
TGCATACAAAAAGTATGAGCATGGATGATATTTTGAATTTTGCCTCACTCACTCAGTTGCAGCAGTTTTATGG
AAACTTTTTGTCTCGTTCCTAATTTGCCTATGATTCGGGCTACGAGGTTGAGATGGTTCGGGCATGTGCTGAG
GAGATGCACCGATGCCCCAGTGCTGAGGTGTGAGAGGTTAGTTATGGATGAATTTAGGAGAGGTAGAGGTA
GACCAAAGAAATATTTGGGAGAGGTGATTAGACAGGACTTGGCACAGTTGCAGCTTACTGAGGACATGATC
TTATATAGGAAGGTGTGGAGGACACGAATTAGGATAGAAGGTTAGTAAGTAGGATTGTGTCCATACTAGTAG
GTAGGACTTAGGAGTGCTTTGTCTTGTTGCCCTTACTAGTAGTCTGAGGACTACTCTTGTAGTTTCTTGTTCTT
CGATCTCTGTGACTATCTATTGTTTCATGTAGGTTGTTTGTAGTAGTATTTTGCTGTAGTACTGTTATGTTACTAT
TTATTGTCTATTGTACTTCCATTATGTCTTTTCTTAGACTATTTTTGTCTTGAGCTGGTGTTTTATCGGAAACAAC
AGTATTTTGCTGTAGTACTCTTATGTTACTATTTATTGTCTATTGTACTTCCATTATGTCTTTTCTTAGACTATTTT
TGTCTTGAGCTGGTGTTTTATCGGAAACAACAGTATTTTGCTGTAGTACTCTTATGTTACTATTTATTGTCTATT
GTACTTCCATTATGTCTTTTCTTAGACTATTTTTGTCTTGAGCTGGTGTTTTATCGGAAACAACAGTATTTTGCT
GTAGTACTCTTATGTTACTATTTATTGTCTATTGTACTTCCATTATGTCTTTTCTTGAGCTGGTGTTTTATCGGAA
ACAACCTCTCTACATTATCACTGAGGTAGAGGTAAGGTCTGCGTATACACTACCCTCAGACCCCACTGTGTGG
GACTACATTGGGTATCTTTTGTTGTTCCTAATTTGCCTTTGTGAAATTTTAGAGTTGAGCACATTAGGCAATAT
TTTCAACCTTTTAAATTCATTATGGAGTAATATATTGATCAAATGGACAGATTCAGCTTTGAAACACTTCATGG
ATGTTGAGATATCAGGGTGGTGTGGGTTAGGCGCACGTCACAGGCCCATTAGGCACCGAGTATCGAATTTTA
CACCGCTGACAGGGATTTCTTGATTATTCAAGAAAGGAACATAACAATAATATTAAAGAGAGAGATATCAGG
GTGAACTCCTTGAATCTTTCTCCCTTTATATGTCTGTCTATGTTTGCATGTGTAGTGGATTTTCTTTTGACAATC
TCTATTCTCTACAAGGGCCTTATGTGTGGTACCCATAAATAGCTAGTTGACTAAATATCCATATCTGTGACAGG
GAAAAAATGGTTGCTTCGCTCATTGCATCTGGGGCATCGGCTGGAGCTGTTACAGATCCCTCTTCACGAGAT
CCAGTTGGTAAAACTGCTGCATCAATTGCTTCCAGCTGTGATCATAAGGGACTTGCAGGATATCTTTCAGAG
GTAGCTCTCACCAGTCATCTTTCATCCCTCACTTTAGAGGAGAGTGAGCTCTCAAAAGGTACTGCTGATGTG
GAAGCAGAGAGAACTATTAGTAGCATATCAAACACAAGTGCCACCATAAATGAGGATCAGCGTTCTTTAAAC
GACACGTTAGCTGCAGTCCGTAATGCAGCTCAGGCTGCTGCTCGTATACAGTCTGCATTCCGAGCACATTCGT
TCCGCAAAAGACAAGAGAGAGAATTTGGTGTCTCTGCTAGTGGAGATGAATATGGTATCCTCTCAAACGATA
TTCAGGGGCTTTCAGCTGCATCAAAGTTGGCATTCCGCAACCCGCGAGACTACAACTCAGCAGCCTTAGCTA
TTCAGAAGAAATATCGAGGATGGAAAGGCCGGAAAGATTTCCTTGCATTCCGCCAGAAAGTAGTGAAGATA
CAGGTGCTTTAAATGTTTGAAGTCAAGATTGATATTTTAAGCCTCATTATACTCGAAAATATGATAGTTAAGCG
GTGATAGACTTCAAATACAATCTCTCACTAATAAAGAAGTGTAATCATATTTATCTTGGGGGAAAAAATAATAT
ATATAAGTTGGGAACATTCTTCAATTTTTTTAGTTTTTTGGTTTTTGGGAGGGTGGAGGATGGAGGATGGAA
AATGTTTGGAGATTAGAAAATAATTTGTTCAGCAGAGAAAGTGACTTCCAGGTGATTTCCATGGCAGGCTCA
TGTACGAGGCTATCAGGTTAGAAAGCAATACAAGGTATGTTGGGCTGTTGGAATCTTGGAGAAGGTGGTGC
TAAGGTGGCGTCGACGAGGTGTTGGTCTCCGGGGATTTCGACATGACACAGAATCAATTGATGAAATTGAG
GATGAAGACATTCTAAAGGTGTTCCGCAAACAAAAAGTTGATGCTGCTCTTGACGAGGCTGTCTCGAGAGT
TCTATCAATGGTTGAATCTCCAGGGGCACGTCAGCAATATCATCGGATTCTTGAGAAGTATCGGCAAGCTAAG
GTGAGCTAAAGAATCATTCTACTACGTCATTTTTGTGATTAATATACATGAGCTAGTGAACTTCCAATAGGTGA

ATATTTACTTAGTATTTTGCATGTCGAATGCTTTATGTACATTTTGGGAATGAACTAAATCCTTTCTGTATCACCT
TTGTGAGATTTTAGGGGCACAGATGCAGTAAGTATATATTCCATTATGCTTTCAATTTTCTTTTACCTTTTCTTG
AAAGGATGGAAAAAAAAAAGGTTTTTGTTATTCCCATCTACTAGTGTCACATAAATGTAAATATTTGATTTCATG
GCTTTAGAGAAGATGTTGTCGTAAAAATAGTCAGGTTCTGGAGTAGCTACATCGTATGCAATGGAACAAGGA
CAAAGAAAAATCCAAATATGACGATATTGGTGGCAGTGTCTATGCAACTGGAAAAATATGAGTGAATGAATG
TTCTTGTGTGTCTGAATTATTTACAGCCTATCTGGGATAGGGGTGGAAACATAATTAGCTTCTTGATCCATGTG
TTTTGTCCACAGCATACTCCATTCTTTTGAAGGAAAATTTCCTTTTTTAAGGAGGGGGATAGGGAGCATTTA
GTTCTCCTTTCATAGTTCGAAATCAGGAAAATCGAATCAACTAATAATCTTTGTTGCACTGCAGGCTGAACTT
GAAGGAGCGGACAGTGAAACAGCATCAACTGCTCATGGAGACATGTCTAACATGGAAAATGATGACATATA
CCAGTTCCCTAGTTATTAATCCAACTGACCTCTTCATTGTCATCCAAACTAGTTTCAGTAGGTTCAAGAATTATA
CATTGAAGTTTGGTTATTTCAATTTGTAAGTCATTGTAAGGTATGTATTTTGTAGGTGCTTCTGTTCATAATATG
TGTATAGGTTTTATAGGTTCAAAACTTGAACTTTATAATATTGGATGCCTGCAAGATTAGCTTGGAAGTTTTAT
TTTTCTTCTCTTAACATGTGCTTGAG
>PGSC0003DMG400016486
TATAATCAACATAATGCATGGTATTCCAGGGGCCTCTTGCTACCGTAACGTCTCAAAATTGGGTTACCAGGCTC
TTGAACTTAAAATACTACTGCAAGCAGTAGTAAAGTTGCAAAGGTGGTGGAGATGTAAATTGCTTCATGCAC
AGAGAACAAAAGCCGCAGTTGTCATCCAATCGCATGCCTTAGGATGGATTGCCCGGCAACGTGCTTCAAGA
AACAAGGAACGATTACTTCAAGCAGTATTAAAGTTGCAAAGGTGGTGGAGAAGTAAATTGCTTCATGAACA
GAGAACAAAAGCAGCAGTTGTCATCCAATCACATATCCTAGGATGGTTAGCCCGGCAAAGTGCTTCAAGAA
ATAAGGACCAATTACTTCAAGCAATATTAAAGTTGCAAAGGTGGTGGAGAGGTAAATTGCTTCATAAACAGA
GAACAAAAGCAGCAGTTGTCATCCAATCGCATGCCCAAGGATGGAAAGCCCGACAACGTGCTTCAAGAAA
GAAGTACCTGACACTGTTAGCAGTATTAAAGTTGCAAAGGTGGTGGAGAGGTAAATTACTTCATAAACGGA
GAACAAAATCAGCAGTTGTCATCCAATCGTATGTCCGAGGATGGATAGCCTGCCAAAGTGTTTCAAGAAACA
AGCACCGAATTGTTGTGATCCAAGTAAGTTATTGATCTCTCTAATAGCACCGTGCTTTTCTTAACATTCTTGAG
GCAACTTCTGAAGTTCAGATGTTATTAGCCATATAGAATGAAGCTCGGTGTTACATGATTAAGCTTGTACTGAT
TGTGCCTATACCGTAGGGGATCTGCTAGATCTTCTTGGATAATTTTCTTGTTTATGTTCTGAATATTTCCAAATT
TATGTGGAGTTGAAGCAGCAGCATCTGCCTTTCTTTTTGAATTTTCTGTTTTCATGCTTTATTTTTCGCTATGCA
GGCATACATGAAAGGATACCTTGCAAGAAAAGATTTAAGAGGGCAGCTTCTTGATTTGCGTCACAAAATACA
AAAATCTGCTGCAAATGTTGATGATGGTATGCGCATAATAAACAGACTTGTCGCAGCATTGTCAGAACTTCTG
AATATGAGAAGTGTCAGTGACATTCTTCATATCTGTGCAACTCTGAGTAAGTTTCTTGTGCATCTGGCACAAAT
ATTGCTGATCATCTCTTGTTGTCAATGTCCATTTACTTACCAATGTTGACGTTATATCTCATATTTCATTGCCCTT
AACTGAGTTGTGCGGTCTTGTCTGATGTATGTTGTCCTTTTATTTGTTGTGCAGCTCTTTTCTATAGAGACTGG
ACTTTAATTTAACAATAAATGTTAGAACAATGCATGTTTAGAAGATCATGAGTTTGCATTCAGTCAAGATTGTT
CACCTAGGGATCACAAAGCAATAATTTCCTCATTTTTCCTATGATAGATATGGCAACACAACATTCACAAAAGT
GTTGTGAAGAACTTGTTGCTGCGGGTGCTGTTGGTACATTGTTTAAGCTGATCCGCTCTCTCAGCCGGAGCA
TACCTGATCAAGAAGTTCTTAAACCTGCTCTCTCCACTCTCAGAAACCTTTCCCGATATCCACATTTGATTGAT
GTACTAATTGAAAGCTATGGATCACTGGAAACAATAGTGTCGGAGTTTTTAAGGTTTGCTATTGACATATTGA
ACATTAAAATAAAGTTTCCAATGCACTTCAGATGACGTAAGCTTTATATATTTCTGCTTTGCAAATGCTTGTTCA
GAAACAAAGAAGAGGGATATTTCATTGCTTCCGATCTTCTGAAGAAGATATTCACAGAGAAAAAAGGTGTT
GAAGCTGTGTGCAAGTCGCCTGCTCTTTTGAAGAGACTGCACAATCATGTGGAAGAGCTTTCAAGAAGAGC
AAAAGCTGACAAGAGGTCTGCTCTTTAGTATACCAATCCCAGTTGCTTAATTTTCTTTCTCCATACATTAGATT
ATAGTGAGCTAATTGGACATAATTTACGCAGGACTAAACCTCACGCAATGAAAGAGCCTGTGGACAAAAGAT
TGAGAGAAGCTGTTGAGATATTGGAATTGATTAAAGTGTCCATGGGAAATCCAAGTAAACGACTCTCTATGA
AGGTGTAAGAAATTTTCTTACTAGTTGGATAAAAGTTCTAAATCTTTCTTGATATTGCTATACATGCAATTTGTA

CATCATGTATAGCAAGAAAGACTAGAAACCTTTAATATCTGAATTGTGAAGAGAGCTTCACTTTATCTTTAACT
TCCCATTCTCTAACACCATATCATTGATCTGACTTTTAGATAGTCCATTAGGGACTCTATGTGATTGTCTGAGAG
CATCTGTTAGTTTTAGGATTACTGTATCTTGGCAGTTTAGTAGTACCTACTTTATTTTCA
>PGSC0003DMG400014709
GGTGAGACACGATACAAGACCATTTCTGGCCAAACGAATAGTGCTTTCGGTGGTGTTTTGTTCTCAGAAATC
TTCAGAACAATACCCCATAGCTACCTCTTTCATTACAAATATAAATTGGATAGAATACACAAAACATTTACACCA
TTCAATATAACCAATATGCTTTTGTCCTAATTTCTCAAAAAAAAGTACAAAAAAATTGGTGCTGTTCTTTGCCC
CCACCAAAATTGGATAGGATACAAAGCTCAATTGCTTACACACTACACCTTTTGCTATGAATTATAACTATACA
CAAGAAAATTATATTACATAAAGATATATTGGTAAAAAAAATGAACTAGGAAAAGATGAATGTACAAGAAATC
GAGGATGACTGTCGAGGATGGTACAATAATATTTCATGCAAACACGAGAAAATGTTGGTGTTCCTAGGCCTC
ACTCATGTAAAAATTGGAAGGCTGGATTCTCAAGAAGTTTGGTTGCAGGTTTGACATCTGCGAAATCCTTGA
CTTGAAGTGAATTTGAAACTTCTTCAATTGAGAATGGGATGCTGCAGTTGAAAAGATGCACTTGTTAGAATT
AGCTTCTTGAATCAACTCAGACATGTTAGCACGTTTACTTTAGGTCATGTTTTTAGGAGTCCTTTAGACTGGT
TAACGTTTATATGCCAGTAGAAATTATAGGGAACTTCTAGTACTTTTTGTTTTTGTTAATTTTTTGTATGCCCAC
AGAAGTAAACGCGAATAAGAGAAAATGATGAGAGATCTTCCATAAGAAGGAAAATAGGGGAACAGAAATA
AGAACATGGAGGAGTGAAAGAAAATAACAAACCCCGCTATAAGGGATGAAGGGTTAGGATACGGAGAATA
GATTCCGTTTCTCATTTTATATGGTGAAGTTTGATTATAGATGGAGTTTAAGAAAGAAAGACAGACTTCAGGC
ATATGCCAAAATTACCCTTACAACTTACGGTCTTAAGGATGTCACGACATTTCTCTGACTATAAAAGCATGTAA
TTAGGAGTACGAGTGTTGTGCTTGCTCTGACCGATAAGCCCATAAACTAACCTAGGTAAATCCATTAGATTTG
TTTTTCTGACTAACCATGAATTGTCCTAAATAAGATAATTCAATCAATCCTTTGTTTTGGGAAAACTGTTCAATC
CCTCTTTTCTCTGGAAAATCCGAAAGCAATGCTTTGTGGCCTTTCATCATAAAGCAAGAGGACGGGAAAGTT
TTGACGTTCAAAGTTAAAAATTTCCTAATATAGAAACATGCCATTTTTTCAAACAGAGTGTCATATAAAATGGA
GCAGAGAGAGTATAGCATGACTGGGACATGATTATGGAGATATGAATATGATAGGCATGTAGTAAGCGCATG
GGAAGGGATTATAAGGAAAGAGGACTCCACTTCATTGGCTTCATGCCGGGAATTAGAGGTACAATTTTTGG
TTGGATTTTTGGTGTGGAGATACAATCTTAAAGTAGGTGTGTCCACGGGTGTAAGCTTTACAGATGATATTAT
TGCAACCATGGCTATCTTAATCACAAGAAGCACCTGAGGTTTATCCTGGGAAATATGATCCAGAGAGTTATCA
TGGGTGGAAGTGGATCCGAAAATCCAGAGGATAGAAATATATCAAGATGGCTGGGTACAAGTATATTCACGG
TGAGATCTTATTGAGATTGCTTACTGAAGAGGGTAGACATGAAGACAGGCAGAAGAGAGTGTGAAAGACA
AAAATGCCGAATAAAGCTATTTCCATCAAATCATGTACCACATAGAATGCAACCCTTACAGCAAACAGTTAAA
GGAGGACAAATTGGATTGTATAAGTTGGTGTTCAATGTGCAAGGATCATATTCTTGTGTTATAGAGAGACTTC
ACAACCCTGGACAATGGAGTTATCCCGGAAATGGTGTTACGGGTGATGGCGAAGTATGGAGGGCTCCCATT
ATGATTTTCTCTGATAATCTTGAATGAAGGGAACTGGTGGATGTTTTTTTTGATAAGGAACTGGTGGATGTTT
TAAGGCAATACAAAAGAACTCTCTCAAAAAATGATGCCGTTCCCGTGGTGGATCCTCCAAGAATGCACTACT
TATGGAGGATTTGACACGCACGTGGTGACATTTTTGAAGAGTCCGAGCACAATAGGAAAAGAATGAGGAAT
ATGAGAAGTTCCTTTATGCTTCATTTATGCTTTTGTGTAATAAAGAGCACACTTACAAAACCCACATTGTACAT
AAAGAAAGCTTCCAGGTTATATTTCTAACGACATAATATAAGGCAAAATCATGATAGAAGCATCCAAGAAAAT
TGCTTGTGCCAAATTTGGCAGAGGATGACATAACTTTTCTAAACATTGAATAGTTTCTCACCTTGGGTTATCAT
CTAACAAAAAAGAGTTGCTCTTGGCATCGTTCGAGTCCTCTGTCATAAGTACCCTCATGTTAGATATGACCTG
TCAGGACAAAAGAAATTCCCTCAACATTCAACTATGACTGATATGAACCTGCACAGGTAGGACTTCAATCGG
AAAAATACCATCAGCCACGCATAACTTACATCTGGGGAGACACTTCGTGTATTGTATTTGTCATCCCAGTAAA
GAGTACATACTCTGTAAAGTTGCTGGACACTGAGAACCTGTAAAACCAGAATTTCACATTTTAAGGAAGGTG
ATATGGTTTATGCTCGATGAAATGGAAACCAGAAAAGATAAATTGATAAACCAAGTTCTGTCCTGGACTGGTA
GAGAAGAAAGTTTTGACAAAGAACTCTTCATATCTAAATTGCAAAATGGTGAAGATCTTAAATTTTAATACTT
CTACAAATGCATTTCACAACACTGGACTCAGAGCTAATGTTGTTGGAGAAAATTGACTTACAGGACACAAAT

CATTAGTGATATCATCATACGAAATTCTGTACTTCTGATGTATAACCTGCAAAAGAGTAACAATTAACAGAAAA
CTCCATATCAATTGTGGATCGCACTGCATATGTATAGTAATAGACATAAGCAAGTTCTAAAGAAAAAAGGAAA
AGCAACAATTATCTTGCAACAGCCAAGTCATAATAGCCATAATGCTTATATTGAAGCACAAGAAATTAGTAACT
AACCAAAAAACTCAGGGAAACTTCAGCAGTCCCGCTTACTACTAATTCATTTCCAGACACGATGTCTCAGAA
CTTTTTTCTTACACAATAAACCATATCTACTTGCTCAAACACTTTCATCTGAGATTAAAAGAAAACTTCCCAAA
AATAAAAAAGGATAAAGGAAATACAGATGGCACATTTAGATAGATTGACGGAACCAGAACAGCTTAGACTA
CCAGAATCAAATAATAGTGAGGCAACACGTTGAGGAAGTCAATATCCATGTAATATATGTTAAGAGATAAATG
AATATGTAAGGGTTCTTAAAATCTAGCATTTAATAACCTTGCAGCGCGGATATGCAAATTGGCCCATGTCCTAC
TCTTTCAGCATAGGTCAGCGTTGAAAACTTGGTTTGTGGTAAGTTATGTGGCTACTTCTAATCAATGATACTG
GAAGAAAAAATATTAAAAGAAAGATTGAAATATATTAACATTACCAAGAATCCAACAACTTGTCGTATATGTCT
GAGTTCATCCCAGGATGAGCCAGCATACTGCAAGCCAGGAATAAATGGAAAAAAAAAGGAATTAGATCCAA
AAAATTTCATTTAGGTGATAACAGTGAACCATAAATTACCTCTTCCTTTGCTTGGGAGCACCACAGCTCTAGC
TCAGCCAAGCCAGATTTAACATATTCTGCATTACTGAATGTACAACACTCCCGGCGAAGAAGAAAACTATGAT
TGCATAATTTAAATGAGATTAGAAAGCTTAAACTGACAAAGAACTAAAGACAACTTAGATAAAAAAAACCCG
GTCACCTGTTAAAGAGCTGTACATTCATGTATGAAAAGGCTTGACTAAATATCTTCTGAACAAGAATCGGAG
GCATCTGAGAATGGAAGGCAGCAAATATTAGCTTTCCTTATAACTAGAAAGATAGTGAAACAGGAAAAGGTC
TGTGAGGCCTTACAAAGTTTTCTTTCAAAGTACAGAGAAGAGAATCAAGGCATTCAATTATCCCTCGCCAGT
GATTTATTGAATAATCTTTGCCAAAGGATCGACCAGACTTTAGCACACTTCCTTTGGAGGTCCTTGGTGCCTG
AATTTTCAACATATCATATATTATTATGCGAAAAGCTGAAGGCTTTAATAGACAAAAGAATGTCCACCTTGCTC
CACAGTACAAAAAATTTAGAAGGAAAAGCAAAACACAGTGATAAATTGAAATATATCATAAGAAACAATTAA
AAACAGGCGAAAAAAAGATAGAAGTGAAGAAAATTTACAAAAAAAATTGAAATGCTATAAAACGATGTGCC
CGTCAATATTATCCCATTAAACAGTAGAAGGGAAACATTTTAAACACATAAAACAAATTCACCTGTATAAGAG
GAAAATTTTGTACTCTCCCCACTATTTTCATTTTAATATAACTTTCACAATTTTCAAAAATTCAAATTTATTAAAT
TATTCAGAATTTTACACTTCTGTGTGATGCTTTTTCCTGTCGAAATGTATGTGGACGAAGGGGAAAAATTAGA
ATAAAATGGAAACAAATATCAATATCAGTGATAACTAGTGGGGATTAAGGTTTAGTCATGTTATTATGCTTGTA
GACTAAACCACAAAATTGAAGAACTATTTCTTCCTTCGATGAGTGCATTTATTTGGCATCATGTAATAATTGTT
AAATTACCAGGGTTCTGCTTAGAGGAGAATATTGACAATCATGCAAAAAAGTTAATCAAGAAGTAAAATTAA
GTTGAAAAGATTTGTAAATTTCTATTTGTGCAATATCATCTTAGAGCCATCCTGAAGTTTTAGAAGCACAAAG
CAAGAAGAAACATGAAGAAATGCAGGAAGGCCTTTTTTATTGAAGACGGTGGATTGCAGTTTGTAAAATTC
AACATACCTGGATACATAAGGAAAGAAGTGACCCTAACTCCTTCTTCAAGTTATCACGAATAATTCCATACATT
TTCTCAACATATGCTGTAAGCTGCTGTTTGAAAAGCAGAGCTGGGTATTTTGCTTGAACCTGGTGCACTACTC
CAGCAAGATTGATGTCAGAAGAGGACGAGCGAAATCCCTGAAAACAATAACAGCAAAATCAGTTAGTACAT
GCATGGGAGCAAAACTACTATAGCTACGATTTTAGTACTCTAAATCAGTTTTAAAGAAGAACTAACTCAACAT
GAAAAGTTCTCATACCAATGTCATTCTCCCAAACAGGGATGTTGCAGGCTGTGGTTTACGGGTAGGAGTCGC
ACCAACTGCACTGTCTGGCTTCAGGCTTTTTTGGATTAACAATAACAATGTTGAGGTATTTGACAGCCAGTAT
GCCATGTGGTCATCACTATCTTGATTCTGCAATTAGAAGACTTCTCATCATATTCTAGTTTGTATTAAGTCTACT
GAAACATGAACCTCCAAATTTAAGAAATTAATCGCGTTTCTGTCTTCTTATCACCTTAGGAGTCATTTTTGAAG
ATTATAAGATTTCATCACAAAGCAGCAGAATGACACAGACAACATTCCTCATATATGCTTTAGTAACAATGCAG
GTTATAATGGTATTCACAGTTATTAACGTAAGGAGTTGAAATTCTTGCAGTCTTAATTATTTTAATGTAGGCAC
CATAGTTGTGTCAAATCTCACTTCACCTTCATTCTTCTTATGGGGGCTACACTTGCAGCGAGATATACTTTGTT
TTAGTTCTATTTATCCTAAACCCTTACTAAGTGATTCTCCATAATTCTAATTTCTGGTTGACTTTCTCGCTGTTTT
TGCCAGTTAACACTCCATCCTTAGCAATAAGCATGCACCATAGAATTATCCATCACCAAGTAAGTAACAGGCTC
AAGGTAGATCCTTGGTGTAAACCCATTTTTACAGAATCCTCCTCTATCACCAACTATTTAGACCGTGATGCTCC
TTTGTACATATCCTTCATATAGTTGGTATGAATTTCTTGCTTCTTGAGAACCCACTAAATGATTTTTCTTGACAT

GTTATCATATGATTTTCCATCACACCAATGAAATATCAATCACATCAAAAAGAGTATCGAACTGCTATAAATAAC
TAAAACTAATCCAAATGTTTTGGTGCAAATAATATGATAATGAGATTATAACACTGATAAATCTTTTTTCTTCGG
AATAAGTAATAACTCTGCTAGCCTTATTGATGAATATCATAGTGACACTCTCCCAACAATAATTTATATAAATGCT
ATGCGACAATCAAAGATGGAAGCAAGTCGATTATTTGATTTTTTATCAAATGCTGACAGCAGGTAGTTGAGT
GAAATCAGGAATAGCTAATGAAATTCTGTTGAAGCTTTGAGCTATTTTCCATCTTAGTTTCAAATTTCAATATA
ACTGGGTAAACACATTTTACTGCTTTGTTTCTATCACTGTTTCAAAGTCAATAGTTGCAATTCTCTATTTAGTTA
CCTCAATGGCAGAACCAATCATCTGCACTAAACGATCAAACACGCTAGTCTTTTCTGCTTCAAAAGATTTCCA
GTGAAGAAGACATTTGTAGATGGTAAAAGCTGCAACAGGCTTGCTTTGACTGAATCCAACATCCTTCATCAC
ACAGTCAATGAGTGCACCAATGTCTTCCTGCAGCATGGTTCACCAAGATATCAGTTTATAAAATGTTTCTCCAC
CAAACAATAAGAAATTTGACACAAAAGGTTCGCCAGGGAAGTACATACACGTTGTCGGTCAATAGGAGGTT
TCCTTGACTTGCTATTAGGGGTTTCAACTTTTTTAGCAGGTGTTGAACCTGGTGGATCCTGCAAAAGTCAATC
ATCTCATAAGCATGGAGCATGAATTGAAATTTGACAACTACCAAACATTAGTGTGGAAAAATCAAGCCATTTT
CTAGTAATCCACTCAACCCGCCCTAATTCAAACGTGTTGAAGTATGATACTTTTTTATTTGACCCAAGGATCGC
AACAGAATGACTCATCCAACTATTGAGTCAAAATGGATCAAAATAGTGTAACGCAACAGGTCAAAATGCAAC
ACAAACCCGAGCATACGAAATCAAGTTTGGAGATTGAGAATTAACAATTAAGTAAATTTGGAAGACTTGAGC
TAATAATTACTCCCTCCGTTTCACAAAGAATGACCTCCTTTCCTTTTTAGTCAGTTTCAAAAAGAATGACCTCT
TTCCTTTTTTGGTAACATTTTACTTTTTGCTTTCCACGTGGCATGTTTAAGACCACAAGATTAAGGGCAATTTT
GTACATTTGACATAACTTTAATTTAGGACCACAAGATTCAAAAGTCTTCTTTAATTTCTTAAACTCCGTGCCAA
GTCAAACCAGACCACTCTTTTTGAAATGGAGGGAGTATATAAGATTTTACACACTTTTTCTGAAAAGCCAAA
ATAAAAAGGAGAAAAGGTTGGGTCATGTTAGGCTGGATGGATTATGACCAATTATTTGATGCAATTTGGTTT
TGCTGCACTTGAGCTAAGGATCTTCTGGAAACACACTCTCTACCTCCATGAGGTAGGGGTAAGGTCTGCGTA
CACTCTACCCTCCCCAGCATTTGTGAGATTTCACAGGGTATGCTGCTGTTGTTGTTGTATTTGACCCAACCAA
ACTATTATGTTGGTTGTATAGTGCTCCGTTTATAGATTTGACCTACCCAAGTTTGATCAAACCTGCCTATTTGTC
ACCTGTACTTCACATGATGTTGCAGGATGACAAGAAAAGAGTATCTTACATTGGTTCTAGTTTCTTCTTTGAG
GTGGTAGCCATTTTCTTCTATCTGCACCTACATAATAGTGTCAATGTGATAGGCAGAATTTGTCAAAGGGAAA
GAGGGAAGGAAAGAAACTAATAAATAATTGAATTTTAAAGGCAAATACCTTGGAAGACAGACTAGGTGAAT
GATCTGAAACCTGCTTGGCAGGGGCTAACAGAGATTGCTGAAGAATCTGGTTCTCAGATTCCAAATCAAAAT
TCCTTTCCTGGAACCTATAACCAGAAAATGCAATGAGTAGAAATGAATTACATTGAATTATACTCACGAGCCA
ATCTGTATAAAAATAAAATTGCCATAAGATTATCCATTCAAGCTACTTATGAGTTATGACATAAGCTCATTAAAC
CTTTGCATAGTAGTCTTCAACTGAACAATTATAGACTCTGCATCCAACACCTGCCTCAACCTCTCCTCACTAAG
TTTGCTTGTCTCTTCGTACTTTTTTTCTGTCTCATCAATCTTCTGTTCTAGAGAGCTTACCAAAGACTGAAATCA
AAAACAAAAAGGAGCATCAAGAGCTGAGTGCAAGTAGCTCCTGTGAATACAAGAAAGTCGTTTACACTTGA
AACTCCCTTGTTTCAACAAACCTACCTTTAGTTTCTCATTTTCAACACTAAGTTTGTTCATCATTTCATGATCAA
CAACAGGAACTTCCTGCATAATAGGAACTTCCTCTGCCGCCCTTTTTGTAGTTTCACGTTCTTTGACAAACAT
TTCTTGTGTTTCCTTGAACTGTAGTTGAACTTCTTGCAAAGCTGATTGCAGTTTTGCATTTTCTTGTGTTTTTG
CTTCTTCCATGTCAGCCTACAAAAACAAAATTATATTGTTTAAACTGGAAGGCAGACACTTAAAAGCATACAG
TGGTAGAGACGGAATTAGCTTATTCTTGTACCATTTTCTTATATTCCTATTCCACTTTGACAGAAGCAAATACTT
CTAGTCAACTAGGCCCATCAATAAATTGTGGTCCATTTTACTTCATGCTACCAGTTCAACTAAATAGGGAGTCT
ATATGTGATTCCAATCAGCAGTGGTTTTGTTTGTGTTTCCTTCGTCAAATATGATTATAGAATTGCATGTATGAT
GTACCTATTCCTGCTCCGAAGCTTTTCTTTGAAAAACATGACTCTAGTTAAAAAGTTTGTTTTTTCCGTCCTCA
GCTTAACAGATCAACATGGACTCATATGAGGATTTACAGTATTAGATTCATGCTCAAACACTTAAAAGCTTTCA
CCAAGTCCATCATACAGCATTATTTCAAGATTGAGTTCCTCTAGTGCATTGTGTGTTCTAGATGATAAATCCTTT
ATGGTTTAAGAATAGGGCACAGCAGCATCTTAAGATAAAGAATTTACCCTCATGCGTTTCTCCAGTTGTAGTC
TCCAAGTTAATTCTTCAACTTGCTTCTCTAATTTATTCTTTGCAGCTTGAAGCGCACCAGTCTCCCTTGCAGCC

TATGCTTAAAATAAGTTAGAATGGAACTCCGAACAAAAATGGAACTGTTTGAGAACAAACAATAGATATTCA
ATCAAAAGTATAGTAATAAAGAAAGGGTGACTTTTGCAAGCAGGCCAACTATCACGTTGTTTGCTTAAACTG
AGCATGGTGCATGAAATGCATAAACTTGACTTTCTAACCTTTAGTATCGATAAATTTAAAACTGGGGATTTCCC
TAGGAAGATATAAACTGTTGAATGTAACATGAGCTGGCACTAAAATGTGAGATAAATCAAATCTGGATGAATA
TACAAAATATCATTTGAAATTATCTGTCTTGCACAACAATTCATGGATCTAAAAAGCAAAGTCGACTTGGCCG
TAGGGGAATTTGACAAAATGCAACTCTAGCAGCATGGCATTATGACTTGTGTACTCAGAGACTCTCACTGGC
TGCAAAAAACGAGTCCCTAAGTTTTACTAAGAGACACATTTCCAGTTGTGTACTATAGCTTGCAAGTTCTTGA
AGGCAGTTACTTAAATGCTTATAAGAAGTTAATGCTGATACCCTTCAAAGTATTCACTCCAAGTATGAATGGCC
TAAAGCATGAAAGAGAATTTAGAGGTCTAAATTTGATGTAAGCATGAGCAGTGAGAAGCATAACTGCAGTTA
GTTTCTACAGGATTTAAGCATACAGTAATCTAATACATTTCCGAGAAATTTAAACTGTGTATCAACTCAGAATA
CAGATGCTAACACAGGCTGTAGCAGCTAAGAACCTCTGGATCAATAAAGAGGATAAAGAGGAGATGAAGAA
TGAGATATCACCATCTTAAGTTTCCGTAGTTCCCCACGAGCGACTCTAGCTCTCCAAGCACATTGAGTGGTAA
TAGCAGCTTTCTTGAGTTTCTTGTATTTCAACCGGGCCAAGAATGCACGAGAATGACTCTGTACATATAGCAT
GAAGATCATTATAAAGTGTGAATCACAGCTTCCGAGGAATTAAGTTAATAAACAGGTAGAGCATAACTATTCC
AAATAGTCTGTTGATCCTAAATTAGAAAAGTTTCTCAAGATTTCAAAGAATTATTCTTCCATTACAAAAAGTTT
ACCTGAATGATAATGGCAGCTTTTGTCTGCCTCCTGAATCGTACTTCATTGCGTGCAGCCATCCCACGCATTCC
TGTCTGAATTGAAATGGCAGCAGAGCACAATTCTTTGTAACCCTTCCTAGCAAGATGCATGCGCATGTCTGTC
TGGATCTTCAGACAAGCTGCTTCTCTCCGCAAGCTCTCATATACACGCCGAGCAAGTTCTCCTGTAACAATGA
GAAATGAAAACATTAATAGATTTTAATCTACGAGCCCAAAATCCAAGCGTATAGCAAATGAAATACCTCTGCA
CATCGATTGTATCCTTCTTGCCAACTGACGTAATAATGTAAAATTTCTTCGAGCCATGTGAGAACGAACTTTCC
TCTGAATGATGCTCGCCGATCTCCCTAACACCTCTGTCCTGCGACTGTCTAGCTCTGCCATCTGCCCAGCCCTT
AGAAACACTTTTGTTTTACCAATCTGCAAAGGCAAAATGGGCAAATGATTGGCTTTCCTGAAGCTTCCTCCA
CATGGAAGACATCTCACAAACGAAATCCAGAGGGATTCTGATTATTTATTTATTTTGCCTTTTTAGATCATTAA
AAGAATGAAATATTCTACAGTTGACCTAAGTAAAAAGACCAGGAAAGTATGAAATATATTCCAGACATTAAGG
ATGATGCTTGTGGAATTTAATGCCAACATCTTCTGCATATGATTATAAAACAAGAACCAAAAGAACTCTGGGA
AGAAGATGAAAAAAATACTATACTTATGAAAGGGATACACACTATCATGATCAGTAAAGACACAAAACTAGA
AAATTTCAAAAACTAAAAAAATTCACAGTGCATCCATGAAATTACAAGAAAAAACAAGAAAGATGAGCATAT
AATACAACTGAACAATATAACCAATAATACCTAGAATATAGAGATGCTAAGAAGGAAAGGAAGTACACGTAG
CAACGAGAGGAGGCCACCAGAAAGACGAAAGAATTTACAGATTAATTATTCACCAAATATCTGACCTGCCAA
AAGCCCGATAGGATTCAGGTGTGCAACAAATGCAAAATGGAACCACTGAAATTCTTTAAACATTTTACTGAA
TGAAGAATGTGCTAAAATATGCTAGATAAATATCAGTTAAAAGAGATAAAAACAGACTTTCTGTCAAGTGAG
GTTATCATCTGAAATTGTTAAGAGACTCCAAAATTACAGAAATGAACTAGATAAGTTTGAATTGTTTCATGTTT
AAACTATAACCTCTAATGAGGTAATGTATTACCTGATATCCTTGAAGGCCGACTTTCTCTAAAAGGCGTGTGCA
TGCAGTGACCTCGTCCGTACTGTCATAAGTTAGTCAGAAAAAATCACAACACATTGACAATCTGATCAAATAG
TACAAGGTAAATAGGTACCTTCCATCTAAAACTTCAGGCGAAAGGATGCCAAAGCGATCTAAAAATTCATAA
AAGGGTTTCCGAGTAGGATATCCAGCACAGCTTATTCTTATTGCTTCCATCACTCCCTACAGAAGAAAAAACA
GTGAGCACAAACTTTCCATTCATATATATGCCACCAATTTGAAAAAAATTCAATACATTTCCTTACCCCACAGC
ACAGCTGTTGCAGAACATTGTGATTCTCGAAGATAGAGGGCTTTAGCAGATTATTTGGCTTCACACATCGAA
TATAATGGGGTTCAGTTGCATTCAGAGTTTCAAGCAAAGACTGCAGTTGTTG
>PGSC0003DMG400022027
CAAAAACTACGTGGGGCTTTGAGTTGAACTTGAGATATAGAAGAAGAAGAAGAAGAAAAGAAAAAGACA
CGATTCTTTTGCTCCCTCTCCGATTTTTCTGTAATTTTTCAAGAGAAATTGGATTGGTGATTTAGTATAGGATA
AAGGGGTTTTTTTTCAGTTCATTTGGTTACCAGATCTTGCTTGTTTCTTAGTTTGAAAAGTTGAAAAAAATTG
GAGTCAAGTTGAAGTCAGAGAGCAATTGAAGAAAAGTGAAAGTGAACTGTTGCTTTATATTTGTCATTTTTG

AGGAGATTGGGTTGTGGGAAATCAGTGGTTTCTTGATCTGTTGTCAGTTTATTGTTATTGATGATTCAGGAGC
TGATTTGATCTGATTGGAGTTGTTTATTTAATGGTACTATTGGATTATTTCTTACAGCATTGTTTTTGATCTGCAT
TTCTTTATGTATTTTGTTTGATCTGTTTGAAATTTGATTTAAAGGGTATTCCTCATTGTTATCAGATTGTGTAATG
GACTTTTGACCAAGGAGGATACTTTATGACATTGGGTTGATTTGGGCATGGTGATTAAAACTTGTTTATGGC
AATTTTATATTTGCTAACTAAGTGCTGTGAAAGAGAAAAAAATATAGATTGCTTTGAGTCTATGGATGGATGTT
GAAGGGACTATGTTGTTTTCTTTTTGGTTGAATAGTTGATTTCAGGTCAAGTTGGTCATGTTGACTAGACCCT
ATTTCGGCCTGGTCTGGTATGGAATGAAATTATATTCATGGAATCGACTATACCTTTCCTATTGATGGCTAAATG
CTGTCAAAGTTTCATCTTTGGTTAGGAAATGTTAGATGTCTATGAGTTTATTAATTCTGAAGGTCTATTTCTGTA
GTTTATTGACTCCTTGTTTTTAGCAGAGTGTGGGTGGGGTTACGGGATATGCTCTGTTTCCTGCATGAGCATT
ATCATAAGCATCTTAAAGAAATTAATGTATAATTAATTTCTCCCACTGTCATGCTTTTTTAAGTAAAGCTGGTAA
ACTAAATTAACTTCCGCGGAGCATTCTCATGTATTTTCTGCAACTGTTTAGTGATTGCCCAGAGTGAGCAGTA
AACAGGGCGGCGGCAGTGTAATAAAATGATAAACACATCATGACCTTTTATGTTGCATAAGTGTTTTTTTTCT
AATTCCATCAGCCAAGACTTGCTGCATTAACAATGCTTTTGATAACATTCAATGTAATGTGGCACAATTTCTCT
GGTTTCGAAGGCATTGTTTTCTTCTAATTGCTTGGCCTCTTAATGGCTAGACATGCTTTGGTCCGTAATACAG
ACTGGCAGCCTCAATCTGAGTCCTATTCATTTTGCTTATACATTTAAGGGCACAAAAGAGTACTAACTATTGGT
ATTGTCTCTAATAAATATTTCTGAATGGGCAACCCGCATCGTTGTATTTGCAGGCTGCTTCCATTAGCTTACCG
GTTGGTTCTCTTGTTTGGGTGGAGGATCCTGATGTAGCCTGGATAGATGGGGAAGTTTTGGAGGTTAATGG
TTCAGACATAAAGGTTCTTTGCACTTCTGGTAAAACGGTAAGTGAGCACATGGGATTTTATTGTAGGAAAGA
AGTAATTAGTTTGTTTATTCAACTAACGGGAGAAACAAGCCAGGGCATTGAGTTCTATCCACCCTACTCTCTC
AAACACATTGGTAACTAAGCAGAGTTTTGGGAAATGCTGAACCTTGTTCATTTACTTGCTGAAATCTGTGGTT
ACAGGTTGCTGTTAAGTCTTCTAATGTCTACGCCAAAGATGCTGAAGCTCCGCCGTCTGGTGTGGATGATATG
ACGAAGCTGGCTTATTTGCATGAACCAGGAGTCCTACATAATTTAAAGGCTAGATATGATATCAATGAAATATA
TGTGAGTTTCTTTGTCATTTTATAAGATCTGTTCATTTTCCTTTTAGGTCAAGTAACCTAATAAATGATGATTTC
AGACATATACAGGGAACATATTAATCGCTGTCAATCCTTTTAGAAGGCTACCACACCTATATGATACCCATATG
ATGGCCCAATATAAAGGTGCAGCTTTTGGGGAGCTGAGTCCACACCCTTATGCTGTTGCAGATGCAGCATAC
AGGTTTGTGCTTCTCTGTTTTTATGAATTAAAAACTCTTGTTTGAATTGTCATTGAATATATTTACAATGGGTGC
AGACTTATGATCAATGATGGAGTAAGTCAGTCAATATTGGTTAGTGGGGAGAGTGGGGCTGGTAAAACAGA
AAGCACCAAGCAACTCATGCGCTATCTTGCTTACATGGGAGGGAGAGCTGCAGCTGAAGGTAGTAGATCAG
TTGAGCAGCAAGTCCTGGAGGTATTAAGTCTTAAACTGTTGTACGGTATTGTACCTAGAGCGTTACAACTGGT
TGCTGATAATCGTTTGTATGGATGTAAAACTAAAGATCATCTGTTGCCGTTGTACCTTTATCTACTCTTACGCGG
TGACTACGCTTTTGCACTTTGCAGTCTAATCCTGTTCTGGAGGCATTTGGTAACGCAAAAACTGTCAGAAAC
AATAACTCAAGGTTGGATAGAATTTTGTCCATTTTCTAATTGTTCAGAGGGCATTTTTTTTCTGGAAAAAATT
GCTACATGGTTGTAATATCTTTTGCATGGAGATTGCAGTCGTTTTGGTAAGTTTGTGGAGATTCAGTTTGACC
AAAAGGGAAGGATTTCAGGAGCTGCTGTCAGAACATATTTACTCGAAAGATCGCGTGTTTGCCAGTTGTCT
GATCCTGAGAGAAATTATCATTGTTTCTACATGCTTTGTGCTGCACCACCGGAGGTAATGCACCACTATTTACT
TCATTAGATGGATTTCTATGAAGAAGAAGCACTCTGAACCCACAGTATTTTATATTGTTTCCCCCCTTGAGTAT
TTGGTTCACTCCTCTTCTGTGTTGGAAAATTTAATCAACTTTTTCCATTGGTTTTGTATTCTCGCAATGGTAATC
TTTTAGAGGTTGCAACTTGCAAATGCCTTTCTATTGGTAGCATTTTATCTTGTCCTCTCCTCTGACAGCTTATAA
TGTTGTTGGCTGATTTTAATACTTTGTGATTAGCTTCAGAAGTTAAATAATAGTCCAGATGCAATCTGCTTAGG
TTAAAGGATCTTCATGCCCTCAAGTCATCTGTGTTCTTGCTAGCTCACATGCGACTATCAGTATTCTGATGGTG
GCAGTGCCTAGAGATTATTGCATTGGGACGATAGTTTTTGTCCAGTATGTGTAATCCAATTTAACACCAAATCT
TCTTTAGAGTGTAAATATCCCATGGATGAAGGGGTAAAAGATATGTAATCTGCACGGCTAATGATTTTTTTGAT
ATGTATCCCAACAACTTCCATATTCGTTATTTTTTACCCTTCTGAGAATGCTTTTCCCTCAGTAGTAGGAAAGTA
GTACCATACTTTTGTTTGGATAGCTATAGGGAAACCATCTTTGCATTCCTACTATTCATTTGGAAAAAAAGTTG

ATACGATAGTCCGATTTAGTTATGCTGTGTGGGAGGTAGCAGGTAACCAATGGAATAGTCGAGGTGCACATA
GGCTGGTCCAGGACACCAATGCTATAAAAAAAAAAGAGAACAATACTTTGCTGCAGTAGCATATATTGGTTC
AGGGATCATCCTATAACAACCTGTACACCTACCATGATTGTCCTTCAGGAGGTGCTGCATTGTCTTTGCCTGG
GATGCTCAACTCCATTTCCTTCTCTTTGATAACTTAGTTCATATTGAGGAATAATCTGTCATATGGACTTGTTTT
CCAAGTTTCTTATTTTATCTTTCCGCTCTCAGGACATTCAAAGGTTCAAATTGGACAATCCTAGGACATTTCAC
TATCTCAATCAGACGAATTGCTATGAGTTAGATGGGCTTGATGATGCCAAAGAATACTTAGCTACAAGAAGG
GCAATGGATGTTGTTGGAATAAGTTCTGAGGAGCAGGTATCTTTTGGGAAATTACTTGTGACAGTTAAACAT
GTTCAACTTTAGATTCTCCTTATACTTTCTTGGTTACTTTGTGAATGCTCCCAGGATGCAATATTTCGAGTAGT
GGCTGCAATTCTCCATCTTGGAAACATAGAATTTGCAAAGGGGAAGGAGATAGACTCCTCAGTGCCCAAAG
ATGAGAAGTCTTGGTTTCATCTGAGAACTGCTGCAGAGCTATTCATGTAAGTGGTATATAAGGAATATTGAAT
CAAAGCACTTTTCTTTTTTACTGGTTTGAAGCATTTCGTTTAGAGAGAGAGATAAAGAGACAGAGTAGCTAT
TAAATGTTTATATTACTCATAGGTGTGACGTAAAGTCTCTAGAGGATTCCCTTTGCAAACGCGTTATTGTTACT
CGTGATGAAACCATCACCAAATGGCTGGATCCAGAAGCTGCACTTACCAGTAGAGATGCTCTTGCAAAAATT
GTGTACTCAAGATTGTTTGACTGGTAAGCTTTATTTTTGTGTCTAGACAGGAATTAAATGACATGTTCAGCTAT
CGTCTTTGTTTGTAGTATTCTTTATTTCTACAAACAATTGTCAGTTTACTCAGACCCTCCAACAATTTTTTTGCA
GGCTGGTAGATACGATTAATAGTTCAATTGGTCAAGATCCAAATTCTAAATCTTTGATTGGTGTGCTGGATATC
TATGGATTTGAGAGTTTCAAGACTAATAGGTGCTTATTTGTTGCGTTTTGGTTGGATTGTACGTGAATTTTCTC
TTGGAGTGAACTATTAAAGAATTCCTTTGACATCTCCACAAACAAGCAAATGTCGTTGTACTTCACATTGTGT
CAAGGGTCACATTCGTGTTATTGAAAATCGGTTTGTATTTATCTGTACACTTTCACCAATCAAATTTATTGTGG
ACTTGAGATCTCATTGTTAGTTACTACCTCTGTTCCAATTTATGTGACACTCTTTCTCTTGTTAGTTTGTCCCAA
AAAGAATGTCACCTTTCTATAATTAGAAACAACTTAACTTTAAAATTCTTCTTTTACCCTTAATGAAATATTTATA
ACTACACAAATGTTTAAAATTTGTTTTAGACCATAAATTTCCCAAGTCTTCCTTTCTTTCTACATCTGATATTTAT
GGAAGCTTAAAGAAGCACAATTTTGAATTTTGTTACATTTTGGTAACTCCGAGTGTCTGAATTTTTTCGCATC
TCAAAGCTGATTATTTTGATTAGTGTATCATTTTTGTGGGTTATATCTGCAGCTTTGAACAATTCTGTATCAATT
TAACAAATGAGAAGCTTCAGCAGCACTTCAATCAGGTTCTGGCTATCTCCTTATCTTTTTTGTATTAACTTTTA
GTCTGCTGCTCAGCTTTATGACACCCTACCAATGTTTTTACAGCATGTTTTCAAAATGGAACAAGAAGAGTAT
ACAAAAGAAGAAATTAACTGGAGCTACATTGAGTTCATTGATAATCAAGATATTCTTGATCTGGTAGAAAAG
GTTTGTGCTGAGATTTTGGATGCTTAAAGTCTTGCAATTGCTTACTTGCAAATGTCTTAGAATATATTTGTTGG
GTAAGTCAGTAAAGGAAAAGTTGTTGCTTCTCCACTCTGATGCGATGACCCCCAGTAAACCCATTTTACTGCT
TTCCTCCCTTCCCTCTTTGTAAAATCTCTACATGTCTTCTTGGCAGTCACTTGCCTTTTGTTAGGAATATGTTAT
TCTCTTTGCAAGCGTTTGTCCGATTTATTGATGCAGCTCAGTTTTTCAACTTCATGTTACTAACATGTTTTAATC
ATGGAAGAACCTGTAGGGTCACGGATCATTATACTGCAGGTTTAGGGTTAAATAGCTTCAGAGATGATTTTTC
CATTGATAAACAGTAATAACAGGTAGCAAAAAGCTTATGCAGAGATATTGTCAGCAAGAATAAAAAATTCAC
AGAGATGTTCTAGTAGTAGAGGACCACAAATCATGTTGTCTAGGTCAGATAAAATCTAATGAGCTAATTGGCT
AATAGCTTCTATTTTCTTCCTCATCAAAGATCTTCTAAATAATAGATCATGGATAATCTCGTTTGCCCTCCATTTT
CTCATTCAGTTGATTTTTTTTCTTGTTACCATTTGCACCAGCACTTAGTCACTTGCTCTTTCCTTATTTTAATGA
CATCTTAATTTTTCCCTTTGCTCAGAAACCAGGCGGTATTATAGCACTTCTTGATGAAGCTTGGTAAGTAGCTT
GATGAGGTTCTTCCAGTAACCTTATCATCCACTCTCTTTTATAATTGACACACTTATTTGTGCAGCTTATTATCAT
ATTGGTTGCTTAGGATGCTTACAATGCTTTTGTTTTGTTTTTGTAGCATGTTTCCTAGATCTACTCATGAAACG
TTTGCTCAAAAGCTCTATCAAACTTTCAAAAACCATAAACGTTTCTGCAAGCCCAAGTTGGCTCGTTCTGATT
TCACTATATGCCATTATGCTGGTGATGTAAGTTTTTTATTTCTTCTTATCTATACACCATTTGTAATATCATGTTTA
GAATTTGTAGTTAACGTCAAAACGTGGATATTATATACCTGTTATTTTCTCCAGGTCACGTATCAAACCGAATT
ATTTCTGGAGAAAAACAAGGATTATGTTATTGCTGAGCACCAGGCACTCCTGAATGCTTCAACGTGTTCCTTT
GTATCTGGGCTGTTTCCAACATCAAATGAGGAATCCTCAAAACAATCAAAGTTCTCATCAATTGGCTCAAGGT

TTAAGGCACGTAAAATGCACCTTTTTCTCTCATATTTGCTTTTCCCTCTTATCTGTGCTATGGCTGGGAGCATG
TTGAAACATGATTTGTGTCATATTTTTGCAGCAACAACTGCAATCTTTGCTTGAAACACTGAATGCAACAGAA
CCCCACTACATTCGATGTGTCAAGCCTAATAATCTGCTAAAGCCATCTATCTTTGAGAATCACAACGTTCTGCA
GCAGCTGCGTTGTGGAGTAAGTAAACGTTTTGAATTTTATTACTGGTAATGTACATGGGAGTTGGATTTATGC
TCACTCTTATTCTAATTCTAGGGAGTGATGGAAGCGATTAGAATAAGTATGGCTGGATATCCTACTCGGAGAC
CATTCTATGAATTTCTAGATCGTTTTGGCATCCTTTCTCCTGAAGTTTTAGATGGAAGGTATGCTATTACCTTGT
ATAATTCAAATTATTAATTGATTATGTTTTTATGTTTTGACTAAATTCATGACAGTACGGACGAGGTTGCTGCAT
GCAAAAGGCTCTTGGAGAAAGTTGGACTTCAAAGCTACCAGGTAATTTTTTGAAATTTCTCTATTGGGATAT
GAAACACTTTCAAACTTTTGCATGTACTTTTCTATCCTTTGTATTATTCTAAAATATCATAAAAAATCAATTTAAT
GACAATATTTGTTAGCTTTCTGAACATAGCCTTTTACTTGAACACATGGAAACATTTCTTATTCATTAAGATATA
TCAATCAAGTTTTGTCCTATTTTGTATTTTTTATGCTTAATTGTTCAGATTTTCGACATGTTTAGAAGAGAGTAG
AATACAAATGTTTGGAGGATCACAAATCTGTTGACTGCATTTACCTTTGGCTTGTCCCCCATCTCGTCTATGTC
ATTATCAAATTCTTTCTTTCACATGAGCATTACTAGTTGGGTAATAAGACAAATAGTTTCATATTGGTATTCTTT
CTTGTCTCTTTTGTTGGTCGACTCCTCATGGATGTGCTTTAAACTTCTTTAGGTTTGCAAAATTCTTGTTTGAC
CTAATGATTGATAAGTAAATAAGCAAATAAATACAGCAACAACAACAACATACCCAGTGAAATCCCACAAGTG
AGGTTTGGGAAGGGTAGAATGTATGTAGACCTTACCACCACCTCGTGGAGGTAGATAGATTGTTTATGAAAG
ACCCTCGGCTCAAATGCAGCAATTCCAGATAGAAAGAAGAAAATAGCAAGTCACAAGGAAACAATGCAAAG
TCTACCAGAAGGGAGCAACACCAACAACAAAATAGTGCAATAATCGAAACACAATAAACAACATTAATACAA
CTAGTACATTGACAAACAATAAGCAAATAAATATTGACAACAAAATCTTTGTTTAACCTCTTTCTAGATTTGTT
GATCTCAAATTGGCTCCTTGCTTTAGCCATTTTCATCTTCTTGCCCATTAAGTGCTAAAATGCACTTTCTCATGT
TTTCTTATCCTAGTTTATAGTTTATACTGTTTTCTGCTCATGATGCTGCAAACTTCAGTGGCAAGTCTAATATGC
TGCATTGATTGTCTTCACAGAGTTATTATTGTTCTTGTTTATAAGCATACTTTGATATGGTCTCCATATCAAATTT
CTATCCTAGTTTCATATGTGAGACTTCTCCCATGTTTAGGCTTCGAAAGCCATTCATTGGTCTTTATTTGGTCTT
GCAGATTGGTAAAACAAAGGTGTTTCTGAGAGCTGGTCAAATGGCGGAACTGGATGCTCGAAGAACAGAG
GTGTTAGGGAGATCTGCTAGCATCATCCAGAGAAAAGTTCGTTCTTACATGGCTCGGAGAAGTTTTACAGTG
TTACGTCGGTCAACAATACAGATACAATCTCTGTGCCGAGGTAATGAATTTGCTGCATACCTTGGTTAGTATTT
TAGGATGCACTTTAGGAATCTTGATACTTATTTGATTGTTTCCATTTGGTCATTGTAGGGGAACTTGCTCGGC
GTGTATATGAGAGCTTGCGGAGGGAAGCAGCTTCTCTTAGAATCCAGACAAATGTGCGCATGCATATTGCTA
GGAAGGCTTACGAAGAGTTGCGGTCCTCTGCTGTTTCAATTCAGACAGGATTGCGTGGGATGGCTGCGCGC
AATGAGCTTCGATTCAGAAGCCAGACAAAAGCGGCAATTATTATTCAGGTATGTATTCAATATCTGAAGGATA
AATCTGTCTTTTCTTTTTCTTGTTCCTGGAGAATAGTAAGCCTAAGGATCTTTATAACTGGGGTTCAATTTCAT
GACCTTCAGGTTTATGGTTGAACCAACCCTTGGGGTTAAATGAAGGATAATCATTTCAAAAATTTGAGATAGT
TTCCTACTTTAGTAGATGTAGAGCCTGTTTAGATGGACTTATTTCTTATTGCTTATAAGCATAAGCCATAAGTTA
GAAATTCTAACTAATGGACTTGTGAATTATTTTTATTATCTTACCCAAACTCTACAAAAGTGCTTAAACGTTTTT
GTCTTAAAAACAGCTAAAATAAGCCATTCCAAACAGGCTTGTAGTCAATTTGCAATAGTTTTCTTCGTAGCAA
CTGAATAATGTATCTCAAAATCTACTGCTGAAATTTCATGGACCCTCGTACTATATGTTCAGAGCCATTGTCGC
AAGTTCTTGGCATATTCAAAATTCAAGAAGCTCAAGAAGGCTGCAATTACCACTCAATGTGCTTGGAGAGCT
AAAGTTGCTCGTAAGGAACTGAAGAAGCTTAAGATGGTAATTCTCATTCCACATCTCTCTTTATCATCTTTGTT
GGACAAAATTTTTGCTCTCTGAGACTATCCTATTTTGGTGATATACGCTGCCTGAAATTTTTCAGATATATAATA
TTGTTGTAGGCTTGAACCGTGTAAAAACTAATTTCGAAAGGTTCTTGTGCTGATATTAGATTCTTTAATTCGAA
AGTTGCTTTCATGATTTTGACCAAGGAAGTGAAAGTGACTGCTTTTAAGGGAATTAGTGTTCTACTTTAAAA
ATAAGTATTTAGAAGACCCGAAGGGGGGGGGGGGGTGNNNNNNNNNNNNNNNNNNNNNNNNNNNNN
NNNNNNNNNNNNNNNNNNNNNNNNNNNNNNNNNNNNNNNNNNNNNNNNNNNNNNNNNNNNNN
NNNNNNNNNNNNNNNNNNNNNNNNNNNNNNNNNNNNNNNNNNNNNNNNNNNNNNNNNNNNNNNN

NNNNNNNNNNNNNNNNNNNNNNNNNNNNNNNNNNNNNNNNNNNNNNNNNNNNNNNNNNNNN
NNNNNNNNNNNNNNNNNNNNNNNNNNNNNNNNNNNNNNNNNNNNNNNNNNNNNNNNNNNNN
NNNNNNNNNNNNNNNNNNNNNNNNNNNNNNNNNNNNNNNNNNNNNNNNNNNNNNNNNNNNN
NNNNNNNNNNNNNNNNNNNNNNNNNNNNNNNNNNNNNNNNNNNNNNNNNNNNNNNNNNNNN
NNNNNNNNNNNNNNNNNNNNGGGGGGGGGGGGGGGGGTGTCTACGGATTGGATAGTTGCAGTGAC
AACCCTTGGGAACTGAGTTCAAATTCAATAAAGGTGACACTTGGTGAATTCTTCCTATATGCCTAAGTCTTGG
TAGACGGAGTTGTTGGTACTTATGCTGGTGGGAGGTAGCAGCCTAGCAGGTACTCAGTGGATATGTCGAGAT
TTATGCAAGCTACCTGATACCACCACTATAAAAAAGTTCTTTAAGTTGGCTCATCTACCTCCATGAACTTGCAA
GTCATAGTACTGAATTAAAGACTACAAATAAGGCTAGTACTAAGAATTAAGGCCTTAGTTTAGCAATCACTTG
GAGTCTTGGACTGCAAAAGTGATTATGTGCAGCTACTCAAGTTGCATTTTAGACAAATTTTTCCCTACTGCTG
AGTTGACTGGCTTTTCTAAGTTCCTAAAATTGTTAAGCTCAATGAAAGAATAACTACATCATTATAATCATTATT
TTTCTATTTTATACATCTGACCGAATTGATTTTATTCGATGTTTAACACATAAATAAATAAGAGGTACTCTGTCTT
ATTTCAAAAAAAAAAGTGTACTCTGTCTAACAACTTAAGCTTTTAGATGTGATCGTCACACACTTCAACATAG
TATTAGAGCAGGCAAAGGTCTTAGGTTCAAGTCTCACCGCCACCCATTATCAAAAACAATTTCCACATGCTTG
GCTCATGAAAAAAATCAGGCTCGCACGTGAGGGGGCGTGTTGAAGTTATGAATAAATAAATAAAAGCCGAC
TCTCTAGTTTGTTCCATGCATTGTGCACTTCAACTGAAAAAAGCTAAACATGCGAAAATGCTTGCAAATATGA
GTCTTGTTCTATCGCTACATTCTATTTATCAGGGTTTGGTCTCATCAACTGCTTCTTATCTGAGTTCTACTTCGA
CTCATCCTTTTTTCAGGCTGCACGGGAGACTGGGGCTCTGCAAGCTGCAAAGAATAAATTAGAGAAGCAAG
TTGAAGAATTAACTTGGAGATTGCAGCTGGAGAAACGCATGAGGGTCAGTTAGATAGAATTATATGCATAAT
TGTTCACCCACATTTTGGTAGCTGTTCTTTCATGGTGCACAATATCCTTTTCTCTAAAGCAAGTTGTAGAACCA
TCTTGTTCAATCTTTATCTGTGCCATTGTTGATCCGTTTAAAAAACGGGGAAGGAAACACCAAAAGTTGACTT
AAAAAATAAGGAATACCAACAATTGCCTGTTAATCTAGGTTAAGGATTTTGAAGGCAATTTTAGAACTAGAA
CAGATACATGGAGCACTATAATTTTATTTGGTGAAGGGAACAATAACGCACCAGAATAGAGGCACTCGGTCT
AAGTAATGGACTGCTTCAGCGCCTTGAACGATTGGATCACTTTCTTGCATTCTTTGTTTCATATCATTTGGAAA
TAGTAGTCTGTGGGTGATTAGATTGCATCTAGCATCCCACTGGTTGTGAAAGATACTTAGCTTGAAAAGAACA
CATGTTTTCTACAAATGGGCCAAAACTTCAACAACTTTTTGTGCTTGCTGAATATAAGTATGAGATAATTCTAT
ACCTTACCTTATAAAAGAAGTATGAGATAATGCTAGTGAGAATATAAGCTTGTCCCATCTCAAGTGTTATTTCC
TTACGTGATCATTTTTTTCTTCCTTCCAGCTCATTATTCAAATTGTGTTTTTGTAGGCGGATGTTGAAGAAGCA
AAAACACAAGAAAATGCAAAATTACAATCTGCTTTGCAGGAGATGCAAGTTCAGTTCAAGGAAACCAAGGA
AATGCTTGTCAAAGAACGTGAAAATGCAATAAGGGCCGCCGAGCAGATCCCTATTGTTCAGGAAGTCCCCG
TTATTGACCATGAATTGATGAACAAACTTAGCATTGAAAATGAGAACTTAAAGGTAAGTTGACTGAAACTTTA
AAGTCGTTTCTATTGTTGCTTTGTAAGTTTATTAACTGAAGCTACTCAGCGTGCAAGTTTTGATAACTATTTTT
GAATATTAGACTATGGTAAGCTCTCTAGAAAAGAAGATTGGTGAAACAGAAAAAAAATATGAAGAGACAAA
CAAACTTAGTGAGGAGCGGTTGAAGCAAGCGATGGAGGCAGAGTCCAAGATTGTTCAGTTGAAGACTTCT
ATGCAAAGGTTGAATGAACTTCTAGGCATATGTAGCGTAAATTGATAAATGTGTAAAATTGTTGTTGTCATACA
GACACATTACTCATCACTGGGTCTTTTGGCTACTAGGCTTGAGGAGAAAATTGTTGACATGGAATCTGAGAA
CAAGATTCTTCGACAGCAGGGCCTGTTAACCCCTGCCAAGCGGGTTTCAGATCATTCACCTAGTCTGGCCTC
TAAGGTACTTACCGGGGAAAAGTCATCATTTTCATTCTTCTCTTTCCTCTTGATCAATACTGTTAACAAACTTG
AGGCACTGTTTTTGTTTTTTTCAGATTGTTGAAAATGGACACCATCTTGATGATGAAAACTATACTACTGTAA
GACATGATTTTGTAGTCATTAAGCTAAATCACTTGCAGGATGCTGTAAACTCTTATTTACTGCTTATGAGAGGA
CTAATTTTTGCAGGATGCACTAAGTTCCTCCACACCTTCTAAAAATTTTGAAACCCCCGACAGCAAGATGAG
GAGACCTCCTGTTGATCGACAGCAGCACGTATGTGATTCCATAGATAGTTTGGATTATCTATTTCTTAATTCCT
GATAGAGAGTTTTGTCAAATGAAATCTTTGTTGACCTTGCTACAGGAGGATGTTGATGCACTCATTGACTGTG
TGATGAAGGATGTGGGGTTCAGCCAAGGAAAGCCTGTTGCAGCTTTTACCATCTACAAATGCCTTCTCCATT

GGAAATCTTTTGAAGCTGAGAGGACCAGTGTGTTCGATCGTTTGATTCAGATGATTGGTTCAGCTATTGAGG
TAAGTACTTGTGGGTGCTGATTATTTACATTGGCATGTGCATGTTTTATGGATTTTTAAGAACTGAAGGCTTGT
ATCAAGGAACCTGAAGAGGTTGGACAATAATCACCTGACTGCAGTAAAAAGTGATTACCTGCTTCTTTTTTT
AAGGCCAGGAGCTAAATGTCCATTTAAGTAAAAAAAAATAGAGATAACATCCATCATCTTTATTATTGTTACAG
AGAAAGCATTTTTGCAGTTTTCATCAAAAAAAGAAAGCAATTTTTGCAGTTGCTATTAGCATTGATTTGGAA
AGGACAAAAACTCTGCTCATAACATTCCTATTTAAATTTTAAACCATTGTGGAGGGGGGGAGTCAATATGATA
TAGATCAACATATTTACCTATATCGTTCTCTTTTGAGGATGTCAACAATGAACGTTCTCTTTCCTATTGGGAATT
GATGATCTTATGTATGGTGAAACAAATCATCTTGCTTAACTGAACCTGTGGAAAATATCAATTCCTGATTTTTG
AAGCTGTCAGTGGGAGAGGGAGGGTTATCAGTGTTGGTTGAATTAGTGTGCCCAAGCAACAGTAACACAA
ACAGATTTGGTGGAGTTACTAGCTATATATCAATATTTTCTCTTGAATAAGTTCATGAGCTCCACATTTTAACGG
ATACCTTAGGTTTCTGATTCTTGTCTGGAAATGAGCTTTCCAATCTGAGGTTATCTGGAGGTGTGACCTCAGA
GATAATAGCTGGAAATCAGAGACCTGTAGATGGCAACAAAGGTTTCAAGGTTGAAAAAGAAAGTTATAAAT
CCTAGAGTAGTGAAGCAGATGGGCACTGCGTCTCAGCGTGAGAATTGCAGAAAGTATTAGCTGCAGCTTTT
GAATATTATATTTTTTCCTATATTTGCATAATTCAGTTAGCTGCTCTATATTGTCCGTCAAATCATCATCAACAACT
ATAGGGTGTTACATTTTAATGTGAAATACTTTCTTGATTTAGGAAGTCCTTATGTTTCATTTTGCTGGAGGATT
GCTTGCTAGTTTACACTGAAGTAGAAGTTTCCAAATTTTAAATTTATTAGACATTCAAACCCGATCAGAATTAT
GAATTGATATCTTTCCATTTGAAATAAACCTGATACACCACATTTAAGGTTTAACTTAATGTTAAGTTTTAGAG
GCAACTTCAATCATCAATGAAACATGGAATAGGAATATCATTGTCTAGTAAACTTCAACATAATTTCCATCATTA
CTAAAGCTTGGAATAGGAAAAATATCTGTCAGTTTGCGCTTTCTTCTGAATTGTTTAAATCATCATCCATGAGA
CAATGAATACAGGGGAAGCAGGAGTGCAACCTTCTTTTGTCAAATATTTGAAGTTTGATGCCGCATTTAGAA
CCATTGTGATTAACTTGAAGCATAACAAAGTATCTACTGATTGCAGAATCAAGAAAGCAACGATCACATGGC
GTATTGGCTGTCGAATACCTCAACGTTGTTGTTCTTAATCCAAAAAAGTCTGAAACCAGGTGGTTCAGTTGG
TGCAACTCCTACCCGCAAACCACAACCTCCGACATCTCTATTTGGGAGAATGACAATGGTATTGAAGTTTTTG
AATTTTTCTTCCTTGTTTAAGCTTAATTGTAGTCCTGTCAAGAGTAGTAGCTATAGTAGTTTGACTCTCATGTCT
GTTCTTTCTGATTATGCTGTGTTGTTTTCAGGGATTTCGTTCGTCGCCTTCTGCTGTCAACCTTGCTGCAGCTG
CAGCTGCATTGGTAGTGCGTCAAGTTGAAGCAAAATACCCTGCTCTGCTTTTCAAGCAGCAGCTTACAGCAT
ATGTTGAAAAGATTTATGGAATTATTAGGGATAACTTGAAGAAGGAGTTGGGATCACTCATTTCCTTATGCAT
CCAGGTTTGTCGAAATATTTTCTCAGAAATCCACTACTGCATTTGTAAGAGTTTTCTGTTTTTTTTTTTTTTGG
CTTACCACTTCACCAAGTTTAGGATGCTCTTAGAGGTTGTTGCTTAATTGCTAATAAATATTTGAAATATTGTGT
CTCATGGGGTATGAGGTCGGATTCTATACAGGAACTTCTCTTAATTTTTCTTTGTATTGTTTTTTAGGAGAATG
GTCAACATTCTGAAAGTTCTTTTTGTGTTTTTGTTTCCCTTGTCAAATGAGTAACTTAGTAGGGGTTTTCATCC
TCTTGAGAAAAAGGGAACATATTATAATGCTCATCACATCACTCATATTTCATGAAGAAATGATTTCCTGCGGT
ATATCAAGTCTTGAAGCATTTAGTTAGTATACCCTTTGAATTTGATCATGAGTAAACCAGGTCATGTGGTAAAA
TTCTCTTTTGATGCATACTTTTTCTCCCTTTTTGTCAATATTACTCCTTAGAATGTACCAAGGGAAGAAGAATG
ATAGTTCGCTCTAGCCATACAAATTGAAGTTTTTCACAAATAGTGCTTGGGACATGCTTTCTGATTTTTTAGGT
TATTGAGTATAGCTCTAATTCAATAAAGAATGTTATCCATCTCTCAAGTTATAATAAAAGATCAACATATCCCTAT
AAACCTGCATACTTGTGCTTTTATTACTTGATGATCTAAGACTTGAGAAATAAGCTCATCTGAAATGAGATGCA
TGTATTTGTAACAATTTAGGTTATTTCGTACAATAATATTATAACTCACCAAAGAAAACAATTATTAGCTGTAAG
TTATAGTGATTAACATTTTGTAAAGATAAAGGAATTAAACAAGCTGGAGCAGACCTGCATTGATTTGGTGATA
AGGTAATATTCTGGTGATGTTTGTCGGGTGTTAGAGCAGACTTGCATATCGTGTCAATGTTCCTTTAAGGCTA
AACCATTGTGCTTAGCAAATAATAAACCAACAGGACGCTATCAATGAAACATTACCTTTATCAAAACAAAAAA
TAAAGGATGCTATCAATGAATAGGTATTGCTAGGGCCATGCAAGCTGCTGAAGGTGAGATGAATGTTAACAT
AAAACAATCAGTTACCACACCTACTTATGAGATGCTTTGAACAGACTCAAATCTCACCTCCCTTAGCATTAAAT
ATATGAGTCACTTCAGTGCGCACTAAGACAAGCACTCATGACTTCTGAGGCACACCAACCAGGGCTATCTCC

CAGGAACTCCTCGCCTAGTGAAGAGGTACTAACCGTCGGTCCTATTGTGGCAAAGGAAGGCACCATCCCTTC
TTTCTCTCACAATCATGATAGCCGTGCAGTTGAACATTTGAGTTATGGAGAAACTGAGTATCTAAGCTTATGTA
CTTTAAACTTCTTTTGATTACATATTTGACTGAAGAACTGAAGTTTTGTTGCCTAGAACATAAATGTTATATATA
TATATATATATTATCTGCTTCTTCTCTTTTGTGGTTGGTTCTTGAGATTGTTCGAATTTAGCGCAAGATTTTCCTA
TTCTCTTTCAGATTTTAGTGTAGCTTTGTGGGCTGAGTTGTTTGTCAATTGTAGCCTTCCTTCTACCTAACAAT
AGTAATATGTTGAAATTCAGGCACCAAGGACTGCCAAAGGAAGTCTGAGAACTGGACGATCCTTTGGCAAA
GACACTTCGACAAATCATTGGCAGCGGATTATTGAAGGCCTCAACTCTCTTCTCTGTACATTGAAAGAAAATT
TTGTATGGATCTCATTACATTTTTCTTTTACCAAATTTCCAAGAAAAGCTAATGGTTGCAGTTTATATATATCTAT
GGATGAGTGGAGTAAAATTTTAAAACTAAATTTTGTTTCTTTTAGGTGCCTCCAATTCTTGTTCAAAAGATATT
TACTCAGACATTCTCTTACATTAATGTACAACTGTTTAACAGGTGAGTGAATTTTGTTATTCTAAGTTGTGTTCT
TATTATATGCCAGTTGTCAAATGTTCATTCAGCTACATATTTCAATTTGCAGTCTTCTTCTTCGAAGAGAGTGTT
GTACATTCAGTAATGGGGAATATGTTAAAGCTGGGTTAGCTGAGTTAGAGCTATGGTGCTGCCAAGCAAAAG
AAGAGGTAAGTTCGGCATGTTTGTAATTGTACACATTGCTACTCATGTCTCATTATTGTCAGCTTTATTGAAAA
AAAATGAACTCATTTCCTTTTTGTTCTTTAATTGCAGTATGCAGGTTCCTCTTGGGATGAACTCAAACATATTA
GACAAGCTGTTGGGTTCTTGGTATGTTAATTGTGATGATTATTTCTTGGTATCATTGCTTAGAAATATAGCTGTA
TTAACTTGCCACAAACTGTGAAGGTCTTGGTAGTGTTGAAGGGCTGAGTTGTCTTAAATGGTTGCCCTTCCT
ATATATGTGCCTCATTTATTATTTTATTTGGATATCACTTTTTGACTTCTTTCTTTTATCTGCACTAATGCCATGTG
TGGTTTTGCCTTGATCTTTTAAATTATTGGATTAGTTTTTTCAAGTTTCACGTGAAATTCTTTTGATGATGCTAA
TAATTTACTAACGTGATGGGAAAGATCCCAGTATACAAGTAGTTCCATGAAGTTTCCAGCAAGTAGGCATGGT
TAATCCTGTAACAAAAGTCCCTTGACAATTTATACAGAGTTGAATGTATTGTGCTGGACTAAGCTATTGAAAG
TTTCTCAAGGCTTTTAGCTCCTTACTAGTTCTTATTTTCTTCTGTAGCATTTGCAGTTGTCAGACTTAGTTGTTG
CCAGATAATTGTTGCTCTTTCTTCCTTTTAGAATTTGTTCATGTCTACAATTTTAGATGTAAATTGCAACTAAGA
ATTGATAATATAATTTTTTGGGTTTGTTTTTATGTGTTGCAACAACTGATGCAATTTCCCTTTCCTGTTACTTGT
CCGCAGGTTATTCATCAGAAGTATAGAATATCTTATGATGAGATCACCAATGACTTGTGTCCTGTAAGTCTCTT
TTCCTTTTCAATGTTTGTTCTGTAAATAGTCTTCTTTTCTGTTGACATTTAGCCATTTAGATTGAAAGAGTTCTT
TGTCAAATTCTCCTCTTATTTTATTCACTTGAGTTTAACATACAACACTATATGTACTCTCTCTCTAAATTTCTTTC
TTTTGCAGATTCTTAGTGTCCAGCAACTTTACAGAATCTGTACTCTCTATTGGGATGACAACTATAATACACGA
AGTGTTTCCCCAGATGTAAGATATATGCACACTGATGGCTTTTGTCTTATTGAAGTCCTACAGTACATTTTTTTT
TTTTGATAACCAGTCCCAGTACATTTTCAACCTATAGTTGAATTTCTTTTGAATTTCCCTTCTCTCTTGTTACAG
GTCATATCAAGCATGAGAGTGTTAATGACAGAGGACTCAAACAATGCTGAGAGCAACTCTTTCTTACTGGAT
GATAATTCAAGGTGAGAAACTATCCTCTGTGTGTGAATTTTTGTCATCCTCCGAGTCAAATATTGGACAAGAA
ACATGATCATCTAATCTCTGCTGTTCTTTCTTCCGCTCGCCGTACCCTGTTCTTTTACCTTGCCCTCTTCTTAAG
AACTTGGTATCTCCAAAGGTATGGCATACAGCATACAAACACTTGATTGTTTAAGAACTACTCACTTCAACTT
GCTGTCTTTTTCCAACCGCACACCTTAAAGTTTGTATAGGGGTCCTATGACCCCCAAGCTTTTGTGAAGTGGA
ATTATCTTCCCCATTATTTGCTGATGTGGGACAGAGAGTATTAACTCAATGCGCGCGAGTGATAAAGAATGCT
AGTAATACAGCTTATGGACAGAAAACACCATATTTTTCAATCAAAAGCGATATTATCCTAACAAGAGTTGGGA
AAATGGCATTCTCTCTTGGGAAAATGGCATTCTTCTTCTTACAGTTACCCCAAACCCAGTTCAACTCCAACTTC
CTCAGCTTGTGGTCAGAGCCACTTCCAGAAAACGCCACTTCCTTTTCTGTATAGAATATTGCGAGTCAGACCC
AACTCAATCTGCAGAAATCATCAAATTCAAACAAAAACTTGATGAAATGATTTTAGGTCCTTTGATAATGTTAA
AAGAGATAATGATGAAGATGATTTAAGTGACCTTTATCAAAAAAAGATTCAAGTCATTTGATAATGAACGGGT
GGTTATGGTGGAAAAACAATGAGAAAGATGTTTCGGGGGGTGGGGGGGGGGGGGGAAAAGAAAAAAA
AAATTCTTGAACAATTGACTTTCACTCTTGAGAAATTTTGAAGGCATTTTAGTTCTAGGTTCTAAAAGGGTCT
TCGGCTAAAATTGGTTTGAATACTTTGATTTTATTGCTCGAGGTTTGAAGTCGGTGTTGAGTTTGTTCTTAGG
GGGTGGTAGAAAAGAAACAAAAATCACATTCTGGAGAACAAGAGGAAAAATGACGTGGAAAAATGGGTG

GCAGTATTTGGAGTTACATTGCCCAACTTAAATCTGTTTTTTGGTCTCTTGGGATGGTAATTACTCTTATTCTCC
ATTAGTTCAATCAGGTTTTTTCTTCCTTCTTATTATCCCTGGTTGAAAACTTCTTTGGTTAGTGATATTGTTTTG
ACAATTAACCTGTTTCAGTCATTCAAATTCCCTGTGTAATTAAAAGCAGAGGTATATAATTATTTGCTCCCCCAT
CAAATAGATCTCTGAAAGTCAACTACAGTACTCGCTCCACTCACTTGACTTACAAATTGTGCTTTACACACCTT
TTGTACACTTTTGTCAGTAAACTATCTGGGACTTTTCTATTTTCTTCTTTAAACTTATATTCAAATTGTCAATGAC
AACTTTCTGTTATTGAAATTTGCCCGATCCACTGTTATGTTAGTACTTATCCTTGAGTTGAGGCATAAATAGTCC
TTACCTTCTCTTTCTGCTTATATATTAGCTTTTTCAATGTTTATCTTTCAATGAGCTTTTTCAACTTAAAAATAAA
AGAGAGGACATCTTTCAATGCTTCTACATACATCTAGCTCTTTCTCTTTTTGAACAAGAATATTAGTTTCTTTTG
TTTTTTCCTTTCTTTTGCATATAATCCTCTAGTTTCTTGTTTTATTCCTTGTATAATAATTATATTGACTGGAAATAT
GAAAAAATGTCAATTGTTAGTTCTTTACCCTTCATCCATTGGTTGATTAAGAACGAACAAGATTCATCGCTATT
TTTCGTGAGCTATTGAGTTTGAGCAGATGGAAGTGTTTTTCGATACTGGATGTGTTTAATGGACATTAGGGG
TGAGGATAAGCTTAAAATTCTTTCTGATATGGACTTAAAACTGTGACCAGAATTATTATGTTATAAAACAATAC
TGCCAGAAATGAGAAGTCAAAGGATGAATCCTCCCCCCATATATACAGAAGTATTAAGTAAATAAGCTAGTTT
CATGTTGTTTGTGCCTATAACAAAAAGTATCAAGTTACTTTTAAGTTTCTTCTGGTCTCTAGGAAAAAATATTC
TCTATTCAGGTTAAACTCAAATCTATAAATGATGTATACTAGTTGGAAGGAAAGATGTTGAAAGTTCAGACTT
TGTTAGTTAGTTGTCTTGCTTTTGATGAATTTTTATTTATTTTTGATCGGCTGATATTCTAGAGGTCCAATTTATT
TGAATTTCCTCCTCAACTGATTAATTGCTCAAATAATATTTTAGTCTGCAAGGGATAAGCAGAAAGAAGGAAT
TATTCGTAAACCTGAAGGCTAGATTTTGTACTAATACACCTAAACATCCTCAAGTGCAATCTTTTGAACTAGGT
GATAACCTTAGGTATAACCGATACATATCTTCTTAATGCAGCATCCCGTTCTCTATTGATGAAGTCTCAGAATCA
CTTCAAGTAAAGGATTTTGCAGATGTCAAACCTGCAACAGAACTTATCGAGCATCCAGCCTTCCCATTTTTAC
ACGAGTGAGGCCATGGAACCTCGAGGGTTTCTTGCATTATAGTTACAGATTTGTTGAAAATATCCTAGGTGG
ATATATTCAATTTGTTGTATATTCATTCTTCTTATTCCCTTTCAATTTTTTAATACCAAATATAGATTGCTGTAAAAT
ATTTTTTCTTCTTCTTTTGTGTTTATAATTGTAATTCCAATAGCATAAGGTTCAGAGTCAGCAAGTAGTCAAATT
TTGTATTTGTTTTGGTGCAAAGAGCAGCAGCAACAACATTCTTTTATCAGGAATATATAGGGCAAAGTAATAG
TGATGGGTGTAAAAGTTTTGTATTCTGTCCACATTTCATTATGTAATGAAACAGAAATATTGAACTCAACTATT
GTCTTATGGGAATCATACCAAGGTTGATCTTAA
>PGSC0003DMG400031124
GAAAAAACCTCTTTCTTTCTATTGAAACTTTGGATGTGTTTTTCTTTACTACACTCTCTCAACATATAATACAAC
AGGTCTTCACAATACAATATTTGTAGAATCATCACTTCGGTGACGAAAATAAATATGAAACACGAAACCGAAA
AGATTTACATTTTTGAAAACTCACCACTCTCTATTTTACCAATCAACAAAAGAAAGAATTTCAATCAATCAAAT
CCTATAACTATCATTTTCTTTCTGTGTAAAATCAAAAGAAAATGTCCAAAACTTTTATGCTGCTGCCTGCAGCA
GGAACTGAAAATCTGATCTTTGACAAAGGAGGGGAGGTGGTTCCACATCAGACAAGTTGATATTCTGGAAG
GATCGCGATATTTCTTCTATAGAGAATGGTATGCTGTAATTTGGAAGATTTGTTAGTTGAAGAAAGCAAAAAA
AAAGTCAGTGTCTTTCATGTGTTTAAAGTTAATTACCTTGAATCCACATCAAGCAAGAATGTATTATTTGGAAT
GCTGGCTGAGTCTTCCAATGTTAGTGCTCTCATCTTGCTTATGACCTGAAATTTCAAAAATGATCAACTGAAC
GAAGAAGCAGTTCTCTTGAGTCTTGAAACATATATATCCGAGGTCTACTCAAGTGTTTGCTCAGTGTTGCTTT
TGTCTGGTCTTTTATAACTTTTTGTGAACAGATTCAATAAGACTATTAACTTAGAGGAGGGAAAGAAGTGGC
TAAGAACAAAATTAATTTCAACATGATTTTCTTGCATTGTCATTGCCTTACTTCTAAGGCTAAAGCCGTAAAGA
AGCTAAATATGTGATCTTTTTCTCTAGGGAGCTATGATGAAACCTTTTGCAACATTGAGAGAATCATGTGCTAT
CCACGGCCTTTGGATGTTTTAGTAATCATTAAAGTTGTTCTGGAACATAAAGGAGGTAAGTCTTTCACTGTTG
ATGTGAGAATAACGCAAGAGTGCTTCAGTAGAAAGCATTTTCTAGTGCATGTGTCAAGCTGCAGAGCACTCT
TGGCCTCTCTGTTGAAGAAGATGATAAGATTTTTGTTAGGGATGCATTGGAAGTCATTAATTTAAGTATTTAAT
TATATTTTTAGTTGCTTTATTTATTATTCTAGAATAGGGTTTGTGTTTCAGAGTTTCATAAGGATTAGGTTAAGT
TATAGTTATTAGTTTGGAATAGGATTTAGGTCTCCTACTTCCATAAGTATTAGACTTCCTATTGATGTAATAATAC

TCCTATTTAAAGGGGCTTTTGATTAATAAATCAGTAAGTTATTTTTCAGCAAAAAGCAGGAGATTAGAGTTCT
CTCTTCCATTGAACTCTAAGGTTTCAGTCTCCCTTTGAAGAGTTGAAGAGATCGGTGTTCTCTCTTTTAATGA
ACTCTAAGGTTTCAGCCTCCCTTTAACGAGCTGATGTATATATCGCTCCTGTGAATCGATCCTTCCCTAAAGAT
TCATAACAATTTCTCTCCAGCAGAGCTTATACGCACTTTAAGAAGATTGGTGGGCTGCTTTTATTTAATGTTTG
CTTTATCTATATTTTGCTATTTGAGGACATCTTGTGTTTTTGTTCTTCACTGATAATTTTCTTATAATAAGAGAAG
AAGTCTTATGCATGTGTTGATTCTTAGTTTTTTACTTTTACATGATAATATGTGCATGTGTTGCATGACCAGCTG
GTTGCGGACCATTAGCGCCAAACTGACAAAACCTTGAATACCTGTACCAACTCTCATGAAGCTTAGAGTACC
TCTAGTAACACACTGAAGAAGAGTTCTTACCTCTGGCGATAAACCATGGGCTCCATATTTGTCGTCCCAGAAC
ATAGTTCCAATGCGATATATTTGTGCAATGCTCAACATCTAAGAGAAAAGGGGAAAAAAGCAACAGGATATA
GAGTTGAGTTTTCGATTCTGAGTTCTAATCATAGCTAGAATCCTCTTATTAAAAAAGTCATACTTATACTCCTCT
ATGTCTAAATTCTAATATCGTTGTCACTTAGCCTCTTAGTGATCTTGAAATGAGAAGTGAAGTCATTTTCTTTTT
AGAAAACCAAAAGAAAAAGTAGTTAGGGGGGAAGTTGTGGGGACTTTGATTTTTCTTTTTAAGAACAGAA
GTTAATTACCGGGCAGAGGTCATTCGTGATCTCATCCAATGCCTTTTGAGATTTTTGATGTAACACCTGCGAG
GTGGAGGCATGATAATTTAAGTACTCAGCTATGAGGAGATCTGCTTACAGAGTGAGAGTCTGGATGATAGCT
TACCAGAAATCCTACAGCTTGCCTTATGTGTTGAAGTTCATCCCACGAAGATCCAGCATACTGTTTCATAAGA
ACGAATTAAAACGTTGTTTCTTTTATTTGAACATCAGGTTATTACTCTGAACAACTTTTGTTTACCTGTTCTGTT
GCTTTGGAACACCAACTTTCCAGTTCTTGCAGACCCGCCTTCAGATATTCCCCATTGCTAAATGAGCAGCATT
CACGGCGAAGCAACAAGCTGAAAAGTTTTTATATGTCTTACATTAGGATTGAGGACATTCAGGTTACTTCTAA
AGTGAAAACAGTTAATTTGGAATACTAGCTGAATACCTGTTGAAAAGCTGGACATTTATGTATGAGAACACCT
GACTGAGTATTTTCCTTGTAATGGTGGAAGGGACCTGAAAACACAGATGCCACTCATGAAAACTGAAGAGC
TTCTCTAGACATAAGATTTAAGAAAACTGACAACAAGATGTTACTTCACTTACATTATTTTCTGAGAGTATGGT
TAATGTATTGTCTAGGCTATTCACAATGTTTTGCCAGTGTACGCTGGATGCCTGTTGTTTCGCGATGATGTTTG
AATGTATACTTCTAGATGATCCTTTTAAAGGTTTTATCCTTGCAGATCTTGGTGCCTGTCAATTAGTTTTAGAAT
TTACCTTGAGACTGCTGCAAAATTACTGACAAGGGATTCTATCGTTAAAAAATGGTGCTTGCTTACATGTATG
CATTGGTTAAGAAATGGACTTATTTCTTTCTTTAAGTTGTCGCGGATCATTCCATATATTTTCTCAACGCAAGC
AGTTAGGTGTTGCTTAAACAATAGTGCTGGGTATTTAGCTTCAATCCTTGTTCGGACATTTGGGCTTCCCTCA
ATTCCGCTGTATCCACTGGAAATTGCCATGCTTAATGAAGTTGAACGAAAACCCTGCTGTTGACTCTTGAAAT
GTCAACTACATGTCTATACATGGTCAAAAGCTTATACGATGAATATTTACCTGTGCCATTCTTCCAAACAATGTG
GTTGGTGAGCTGCGATTACGATATGGGGACCTAGTAGGCGCATTACCAGCTTTAATTGTGCTTTGCAAAAGG
AACAACAGAGTTGAAGATGTTGACAGCCAGTAGGCTAGATCACCGGTGTTATCCTGATCCTTCATTGGAAAT
GTCAATAATTTTTTGTCAATAGGGAGTCTAAATTGATTTAGCTTTGATCATTCATTAGGACCATTCCTAGTCAAC
TATGGATCAGAGTAAAATTTCACCTCTATAGATGATCGGATGGTATGAATAATCCTATCAAATATATTCGTTTTTT
CTGCTTCAAAGGATCTCCACTGCAGAAGTGCTTTGTACAGGGTGCATGCAGCTACTGGTCTTCCCTTGTCAA
ATCGCTTGTCTTCCGCAAGGCACTTTATCAGTATATCATGGCTTTCCTGATTATTGAAGAATGAAAACAGAAG
TAATCAGCGATGACTACAAAGGCATCACAACTTTATCTATCAGATCAACTTGTAAAGTTCAAGTTTCTTTTACT
TGCCTGCTGCCTATCTGTCAAGGATCTTTGCTTAGTTAAAGAGATGGGAGGGCTAGAATCCTGCAGAAGCTA
GAAAGAATTTAGGTATCATTTAGAATTAGCATTTTCTGACTGTTATGGTCCTTTAGTGAATAACAGGCTAACAG
CCATTGGTGAGAAGCTCATATACCTTAGGTATTTGTTGTTCTACTTTCATTTCTTCATGCACTTCCTCTGTTTGA
TGTTCATTATCAGCTGGATGTGTGATATCAACGGTCTGAAATTACCCAGTCAGGAATGAGTGGTGTTTGCAGT
TCTACAAGTTAAGATGCTTGCAATGAAAAAGGAGGAAATTTTAGCTTACCTCAAGCCCTTTAGGTGCCCGAT
CATCAGAACTTACTACTTGCTCCACAACTACCCTTTGAGTGCGGAGAAGCTCGTTTTCTGACTCCAAGTCTTT
AATCTTGTTCTTGAGTCTGATTAAGAGGAAAAATCAACTTCATTTGAATTCTATATAATAAAATGTTATTATCTC
TGGAAGCTAACTCAAGATTAAAAGTTTCTCAAGGATATTGTTCCATTCTTTTAGAAATAAGTCCAGGTAAGTA
TTCTTGAAGCAATTATATGTTGTAAAACTTACACAAACAGTGTAAATTGTTTCTCTAGGTTTATGTCCATATTTT

TATGCTCAAATTAAAGTGTCGATTGTGAAATATGAGCCTGTTTCGTTGAGATATCAACGTAAATATAAGTGTAC
TATCTGTCTTCTAAATGAAGGATAACTATCTTCTCTAGCTTTTCTTGGTGCCTAGTCGCAACATTTTAGCTTGCA
TAGGTTGTTCCAAGATGTGTATACTTACATGTCCATTTCATCAGAAAGGGCCTCATTTGTTGATGCTACTAAAG
CCTGCTGACGGAGAACCTGATTCTCTGATTCAAGGTTGGATAGATTCAGTTGTAATCTGAAAATAAATATTAA
GAAGAACAAGTAAGGCAATAAGTTTCATAGTAATTCAATTTGTATTAATTTCAAGCTTCAGTCACTTCAACATA
CTACACAAAGAAGTAAAGGAACTACCTTTCTATGGACTCTTGGAACTCCGAAACTCTTAGCTGAGATTCTTCT
GCCTCTTTACGTGTGGCCTGGCATTCTTTTTCAACTTCGTTGTAGCTCTGTTCGAAGTCTTCTACTCTCTTCTT
GAGCTCTCTGATTTCTTCCTGAATATATGACAGGTAAATTGCATCAGATGTGATGCTGTTAGAAGCACACTAAT
TTATGACGTATATGTATTCTGCTGCAGAGATTGCAGTGAGTGGATTTTCTTGTTTTAATTGTGAAAGTTTTTGT
TTTACATTTTTAAGTATGGATAATCTTCATAACTCAAATAAATTTTTATCTTCCCGCTTTTTATTAAATACATTGAT
TTCCAGAATTTTCCTTTTATGGTCAAATATCTTGATGAAACTTTTCTTTGTTTCAAACCCCTTTCTTTGGGGAG
GTGGTTATGACTAAGTTTCTACATGTTTTTATCATCTCACTTGACTTCTTACTTCGCAAACAAACTCCAAAGGT
TTTTGGAACGGCTCGATGCTGTACCAATTACTGTAAATGCATAGAATTATGTTTTTTGATTTTACCTCGAGTTT
GTTGTTTTCCTCTGTCAGCTTCTCCACTTTGGTGTTGTCAATCACTGGTACCTCCTTGATAACTGGAGGAGCT
TGTTCAATGGCTATCTTTGCTGCTTCTTTCTCATGAATAATTGCATCATGGGCTTCGTCTAGTTGTGTTTGCATT
TCTTGTAAGGCTTTTTGCAATTTTGAAATTTCTTGCCCCTTTGCTTCTTCGAGATCAATCTGGAGAAACACAAT
GTTTTTGTTCTTACTATTACTATGTCATAAAATCATTTACTGTTTACTGATGGTATAAGAAAACTCTAATTGCCAA
AAATCTACATTTCTTCACAAGTGAAAATTCTCTCTCATCCTAAGTGTTAGCACAAGTCAGAGTTATAATTTTATG
CAGTCATGTAAGTATACTCTGACTGACTGTGATGCACTAATGATGTTTCCTTTGGCCTTCACTATGTGTGCCTC
CCTAGTGAGCCCCCAACTCTATAACATTTGGTCTCAAACAACTCTATTTTATTGAGTTTCAGGCAACTATAATA
GAAGCATTGCTCTATAGAACCCAGCTTGGAGTATTTTCGACTGTTATGCTGTTTCCGTTTAGAGCAAGCATAT
TGATTGTACATACCCTCAAGTGTTTTTCAAAATCTAATCGCCATGTTAACTCCTCGACACGCTTTTCCAGTTTG
TCCTTTGCTTCTTTAAGTGCCCCAGTATCTCTTGCATCCTGCATTGGAGAAGATTTTTTATGCCAGTTAACATAT
AGCAGATATAGTGGCACTTCAATGGATTTGAATAAGCAGTAATGCCAATCTTTTCACTAAACAAGAAACACTC
ACCATCCTCAATTTCCGAAGCACCTTCCTCGCTAACCTGCCTCTCCAGAGACATTGAAGTGAAAGACTTGCTT
TTTTCTTCTGTTTATAAGTTGAGAAGGCATGGAATCCTCTCCATTGAGTCTGCAAAAATAAAAGACGAAGTAA
GAAAATAAGGCAATGTTATGTCTTTTTTATAATCAGTCTTACAATTGAATATGAGGTAATAAGGTAACTTCTGA
TTATCTTCATATTAGTGCCCAACAGAAGGTTAATTTCAGGAAGCAAATACTGACAGTAGAAAATAGATTTGGA
CCCTCTTTAGATGTGGTTTTCTTATTCTGTTTACCTTGAAATAACTTTTGTCAGATGAATGAACGTAGGATGCA
AATTCACCTTTTTTGGTGCAATCTAATTACCTGAACTATTTTAGCTGCTTTATTTCTCCTCCTCTGCCTATATTCA
TTTCGTGCTGCCATAGCTCTCATCCCTGTTTGAATGACTACAGCTGCTGCCTGTAACTCCTTGTAAGATTTTCT
TGCTGAATGAGAACGCGCATGTTTCTGTATGCGGATTGAAGCAGCTTCCCTTTTCATTTGTTCATACAGCACT
CTGGCAAGTTGTGCTGGGTAGGTAGAACATAATTTTTTTCTCAAAGTCCCGACAAGAACACCATATAAAAAT
GGGAAAGCTAATGTAGCATTGTGTTGATTAACATATTTGTAAGATTAATATTAAGAAAAGTAGTTGGAACCCT
ATTTTAGGGAAAATTCAGAACATGCCAAAACTAGAATATGTCAAATCAGCATGTATGCAAAAAGTGCCAATCT
TGTTGTTGCACATATTATGCTGACACTTCTGATTTTTACCATAAATGAGTGGTCATTTGTATCATTGCGGTACAA
GTTACCTCTCCAAAGTTTCTGGAAATGAATTGTAGCTCTCTTTAGGGCTATGAACTCCTTTCGGGTAAGATAT
GTTCGAATTTGTCTCTGAATGCGCTTTGCAGCATGAGCTAGAACTTCTGTTCTTCTGGCATCTAATTCAGCCAT
CTGCCCGGCTCTGAGAAAAACTTTGGTTTTCCCAATCTGAATAAAATAGATAGAAGACTCTATTTCATGAACA
AATCCATAAAATGTGCAATTTTATGTCCAGTTTTGTGGAAATGGTTTATTTCTGTTTATAAGTATGGCAATAGTC
TAGGTGTAATACCTGATAACCCTTTAAGCCCATTCTATCACAAATTGCAATGCATGCTGACTTCTCATCACATCT
GCTTCAACGAAAGGGGATTAGCTTGTGATATATCAGTATGTTGATGCTATTCATGTACATACAATAACGTTTAT
GAGTCTAAAGAAGACTGTTTGCTGCAAATATGGTATTGCTACTACTAGTGTATAATATATTTCTCCCTGCCTCAA
ACTTCCAGTATTTGTTTCTTCCTGAAAAGGATTTAAGATGTGAAACCAAGTTATAACCGATGTGATATATACCC

GTCAAGAACATCTGGAGCTAACGTTCCAAAGCGGTCAAGGAACTCATCGAATGTTCTTTTCGTTGGGTATCC
TGCACAACTTATCCTGATCGCCTCTAAGACACCCTGCAAAAGTGTCACGAAAGGTTTGTCAGAGAGAGAACT
ATGAAGAATCACATATTTTCTGATTGAGAGTAGAAAATTAAAGCACCAAAAAGTACATGGAGCACTTTAACTT
ACCCCACATCTTAATTGGTTTAATACATTCATGTTCTCAAATATTCCTGGCTTTAGAACTGTATTGGGCTTTACA
CATCTGATGTAATGTGGCTCTGTAGTGCTCAAGGTCTCCATTAAAGATTGCAGTTGTTGCTGCAAATTGAAGT
TCCGTTTGAAAATAAATCAGGTTCATAGGATTTTCTGAAGTAAATATACTTGTTTCTGTCCTGTTTATAGACTTA
CCTTAAACCGTGTGCCGATGGATGAGAACTTGGACTGCTTGGACGATTCTTCGGGCAATGGAGGGAAGAG
GTTTGCAACGAAAAAGCACTTTGAGTCCATCAGAAGAGCTTGAAATTCTGCTATCACATAGTCCTTATTTTTG
TCAAGGAAGTGATCTGCTTGGTAAGTAACCTGTTTGATCAAAGATTTAAGATGACCAGAATTCTTTGACTGA
CAATTGAATGACCAAATATCTCAAGTTAACATACATCACCAGCGTAATGATTGATTGTAAAGTCAGTACGAGC
AAGTTTTGGCTTGCTAAACCGTTTATGGGCTCTGTATGTCTGGTACATCTTTTGGGCAAATGTCTCATGAGTT
GCTTTTGGGAACATACTGTGCAAGAAAAAAATTAAGTTATATCTTCTCCTTCAATCATTCTTTATAATTCAGGA
AAAAGGCATTATACACTGTGTTTTGAGAATTTAATGACTTTGTCTAGCAAATTATCCTACTTTCACAGGTAGTT
ATGGTGTTGAAAAGAGGAAGTTTGCGTACCAAGCTTCATCAAGAAGAGCAATAATGCCTCCAGGTTTCTGC
AAATTTGATGCAAGGTTATCAAAGGTTTGCTTGTTAAATTAAAATAGTGTGACAAAAATGTCTGCAGTCAGA
ATGAGGGTACCTTCTCAATAAGATCTAAAACATCTTGGTTATCTACAAACTCCACATAACTCCAGTTGATTTCT
TCTGTCGTATAGTCGTCTTGTTCCATCTTGAATACGTGCTGTAAAAACAGAGTTTCTATTTAATATTCTACTTTAT
TTTAAATGGGAGTGAAAAATGAAGGCACTGAAGAGTGAATAAAGGTTCTCATGATTTACCTGGTTAAAATGC
TGCTGCAACTTCTCATTTGTTAGGTTGATGCAGAATTGCTCAAAACTGAGGTTAAACATTAAACGAGCTGTTA
AGTTTGAGGTAAAGATTTTATATACTCAGAAGTGGAGAAAACAAGTATCCAAACCTGTTGATTTTGAAGCTC
TCAAAGCCATATATATCAAGGACGCCAATTATGCTTTTTGCTTCAGGATCCTGTCCAATTGAATTGTTTATCTTG
TCCACAAGCCTGCCACATAGAAGCTGTCAAAAGGATTTCATTTTGAATGTTGTTTTTTCGTGTAGCTGAATCT
AGAGTTATGCTAGATAATGCTAATAAACTCAAAAAATAGAATTTCTGAATGTCTTTTTGAACTAATATGATACT
GACTCACTGTGGCAAGCAGCCATCATTCTTCTAGAGATTGACTATCTGCATGTCAACAGGACACAAAACACT
ACTATGGGTTGGTTTCAAAATTTGAAACTAAAATAGGAAACCAGGCTTGAAACATACATAACATCATTCAATC
ACATTAAGAAATTTCAGCTAGAAAAATTCTCCATTATCTTCAATCCTGTCTGATCACTCTTCTATACGTTGAATC
TCTTTTGTTCTTTATGAGAAGCTCCAATTAGTTATTACTTTATTCTTTATAATCAGAATTATAGTGACAAGAGAA
TTAGAAAATTTTGGTATAATTGTGCATTATTTGTGGCTTCCTGTGATTATCCAGAGCTTCATTAGGAAAAAGTT
GTGACATATATAAAGTCACATTCATGTGCAGTAAATGTTATTGCCTATCAGGTTCCAGGTTATTCTGTATTTCTT
TCACAATAAACTTGTGACTTTTGCTCTCCCTGGCTGTTAATGCCGTAAGTATCCAATTGTTTGGAGTCGAGAA
AGTTCTTTTTTGCTTTGTTAGTATTAAGATGTTCCCCTCTCATGAGCTTAGATACCACCCACAGTTTTGCAGCC
AGGGAGAGACTTAGTCTGGGAGCCACATTCTATATATTGCTTACCAGTCAAACAATCTGGAGTATACGGTCTT
TGCCAGGGCATCCCTGCTCGTGGTTGCAGCTGCTGGATCCAGTAGTTTTGTGATGTTTCCATCAGGAGTTAC
AATGACACGCTTACAAAGTGAGTCTTCTAGTGCCTTCTCATCACACCTGTGCATATGTTTATTAGTTCAAAGTT
TAAGCGCCTTGAGTTCTGAGTTGAGGTAAACTAAGGTATATCCTCTTACATGAATAGCTCTGCAGCTGTCTTC
AGATGAAAAAGTGACTTCTCATCTTTTAGTTTGGAGGAATCTGCCTCCTTTCCTTTCACAAAGTTGATGTTTC
CGAGATGGAGTATTGCAGCTACAACACGAAATATAGCTTCCTACAGAAATGATGAGTATAGGTTTTAATATATT
CTTGGTGCATAATGGGATTTTTCCAATTTCTTCTGTATTCTGGGCACACACCTGCTCCTCCTGACCGATTCCAA
CAACATCCATAGCATTTCTGGTTTCAAGATACTCTCTTGCATCGTCAACATTTGCAACTTCGTAACAGCTACTT
TGGTTTAGATAATGAAATGATTTTGGATTCCCAAGTTTGAATCTTTTCACATCCTGTAAAAGATTCCGTAGATC
ATCTAGGTGGAAGCTCAATATCATTTGTAAAGAGTTGGAAATGGGCTGTCCCATGGAACTCACCTCTGGTGG
TGCAGCACAAAGCATGTAAAAACAATGGTAGTTTCTCTCTGGGTCTGAGACTTGGCAAACACGTGACCTTTC
AAGAAGATATGTCCTAACTGCAGCCCCAGAAATCTTCGCGTGCTTATCAAACTGAATTTCAACAAATTTACCA
AAGCGACTGGTACAACGACTCAGTCATGTCAATTTTTTGTAGAGAGAGAATATGAAAATCAAAATGAATATA

ATAGTGATAAAATCTCTGATGTCATTTTTGGTTTACCTGGAATTGTTGTTCTTCACAGTCTTTGCATTCCCAAAT
GCTTCTAAAACTGGGTTGGACTGAAAAAGAAGAAATATGAATTTGCATTTGCATTGTTAACTTGCAGATCATT
CGAGTATAGTAGTAAAGAGCAATTATTGCCCACCTCCAAAACTTGTTGCTCGACTGTTCTTCCTTCAGTGCCA
GACCTTCCACCCATAAACGCGAGATATCTCATTAACATTTTCGTTGTCTCAGTTTTACCAGCACCACTCTCTCC
ACTGACCAAGATAGACTGGTTGCCATGCTCATTTATCAATGCCCTGACAAACTTGTTAGATTAGGCAAGTTGT
TCATTTCTTGCTTGGCATAACCAAATAATTTCAGTTGCACTTCTGATTTACCTATAACAGGCATCTGCCACTGC
AAAAAGATGTGGACTTAGCTCTCCAAATGGGGCTCCCTTGTATTGCTGCATCATATGGGTATCATACAGATGT
GGAAGTCTCCGAAATGGATTGACCGCAATTAGGATATTCCCCGTGTAAGTCTGCAAAATTGTGGATATTTTTA
CATCAATAAGACTTGTTTTCTGTAGCTACTCTTTGTGGAGTTCATCATCGACTGAAAAGTATGACTTACATATAT
CTCATTTAGAGAATACCGACAAGCAAGGTTATTCAGAACGCCAGGCTCATGGAGATAAGCTAACTTGGTCAT
GTCATCAACCCCAGATGGTGGTGCTTCGGTATCCTTTGGGTATATGCTAGAAATAGAAGCAACTGTCTGAAGC
AGAAAGTAGTTCAATGTCTTAATATAATTAGATTTTCATTGTTCGCATGTTGAAAACTTTAGTTTGTAGAAGTC
GTGATTATTGCATCCTAAAAATATCAAGGGACCACATTCCTAAATCTTCATAGCAGAGTACACCTATCATGAAA
ATCATTAATACATCTGCTTAAGGCAAAAGGAAAAGCTGCATAAGCTGCCCTACAGGTATGAACCCTCAATTCG
ATTGTTTTCCAAGAATGTGTGACAGTAGACAGGAAAAAAGTCCTTTGTTGTTTTATATCTGATTGATCATATTG
AACTATTACATTCCTATTTGTTCCATGACCAATTTCATTTTTTGGGGAACTTCTATTCCAGGTTGTATGTGTTTC
AATAAGCTTATTAAAGCTAATAAGGTCCTAATTTTCATATACTATACAGTATAACTTTGATATTATACTACATCTAC
TTTTTGACACTAGTATGGTTCAGACATCCTTTTTCTGCAATCATATTATATTCCCTGAAAGATGCTATCAACATC
AAATTTCTGTGAGGCTCTGAACTTATTGATTAGAATAAGACTGCTTACCGTCTTCCCATTAGTTGTGGCTACTG
TGGCATTTGAGCCTTTAATTTCTGTGACTTCTCCATCAATCCAGGCATCATCAGGATCCTCAATCCACACCTGG
GATCCAACTATGATGTTTACAGGAGTTCCCTGAATCACAAAAGATTATTACAGAGGTCAACAATTGATAAGTG
CTTCATCAATTGAAACAGAACAAGATAAACTATTGAACATAATAAACAAGCTCACCATGGCAGTAGTTGAGA
CTATTTGATCATGGAACTCAAAGGAGAATTGTTATAAGAGATGGATGAATTGATATAGAGGGAGAAAAGGAA
GAAGGGAAGAGAAATGAATTTCAGGGAGAATGTGGTGCTCTTAAGGGAATGATGAATAGATGAATGTAGA
AACAGGAAAGTAATAAAAAAAGTG
>PGSC0003DMG400020947
AAAGCTTCAAACAAAATATACATATAAAGTTGTTTAGAGAGAGAAAGTCTACCCATTTTCTTCTTCACAAAAT
ACATACAGATATACGGAGGCATATACATATATATATATACGCAGATATATATAGATAGAGAAACAAACAGAAACG
GTTTTCGCTTTACCATAAGTTGATTCATCGGAGGCAAGCTTTGCTTTCTTCTTTTTCATCATCGTCGTCGCTTA
CTTCGGCTGGAATATTTGAAGTTTGGGGAGTCCGATCTGACTCACCGACTCAAACTTCGTTTCTCTTTGCCTT
GATGAGTTGGGGAGCAGGAGATCGATAGTTCAAAACTCGTCAATGGTAAGTCTATGCTGAAACGGCGAGGT
TCCTTGTCGTTTTATACCTCTTGCCTTGTTTCATTTCCGAAGGACTTATTGCTTGTTGCTCTAGTCAAATCTGTT
CAGCGCAGAGTCAATTTGCCGTTTATTAATTATTTTGAGGTTATTGTGTGGATGAAATTTAATTCGGGTATCAT
TAGGAAATATTAGCGTTTTTTTTTTGGGTATGTGTGGAATATATAGGAATTGGACCGAGAAGAGTGAGAACT
TCTATGAGGTTTTTATGACTGTATATCGATGGATGGTTTAAAGGTATTTCTTTGAAGTCATTTCGTAAAATTTGA
TTTTATCCATTTTTTGTTAAGTCTGGAATTTGGATGTCAGAAAGAGATAAACGCTAGAATTAACAACTGAATT
GTAATTTGATTTTATTATTAGAATAAACAGAGGTTGCAGACCAGAGTGAGTAATCCGCATATTAAAAGAGGTT
GTATGTAGTGTTAATTAAGAGGCTGCTTGTAGTAATAATTTGAGGAGCTAAAATTTGGAAAATGCAACTTCAG
CTTTAGAGGTGAATGTGGCAAATAGACACTACACTACTGTGTTTAAATTAAACTTTATCTTGTTAGAGGTATCC
ATATTTATTTATTTTTGGAAATATTAGAGATAGAAATACCATTCAATAGAGCACTTAGTAATAATAAATAAGATG
TAAAAATTAATAATAGTGAGATAGAGTTTTGGATACTGCTACGGAAGGCGTGTCTTCACAGTAGATTTCCGTC
GCCACCTAGGTAATAGCAAGGTGACTTCCTACTCCAAAGGTATAACAAACCACTTAAGAATCTTCTAATGTGC
ACTCAAATAGAAGTTCCGGAGAACTCTTAAATCTCTCGACTCTCTCAACTGTTTTTTGCTACTTCAAAATCTCT
CAACTGCATTTATTTGAATTTACAATGGATGAAGAAAAGAACAATATGAATAGTGGAAAGATGAAGGGCATG

AAATCACTAGATTTGAATATTTTGACCAGTGGAATAGGTGTTTACAGGGAAGAAATAATTAACAAGACTTTCC
AAGGCTACATGTATACTTTAAAGATTGAATTACAACTTTTTATAAAGAATGGATGTATCGTTTATAACATGGATG
GGGCTTGGTTACGTGTAAGAGCTTAACACGTGATGTGTGGGTTAGATGCATGTCATGGGTTCGAACCCTGCC
GCTGAGAAATAGGCGCATGTCACATGTTCGAACCCTACTGCTGAGAAATAGGCGCATGTCTCATGTTCGAAC
CCTACCGCTGAGAAATAGGCGTATGTCTCATGTTCGAACCCTACCGCTGAGAAATAGGCGCATGTCTCATGTT
CGAACCCTATCGCTGAGAAATAGGCACATGTCACATGTTCGAACCCTACCGCCGTCAAAAATCTTGTGTTTAA
GTGGAGAGAGGTAGAGAGGTGAACTCATTATCCACCGAGTTCCGAATCTTGCCCCAGTTGCCTTCAAGGAT
TTCTTGGTCATCAAAAAAAGAAAGAATGCATTAATTTTTTCAGAAAAGGTAGTCTCTTTGCAACAGTAAACA
CATTAAATTTTGAATCATTGAAAATAAATTTCAGTGTTTGTAGCACCTCAATGCTCTAAAATCAAAGTTGTCCA
ATGTCAGATGCAGCTATGACCACTGATCTCTTGTGGAGTCTCCTTATGTAATTACATTGGTCCAAAACTTGAGA
TGTAAAAATGAGTGACACATGGGTGATAAGATGTATATTTGCAAATATATATCATGGGACTTCTTGACACTGCA
GGAAGGGAAGAAATTGATTAAGATATCATTTTCAGTTCACAAAGGACAGCCATATTAATATGAATTTAAAAAG
TGTGTTTGTTGTTAACCTAAAAATACTACAGCAAATAATTATGTGTATTGGTCTCTTGCATAAACGAATAATGTG
TACTAGTCAAAGTAAAAGCCCATGTAATATTTTCACAATGCAAGGATACAGTTAAAAATCAGCTCTTGACATTT
TCTTTTTGATTTTTTCTAATTACATTAGGGGAGTGGGAAGAGTGAAGGGAATAGTTTGGATAGAAACCACCT
ATTGTGTGGAAGTCTGCGACCAGTACCACTAGTCTAAAGCCCTAAACGTCATTCTAGCATCTAAGTGATGAAA
GTTCCTATGGTCAAATTAGTACAAGGCAGGTGTTCTTTCAGACGTTTTGTGCTTCAAAAATATTTCATAGCTG
ATGAGAAGTTTGTAAAGAGAAAGAACATAAACAAAGTTATGTTTCTTTGTTACTTTTATCAATTTGATAACATT
TTCATTATTCGAGAAAGAATTTTGTATTTGCTTTGAAAAGGATTACAACATAATATTGCTTCAAAGAGGAATG
AAATAGGTTGTCATTTTAAGGAACCCATTAAGAAAAGAAGCAAGAAGGTCACGAGAAAAGAAAATTTCTGA
AGTTTCTGTTTAACTTATTTTTTTATCTGAAATGCTTCAATTGAGTTAGTGGAAGGTTTGATAGTTTCTTACATT
AGCTTTAATGATCTTTCCCGAAATATCAGCTGCCCTTTTTTTTATAACTATGGTGGCCACACCCACTTGCACGC
ACCTCGACTGATCCACATGGTACCTTCTATCTCCCATCAATGCTAGCACAAGTTTGGGGTGACTTTGTCCACC
AAAGCTTAGATAGATGGAATGAAATCATCTAGCAGTTCTTTTCCTCTCAGCCCTCTCCTGATATTTATTCTAGA
AATATCATCGTTTATTTTAGGATTCCAATTTTCTTTGTATATAGGAAGGATGCCTTAGAGCTTACTCATGTTAAG
ATATGTTTGGAAAGCGTTGACATATGAGGTGCTTCATTGTATTCAAATTTTAGCCTGCCTTGCCTGTTCAAAAA
GAATAGAAGTTTAGACCGACAGTTTCATTCCCTGAGTGACTCCTGTCCGTTTAGCTATATGTGTTGCTTTACAA
ATTTCCTATTATTATTTTTCGTATTATGATAGAGGATAGTAGAAAGACCTCTATGCCTAGCACTGATGGAGAGTA
CTGCTTTAGATGCATGATTTCTTTTTCCGGCTGGATTCTGCAGTTCGACTGCATGCGTAAATTTCACAAAATGT
CTTGCATCTTCTTCTTTCTCAAAAAGTTGTCTTGCCTTTTCTTGTCTGTTTCTTTCCATCTCTTAGTTTCTAAACA
AGGCAATGCTAATTTATTTTTTAAAAGAAATGTCAACACTGGACGAATTACCCACCCTTTCTTCCTCCCTTATT
TCTTATTCTCTCCCCGTTAGTATGAACATAGTTTAAAGACTTGTCTTGAAGAATTCTTTGATCTTGTGTCTCAAT
TAATAAATTAAGTATGCCTCAAGTTGGAATTTGATATATAAATCCACTCTATTCAGGTCCATTTTATCTGAATACT
GTGTATAAAGATTAAGTTTTCTGGTGTCTTATTCGAATGACTGACATTTCGTGCAGTAATCATTGTCCTCTTGG
TATCTCATGCAAGTACCTTTTTGCAGGCATCAGTCAACATTATTGTCGGTTCTCATGTTTGGGTGGAAGACCC
TAAATTGGCATGGAAAGATGGAGAAGTAATCAAAATACATGGTCAAGATCTTCATGTTAAAACCTCCGATGG
GAAAGAAGTGAGTATTTGGTTTTTTAACAGCAATATTTTTTCAAGGGCCATACTTTGTGTCAATCTGGAGCGA
TAATGAATGAATGTTCTCATAAATTCTGAACTTGTTAATGGATCTGGTCAGACCTCACTGGTGAAAGTAGATAT
TTTTTAATATTGAGAAATCGGTCATTTGTATTCTATTAAGGTGAAATATCCTGCATATTGGTTCCTTTTCCTAAA
GATAGTTTTTATATACCGTAAAGAACATACGACGTGCTATTGTTTGTATGTGTGTATTTGTGTGTGCATACATGT
ACGCAATATGCATGTGTGTGCATACATGTATGTAATATGCATGCATGCTATATATCCAAGTACACCATACTGAGT
ATATTCAAAACATTCTTATAACTCGTTATGGGTTGCGACCAATGGATGGTACTGAGAACTAACGCCTTCCCAAT
TCTAGCTAATGCAAATAATTGTTAAATCCTTCAAAAGGGCTATGAATGCACAAGCTTAGACCCATGTGATGGT
TCACTGTAACTGGAATGCTTCGCGTGGAGAGAAATTAGAAGCAGGTCTATGGTAGAAGCTTTCCTTTTTTGA

CTGACATTATGAACCAAGTTACTTACGTGTACTTAGATTGGTTCACTTCTCTTTACTTTCTTATAAATGAATTCA
AAAAGTATATAGGGTGATGAAACTGATGAACACTTTTCATATTGACGTTGTGGCTTTCTTTTGCATTTACACCT
TGATTATTTTTCTAAATGTTAATTCTTGTTTCCTCATGCATTGATTCTGCTTTACATGCACTTTTGCTCGTCACCT
TCCTTATGCACCAAAAGTATCAGTTTTCTCTATTTCAAGTTAGTGTCAATATTTTAATTTTGATTGTCTATCGTCA
AAGGCGGATCCATGATTTTAAAAGTTATGAGGTCCTACAATGACTTCAAGTTAGCATACGTTAATAAGTGGGT
TCACACTGAAATTTATAAATATGTAGTACATTTTAATGCATATATAGGGTTAGAGCAAAAGCTACTGGGTCTGT
GAACCCTGCACACATGTTAAATCCACCTCTGTCTATCATCATATTGGCTTCCAGCGAACACTTGATTCTTCTAG
ACTGTAACTTTGTTAACTGCGTTCTTCTTGTAGTGTTAAGTTTTATGTTCCTCCTAGTTTTGCAGTCATTAACTT
ATTCAAACATGAGTAGTTTTTCTGGAGGACATTTCCACACTTTCAGATTACCTGCTGTCTGCCGGTCCCTGAA
GTTTGAAACCAACAAATCAGTTCTTGCATTTTTTTGGTTGTGCCCAAAGTTTTTTCTTTTTTATCTAAGGTTTT
TATTGTTTCAAACTGTTGATCTCAGGTTGTTGCTAAAATCGCTAAAGTGTTTCCTAAAGATACTGAGACTCCTC
CTGGAGGTGTAGATGATATGACCAAGCTTTCCTATTTGCATGAACCTGGAGTTCTGCAGAACTTGGCCACCC
GATATGAGCTCAATGAAATTTATGTAAGAGCTCTGATTTTCCTTGTAATCATGCAAATTGATCGTCAAGACCTG
CTAATTGTATTCTATGAATTTATTATCCTTTGGAGAAACTTTTCTTAGTTTTTCTTTATCTGTTGTCTAGACGTAC
ACTGGGAACATATTGATTGCAGTAAACCCTTTCCAAAGATTGCCTCATCTGTACGACACTCACATGATGGAAC
AGTACAAAGGAGCAGCATTTGGGGAGCTAAGTCCGCATGTTTTTGCAGTTGCAGATGTTGCATATAGGTTGA
GTTCTTCTTTGATGTGTGTTCCTCTTTTTAGTTGCTTTGGCATGACACTTATCTATTTCTCATTTTGGTCTCAGG
GCAATGATCAATGAGGGAAAAAGCAATTCAATTTTGGTTAGTGGAGAAAGTGGTGCTGGTAAAACTGAAAC
TACTAAGATGCTTATGCGTTATCTTGCGCATCTTGGGGGCCGGTCAGGTGTCGAGGGACGAACTGTAGAACA
ACAAGTTCTAGAAGTAAGATGACCTTTCACTTCAACTTTAATCTATCATTAGCATTTTCCGCTGAAACTGGTCT
TATTCTATCAAATTACTGTCTCAAAATTCTTGTTATTGTTAACACTTGACACACAGTCCAATCCCGTTCTTGAAG
CATTTGGAAATGCCAAAACTGTGAGGAACAACAACTCAAGGTTAGCCACTTCTCTAATTGATGCCACAGTTG
ATGCTAATTGCTTCAGCAACTTTGTTGATGATTTCTAAATATGATGTTCCTTTCCGTGATGGAAATGGTATAATG
CTTTCAGTCGTTTTGGTAAATTTGTTGAGATACAATTTGATAAGAGTGGGAGGATATCTGGGGCAGCTATACG
AACTTACCTTCTGGAGAGGTCTCGCGTCTGTCAAATTTCAAATCCTGAGAGAAACTACCATTGCTTTTATCTT
CTTTGTGCTGCTCCAGCTGAGGTATCTATGCTTTTTTTTAATTGTCTTTAGCTGATCTAACATTGGTGATGCAC
ATAATTATGATATTCTTTTGCTTTGCAGGAGGTTGAGAGATATAAACTACAGAACCCAAAATCATTTCACTATC
TTAATCAGTCCAAGTATTATGAATTGGATGGAGTAAATGATGCTGAAGAATATCTTGCAACAAGAAGGGCTAT
GGATATCGTAGGAATCAGTGAGGAAGAGCAGGTTAGTTTTCCTCCCGACTAATTTGTTCTGCGGTTAACATG
AATTGCTAATGCCTTAACAAATTTGTTCAGGATGCAATTTTCAGGGTGGTTGCTGCAATTCTTCACCTTGGTA
ATGTCGAATTTGCAAAAGGTGAGGAGATTGACTCTTCTGTGATTAAGGATGAGCAGTCTCGATTTCATCTCA
ATATGACGGCGGAGTTACTCAAGTAGGTCTATTTTCTACTGTTGAATCTCACTGCATGTTCTTATTTTGGATATG
TTATTTCTGCTGGATCTTATGAATAAGAATTGATAGGTGTGATGCCAAGAGCTTGGAAGATGCACTAATTACA
CGTGTGATGATCACACCCGAGGAGGTTATTACAAGGACTCTTGATCCAGAAGCTGCTCTGGGTAGCAGGGA
TGCTTTGGCTAAAACCATATATTCTCGCCTTTTCGACTGGTAAGCAAGTCAAATACATACACGCACTTGTTTGG
TGTTCTATATTTCTATCTTCTTTTTGTATCTTAAAATATTCGTCTATTGACTCCAGGATTGTGGAAAAGATAAACA
TCTCAATTGGCCAGGATCCAAACTCCAAGTCAATAATCGGAGTTCTTGATATTTATGGGTTTGAGAGTTTTAA
AACGAACAGGTAATCTTTCAGTATGGCATATGCTCTGCTGCTGCTGCTTACTTCAAACCTTAGTGGTGCCTTTC
TAACAGTTTCCCGTGTAAAACAGTTTTGAGCAATTCTGCATCAATTTTACAAATGAAAAGTTGCAACAACATT
TTAACCAGGTTAGATCTCATTCTTGCATTTTAAAACTGTTTTCCTCATTTCTTTATTTTCAATCTGTTAAGGGTAT
ATATGAGTAAGTTGTGATGTGTTTGCTGCCCCACCTCTTTGCTTATTAGGGGGGATCTTGGGTATTTAATAATG
TCACTCCATCTTATGATTTCAGCACGTGTTCAAGATGGAACAAGAAGAATATGAAAAAGAAGAGATTAACTG
GAGCTACATAGAGTTTGTTGACAACCAAGATGTGCTGGATCTGATTGAAAAGGTCTGCAGCCAATGGGTTG
ATTCATTGTTTATTGATTGATTTTTTTCATTTTGTTGGAAAGAGGTATACCTCTGTAACTTATCATGCGATGCTAT

GATTTGTTATTAGTTCTAGCCTCAGTCTTTTGGTAACTTAAATTGTAACAACTGAAGTTGAAAACTTGCAGAA
ACCTGGGGGAATTATTGCTCTGTTAGATGAAGCCTGGTATGCATGCTTGATTATTTATTCATCATATATATATATNN
NNNNNNNNNNNNNNNNNNNNNNNNNNNNNNNNNNNNNNNNNNNNNNNNNNNNNNNNNNNNNNN
NNNNNNNNNNNNNNNNNNNNNNNNNNNNNNNNNNNNNNNNNNNNNNNNNNNNNNNNNNNNNNN
NNNNNNNNNNNNNNNNNNNNNNNNNNNNNNNNNNNNNNNNNNNNNNNNNNNNNNNNNNNNNNN
NNNNNNNNNNNNNNNNNNNNNNNNNNNNNNNNNNNNNNNNNNNNNNNNNNNNNNNNNNNNNNN
NNNNNNNNNNNNNNNNNNNNNNNNNNNNNNNNNNNNNNNNNNNNNNNNNNNNNNNNNNNNNNN
NNNNNNNNNNNNNNNNNNNNNNNNNNNNNNNNNNNNNNNNNNNNNNNNNNNNNNNNNNNNNNN
NNNNNNNNNNNNNNNNNNNNNNNNNNNNNNNNNNNNNNNNNNNNNNNNNNNNNNNNNNNNNNN
NNNNNNNNNNNNNNNNNNNNNNNNNNNNNNNNNNNNNNNNNNNNNNNNNNNNNNNNNNNNNNN
NNNNNNNNNNNNNNNNNNNNNNNNNNNNNNNNNNNNNNNNNNNNNNNNNNNNNNNNNNNNNNN
NNNNNNNNNNNNNNNNNNNNNNNNNNNNNNNNNNNNNNNNNNNNNNNNNNNNNNNNNNNNNNN
NNNNNNNNNNNNNNNNNNNNNNNNNNNNNNNNNNNNNNNNNNNNNNNNNNNNNNNNNNNNNNN
NNNNNNNNNNNNNNNNNNNNNNNNNNNNNNNNNNNNNNNNNNNNNNNNNNNNNNNNNNNNNNN
NNNNNNNNNNNNNNNNNNNNNNNNNNNNNNNNNNNNNNNNNNNNNNNNNNNNNNNNNNNNNNN
NNNNNNNNNNNNNNNNNNNNNNNNNNNNNNNNNNNNNNNNNNNNNNNNNNNNNNNNNNNNNNN
NNNNNNNNNNNNNNNNNNNNNNNNNNNNNNNNNNNNNNNNNNNNNNNNNNNNNNNNNNNNNNN
NNNNNNNNNNNNNNNNNNNNNNNNNNNNNNNNNNNNNNNNNNNNNNNNNNNNNNNNNNNNNNN
NNNNNNNNNNNNNNNNNNNNNNNNNNNNNNNNNNNNNNNNNNNNNNNNNNNNNNNNNNNNNNN
NNNNNNNNNNNNNNNNNNNNNNNNNNNNNNNNNNNNNNNNNNNNNNNNNNNNNNNNNNNNNNN
NNNNNNNNNNNNNNNNNNNNNNNNNNNNNNNNNNNNNNNNNNNNNNNNNNNNNNNNNNNNNNN
NNNNNNNNNNNNNNNNNNNNNNNNNNNNNNNNNNNNNNNNNNNNNNNNNNNNNNNNNNNNNNN
NNNNNNNNNNNNNNNNNNNNNNNNNNNNNNNNNNNNNNNNNNNNNNNNNNNNNNNNNNNNNNN
NNNNNNNNNNNNNNNNNNNNNNNNNNNNNNNNNNNNNNNNNNNNNNNNNNNNNNNNNNNNNNN
NNNNNNNNNNNNNNNNNNNNNNNNNNNNNNNNNNNNNNNNNNNNNNNNNNNNNNNNNNNNNNN
NNNNNNNNNNNNNNNNNNNNNNNNNNNNNNNNNNNNNNTATATATATATTTCTTCTTCTTGTTTTCGG
TTCGTCAGCTTATGATATAATTTATGTTCATTTATTGGTCCTTGCAGTATGTTTCCTAAATCTACTCATGAAACAT
TTGCTCAGAAGTTGTACCAAACATACCCTAAAAACAAGCGCTTCATCAAACCTAAACTTTCACGGACGAGTT
TTACAATATCTCATTATGCTGGAGAGGTGCTGACTAGTTCGTCTTTATGGAAACATCTATACCTTCACAGTTTTC
AATGCAAGGCTAAAGTGTCCATATGATATGCAGGTGACATATCAAGCGGATCTGTTTCTGGATAAGAACAAA
GATTATGTGGTTGCAGAACATCAGGTTCTGTTAACAGCCTCAAAATGTACTTTTGTGGCGGGTTTGTTTCCTC
CTCTACCTGAAGAATCATCGAAATCGTCCAAATTCTCCTCCATAGGGTCTCGTTTTAAGGTTAATTCATCTTTAT
CCATCAAGTATTTTGGTTAATGTAGATTTAGAAACAATTGAAAGAACATGCTTCACCAATCCCAGAAAAATG
TCTCTTCTTTTTCTTTATAGCTTAATTAGATGTATGGATTTCTATTATCTGGGTTTTCTATTGTGTGAGTTATCTTT
CTCTTAATAACTTGATATTAATTACCAGGCGTAACATCTGAGTCATCACAAAGGTTCTTGAAGATGAAAATAAT
TAATGGATCAGTTGTATATGAAAAAATAAAGACTAATCTATATGTCTAAGAGAAGCTTCTTACAAATAAATGTC
TTATGAGGAGCTCTTGCGTTTAGTTTTCATTGTATACTAGGAGCTTCAGATTTGTCCAAGAATCATGTGGAGT
AATAGAATAATGTATTGCTTCATGTAACTGAATGAGTTGTGCGTTACAATGGAATTTCTGAAGTTTTTGCTATC
CATTCTTATTCAATTGTATGCCTAGTAAATTCTAGTTAGAGTAAGTCTTGCCTTTGTCTTGTTAGGCCCATGTAT
CATTTGACTGATTGGTATGCTACTTTTTACCCGTAGCACGTTGTGGATGTTAATGCTTGGACCTCCATCTCCAC
TTCTTTTATCACAATATTAATTTTGATTGTGTTCTTTTTGTTGCAATATTGCTTTTATCATCTCAAAAGATTCATAT
TTCTGCTATTGTGTTGTGTTTTAGTGCTTCTGTGTTTGTTTTCTCCGTCTCTCTTTCTAGCAAAAATGGTTCATT
ACAGCATATGTGAACTTCTAGTATATGTTTTTATAGTGCTTGTGTTTGTTTTTTTGTTTTCTCCGTCTCTCGTTCT
AGCAAAAATGGTTCATTACAGCATATGTGAACTTCTAGTATATGTTTTTTATAATGATACTTTTCTGATTGCTGA
AGCCTTTTCTTACATGCTTGCAGCTACAACTGCAATCTTTAATGGAAACATTAAGTTCAACAGAGCCTCACTA

CATCAGATGTGTGAAGCCTAATAATGCCCTTAAGCCTTGTATCTTTGAGAATCTGAATGTGATCCAGCAATTGC
GATGTGGTGTAAGTGTCTATTGATGCACATCCTTGTACCTGCGATTGTAATGAATTTATTACTTCTTCTTGCCCT
TCACATAAGTTCTTTTTATTCTAGGGTGTCTTAGAAGCTATCAGAATCAGTTGTGCTGGATATCCTACTAGACG
TACATTTTATGAGTTTCTTCTTAGATTTGGTGTTCTTGCTCCAGAAGTTTTAGCTGGAAGGTAAGTTTCCAGA
CTCATTGTGTGTCGATTCTAACTGAAACTTTTATTCTCATATGTGAATTTCCTTCTTTCCTATCAGTTTCTTTATG
ATTTTTCTGATGATCTATAATGATCCCTTGTCAGCTATGATGACAAAGTTGCATGCCAGATGATTCTAGACAAG
AAGGGACTTAAGGGTTATCAGGTAAATTTTTGATATTGTGCTGAATCAAATGTTCTCATCTTCCAACTAGAGT
AGGTTGGTTATTGAACTGCTGAGGGAATATATGAGCTAGGGAAGTATTAGACAAGTTCTTTTATATACCTTTC
GTGGCATCATGAAATCTGCATAGAGATTTTGGTGTCTTTACATTATCTGAATGCCCGTCTTTACAGATGGGAA
AGACAAAGGTCTTTTTGCGGGCTGGACAGATGGCTGAGCTCGATGCTCGGAGAGCTGAGGTACTTGGAAA
TGCAGCAAAAATTATTCAAAGACAAATCCGTACATATATTATGCGAAAAGAATTTGTTTCTCTGCGTCAAGCT
GCTATTCAGTTGCAATCATGTTGGCGAGGTATGTTTCAATCAGTCATTTGAAAGCCTACAATTAGGTCACTGA
AACTTCTTTCATCTGCTCCCTACTTCACTGAGAAGAATGTTTTGGGACTGGCCCATGGAGGAGTAGCTGTAG
CTTCCCTTTACATTGTTTGACTTTGATTTTTAAACATACATTTAGCCTAATATGGTGCTTTGAGGCCAACTTTAA
GGAATGTTTCTAGTATTATATGATTTATGATGCTTGTGTATCCCCATGGTGCTTTATTCAAGTTTCAAAAGCTTC
GGTTCAATTTACATATTTACATATTCATGCACATTATTATTTATTTTTTATTAGAAATAAATTATTGATGATGTGGG
GGGGAATACCCTGGATAGCCAGAAGTTTTAAAGAAGTAGATGAAAATATGGTTGGTTACGATAGACACCAG
CCATCTTATACTAACGGGAAGCTCATGGGTGCACCAAAAGTGAACTGAAGCCAATAGAAACATCCTAGTCTG
CACAAAGTCGTATTCCACCTCTTCAAAAGCTTTACAGCTCTAATTTGGTATGGTCAATACAATGAAAAAAGGA
AAAAAGCTCTACAGCTCTAATTTGGTTAAGTCAATACAAAATTGAGCTGGCATTGAGAAGTCAACTGGTTTC
AAGAGTTCTCCTGACCACAAAACTTGAAAACATATGTCAATTGCTCCAATTTATGATTAGAAATGCATATTAGG
TTTCAACTTTTCTCTGCTAGTCTTGTTTGAGGACAATTACTTACTCTCTTATCAGCTGACTTGTCAATGTTTCTT
CTTATAACTATTAACACCGTGTGTACTATTTTCTTGGTCATAATGTCAGCTATGCTGTCCTGCAAACTGTATGAA
CAACTGAGACGTGAAGCAGCTGCTCTGAAGATTCAAAAGAATTTCAGATGTCATGTTGCACACATAACATAT
ACAACGCTGCATTCTTCTGCAATTATGTTGCAAACAGGCATGAGAGCCATGGTTGCTCGGAATGACTTTAGA
TTCCGGAAACACACTAAAGCTGCAATTAAAATACAGGAATTTATCTCTCTACTGCTATTACAATGCTTGTGATC
TTCCCTATCATATATGCAGAAGATACATGAAGTCTTGAAATGTTGAAAGAGTATAATATCTGATACATGATTTGC
TTACTATCCTGCAGGCTCATGCACGTGGCCATGCCGCTTATTCTTATTACAGAAGTCTTCAGAGAGCTGCGAT
CATTACTCAGTGTGGTTGGAGGCGACGGGTCGCCAGGAAGGAGCTTCGAAATCTCAAAATGGTTTGTTATG
CATTTCTGTGATCATGTCTTGTAGTAATGTCTTCATATCATCTGGTTCAGAGAAGGAAGATCATTGTTAATGAG
AGATTACTTATACAGGCTGCAAGGGAAACAGGTGCTCTCAAAGAAGCCAAGGACAAGCTCGAAAAGAAAG
TGGAAGAACTTACATGGCGGTTGCAATTTGAGAAACGACTCAGGGTAAGTTACTCTTATTTCTAGGGAAGTT
TCTCCTTGGTTCATTTCCTAGATTCTGACACCATTGTTCTTTGGATTAATACAGGCTGAGCTGGAGGAGACTA
AAGCCCAAGAAGTTACAAAGCTACAGGAGGCACTGCATACAATGCAAAAGCAAGTAGAAGAAGCAAATGC
TAAAGTTGTCCAAGAGCGGGAGGCAGCACGGAGAGCAATTGAAGAAGCACCTCCAGTCATCAAGGAGAC
CCCAGTTATAGTTCAAGACACTGAAAAAATAAATGCCCTGTCAGCTGAAGTAGATAATTTGAAGGTATGGTTC
CACTACCCCAAGGTTTATTATCTCTATAGTTGGACGCAAACCTTAATAAAAACTGCCAACCAATTCTTTATTTCC
TTGTGAGTTAGAGGACATATGAATATGAATCTGTCCTCTAACCAAATTAGCCAGGAAGTTCAAGTTAGAGGG
CATATGAATATGAAATAGCATATGATGTTGGTTATTGCAAATAAAACCCCCTTGCCAAACATCCAATTCTACATA
AAGTTTGGATTTCATAGAGAAGTATTGCCCCTGCGCTTTGATAATTAGGGTGCTACAAGGGTTTGAACAACT
TTAGGATGGACTTCACCTGTTTTATTTAAAGGGAAAGAGGAAGCCCCAGACGTCAATTCTTTGATGACGACC
AAAAATTGAGTGCTAGTACATCTGAGACTTCTTAATTGGCTTGTTGTAAATAGACCAACGGTTATGGAACAAA
GGATTATCTTGGACCTAGACCAAGATATTGATGATGATAGCCAGGAGAGGAAGGAAGCTAGTTAGTCATAGC
TCAAGACTGATCGATGTATGGAAATCCTATGGAACATATAAGTCTTATTGTGCTAATGGCTATAGCAGGATTGC

TAGACAAAAGCCTGGTATTGAAGTAAAAAAAGGTAGATAGGTGGGCCCATTATTCACGGACTTCAAACCATG
CACCACCAGCCCTGAAGGATTTCAGTTATGAAAAAAAAAGATAGTTGGATTGGGAGCGCTACAACGTACAA
TTCCTGTCACTGCATTTATTTGATCCATTTTACTGGATAAACACTGTTACAAGTGCATATACTTGACTTTGATGT
TGCAAAATATTCTGTATCATGGTTTCTGCAAGTGTACTCCTTAAGTTCATTCTCCACCTTGAAGTTGCTTCTGA
GCTGGCCTAGGAGGAGGAATTAACCTCAAAAGATTCCAATTCATGTGCTAAATAATAAACCAATAATAAAAA
GTTGATCTTTAATCGGTTGCTATTTCGTATAAAAAAGTTGTTTCATGAACTATCCAGGCTTTGCTGGCATCCGA
AAAGAAAGCTACAGAAGAGGCTAGAGATTCTTCCAGGGATGCAGAGGCCAAAAACACAGAGCTGGCTAGC
AAACTAGAAACTGCTGAGCGAAAAGTAGATCAGCTTCAAGATTCTGTGCAGAGGTTTGTAAATTAATATTGA
CTCTCTGTCTATGACGGAGTCAACTTATTCACCTCTCTTTATGGGTTTATTGTTGTTAGTCTGACCTTGTAATCA
AGAGTCTTTTGATCAAACATACAGAGCAAAAGTAGCACTGATTTCCCACGATTCATGTCTTTTTTTATTTTTGA
TTGAACCTAAATAAGGTTATGGGCATTGATCTTAAGATTTCTAGCTATGAACAACCAGCATGCAGTATAATAGT
AGATTTGCATCCTTAAATCATCACGTTTTGTGAGCTGGACTTCCCAAACATAGGATTTTTCATTCTTAAAGAAT
GTAAAGACATTCTGACGCTCTCTTTCATCTACTGCCTCTTTGTTTGTCTCTTGTTTCAGGCTTGAAGAGAAGC
TTTCCAATATGGAATCAGAGAACCAAGTGCTTCGACAACAAGCTTTGACCATGTCACCAACTGGAAAAGCTT
TATCTGCACGGCCAAAGACTACCATCATACAGGTGCACAACCAGTTGCCAATTCTCATCTTCTTAGATTTTTTT
TTGAGGGAGTAGATTTTCATATTAGCAGCAGGTGGTATAGATAATTACTCTCTTCATGAAATTGCAGAGGACT
CCGGAGAATGGAAATGTTATAAATGGAGAATCAAAACCTAATTCTGTAAGTTCAATAGTTGCAGATTCAGTGT
TTACTATCATTTTTTAATGTAAATTAAACGTCTGCCTCTTTTGATGTATATGCTTGTTTATATGGCATAGGATATG
AGTCTTGTTGTAGCAAGTCCAAAGGAGCCTTCATCTGAAGAGAAACCACAGAAGTCTCTAAATGAAAAGCA
GCAGGTAAGTGGTCCACTGGCTTGCACAAAATATTACCTGATCAATTATGCTTGTTTAATCACACTGTGACATC
TAAACTTCGGAAATCGTTTTTGTACGATTTATCCTTTTTCACAGGAGAACCAAGACATGCTCATTAAGTGCATT
TCTCAAGATTTGGGCTTTTCTGGAGGCAAACCAATTGCAGCTTGTCTCATATACAAATGTCTGCTCCACTGGA
GGTCCTTTGAAGTGGAAAGAACTAGTGTTTTTGACCGTATAATACAAACCATTGCTTCAGCCATAGAGGTAG
AAAGCTTATTACTTTGGTAATGTTATATCATTCCAGTTTTCCTGAACTTAATGTTTATAAATCTAAGGCAATTAAT
TTCTGAAACTTCTTCATTAAAGGTCCCAGATAATAATGATGTATTAGCCTACTGGTTATGCAATACGTCCACATT
GTTGATGCTGCTTCAACAAACACTTAAAGCTAGTGGGGCTGCTAATTTGACTCCGCAGAGGCGGAGATCCA
GTTCAGCCTCTTTGTTTGGAAGGATGTCCCAAGTATGAGCTCTATGGTTTATATTAAGTTCTCTGGTTTCAGCT
GGTTGCCCAATTTCTAAGTCATATCATAGTTAATACTTCTCCTCTGCTATCTCTAATGTTTATGTAGGGCTTGCG
AGGTTCTCCTCAGAGTGCTGGGCTTTCAGTTCTCAATGGGCGTATGCTTGGGAGATTGGATGACTTACGTCA
TGTTGAGGCCAAGTATCCTGCGCTGCTGTTCAAGCAGCAACTGACTGCCTTTTTGGAGAAAATATATGGAAT
GATAAGAGACAATCTGAAGAAAGAGATCTCCCCATTGCTTGGGCTATGTATTCAGGTGCATTACTTTTTATGA
GCTAGTATTATTTATGCCACAGGTCCCAGAAATATTATGGAATAGAATGTTTACTGCTTGATTTGTAGTTTCAG
CTAGTCCATATGCTTACTGTAGAATTGTTATGATAACTTGGGGTTTTTAGCACTTGGGTTGGTTGATTACCACT
GAGTAGTTCTCTGATGCTTGAACTATTCAATGTGTTAAAGCAGTACAATCTTATAGATTGATTGTCACTTAAGA
AGTTAATCGGTATTGTAAATTATGCCACGAGCAAGCTCTTGAATGTTTCTGCCTGGTCAAGCCAGGAGTTCCT
CTTATCTATTAAAATGACTGTAAAATAAATCAGACAAATCCAATGACTAGTGATGATACTTCTCTTCCAGCCCA
AGGTGTTGGAACGAGTGGATCTTTCTAGGTTTAGTACTGTGCCTTGAAGCTGAGCAATTTATAGAGAGACCA
CCTTTTCTCTTTGGGACACTGTTACCTTTTCTATTTTGTTCTCTCCCCATTGTAATTATTTTTTCTGCCAAGAGA
AAGAGGGAAGGAGTTAATTTTGTCAGATCATTATCTACATAATGTATGCAGGCACCAAGAACATCTCGTGCAA
GTTTAATCAAAGGAAGATCCCAAGCTAATGCTGCTGCCCAGCAAGCTCTATTTGCTCATTGGCAAAGCATTGT
AAAAAGTTTGAACAACTACTTGATGATGATGAAATCAAACCATGTAAGTTAAAGATTGAAGTGTTAAATAATA
GCAACTGTCATTTTGTCTCCTTGATGTTTATATGTTTCCATTGCTCCACCTATGTAGGTTCCTCCCTTCTTAGTTC
GGAAGGTTTTCACTCAAATATTTTCCTTTATCAATGTTCAACTTTTCAACAGGTAAATAGTTGTATGTTTTTACA
TTAAATAGATTTCATTCGGTACATTGTCACCTTCTTATGTATCTGTGTGTTAACCTTCTTTACATTCAGTCTTCTT

TTGAGGCGTGAGTGTTGCTCATTTAGTAATGGAGAGTTCGTGAAAGCCGGGTTGGCTGAATTGGAACAGTG
GTGCTGCTATGCAACTGAAGAAGTAAGCATGTTCTACTGTATCTTTTCAGTTCTTAGTGCTATTCAGCATCTTA
TTTCTGTATTTTCATGTCCAGTTTGTAGGCTCAGCATGGGACGAGTTGAAGCACATTAGACAGGCAGTTGGA
TTCCTAGTAAGAAACTGATTTCGGGGCTATGTGCTAAATGAATTTTTGTATGATGTGGTGCTTTTATCTGACTT
TTTTTTTCTCTCAACTTCTCGCAGGTTATACATCAAAAGCCCAAAAAGTCATTGAATGAAATCACTAATGAACT
TTGTCCAGTAAGGAGTCATTTTTCTATACTTCTCCATTCTTCCCATTTTTTCTGCATAAAATTTGCTTCTTTCTTT
TACCTATGAAAAGAAAAAAAAAAACCTTTATTTCTTTTGACTTCTCGCATGCTGCTTTAATTTATATTATTCTTTT
ATTAGGTGCTTAGCATACAGCAACTGTATAGGATCAGCACTATGTACTGGGATGACAAATACGGAACCCACAC
TGTTTCTTCAGATGTAAGTTCTTGCACGGTTGCACCTACAGTTTGGATGCCTATGTAGGTTTACAGTAGTTTTT
GGTCTTGTTATTAAACAGTTCGCTTGGTTCCTGATCATGGGGTTTTATGCTTTACCTTCAACTGATAGGTTATT
TCAAGTATGAGAGTTATGATGACAGAGGACTCCAATAATGCTGTCAGCAGTTCCTTTTTGTTGGATGATGATT
CGAGGTATCGTTTATTAAATATCTCATCCTCAATCTTTGGTATACATGTACATTTTATTGAGCACCTCAAATTCAA
TATCTTGTCTTAAATTATGACGCAGCATTCCATTCTCTGTGGATGACATCTCCAAAACGATGCAACAAATAGAC
ATTGGCGATGTTGAACCTCCTCCATTAATCCGTGAGAATTCAGGATTTGTATTCTTACATCAACGGTCTAGTTG
ATGGTGGTTCTAAATATACAATTCAACATGGAGATTGATGAATGTTTGCATAGCTCCGGAGAGCTGACGCTCC
TGCACATCAAGGAACATGCAGAACTTGTGCATACATTGATTGTTGCATTTAATGGCTAGTGTATATTTTTGTCC
ACTTGCTTTTGGACAATCGTAGCTGTTTGTTGTCCGGTTCAATTTATTCTTTTTTCCTCTCCTGCTCAACTTGG
GAGGTATTTTTATTATGCCAAAAGGCTTCTAGTGTGGTCATATGCAGGTATAGGCAGATGCATTGGTTTTTGTT
GCTTGCCTTTATTTTTTTGTCCTTGTCAGAGTTGTAAAGTCACTAAAGTTGTCAATTTTAGGTCCTTATTTGCT
GTAGTGTGTAGGTGAAGTGCAGTTGAAGGTTGTGTTGTATAAGAAGTGTTAAAATTATTAAGGCAAAAAAGT
TTGTTAGTCTTTTCTTCCCATTAGTGACTAGTTGATCTTTCATGATTTTG
>PGSC0003DMG400010897
TTCCCACTTTTATGTTTTGATATAGTTGATCAGTACAAACGAAAGGAGGTTCATGCATTATGGCTAGATTCTCT
TACCACTATAGAGCATGACAAAGTATATATTTTTCAAAAATTGCTCAACTATATGTATAACTGTCCATTGCTCCAT
CATAGTATGCCCCAATGGTTGCACGCGATTTCTGGCGCCGTCTCCTCCGTCTGTGAGTGCAGTTTACTGACTG
TTCAGTTACAACCTGGAAAAGAGTTGGAATTGACAAACAAACACGAGTCATGGACCAAATAGAAACCCCTC
CTTTTTCGAGAAAAATGTAGTTACAGAGAGAAGAAGCACTAGTAGAACAAATTCAATCGGTTAAAAACCAA
ACGATGAAGCTAGAAATCTAAACTGAGATTCACAGTCACATTTCCATTTGTAGCTGAGATGAGTCTACATTGC
TTGAACATTGTATGAGCTTGATCAATGCAGTCATACGCACCTTGATGCTCACACAAATCAAAGATGTGTCTGC
AAGTTCCTAGGACTGAATTCTGATTAAGGGCCATTAGTCTGCACGTGGCAATAAGAAACTGAAGCCTGAGTT
TTCTCGAATAAGTGGGGGTGGTTCAATGTCTGCAATATCAATCTGATCCATTGATTTAGACAGGTCATCAATTG
AGAATGGTATGCTGCACCATGATCAACAAGGGCGATCAGTGATAAAGTGAAAGTACATTCTAAGGAAGTGG
TGCACAAAAATAGGTAATGGCAAGTTTAGGATACTGGAATTGTATATCCAACCTCGAATCATCGTCAAGCAAA
AAAGAATTGCTGACTGCATTGTTAGAGTCTTCAGTCATTAATACACGCATATTGGCTATAACCTGTCAAATAAT
CGACAAACTCTACTCATTACTTGCCAGCCAGCAGAGGACAAAAGTAAAGAGTGTTTTGGGCAGATCAAAGA
TGCAGGAGACAGTGTTCTAAGCTGTATAAGCCACTCCCAGCAAGAAATAATGAATTCATGCTGTAGTGAAGC
AGGGATTGAGGACATGCTATTGAAAAATTGTTGTATCTAACTTGAGAATGACCATAAAATCAATATGCACTAC
ATGACGCAAAGGAACATCAAAATTATAGATTATCTGGTATCGAAATAACATCTACAATTTTTTCTGGACAAAA
GTTATGGCTTACATCTGATGAAAGGCTATGTGTGCCATATTTGTCGTCCCAATACATTGTGCTGATTCTGTAAA
GTTGTTGAATGCTAAGCACCTGTTCATGATTTACAACTTAAATGAAAATGATAATGTATGGGCTCAAGAGATA
GTTTTCGGAATATGTAATAGTCCAGTACTTACAGGACATAGATCATGGCTTATTTCATCCAACGTCTTCTTTGG
CTTCTGGTGTATGACCTAGTATAGTCCAAAAGGAGCACACTTAAGGATCACTGCTGATAAAAAATGGACAAA
GCTCATTCCAAATTCTTTGGAACTACAGTATTTAGGTTTGTGCACTCAAATTCCGTGACACACAATCATGATTA
TATTGCAAAGGAATAATAAGTATGTAAAGAGATTTCGTTTAACACTGCAAGGACAGAAAATTGCTATTCTAAT

TTCCTTCTATTTTATATTACTAGGAAAAATATTGCTGTCGTTTCTTTTCCTGAAAAATATGCACCAGAGGATGTG
TCCTTTGCCTTTGCCTTTCAGTTTACTATGCAGTTGTATGTTTAATCCAAATTAGAAGATGCATTACCAAGAATC
CAATTGCCTGTCTTATATGCTTGAGCTCCTCCCAAGCTAATCCTGTATACTGAACAAGCTCCCTTCATCAGTGA
AAACAAAAGACACGGTGAATAGCGTTTAACAAGTGGCAGGAGACCAAGGTACCTCATCAGTTGCTTTGTAG
CACCAGTGCTCCAGTTCCGCCAAGCCAGTTTTGACATATTCACCGTTGCTAAATGAACAGCATTCTCTTCTCA
GCAAAAGGCTACAGCAAGGAGGCACAAATAAAGAAAATAGGGTTTATTATATAGTTGAATCTTGAAGCTATA
AGAATAATATGAGATTCCCTTGACAACAGACCTGTTAAATAATTGGACATTGATGAAAGAAAAGACTTGTGTA
AATACTTTGCGCACAAGAAATGGAGGCACCTGAAAAAAAAGAGAGAAAATAACCCTACTATCATGAGGTAA
CCAATATACACTTCTTTTCATAAATTTATGACTTAATTATCTTGAGACTGTTCTGCCTCAATGCATCAATTACTTA
ACAGAAGATCACAATCTTCAGAAAAGTTGGGCCGTTCTTCCTTCAGCTAGAATATATTACTTATATTACACGAA
AGTAGCTTTTTATTAACTACTACGCTAAACATTGTACTGACAGCCTGTCAATGTGATTAGAGTTCCATAAGATC
TTTCACTCTAGAGCAAAGCAGTAATCTGTCAAATTAGCTATATTTTCAAAAGAGAACTAGTAACCACAGGTGA
GTTGAATCAAGTTATAAATTGCATCTCATATCCTATATCATATTAGCCCTTTTGATAAGAAAATGATGAGAGCTA
CCCAACACCATTGCTTCGCGAACTCTCTTAAGTTGGACTGTAGAGGATAGTGGAGCTCAGGATTTGTGTAGT
TTCAGTTCAACCGACAAGAACTAGGAGAGCAAGCATTTTCACTATTTATACTTGTAATCTGATTAAGATCAGA
GCAGATCAGGTATTCATCCTTATATCAAAGTAAGAATCATAAATTCCAAAAATTGTCTGCAAAACCACTTCAGT
CCTATTGCTATAGCTACTTTTGGCTCTGATCTCTTTCTCTTCTTTGTCCTGTTCACTCAAGTCCAATATTCAGATG
TGAAAAGATCAGATACCATTCATATCACTTTTATAAACTTCTTAAAGTAAAACCACATGCACCTAAGAACTCCA
CCATGATAGTTTTCCTAAACTTTTTTGCTAGTAAGAGCCTCATCTCTAGCAATTGTCAAAATGTCTTAAATACTC
CCTCGTCCACTTTTACTTGTCCACTATACTAAAAATAGATGTCCACTTTTACTTGTTCAGTTTGGAAAACCAAG
AGATAATTTACCATTTTATACCTATTTTACCCTTCTTATCAAGTACTATTCATCTTCCAACTTAAAATAATTAGGG
GTGATATATTAAAATTACCCTTTTCTTTATTGCTTCTTAAGGGTGTGCCAAGTCAAACATGAACAAGTAAACAT
GGATGGAGGGAGTACTAAAGATATAGGTGGATACTGGAGCTCAAGAACAATAACAACTTCCACAGTACTGG
AAACTAGCTTTTTCATTGGATTTGCGAGCAAAAGGTGATATACGTTATAGTTCAGGTATTAGCAAGATATTGT
GGAAAGTTCCTAAAAAAGTTGTGTAAATCTCTCACTACCGTGTGAGTTATTCCTTCCACCTATATGAGGGATG
CTCTGCTTGTTGGTGAAAAGAAGGGTAGAGAAAAAGAAAATTCATCAAAAGACAAAACATGCTCCAGAAA
GGACTATGTGAAGATGTGTATCCATGAGAGAAGAAAGTTACATTTATTTTGCTAAACTAGAGCAGAAGAGTT
ATTGGAAAAAGATGATATATAATCATAAAGATCATTCCATGGGTTGATGATATCAACACATTTTATTTATAAATA
GGTTCAACAGCAAGTTATCTTTCATATCATGCAGAAGACTGTTAATTCACCTTCCAAGTTCAAAGCAAGTGAA
TGGGAGGAAAGCATAAAAGGAAAGCATGAAAAAAAAAAAAAAGCAATATGTTTCTGTGACTCTTACATGATT
GGCTTTCAATAAGTTCAAAAAGTTAGCAAGGCTCTTGACAATTCCTTGCCAGTGAGCGATCAAAATTTCCTG
AGCAGCTGCATTTGCAAGTGTACGTGCTGTTGTCCCTTTAAGTAAACTTGCTCTGGATATTCTTGGTGCCTAA
ACCATAGATTGCAACATGTAAAAAGGCTTTGGTACACATAGAATCTAATAATATCTACTATTTCAAAACAAATAT
TTAGATAGATCTATGACAATATCTTGAACCTGAATGCACAACCCCAGCAGAGGAGAAATCTCTTTCTTAAGAT
TATCACGGATCATTCCGTAAATCTTTTCTACATATGCTGTTAGCTGCTGTTTAAACAACAGAGCAGGATATTTG
GCCTCTACTTGGCGTAAGGTATCCACTCCACCGGCTGAATCACCATCAATGAGAGAAATATTGACACCTTGAG
GGGTTCCACGAAAGCTCTATAAATTGTTTAATCAGCATGAAGTGTTACACAAATGATAAATATCACTGTTAAG
ATGAACTTTTTTCACCTGTGTCATTCTCCCAAATAGAGAGGCTGATGATGATCGCCTGTGCTGTGGGGTCATC
CCAGCAGCACCACCAGCTTTTAATGTACGTTGCAGCAGCAGCAATAGAGTGGATGCATTGGACAGCCAATA
GGCTAGCATATCATTGTTGTCCTGAGTCTACAATGTTAAAAAGCTAGTTTTTAAGTGAAAAAGAGACATTTAT
TTTATTGATCTGCATCTTTCGCAGTGAAATTGTATAACTATATTAATACTGACCTCAATAGCTTGGCCAATGGTT
TGTATTACCCTATCAAAGATACTGGTCCTCTCTACTTCAAATGATCGCCACTGCCTAAGGCACTTGTAGATAAT
GCAGGCAGCAACAGGTCTGCCTTTAGAGAAGCCAAGGTGCTGTGCAATACACCGGATGATCAAGTCCTGAT
ACTCCTGTTGTTTATCATTAAGTGATTTCTGTGGTCTTCCCTCCACCTCAGCACTCTCCCTTGAAAATGAAGTG

CTATGCAGATCCTTTTAAAAAAGCAATTGTTAGTCAACTAACAAAAGAGGTCAGAAATATACTAAAAAGACA
ACTAATATGCAAGCCTTTGACTTACTACGCTATTCCTTGTGCTGCTCTCATTCCTCTGCCAAAATCATAGAACA
GGGAAAAAATTAGTTATAGATTTAACATCCAATATGAGTGAAGGTTTTCAGGATTACCTGAATACTTGATCTTG
ACCGTCCCGACAGCAATTTATTGTTCTGTGCCATTGTCAGAGCCTGCTGGCGAAGTACTTTATTTTCTGACTC
TATGTTGGTGAGCTTTTCTTCGAGCCTATTGGTAAGAAGGACAAATATTTTCATCAACTAAATCCTGAAAATAT
CCAAGGTACTCCTAGATATCCATTTTATATACAAGGATATAACTGACAGACAAGAATTGGTCTATTCTTTAATTT
TAATTGCAATATTCCAAACAAGTATTCAAACCAGGTATAGATCCAGAGCTATCCTTTCCTCTACATTAGTTAGA
GGTGACTATGACAAGCATTAGTCACTAAAAGAGGACCATTAACTTATGGATCCTCCTGAGCACAGCTTCAGC
AATTACCTTCAAGTCACAGGACAAATTAAAATGGTGTATGGACATCTATAAAAATATAAACCCATTGTATGAAC
AACTCATCTAAAGGCTTGAACTGAGAGAGGATACACATTTATTTATTTATATCATTTGAAGCACTCCCCCACAT
CTGAGCCCCTTTTTTTTGGGCTCTCAAGAAAGTAGAAATATTTTGTTGATGGATGGTGGTGGAGCTTGAACC
CACGTCCTCTGCCTGCATTGATACCATGTGATATTGGATGAACTGACTGCTCTAACAGCTTAAGCTATTAGAGA
GGGGATACTTTTATTTATTTGTAACTCATAAAGAGGCATTGCTAGCTATTATAGGATGTCAAACTTCTTGGTGA
AGCCTCTATGACTGTCTCTCATCCTGAATCATTTCAGCAGCTAATTTGATTGAGACCATACCGTCCCTATCATGA
AAGAGGTAGAAAAACAACTTGGTATTTAATTCAGGACACTACTCTCGAGTTTGAATCTTAGCTTTAGATCAAT
TAGAAGAATAAACAGAAGGATATCCCCCCTTCACAAGTAGATTTTGAAAAAGGAAATCTGTTACTTGTCATG
AATGACCCAGCATTTGTTAGTGCCGGTCATGGCCCATTTGCTGGGTTGTGACGTTTCTGTATTTTAGTTATTTC
TTTTGGATTCTTGTACAAACTTTTTGTAATTTTATTGACTCAAAAGGTTGGTAATCTCTATTCTTACAATAGATA
AGGAATAAAACAAGTATTAATTTATTCCCACCTGCTCATTGATTCCTGAAATTGTTGAACTTTTCTTTCAGTTTC
TTCCAACTTCTTGTGCTTTTCTTCACTTGATTCTTGAGATTCAGCACATTTCCTTTCAGAATCATCAGCACGTT
GCTTTTCAGATTGCAATAACACCTATATAAAGGTATCAGCATGAAAACATCAACCATATGTTAGTTGAAAAAA
AATGAACTTCCACACAAAGTCTGTATTCTGCTCTCCTTTGTTCTTTGGTGGGGAAACACCAGGTTGAGAGCA
CTTGGAGTTAACTTTATGAGTTCAGTGTCGGTACCTTTAAATTTTCCACTTCTGCATTTAAAGCATCAATCTTT
TCTGTATCCTCAACAAGAACCGGCTTCTCTTCCACAATAGAGGTTGCTTCTTCAATTGCTTTCTGAGCAGTCT
CACGTTCCTTGATGAGCAGTGCATTCGTTTGATCCACTTTGCTATGCACATCTTCCAAGGTATTCTTCAGCTTT
GCTATTTCCTGGCTTTTTGCCTCTTCCAAGTCCGTCTGTGAAATAATGCATCAAACAGAGTCCCTTTCTCAAAA
TGGACAACTTATCATAATCCACAAGGTCAAAAGTGGCAATAATATTTAGCTTTCAAGTAATATCTTGTTCCCCA
CTGATTTTCAGGTTAATTCTCTCCTTGATTTACATATCATGCAGGAAAAGGAAATGCATGATCTGCTTAATTCA
AGTGTATTACCCTTAGACGTTTCTCCAACTGCAGACGCCATGTAAGTTCTTCAACCTGTTTTTCAAGCTTATCC
TTTGCTTCTTTTAGTGCACCTGTTTCTCTTGAAGCCTGAAGATATTGGAAATGTGTGTTAGAGATCGACAAGC
AATGAGAATATTAGCAATAGACTATGAGATACGGATAGACCAAAAATAAGATTTTACTCTATCAATAGCAAACA
ACAAATCTGATACAAATACAAGGGATACTATATGGATAACCACAATATGTGAAACAAGAAGGTTTAAATCTGT
AATTAGACAAAGGACTTCAGAGTTCATCACTCAGTGGCATGTATATATTCATTGTTAGTTACATGCAGATTGCA
TGCTTTTGATTCGACTTTGAATGTTCAGAAGATTAAATTGCTGATCCTGTTTTTATTGCTTTGCTCGAGAATTT
CATCCATGTATGAGTTTTACCAACAGGAAGCTTCATAATGCTCATCTCTTTCCAATAAAGGTTCAGAGGAGCA
ATTAAGTGATCCATCATGCTGCGGAAGAACTTTAGCCTTTCGTTATGCAAATAACAGCAAACAAGGTGAATTA
TGCACCATGTAAGTAAAGGACTTAGCTAGCAGATTATGTACTTAGGTCACAGAGCATCATCTCAGTGCAAATC
AAAGGAAATTGAAAATGTGATACAGGATAGACGGGATAAAGGTCCAAAAACTACCATCTTTAGTTTCCGAAG
CTCTTTCTTTGCAACCCTTCCCCTCCATCTGCATTGTGTTACAATTGATGCAATTATGAGTTTCTTGTAGTACGA
AAAGGCTCTGTGACCATGCCAATGTGCCTGCAAGTAACTTTTCTAGTTAATAGCGAGAACAAAAAACTTGGT
CAACCCTCTGAATCAATAGAAAACAAGTAAACAAACTTGTATATTAATTGCTGCTTTGGTTTGTCTTTTATATCT
GAATTCCTTTCGTGCAGCTGTCGCTCGAATTCCTGTCTGAAGAGCAATGACATTGATTTTGAGTCCTGTGTAG
GACTTTCGGGCCAAGTGTCCACGCAATTTTGTCTGGATTTTGATAGAAGCAGCTTCTTTTTTCATATTGTCGA
ACACTTTGCAAGCAA

>PGSC0003DMG400003722
ATGAGGTACAATTTCCAACAAATATAGAATATCTACTACAACACTTACAAGACTGGTTAATCAGTGAGTGAAA
GAAGAAGAAGAAGAAAATGGGAAAATCTACAGCTTCTTCTTGCTTAAAGATCATTGCTTGTGGCAGTGATTC
TGTTGACCGTGATGAACTTGAAGCTCATCCTGAGGTGAAAAATCTACAACCCCTTTTTCTCAGATCAAATTTT
CATTTAGTATATTCTGTTTTTTTCCATTTCAAAGATGGTAATGTGTGTTAAAAATTGAATCTTTTTTGTGAGAAA
ATGGTTTTGAAGTGAATCTTGGATCAACCCATCAAAATGTGGAGTCTGATCTTTTGTGGGGTTTTTTTTGTTT
TTACTAATTTGTTATGTTTTTGAGATGATGGAGTTGAGCTGATTTGGTTCTTGAATAAAGGAGGTTTTTTTTTT
GTGGAAGGTATGTAGAAAAGTTTTGAACTTTGTCACTGGATCTCTATTAGGGGATGTAAGATTGTGAAATGT
GGACTGATTTGTGGTTTGGTTGAGAACTTGTTAGATGTGTGCACATCAATGTTAAATGAGAGTGTTTGAGAT
ATTAAATTGTTCAATTGTTCATGTTTGTTAGTTAAATAATTGCCAACTTCAAAGGAAAGGCTTGATTTTGATCA
GTTGATATAAAATCATTGACACCATGAAGTTGTGAGTGTACTGTATGGATTCAATGTGTTTCGAAATGGAAAT
GAATTGGATCACTATTGTAAGTGGATTTCTTTTGTTTAGATGGGAATGAAAAAATAGTGATGCAATTTACCTA
GAAAAGAAAATTGATTCAATTTGTGACTGCCAATGAGGGATGTGTTTTCTAAGATAAATGCTAGTGAAACGT
TTCAATTTCTGGACCAGTCAATTAACCAACGTCGCCTCAATTGCAAAAATACCATATTTGTCTTTCAGTGTTTG
TCAAAATTTTGTTTCATTAATTGAGTTGTACTTGTTCTTAGTAATGTCTTTTCAAATTTACAATGTAAGTTTAGA
TAACATCTAGTTATCATTTCAAGTTGAGTATGTCATATTTCAACGTCAAGTTAATTTTTTATGATGATGTCAACAT
GATAGTCCAATGCAATCACTACTTGTTTCCAAATGCTTTATGCCAAATATATCTTGTCTTCCTTCTTACATCATTA
TGGACTTGCTGCTGTGTAGTTTGTAATATAAGTTGAGCTGATAATTCCAGCTATGAGACTTCACATTACCTACT
ACCTGATTGGTGGAATGCTTAAGTATGGAAAAACAAATCAATTACCGATCCGCTGACATACATGTGACGAAA
CATGATTAAGAAGACAAGCTTGTGCATGATTTCATTTCTTATCAATGGTCTCTGGAGGTTAAGAGGTTGGCA
GAAAAATAATCATCATTTGTGCAAAAAATGAACAGATATGATTAAGAAGATCTTGTCGTCAATGGATTCATTTC
CCATCTGAGGCTTCTAGGCATATTAATTTTTCTTAGTAAGAAGCTTGATTTTCAGATATTCCTACTATATGCATC
ATGACTCATCTATGTATGCGTGCTACATGATTCCAGAAATGCCAATCTACAATCTGCACATCGTCCTGTCTTGTC
CCTCGTGGTCTATAGTATGGATCTAATAAAGGATATTGATCTCTTTCCATTGTTTCCCTCTTTGTATATATACTTC
ATGGGTAAAAAGAGAAATTGATATACATGTGGATGGGTGTTAAACTCAGCTACTTGCAATACAACTTTATATC
ATATTGGATGTTCAGTTAAATATCTTACACAATTCTTATTAGGAATTTTTCAGTTTCTCTTTTGCCTTTTCCATCT
CCTTTATACTTTGTCGAATGAATTATTTGATACATGTTCTTAGAGTAAAAGCTCAAGCGACAAACGTGGATGG
AGTTTCCGCAAGAAATCTGCCAGGCATCGTGTATTAAGCAATACAGTAGTTTCAGAAACACCATCTGGGAAC
AAGGACTGGCCAGAAGCTGCTAATGCCAATCTGCAAACACAATCTAACTCCACCATTCCAGAGAAGGCATCT
GTTGTCCAATGGGCAGATGAGAAACCCCAGTTTCCAACGGTCGAGAAGTCTCAGGTGTCGGCTGACGAGA
AGCCCCAGGTCTTGGCAAATGAGAACCCCCAGATCTCAGAGGATGAGAAGCCACAGGTTCTGGAAGATGA
GAAGCTCCAGGTCTCAGTGGATGAGAAGCCCCAAGTCTCCACGGACGCGAAGCCACAGCTCTTGGTGGAA
GTCTCAGTGGATGAAAAGCCCTTGATCTCCGAAAAAGTGAATCTCGAGGTCTCCGAAGATGAGAAGCCCAG
CGTCTCTTCAGATGAAAAGGCCCCCATCTCATCAGAAGAGAACTCCCTGCTCTCAGACTTGGTGGATGCAAA
ACAATCAGAGCCAGTAACAGCTAGAGTCAATGATGGTAAAGCTGATGTCATCCTGGATGAACACGCTCTTGT
TATCCAGACTGCAGTCAGAGCATTTCTGGTACCTCAATTTTGTCATCTATTCTTTCAATTAATTTGGCCATTTTA
TGGTTAAAGCTTTTGGCAATATAAATTTAATTTGTATTTGATTGTTTCTTCTCCTAGGCACGAAGAGCTCAATT
GAAGCAAAAGCATATAACTAAATTGCAAGCTGCTGTACGTGGACATTTAGTTCGCAGGCATGCTGTAGGAAC
TCTGCGATGTGTTCAAGCTATTGTCAAAATGCAAACTCTTGTTCGAGCACATCACACTAATCGCATTGCAGAA
GGATCTAGTATCAAGGAAAAGCTAAAAGTAAGTATTGGAAAGATCAAGAGTATGAAACTATATAGGCAATATT
TCTTTCTCAAGCATAAATCTGAATGTTTTTGCTCCCCTCGCTTTGGAATCCTAGATTTGACACTGAGGCACTCT
TAGCTATCATTTGAGGCATTTTTTCTTTTCCCTCTCCCCCCACCCCCCTCCTTATGGGTGTTGTTGAGCCCTTTC
AACTGATGGAGTCACATCTCTGATGTTTCTTTCTTTCAGGGAAAAGAGAACTCAGGAACAAAATCAGAGTTC
ACATACATTTCTATTTCAAAGCTACTGAGCAATAGCTTCGCTCGACAGGTATATATCAGTCATTTTCCGCTATAT

GATCTGAAATATGCGATATTCTTTTTTAACTATGTATCATTTCCCATCCAAACTGATTTGTTATTTCTCTTTTGGT
ACAAGCTCCTTGAATCAACTCCAAGGACCAAAAGTATAAATATTAAGTGTGACCCTTCCAAATCTGATTCTGC
CTGGAAATGGTTAGAGAGATGGATGTCTGTTGCATCACCAGGAAATCAACTGTCACCACAGTCAGAATTATC
TGCCGAGCAGCAGGAGAATGAACCTACCGAGCACCACAGTAACCTTATGGAAAGTAAAGTTCAGCTTGATT
CCGAGTCAATGGACTTCAGAGAGGGTGAGGAGGCATCGCTGTCTGCAGTGCCATCTGAAAGTGATGATAAT
TTGATCACTTATGATGCAGACAGCTTAGATTTTCAAGCCGACATACCGACTTTACCTCCTCAGCCTCTGAATGT
TGATGAAAAAACTTCAAGAGATGACTGTTCTATTCCTACTCAACTTAAGGAGGCCAGGGCTCTTCCTGAGAT
GGAGCCCAATTCTTTCCCTGCAAACACTGAAGTTGAGAGAGAGGATACACATTCCCTTGAACTTTCAGAGA
CCGAGAGCAAAAAGATTTACATGGATCAAGAAAGGCAAGTAATCCTGCATTTATTGCTGCTCAGTCAAAGT
TTGAGGAACTCACTTTGGCAGCTAAATCAACCAAAGTGACTAGTTTGCCCAATCATAAAACTGAAGATGAAT
CTAGTGAAGATACGTTTTCAACTATCACTGATCATTCATTTGGGGCAAGGGAAGCTGCTCCGTCAGAAAATTC
TGTTCCTCATAGTACAAGAGCTCAAGTTGGTGGTTCAGAATGTGGCACGGAGCTTTCTATTTCTTCTACCCTG
GATTCACCAGATAGGTCTGACGTCGGAGGTCATGTATTTGAGCAGGAACTTCCTTCCAATGGTGGAACTGAC
CATCGTAAGAGCAATGGATACCCTCACATTGAAGATGATAGTACAAATGACTTATCACACTCCGACTATGTTCA
GGCAGGGAGAGAGGATCCTACTGATGATGCTAAGCATGTAGATGTTATGGTCAGTTCAGATCTATCACCCGA
AGAACAGAAGCCAGAAAACAATTCAGTTAATGTTCAAATAGAGCACGAAGCTAAGACGGATCGACTATACA
AGTCATCACCAGACGCATCTCCAAGGAGCCATATAACCGTCCCTGAATCCCAAGGGACACCTTCTAGTCAAG
TGTCAGTGAATCCTAAGAAGCTAAAAAGTGAAAACAGTGGATCAATTCCCAAGCCCCGTTCTGCACCTGCTA
GCAAAAAGTCTCCTTCAAAGCTAAACCATGCTCCAGGCACAACTAGTTCGGAACAATTATCTAAGGATCATA
ATAAAAATGAGAAGCGACGAAACTCGTTTGGTTCAACAAAAGCTGGACAAGCTGATCAGGAGGCTAGAGA
TAACAGTACTAGCAGTTCTCTCCCAAGTTACATGCAAGCAACAGAATCTGCAAGAGCCAAAGTTATCCCAAA
TAGCTCCCCAAGATCTAGTCCAGATGTCCACAATAAAGATGAATATATCAAAAAGAGACACTCTCTCCCTGGT
TCAAATGGTAGGCAAGGTTCACCTCGTATCCAGCGGTCTCTGTCTAATGCACAGCAGGGTGCAAAGGGAAA
TGGAACTCAATCTCCACAGGGTATTTTCTAAACCTTCAGATGCCTTGCATAATACAATCTATTGCTATTTGTTAC
TGCAATGAGAACTGACTTGAGTTATGACCCGATACTCCAACTCATTTCCTACCTAGTACCTCCTTTCCCCCTTT
GATAGAATCAAGATAGGATCAAGTATGATATCCCGAGTCTAGTCACCTGAAAACTACTAACACTCTGCTGATAT
AGATGAGTAAAAAACCTTAGTGTTTTAAGCACTAACAAATGGTATCAACTTCCTTTTTGAATCCGGCATGTAC
GTCAATTGCAGCACTGTTTATTCACACGTCGATCACCTGACATTTATTCTTCCCTTTGCACTCTGACCTTTGTTT
CTTTTAACTACTCTTACAATGTTATATTTAGACCACTTAACTGCACTACTTTCATCGACACCAAAATTGTTGGTT
GTAAACCATGCATGTGTGTCCCCTAGAGATCCTCTTAACCTTGAGCTTTTTCGTGCAAATTTTGGCAGTTTGC
ACAGGCATTAAGCTTCATAAGGATGAAAATTCACTGATAATTTATGATGACAATTGTGGGTCACCAAGATTCT
TTAACTGCTGGACTGACTTTTCTTTTCTTCTTCTTCCAATTCTGAGCTTTGTGGAATTCTCATATGTAATATTTT
GTCGGTGTCTGATGTGTGTTTTTCAGAGAGGAAGTGGCAGAGATGAGTAGAGTTCCTCTGTGACTCAACTA
ACGGATGGCATATGACGGTGTGGCTTATTGGCAATTCCTTTGGATGCTGGTCATCTGCTCTTACGAACTCCAC
AGCAAAGCTATCCCCAGGACCATTATTTATTTGTGAAGAGAAATGTAGAAAGAGTTATTTTTCTTGTAAATTT
ACTATTTTCTCATGCCTACTCAAACCCCCCCTTTTCTTTTTCAAGGATTTTGTTTGAGATGCTTGTTCTTCTTTT
TACTTTACATCACCTTTGTGTGTATCTTTATATTGTGTGCTTTTCTGGCTGATGTATTTCTGACTGCTAATGAGA
AGTTTGGAAGAGTTGCTTTCAGCTGTAGCCTGTAGGTGCTGCCAACCCCCCTCCCCCACTATGGC
####cds
>PGSC0003DMT400003322
ATGGGGAAAAAATCTCCTGCCAAGTGGATCAAGGCCGTGCTTTTTGGGAAGAAATCATCCAAATCTCATTTA
TCAAAGGATGCTTCGGGTGAGAAAATATCTTCTGCAAAAGCGCCGGTGGGGGATCTCTCTCTTGATTCACCA
AGTTTGGATCTACCAGTTCAGAATTTTGATAATGGTGGTGACCAGGCTGGATTGGAAAAGGGTACAAGCAC
TGACTTTGCATGTGAAACCGCCTCATTATCCTCTGCAACACATGATATAGAACCGCATGTAAATGGGACAATCT

CCACAGATGATGCTGAGCTGAAAAGACAAGAGCATGCTGCCACCATAGCACAGGCAGCCTTTAGGGGCTAC
TTGGCTCGTCGGGCTTTTCGAGCTCTCAAAGGCATCATAAGGCTACAAGCCCTTATTCGTGGGCACTTGGTA
AGGAGACAGGCAGTTTCTACTTTACGCTGCATGCAGGCGATTGTCAGGATTCAGGCATTAGCTCGTGGCCG
AAGGATTAGACTCTTAGATCCTGGGCATCAGCTGCTCGGAAAGTATAATTTTGAGGAACTTAAGGATCCTGA
GCAGAGGCCTGCAAAACTGACAGCTTATGCATTTCCACGCAAGCTTCTTGTGGCAgTGCCaACAGCCATGCC
TTTGAGCCTTCAGTATGATGAGTGCGAACCTAATTCAGCTTGGCAGTGGCTTGAAAGATGGTCACTATCTCG
CTTTTGGGAACCACTTCCACAACCAAAGAAAGTAGTTGGAGCAAAGTCGCTGAAGAAGCAGGGTAACAAG
CCGAGTGTTGAAACAGAAGCTGTACGACCAAAACGAAGTGTCAAGAAGGTTCTTACAGCATCAAATGGAG
ATGCTTATGCCGTTAGTTCCTCTGAACCAGAAAAGGCTAAACGCAACCCAAGGAAATTCTCGAATCATCACAT
AGAGCCAGTGCAGGATCAGCCTCAAAATGAACTTGAAAAAGTCAAGCGCAATTTAAGAAAAGTTTCTGCAG
CTTTGGCAACATCTTCAGAGAGGTCTGAAACAGAAATTGAGAAGGCGCAACAGACTCCTAATCTGGCTCAG
GCTCAGGCACAGGCCATAGCATCAAAATCCTCAGCTCCAGATGTTGTTGAACAAATGATGGTTAATTCTTATG
AGAAAACGAGTGATTCAGTTCCTGAAATCGAGAAATTGGCTGAAAGTGAAGCTCCTTTACCTGTACCAGTG
GATGAGCCAAGTGATGTACTGCACGATCATCCTACCACTGAGCAGCAGCAGTCGGAGGATGTTAACAACAC
CGCAAACAGCCCAGTTGTCAATGAGGAACTTAGCTCGATGGAAGATCAGACTACCAAAGAAAGAATTAGGA
GGAGGAAGTCTCTCCCCACTAAGCAGGACAATTCTGAGAATATTTCACAGAATACACCAAGTGTGCCTAGTT
ATATGGCTGCTACTCAATCTGCTAAGGCAAAGCTTAAAGCTCAAGGATCTCCCAAGGTAAGTGATGATGGAG
CTGAAAATGGGTTTGTTCGGCGTCATTCTCTACCATCCTCTGCCAATGGCAAATTCAACTCATTGTCACCTCG
GATTCAAAAACCAGGGCAAGCAAATGGAAAAGGTGGAAACAAGACCAGACCGATGCCCTCCTCGAAAGAC
GAAAAGGTGCTTCCTGGTTGGAGGAGATGA
>PGSC0003DMT400003323
ATGGGGAAAAAATCTCCTGCCAAGTGGATCAAGGCCGTGCTTTTTGGGAAGAAATCATCCAAATCTCATTTA
TCAAAGGATGCTTCGGGTGAGAAAATATCTTCTGCAAAAGCGCCGGTGGGGGATCTCTCTCTTGATTCACCA
AGTTTGGATCTACCAGTTCAGAATTTTGATAATGGTGGTGACCAGGCTGGATTGGAAAAGGGTACAAGCAC
TGACTTTGCATGTGAAACCGCCTCATTATCCTCTGCAACACATGATATAGAACCGCATGTAAATGGGACAATCT
CCACAGATGATGCTGAGCTGAAAAGACAAGAGCATGCTGCCACCATAGCACAGGCAGCCTTTAGGGGCTAC
TTGGCTCGTCGGGCTTTTCGAGCTCTCAAAGGCATCATAAGGCTACAAGCCCTTATTCGTGGGCACTTGGTA
AGGAGACAGGCAGTTTCTACTTTACGCTGCATGCAGGCGATTGTCAGGATTCAGGCATTAGCTCGTGGCCG
AAGGATTAGACTCTTAGATCCTGGGCATCAGCTGCTCGGAAAGTATAATTTTGAGGAACTTAAGGATCCTGA
GCAGAGGCCTGCAAAACTGACAGCTTATGCATTTCCACGCAAGCTTCTTGTGGCAgTGCCaACAGCCATGCC
TTTGAGCCTTCAGTATGATGAGTGCGAACCTAATTCAGCTTGGCAGTGGCTTGAAAGATGGTCACTATCTCG
CTTTTGGGAACCACTTCCACAACCAAAGAAAGTAGTTGGAGCAAAGTCGCTGAAGAAGCAGGGTAACAAG
CCGAGTGTTGAAACAGAAGCTGTACGACCAAAACGAAGTGTCAAGAAGGTTCTTACAGCATCAAATGGAG
ATGCTTATGCCGTTAGTTCCTCTGAACCAGAAAAGGCTAAACGCAACCCAAGGAAATTCTCGAATCATCACAT
AGAGCCAGTGCAGGATCAGCCTCAAAATGAACTTGAAAAAGTCAAGCGCAATTTAAGAAAAGTTTCTGCAG
CTTTGGCAACATCTTCAGAGAGGTCTGAAACAGAAATTGAGAAGGCGCAACAGACTCCTAATCTGGCTCAG
GCTCAGGCACAGGCCATAGCATCAAAATCCTCAGCTCCAGATGTTGTTGAACAAATGATGGTTAATTCTTATG
AGAAAACGAGTGATTCAGTTCCTGAAATCGAGAAATTGGCTGAAAGTGAAGCTCCTTTACCTGTACCAGTG
GATGAGCCAAGTGATGTACTGCACGATCATCCTACCACTGAGCAGCAGCAGTCGGAGGATGTTAACAACAC
CGCAAACAGCCCAGTTGTCAATGAGGAACTTAGCTCGATGGAAGATCAGACTACCAAAGAAAGAATTAGGA
GGAGGAAGTCTCTCCCCACTAAGCAGGACAATTCTGAGAATATTTCACAGAATACACCAAGTGTGCCTAGTT
ATATGGCTGCTACTCAATCTGCTAAGGCAAAGCTTAAAGCTCAAGGATCTCCCAAGGTAAGTGATGATGGAG
CTGAAAATGGGTTTGTTCGGCGTCATTCTCTACCATCCTCTGCCAATGGCAAATTCAACTCATTGTCACCTCG
GATTCAAAAACCAGGGCAAGCAAATGGAAAAGGTGGAAACAAGACCAGACCGATGCCCTCCTCGAAAGAC

GGTAAGAAAAAATGA
>PGSC0003DMT400042507
ATGCATGGTATTCCAGGGGCCTCTTGCTACCGTAACGTCTCAAAATTGGGTTACCAGGCTCTTGAACTTAAAA
TACTACTGCAAGCAGTAGTAAAGTTGCAAAGGTGGTGGAGATGTAAATTGCTTCATGCACAGAGAACAAAA
GCCGCAGTTGTCATCCAATCGCATGCCTTAGGATGGATTGCCCGGCAACGTGCTTCAAGAAACAAGGAACG
ATTACTTCAAGCAGTATTAAAGTTGCAAAGGTGGTGGAGAAGTAAATTGCTTCATGAACAGAGAACAAAAG
CAGCAGTTGTCATCCAATCACATATCCTAGGATGGTTAGCCCGGCAAAGTGCTTCAAGAAATAAGGACCAAT
TACTTCAAGCAATATTAAAGTTGCAAAGGTGGTGGAGAGGTAAATTGCTTCATAAACAGAGAACAAAAGCA
GCAGTTGTCATCCAATCGCATGCCCAAGGATGGAAAGCCCGACAACGTGCTTCAAGAAAGAAGTACCTGAC
ACTGTTAGCAGTATTAAAGTTGCAAAGGTGGTGGAGAGGTAAATTACTTCATAAACGGAGAACAAAATCAG
CAGTTGTCATCCAATCGTATGTCCGAGGATGGATAGCCTGCCAAAGTGTTTCAAGAAACAAGCACCGAATTG
TTGTGATCCAAGCATACATGAAAGGATACCTTGCAAGAAAAGATTTAAGAGGGCAGCTTCTTGATTTGCGTC
ACAAAATACAAAAATCTGCTGCAAATGTTGATGATGGTATGCGCATAATAAACAGACTTGTCGCAGCATTGTC
AGAACTTCTGAATATGAGAAGTGTCAGTGACATTCTTCATATCTGTGCAACTCTGAATATGGCAACACAACAT
TCACAAAAGTGTTGTGAAGAACTTGTTGCTGCGGGTGCTGTTGGTACATTGTTTAAGCTGATCCGCTCTCTC
AGCCGGAGCATACCTGATCAAGAAGTTCTTAAACCTGCTCTCTCCACTCTCAGAAACCTTTCCCGATATCCAC
ATTTGATTGATGTACTAATTGAAAGCTATGGATCACTGGAAACAATAGTGTCGGAGTTTTTAAGAAACAAAG
AAGAGGGATATTTCATTGCTTCCGATCTTCTGAAGAAGATATTCACAGAGAAAAAAGGTGTTGAAGCTGTGT
GCAAGTCGCCTGCTCTTTTGAAGAGACTGCACAATCATGTGGAAGAGCTTTCAAGAAGAGCAAAAGCTGAC
AAGAGGACTAAACCTCACGCAATGAAAGAGCCTGTGGACAAAAGATTGAGAGAAGCTGTTGAGATATTGG
AATTGATTAAAGTGTCCATGGGAAATCCAAGTAAACGACTCTCTATGAAGGTGTAA
>PGSC0003DMT400057729
ATGGTGACAACTTATCAGATGAAGAGTCTTAGTACATTAGAGCTTATGTTAGAGGAACTTCAGCAAGAAGAT
GAAGGAACAAATGATTTGCCACCACCTTTGCCTGTTAGGCCTGTCATTAAGGCTAGGTTGCCTAAGGGCAG
AAGGAAATTGACATTTGGTAAAGAAAAGAGCAATCTTGAAGATATTAGGGTTCAAGAAAATGTATTTGTTGA
TCAATGGAGTACTTCAGCAGAGAGGGATTCTGCAGCCATGACAGATAAGATTTGTTTGATGGTTGATCAGAG
AGAAGGAGCTGAACTAAGTAGAGTACTGCAAATTCAGAGATGCTTTCGCGGCTATCAAGCCCGCCAGTACT
ATCATGAGCTTAAAACAGGAGCAGTAGCTCTACAATCATTTGTGCGTGGTGAAATTGAAAGAAAGTATTATC
AGGGTCTCACTAGGAGGTTAGCAGCAATTATCTTTATACAGAAACACATAAAGAAGCATCATCATAAAAGAA
CAGAACGACAACGAACTGCAGCCATATGTTTGCAGTCTGTAATTCGAGGTTGGTTGACTCGAAAGAAGTCC
AATCTCTCGGGTGATGAAAAACGATCTTGTGTTCAGAACATTAGAGAAAAAAATGACCTTGACAATAAGGAA
CCAGAAACTAAGGTACCGCGTTCAGTTCTACTTGATCTTCAGAGGCATATTTTGAAGACAGAGGCAGCTCTG
GAGAGAAAGAAAGGGGAAAATGCAGCTTTGAGGCTACATATTCAGCATTATGAGATAAAGTGGAATCAGTA
TGAATCAAAAATGAAAGCTATGGAGAAGATGTGGCAAGATCAGTTAACGTCTATACAAATAAGTCTGGCTGC
AGAAAGGGAAAAACACGGGGACGAAAAGACTAAGGGAAAACTCAGACTACTGATACTTCAAGATCAAGAC
GAAAATGTGCATAACGGATTCCCAAGAACTATATCTCTTCGAACAAGTGCATTGAATCATCCTGATGAGCAAC
CAAGCGGGAGATCAAATCCCAAGAATAAGTGTAATAACCACCATGTGATGGACATGGTCAATCACTATCAAA
ATTTTGTTGTCCACGATCAATGTAATTCAGGGGAGGAGGGTTCCGCCCTCAGGCCTAACGATGAGCTCCAGA
AGCTGAAGATTAGATTTGAAGCATGGAAGAAGGATTACAAGAATAAATTACGCGAGGCCAAAGCAACAATG
AAGCAACTAGGACACTCTGAAAGGGGGAAGGGTTCAAAGATATGGTGTGGAAGATGA
>PGSC0003DMT400009431
ATGAGGATGTCTAAGAAGAATTGGTTTGGTAATATCAGAAAGAAACTATTCAGGTCATCTCCTCCACATAAAA
ATATCATTGTTCTTCATAACAACACAATTACCAATAGGACCAGCAGCGCCAATGGACGGTCATCTACAAAGAA
AAATAATGGGCACATCTATTTTATGTCTAAAGAGGATATGGCTGCTATTACTATCCAATCTCATTTTAGGGGTCA

TCTTGCGAGACGGGCGTTTAAGGCACTGAAAAGCCTTGTGAGGCTTCAAGCAGTGGTAAGAGGGGCATAT
GTGAGAAGACAAGCAAGGATAGCTCTGCATTGCATGCACGCCCTTGCACGGTTACAAGTTACTGTCCGAGC
TAGGCAACTCCTCAGCAAGTGCAATGACCATTGA
>PGSC0003DMT400053991
ATGGCATCAGTCAACATTATTGTCGGTTCTCATGTTTGGGTGGAAGACCCTAAATTGGCATGGAAAGATGGA
GAAGTAATCAAAATACATGGTCAAGATCTTCATGTTAAAACCTCCGATGGGAAAGAAGTTGTTGCTAAAATC
GCTAAAGTGTTTCCTAAAGATACTGAGACTCCTCCTGGAGGTGTAGATGATATGACCAAGCTTTCCTATTTGC
ATGAACCTGGAGTTCTGCAGAACTTGGCCACCCGATATGAGCTCAATGAAATTTATACGTACACTGGGAACA
TATTGATTGCAGTAAACCCTTTCCAAAGATTGCCTCATCTGTACGACACTCACATGATGGAACAGTACAAAGG
AGCAGCATTTGGGGAGCTAAGTCCGCATGTTTTTGCAGTTGCAGATGTTGCATATAGGGCAATGATCAATGA
GGGAAAAAGCAATTCAATTTTGGTTAGTGGAGAAAGTGGTGCTGGTAAAACTGAAACTACTAAGATGCTTA
TGCGTTATCTTGCGCATCTTGGGGGCCGGTCAGGTGTCGAGGGACGAACTGTAGAACAACAAGTTCTAGAA
TCCAATCCCGTTCTTGAAGCATTTGGAAATGCCAAAACTGTGAGGAACAACAACTCAAGTCGTTTTGGTAAA
TTTGTTGAGATACAATTTGATAAGAGTGGGAGGATATCTGGGGCAGCTATACGAACTTACCTTCTGGAGAGG
TCTCGCGTCTGTCAAATTTCAAATCCTGAGAGAAACTACCATTGCTTTTATCTTCTTTGTGCTGCTCCAGCTGA
GGAGGTTGAGAGATATAAACTACAGAACCCAAAATCATTTCACTATCTTAATCAGTCCAAGTATTATGAATTG
GATGGAGTAAATGATGCTGAAGAATATCTTGCAACAAGAAGGGCTATGGATATCGTAGGAATCAGTGAGGA
AGAGCAGGATGCAATTTTCAGGGTGGTTGCTGCAATTCTTCACCTTGGTAATGTCGAATTTGCAAAAGGTGA
GGAGATTGACTCTTCTGTGATTAAGGATGAGCAGTCTCGATTTCATCTCAATATGACGGCGGAGTTACTCAAG
TGTGATGCCAAGAGCTTGGAAGATGCACTAATTACACGTGTGATGATCACACCCGAGGAGGTTATTACAAGG
ACTCTTGATCCAGAAGCTGCTCTGGGTAGCAGGGATGCTTTGGCTAAAACCATATATTCTCGCCTTTTCGACT
GGATTGTGGAAAAGATAAACATCTCAATTGGCCAGGATCCAAACTCCAAGTCAATAATCGGAGTTCTTGATAT
TTATGGGTTTGAGAGTTTTAAAACGAACAGTTTTGAGCAATTCTGCATCAATTTTACAAATGAAAAGTTGCA
ACAACATTTTAACCAGCACGTGTTCAAGATGGAACAAGAAGAATATGAAAAAGAAGAGATTAACTGGAGCT
ACATAGAGTTTGTTGACAACCAAGATGTGCTGGATCTGATTGAAAAGAAACCTGGGGGAATTATTGCTCTGT
TAGATGAAGCCTGTATGTTTCCTAAATCTACTCATGAAACATTTGCTCAGAAGTTGTACCAAACATACCCTAAA
AACAAGCGCTTCATCAAACCTAAACTTTCACGGACGAGTTTTACAATATCTCATTATGCTGGAGAGGTGACAT
ATCAAGCGGATCTGTTTCTGGATAAGAACAAAGATTATGTGGTTGCAGAACATCAGGTTCTGTTAACAGCCT
CAAAATGTACTTTTGTGGCGGGTTTGTTTCCTCCTCTACCTGAAGAATCATCGAAATCGTCCAAATTCTCCTCC
ATAGGGTCTCGTTTTAAGCTACAACTGCAATCTTTAATGGAAACATTAAGTTCAACAGAGCCTCACTACATCA
GATGTGTGAAGCCTAATAATGCCCTTAAGCCTTGTATCTTTGAGAATCTGAATGTGATCCAGCAATTGCGATG
TGGTGGTGTCTTAGAAGCTATCAGAATCAGTTGTGCTGGATATCCTACTAGACGTACATTTTATGAGTTTCTTC
TTAGATTTGGTGTTCTTGCTCCAGAAGTTTTAGCTGGAAGCTATGATGACAAAGTTGCATGCCAGATGATTCT
AGACAAGAAGGGACTTAAGGGTTATCAGATGGGAAAGACAAAGGTCTTTTTGCGGGCTGGACAGATGGCT
GAGCTCGATGCTCGGAGAGCTGAGGTACTTGGAAATGCAGCAAAAATTATTCAAAGACAAATCCGTACATAT
ATTATGCGAAAAGAATTTGTTTCTCTGCGTCAAGCTGCTATTCAGTTGCAATCATGTTGGCGAGCTATGCTGT
CCTGCAAACTGTATGAACAACTGAGACGTGAAGCAGCTGCTCTGAAGATTCAAAAGAATTTCAGATGTCATG
TTGCACACATAACATATACAACGCTGCATTCTTCTGCAATTATGTTGCAAACAGGCATGAGAGCCATGGTTGC
TCGGAATGACTTTAGATTCCGGAAACACACTAAAGCTGCAATTAAAATACAGGCTCATGCACGTGGCCATGC
CGCTTATTCTTATTACAGAAGTCTTCAGAGAGCTGCGATCATTACTCAGTGTGGTTGGAGGCGACGGGTCGC
CAGGAAGGAGCTTCGAAATCTCAAAATGGCTGCAAGGGAAACAGGTGCTCTCAAAGAAGCCAAGGACAA
GCTCGAAAAGAAAGTGGAAGAACTTACATGGCGGTTGCAATTTGAGAAACGACTCAGGGCTGAGCTGGAG
GAGACTAAAGCCCAAGAAGTTACAAAGCTACAGGAGGCACTGCATACAATGCAAAAGCAAGTAGAAGAAG
CAAATGCTAAAGTTGTCCAAGAGCGGGAGGCAGCACGGAGAGCAATTGAAGAAGCACCTCCAGTCATCAA

GGAGACCCCAGTTATAGTTCAAGACACTGAAAAAATAAATGCCCTGTCAGCTGAAGTAGATAATTTGAAGGC
TTTGCTGGCATCCGAAAAGAAAGCTACAGAAGAGGCTAGAGATTCTTCCAGGGATGCAGAGGCCAAAAAC
ACAGAGCTGGCTAGCAAACTAGAAACTGCTGAGCGAAAAGTAGATCAGCTTCAAGATTCTGTGCAGAGGCT
TGAAGAGAAGCTTTCCAATATGGAATCAGAGAACCAAGTGCTTCGACAACAAGCTTTGACCATGTCACCAA
CTGGAAAAGCTTTATCTGCACGGCCAAAGACTACCATCATACAGAGGACTCCGGAGAATGGAAATGTTATAA
ATGGAGAATCAAAACCTAATTCTGATATGAGTCTTGTTGTAGCAAGTCCAAAGGAGCCTTCATCTGAAGAGA
AACCACAGAAGTCTCTAAATGAAAAGCAGCAGGAGAACCAAGACATGCTCATTAAGTGCATTTCTCAAGATT
TGGGCTTTTCTGGAGGCAAACCAATTGCAGCTTGTCTCATATACAAATGTCTGCTCCACTGGAGGTCCTTTGA
AGTGGAAAGAACTAGTGTTTTTGACCGTATAATACAAACCATTGCTTCAGCCATAGAGGTCCCAGATAATAAT
GATGTATTAGCCTACTGGTTATGCAATACGTCCACATTGTTGATGCTGCTTCAACAAACACTTAAAGCTAGTGG
GGCTGCTAATTTGACTCCGCAGAGGCGGAGATCCAGTTCAGCCTCTTTGTTTGGAAGGATGTCCCAAGGCT
TGCGAGGTTCTCCTCAGAGTGCTGGGCTTTCAGTTCTCAATGGGCGTATGCTTGGGAGATTGGATGACTTAC
GTCATGTTGAGGCCAAGTATCCTGCGCTGCTGTTCAAGCAGCAACTGACTGCCTTTTTGGAGAAAATATATG
GAATGATAAGAGACAATCTGAAGAAAGAGATCTCCCCATTGCTTGGGCTATGTATTCAGGCACCAAGAACAT
CTCGTGCAAGTTTAATCAAAGGAAGATCCCAAGCTAATGCTGCTGCCCAGCAAGCTCTATTTGCTCATTGGC
AAAGCATTGTAAAAAGTTTGAACAACTACTTGATGATGATGAAATCAAACCATGTTCCTCCCTTCTTAGTTCG
GAAGGTTTTCACTCAAATATTTTCCTTTATCAATGTTCAACTTTTCAACAGTCTTCTTTTGAGGCGTGAGTGTT
GCTCATTTAGTAATGGAGAGTTCGTGAAAGCCGGGTTGGCTGAATTGGAACAGTGGTGCTGCTATGCAACT
GAAGAATTTGTAGGCTCAGCATGGGACGAGTTGAAGCACATTAGACAGGCAGTTGGATTCCTAGTTATACAT
CAAAAGCCCAAAAAGTCATTGAATGAAATCACTAATGAACTTTGTCCAGTGCTTAGCATACAGCAACTGTATA
GGATCAGCACTATGTACTGGGATGACAAATACGGAACCCACACTGTTTCTTCAGATGTTATTTCAAGTATGAG
AGTTATGATGACAGAGGACTCCAATAATGCTGTCAGCAGTTCCTTTTTGTTGGATGATGATTCGAGCATTCCA
TTCTCTGTGGATGACATCTCCAAAACGATGCAACAAATAGACATTGGCGATGTTGAACCTCCTCCATTAATCC
GTGAGAATTCAGGATTTGTATTCTTACATCAACGGTCTAGTTGA
>PGSC0003DMT400053990
ATGGCATCAGTCAACATTATTGTCGGTTCTCATGTTTGGGTGGAAGACCCTAAATTGGCATGGAAAGATGGA
GAAGTAATCAAAATACATGGTCAAGATCTTCATGTTAAAACCTCCGATGGGAAAGAAGTTGTTGCTAAAATC
GCTAAAGTGTTTCCTAAAGATACTGAGACTCCTCCTGGAGGTGTAGATGATATGACCAAGCTTTCCTATTTGC
ATGAACCTGGAGTTCTGCAGAACTTGGCCACCCGATATGAGCTCAATGAAATTTATACGTACACTGGGAACA
TATTGATTGCAGTAAACCCTTTCCAAAGATTGCCTCATCTGTACGACACTCACATGATGGAACAGTACAAAGG
AGCAGCATTTGGGGAGCTAAGTCCGCATGTTTTTGCAGTTGCAGATGTTGCATATAGGGCAATGATCAATGA
GGGAAAAAGCAATTCAATTTTGGTTAGTGGAGAAAGTGGTGCTGGTAAAACTGAAACTACTAAGATGCTTA
TGCGTTATCTTGCGCATCTTGGGGGCCGGTCAGGTGTCGAGGGACGAACTGTAGAACAACAAGTTCTAGAA
TCCAATCCCGTTCTTGAAGCATTTGGAAATGCCAAAACTGTGAGGAACAACAACTCAAGTCGTTTTGGTAAA
TTTGTTGAGATACAATTTGATAAGAGTGGGAGGATATCTGGGGCAGCTATACGAACTTACCTTCTGGAGAGG
TCTCGCGTCTGTCAAATTTCAAATCCTGAGAGAAACTACCATTGCTTTTATCTTCTTTGTGCTGCTCCAGCTGA
GGAGGTTGAGAGATATAAACTACAGAACCCAAAATCATTTCACTATCTTAATCAGTCCAAGTATTATGAATTG
GATGGAGTAAATGATGCTGAAGAATATCTTGCAACAAGAAGGGCTATGGATATCGTAGGAATCAGTGAGGA
AGAGCAGGATGCAATTTTCAGGGTGGTTGCTGCAATTCTTCACCTTGGTAATGTCGAATTTGCAAAAGGTGA
GGAGATTGACTCTTCTGTGATTAAGGATGAGCAGTCTCGATTTCATCTCAATATGACGGCGGAGTTACTCAAG
TGTGATGCCAAGAGCTTGGAAGATGCACTAATTACACGTGTGATGATCACACCCGAGGAGGTTATTACAAGG
ACTCTTGATCCAGAAGCTGCTCTGGGTAGCAGGGATGCTTTGGCTAAAACCATATATTCTCGCCTTTTCGACT
GGATTGTGGAAAAGATAAACATCTCAATTGGCCAGGATCCAAACTCCAAGTCAATAATCGGAGTTCTTGATAT
TTATGGGTTTGAGAGTTTTAAAACGAACAGTTTTGAGCAATTCTGCATCAATTTTACAAATGAAAAGTTGCA

ACAACATTTTAACCAGCACGTGTTCAAGATGGAACAAGAAGAATATGAAAAAGAAGAGATTAACTGGAGCT
ACATAGAGTTTGTTGACAACCAAGATGTGCTGGATCTGATTGAAAAGAAACCTGGGGGAATTATTGCTCTGT
TAGATGAAGCCTGTATGTTTCCTAAATCTACTCATGAAACATTTGCTCAGAAGTTGTACCAAACATACCCTAAA
AACAAGCGCTTCATCAAACCTAAACTTTCACGGACGAGTTTTACAATATCTCATTATGCTGGAGAGGTGACAT
ATCAAGCGGATCTGTTTCTGGATAAGAACAAAGATTATGTGGTTGCAGAACATCAGGTTCTGTTAACAGCCT
CAAAATGTACTTTTGTGGCGGGTTTGTTTCCTCCTCTACCTGAAGAATCATCGAAATCGTCCAAATTCTCCTCC
ATAGGGTCTCGTTTTAAGCTACAACTGCAATCTTTAATGGAAACATTAAGTTCAACAGAGCCTCACTACATCA
GATGTGTGAAGCCTAATAATGCCCTTAAGCCTTGTATCTTTGAGAATCTGAATGTGATCCAGCAATTGCGATG
TGGTGGTGTCTTAGAAGCTATCAGAATCAGTTGTGCTGGATATCCTACTAGACGTACATTTTATGAGTTTCTTC
TTAGATTTGGTGTTCTTGCTCCAGAAGTTTTAGCTGGAAGCTATGATGACAAAGTTGCATGCCAGATGATTCT
AGACAAGAAGGGACTTAAGGGTTATCAGATGGGAAAGACAAAGGTCTTTTTGCGGGCTGGACAGATGGCT
GAGCTCGATGCTCGGAGAGCTGAGGTACTTGGAAATGCAGCAAAAATTATTCAAAGACAAATCCGTACATAT
ATTATGCGAAAAGAATTTGTTTCTCTGCGTCAAGCTGCTATTCAGTTGCAATCATGTTGGCGAGCTATGCTGT
CCTGCAAACTGTATGAACAACTGAGACGTGAAGCAGCTGCTCTGAAGATTCAAAAGAATTTCAGATGTCATG
TTGCACACATAACATATACAACGCTGCATTCTTCTGCAATTATGTTGCAAACAGGCATGAGAGCCATGGTTGC
TCGGAATGACTTTAGATTCCGGAAACACACTAAAGCTGCAATTAAAATACAGGAATTTATCTCTCTACTGCTAT
TACAATGCTTGTGA
>PGSC0003DMT400049643
ATGATTCAGGAATATGAATTGGACGGAATTGATTGGTCAAAAGTAGATTTTGAAGACAACCAAGAGTGTCTG
AATCTTTTTGAAAAGAAGCCAATTGGCCTTATATCTTTGTTGAATGAAGAATCAAATTCCCTAAAAGCCACAG
ATTTGACCTTTGCATGTAAACTTAAGCAGCACATCAAATCTAGCCCTTGCTTTAAAAGCGAAAGAGAAGAAT
TTTGTATTCGTCATTATGCTGGAGAGGTAACTTATGATGCAACTGGCTTCTTAGCAAAGAACAGAGATGTGTT
GCATCCTGACATTATTCAGCTACTCTCATCAAGTGATAGTCACCTGCCTGAAGATAAAAAATTCTCAATTCCAT
CAACTGATACAGGGGTGCTAGATTTTAAGAAGCAAAGTGTTGCAACTAAGTTTAAGGATAATTTGTTCAAAT
TGATGCAGCAATTGGAAAATACCATACCACATTTCATATGTTGCATAAAACCAAATAATAAGCAGCTTCCTGGC
ATGTCTGACAAAGATCTTGTCATAGAACAGCTCAGATGCTGTGGTGTTCTTGAAGTGGTTAGAATATCAAGAT
CTGGCTATCCTACTAGGTTAACACATCAAGAATTCACAAGCAGGTACGGCTTCCTTCTGCCAAAGGATAATGC
ATGCCAAGATCCTTTAAGTATGTCAGTTGCCATTCTTCATCAATTTGGTATTCTTCCGGAACTGTACCAAGTTG
GGTATACAAAGTTATATTTCCGATCAGGACAGATTGCTTCATTGGAGGATGTAAGGAACCAAGTTCTGCAAG
GTACTCTTGAGGTGCAGAAGTGCTTCCGTCGTCATCGTGCTCGTCGGCACTTCCATGAGCTGAAAGGAGGA
GTAATCATACTTCAATCATTTATTCGTGGTGAAATAGAAAGAAGGCTGTATAATACTAAAGTGATGTCTAAAGG
GAAGGTTGCTCGTGAAGGAAGTGATGAGCAGCTGGTGGCTGTTGTGCAGATACAATCAGCTATTCGTGGTT
GGTTGGCTAGAAGGGATCTTCGTAAACTGCAGAATTCAAAAATGTTAAATGTAGACAAACGAAGATCAGGC
AGAAAGACGGAGGTCAAGGAGTTGCCTCGAGAAATCCTACCATCTGTTGTAGAAGACCTCGAAAGACGAG
TTGCAAAGGCCGAGGCAACCATTGAACAGAAGGAAAAGGAAAATGCTGCCCTGAAGGAACAAGCAAACC
AATTCGAGGCCCGATGCTTAGAATATGAGGTCAAGATGAGGTCAATGGAGGAGATGTGGCAAAAGCAAATG
ACATCATTGCAGGCTAATCTAGCTGCAGCCAAGAACACTCTTGCCGCTGGTGACACTACTGGTCAACCTGGA
AAGCTTGAAGGTTCCCCATCTCCTCACTATTATGATTCTGATGATGCAACATCTATGGACACTCCTGCGGGAC
GCACTCCAATTAACTTTTCTAACAACAGCTTGGGTGTTGTAACTAATAGAGAGGTTAATGGTGGTTTATCCTT
AATCAGCCACCTTACAATGGAATTTGAACAACGGAAGCAGAATTTTGACAACGAAGCCATGGCAATTGTTCA
CTTGAAGCCAGGGCTGTTACATTCTACTAATAATCCTGCAGATGAGTATCGAAGACTTAAACACAGGTTTGAG
GAATGGAAAAAAGAGTACAAGGTCCGGTTAAAGGAGACGAAGTCAAAAGTACACAAGCTTGTTCATTCTA
AAGCAGGGAAGAGTCATAGAAAATGGTGGGGTAAGAAGAGCAAGTGA
>PGSC0003DMT400038175

ATGAAGAAGAAACATATCCCCTCATATAGCAAGCAAACACAAGGCCAAGGGACAACAACAACAACAAATGA
TCATCAACTAAGTTTAGTAACACAAGATGAAAGTTTGCTAACAAAATGTTTCATAGGGAAGAGTATAATTGGT
GGGCCTTCTTGGTTAGGGTTAATGAAGAAGGCTTTTAGATCACCTATCAAAGAGAATGATATCAAAAGTATTA
GAAGAAGGGAAGAAGCTCATGACCAAGAAGAAGAAGAAAAGAAGAGGGGGAAACGACGATGGATCTTC
CGAAAACCTACTGTTCACGAAACAACATTAATTCATCACAATCAAGATCAAGAAAATGCAGCTACAACAAGC
AAAGGAAATTTGGCTACAATTTTTTTGCCAAAAAATGCTGATTTGAAGCAAAAAGGAGCAATTGGAGTAGC
TATAACTAGGGGAGGATCTAGCTTATCAGCCGCTACAACTTCTACAATAGCCACTGATGTCGCAATTGCTAAA
ACACAAGCAACTATAGATATCACTCAGCTCACACAACCTCCTATTTTAGTTAAACAACAATGTGCCATCCTTGT
CATTCAGACAACATTCAGAGGGTACTTGGCAAGAAAAGCACTTGGAGCGCTCAAGGGTGTCGTGAAATTGC
AAGCTTTAATAAGAGGCCATAATGTTCGAAAACGAGCGCAAATTACACTCCAATGCATGCAATCTCTTGTTCG
CGTGCAAAATCAGGTGTGTGATCAACGTAGAAGGTTATCTTGTGAAGGAATTAGTTGTGATTCAATGTTCAA
AGAGCCAAAAAGCATTTTGGAGCTTCATCTCAATGATAAGGAGTCCAATTCTATAAATCAAGCTAGTATTCCG
GACAACGATGGTTATGATCATTTACATGCACTAGAAAAAATTGAAGCTTTGTTACACAAAGCAAAGGTAGCT
GCTAAAAAGCGCGAAAATACGCTTGCTCATGCATTCTCTCGAAAGATGTGGACCTCGAATAAGGATGAAGAT
TCAAGTAGCAATACAGAACTTGACGAGGATTTGAGAGTTTTTGACGTAATAGATGAAAAGAATCGCAAAAG
TACTAGTAGAGCTTCATGCGATCAACCAAGAGATCGCATAAAGAACATTGAAATTGACACGGCTTGTTCTTAC
TCTAACTCGGACACTGAGTTTTGGCGATTACACCATCAATACTATCACGACCATCAACAAAAGTTCTATTCATC
GTATGTAGTCCCTTCTCCTCTCTCCCATAGAGCGAATATCAACGCCTTGCCCATGACACCACCGTTAAAGATGA
AAAATATCCAAGTTCACTCTGCTAGTCCTCGCTACAGAAGGGAAGAGAACAACCATCACCGAATGGCTCTTC
TCTCCCATAGAGCGAATGTTAACTCTTCACCTACAGCAGTTGATCAGCCAAGTTACATGGCAGCTACAGCATC
AGCTAGGGCGCGAGAACGGTCACAAAGCACTCCAAGACAGATACCGATGACTCCAGAAAGAGAAAAAAACA
AGTTCAACAAAGAAACGTCTATCTTTCCCTATTCAATGA
>PGSC0003DMT400079921
ATGGGAACTCCTGTAAACATCATAGTTGGATCCCAGGTGTGGATTGAGGATCCTGATGATGCCTGGATTGAT
GGAGAAGTCACAGAAATTAAAGGCTCAAATGCCACAGTAGCCACAACTAATGGGAAGACGACAGTTGCTTC
TATTTCTAGCATATACCCAAAGGATACCGAAGCACCACCATCTGGGGTTGATGACATGACCAAGTTAGCTTAT
CTCCATGAGCCTGGCGTTCTGAATAACCTTGCTTGTCGGTATTCTCTAAATGAGATATATACTTACACGGGGAA
TATCCTAATTGCGGTCAATCCATTTCGGAGACTTCCACATCTGTATGATACCCATATGATGCAGCAATACAAGG
GAGCCCCATTTGGAGAGCTAAGTCCACATCTTTTTGCAGTGGCAGATGCCTGTTATAGGGCATTGATAAATG
AGCATGGCAACCAGTCTATCTTGGTCAGTGGAGAGAGTGGTGCTGGTAAAACTGAGACAACGAAAATGTTA
ATGAGATATCTCGCGTTTATGGGTGGAAGGTCTGGCACTGAAGGAAGAACAGTCGAGCAACAAGTTTTGGA
GTCCAACCCAGTTTTAGAAGCATTTGGGAATGCAAAGACTGTGAAGAACAACAATTCCAGTCGCTTTGGTA
AATTTGTTGAAATTCAGTTTGATAAGCACGCGAAGATTTCTGGGGCTGCAGTTAGGACATATCTTCTTGAAA
GGTCACGTGTTTGCCAAGTCTCAGACCCAGAGAGAAACTACCATTGTTTTTACATGCTTTGTGCTGCACCAC
CAGAGGATGTGAAAAGATTCAAACTTGGGAATCCAAAATCATTTCATTATCTAAACCAAAGTAGCTGTTACG
AAGTTGCAAATGTTGACGATGCAAGAGAGTATCTTGAAACCAGAAATGCTATGGATGTTGTTGGAATCGGTC
AGGAGGAGCAGGAAGCTATATTTCGTGTTGTAGCTGCAATACTCCATCTCGGAAACATCAACTTTGTGAAAG
GAAAGGAGGCAGATTCCTCCAAACTAAAAGATGAGAAGTCACTTTTTCATCTGAAGACAGCTGCAGAGCTA
TTCATGTGTGATGAGAAGGCACTAGAAGACTCACTTTGTAAGCGTGTCATTGTAACTCCTGATGGAAACATC
ACAAAACTACTGGATCCAGCAGCTGCAACCACGAGCAGGGATGCCCTGGCAAAGACCGTATACTCCAGATT
GTTTGACTGGCTTGTGGACAAGATAAACAATTCAATTGGACAGGATCCTGAAGCAAAAAGCATAATTGGCG
TCCTTGATATATATGGCTTTGAGAGCTTCAAAATCAACAGTTTTGAGCAATTCTGCATCAACCTAACAAATGA
GAAGTTGCAGCAGCATTTTAACCAGCACGTATTCAAGATGGAACAAGACGACTATACGACAGAAGAAATCA
ACTGGAGTTATGTGGAGTTTGTAGATAACCAAGATGTTTTAGATCTTATTGAGAAGAAACCTGGAGGCATTAT

TGCTCTTCTTGATGAAGCTTGTATGTTCCCAAAAGCAACTCATGAGACATTTGCCCAAAAGATGTACCAGACA
TACAGAGCCCATAAACGGTTTAGCAAGCCAAAACTTGCTCGTACTGACTTTACAATCAATCATTACGCTGGTG
ATGTTACTTACCAAGCAGATCACTTCCTTGACAAAAATAAGGACTATGTGATAGCAGAATTTCAAGCTCTTCT
GATGGACTCAAAGTGCTTTTTCGTTGCAAACCTCTTCCCTCCATTGCCCGAAGAATCGTCCAAGCAGTCCAA
GTTCTCATCCATCGGCACACGGTTTAAGCAACAACTGCAATCTTTAATGGAGACCTTGAGCACTACAGAGCC
ACATTACATCAGATGTGTAAAGCCCAATACAGTTCTAAAGCCAGGAATATTTGAGAACATGAATGTATTAAAC
CAATTAAGATGTGGGGGTGTCTTAGAGGCGATCAGGATAAGTTGTGCAGGATACCCAACGAAAAGAACATT
CGATGAGTTCCTTGACCGCTTTGGAACGTTAGCTCCAGATGTTCTTGACGGATGTGATGAGAAGTCAGCATG
CATTGCAATTTGTGATAGAATGGGCTTAAAGGGTTATCAGATTGGGAAAACCAAAGTTTTTCTCAGAGCCGG
GCAGATGGCTGAATTAGATGCCAGAAGAACAGAAGTTCTAGCTCATGCTGCAAAGCGCATTCAGAGACAAA
TTCGAACATATCTTACCCGAAAGGAGTTCATAGCCCTAAAGAGAGCTACAATTCATTTCCAGAAACTTTGGA
GAGCACAACTTGCCAGAGTGCTGTATGAACAAATGAAAAGGGAAGCTGCTTCAATCCGCATACAGAAACAT
GCGCGTTCTCATTCAGCAAGAAAATCTTACAAGGAGTTACAGGCAGCAGCTGTAGTCATTCAAACAGGGAT
GAGAGCTATGGCAGCACGAAATGAATATAGGCAGAGGAGGAGAAATAAAGCAGCTAAAATAGTTCAGACTC
AATGGAGAGGATTCCATGCCTTCTCAACTTATAAACAGAAGAAAAAAGCAAGTCTTTCACTTCAATGTCTCT
GGAGAGGCAGGTTAGCGAGGAAGGTGCTTCGGAAATTGAGGATGGATGCAAGAGATACTGGGGCACTTA
AAGAAGCAAAGGACAAACTGGAAAAGCGTGTCGAGGAGTTAACATGGCGATTAGATTTTGAAAAACACTT
GAGGATTGATCTCGAAGAAGCAAAGGGGCAAGAAATTTCAAAATTGCAAAAAGCCTTACAAGAAATGCAA
ACACAACTAGACGAAGCCCATGATGCAATTATTCATGAGAAAGAAGCAGCAAAGATAGCCATTGAACAAGC
TCCTCCAGTTATCAAGGAGGTACCAGTGATTGACAACACCAAAGTGGAGAAGCTGACAGAGGAAAACAAC
AAACTCGAGGAAGAAATCAGAGAGCTCAAGAAGAGAGTAGAAGACTTCGAACAGAGCTACAACGAAGTT
GAAAAAGAATGCCAGGCCACACGTAAAGAGGCAGAAGAATCTCAGCTAAGAGTTTCGGAGTTCCAAGAGT
CCATAGAAAGATTACAACTGAATCTATCCAACCTTGAATCAGAGAATCAGGTTCTCCGTCAGCAGGCTTTAGT
AGCATCAACAAATGAGGCCCTTTCTGATGAAATGGACATACTCAAGAACAAGATTAAAGACTTGGAGTCAG
AAAACGAGCTTCTCCGCACTCAAAGGGTAGTTGTGGAGCAAGTAGTAAGTTCTGATGATCGGGCACCTAAA
GGGCTTGAGACCGTTGATATCACACATCCAGCTGATAATGAACATCAAACAGAGGAAGTGCATGAAGAAAT
GAAAGTAGAACAACAAATACCTAAGCTTCTGCAGGATTCTAGCCCTCCCATCTCTTTAACTAAGCAAAGATCC
TTGACAGATAGGCAGCAGGAAAGCCATGATATACTGATAAAGTGCCTTGCGGAAGACAAGCGATTTGACAA
GGGAAGACCAGTAGCTGCATGCACCCTGTACAAAGCACTTCTGCAGTGGAGATCCTTTGAAGCAGAAAAAA
CGAATATATTTGATAGGATTATTCATACCATCCGATCATCTATAGAGGATCAGGATAACACCGGTGATCTAGCCT
ACTGGCTGTCAACATCTTCAACTCTGTTGTTCCTTTTGCAAAGCACAATTAAAGCTGGTAATGCGCCTACTAG
GTCCCCATATCGTAATCGCAGCTCACCAACCACATTGTTTGGAAGAATGGCACAGCAGGGTTTTCGTTCAAC
TTCATTAAGCATGGCAATTTCCAGTGGATACAGCGGAATTGAGGGAAGCCCAAATGTCCGAACAAGGATTG
AAGCTAAATACCCAGCACTATTGTTTAAGCAACACCTAACTGCTTGCGTTGAGAAAATATATGGAATGATCCG
CGACAACTTAAAGAAAGAAATAAGTCCATTTCTTAACCAATGCATACATGCACCAAGATCTGCAAGGATAAA
ACCTTTAAAAGGATCATCTAGAAGTATACATTCAAACATCATCGCGAAACAACAGGCATCCAGCGTACACTGG
CAAAACATTGTGAATAGCCTAGACAATACATTAACCATACTCTCAGAAAATAATGTCCCTTCCACCATTACAAG
GAAAATACTCAGTCAGGTGTTCTCATACATAAATGTCCAGCTTTTCAACAGCTTGTTGCTTCGCCGTGAATGC
TGCTCATTTAGCAATGGGGAATATCTGAAGGCGGGTCTGCAAGAACTGGAAAGTTGGTGTTCCAAAGCAAC
AGAACAGTATGCTGGATCTTCGTGGGATGAACTTCAACACATAAGGCAAGCTGTAGGATTTCTGGTGTTACA
TCAAAAATCTCAAAAGGCATTGGATGAGATCACGAATGACCTCTGCCCGATGTTGAGCATTGCACAAATATAT
CGCATTGGAACTATGTTCTGGGACGACAAATATGGAGCCCATGGTTTATCGCCAGAGGTCATAAGCAAGATG
AGAGCACTAACATTGGAAGACTCAGCCAGCATTCCAAATAATACATTCTTGCTTGATGTGGATTCAAGCATAC
CATTCTCTATAGAAGAAATATCGCGATCCTTCCAGAATATCAACTTGTCTGATGTGGAACCACCTCCCCTCCTT

TGTCAAAGATCAGATTTTCAGTTCCTGCTGCAGGCAGCAGCATAA
>PGSC0003DMT400079917
ATGGGAACTCCTGTAAACATCATAGTTGGATCCCAGGTGTGGATTGAGGATCCTGATGATGCCTGGATTGAT
GGAGAAGTCACAGAAATTAAAGGCTCAAATGCCACAGTAGCCACAACTAATGGGAAGACGACAGTTGCTTC
TATTTCTAGCATATACCCAAAGGATACCGAAGCACCACCATCTGGGGTTGATGACATGACCAAGTTAGCTTAT
CTCCATGAGCCTGGCGTTCTGAATAACCTTGCTTGTCGGTATTCTCTAAATGAGATATATACTTACACGGGGAA
TATCCTAATTGCGGTCAATCCATTTCGGAGACTTCCACATCTGTATGATACCCATATGATGCAGCAATACAAGG
GAGCCCCATTTGGAGAGCTAAGTCCACATCTTTTTGCAGTGGCAGATGCCTGTTATAGGGCATTGATAAATG
AGCATGGCAACCAGTCTATCTTGGTCAGTGGAGAGAGTGGTGCTGGTAAAACTGAGACAACGAAAATGTTA
ATGAGATATCTCGCGTTTATGGGTGGAAGGTCTGGCACTGAAGGAAGAACAGTCGAGCAACAAGTTTTGGA
GTCCAACCCAGTTTTAGAAGCATTTGGGAATGCAAAGACTGTGAAGAACAACAATTCCAGTCGCTTTGGTA
AATTTGTTGAAATTCAGTTTGATAAGCACGCGAAGATTTCTGGGGCTGCAGTTAGGACATATCTTCTTGAAA
GGTCACGTGTTTGCCAAGTCTCAGACCCAGAGAGAAACTACCATTGTTTTTACATGCTTTGTGCTGCACCAC
CAGAGGATGTGAAAAGATTCAAACTTGGGAATCCAAAATCATTTCATTATCTAAACCAAAGTAGCTGTTACG
AAGTTGCAAATGTTGACGATGCAAGAGAGTATCTTGAAACCAGAAATGCTATGGATGTTGTTGGAATCGGTC
AGGAGGAGCAGGAAGCTATATTTCGTGTTGTAGCTGCAATACTCCATCTCGGAAACATCAACTTTGTGAAAG
GAAAGGAGGCAGATTCCTCCAAACTAAAAGATGAGAAGTCACTTTTTCATCTGAAGACAGCTGCAGAGCTA
TTCATGTGTGATGAGAAGGCACTAGAAGACTCACTTTGTAAGCGTGTCATTGTAACTCCTGATGGAAACATC
ACAAAACTACTGGATCCAGCAGCTGCAACCACGAGCAGGGATGCCCTGGCAAAGACCGTATACTCCAGATT
GTTTGACTGGCTTGTGGACAAGATAAACAATTCAATTGGACAGGATCCTGAAGCAAAAAGCATAATTGGCG
TCCTTGATATATATGGCTTTGAGAGCTTCAAAATCAACAGTTTTGAGCAATTCTGCATCAACCTAACAAATGA
GAAGTTGCAGCAGCATTTTAACCAGCACGTATTCAAGATGGAACAAGACGACTATACGACAGAAGAAATCA
ACTGGAGTTATGTGGAGTTTGTAGATAACCAAGATGTTTTAGATCTTATTGAGAAGAAACCTGGAGGCATTAT
TGCTCTTCTTGATGAAGCTTGTATGTTCCCAAAAGCAACTCATGAGACATTTGCCCAAAAGATGTACCAGACA
TACAGAGCCCATAAACGGTTTAGCAAGCCAAAACTTGCTCGTACTGACTTTACAATCAATCATTACGCTGGTG
ATGTTACTTACCAAGCAGATCACTTCCTTGACAAAAATAAGGACTATGTGATAGCAGAATTTCAAGCTCTTCT
GATGGACTCAAAGTGCTTTTTCGTTGCAAACCTCTTCCCTCCATTGCCCGAAGAATCGTCCAAGCAGTCCAA
GTTCTCATCCATCGGCACACGGTTTAAGCAACAACTGCAATCTTTAATGGAGACCTTGAGCACTACAGAGCC
ACATTACATCAGATGTGTAAAGCCCAATACAGTTCTAAAGCCAGGAATATTTGAGAACATGAATGTATTAAAC
CAATTAAGATGTGGGGGTGTCTTAGAGGCGATCAGGATAAGTTGTGCAGGATACCCAACGAAAAGAACATT
CGATGAGTTCCTTGACCGCTTTGGAACGTTAGCTCCAGATGTTCTTGACGGATGTGATGAGAAGTCAGCATG
CATTGCAATTTGTGATAGAATGGGCTTAAAGGGTTATCAGATTGGGAAAACCAAAGTTTTTCTCAGAGCCGG
GCAGATGGCTGAATTAGATGCCAGAAGAACAGAAGTTCTAGCTCATGCTGCAAAGCGCATTCAGAGACAAA
TTCGAACATATCTTACCCGAAAGGAGTTCATAGCCCTAAAGAGAGCTACAATTCATTTCCAGAAACTTTGGA
GAGCACAACTTGCCAGAGTGCTGTATGAACAAATGAAAAGGGAAGCTGCTTCAATCCGCATACAGAAACAT
GCGCGTTCTCATTCAGCAAGAAAATCTTACAAGGAGTTACAGGCAGCAGCTGTAGTCATTCAAACAGGGAT
GAGAGCTATGGCAGCACGAAATGAATATAGGCAGAGGAGGAGAAATAAAGCAGCTAAAATAGTTCAGACTC
AATGGAGAGGATTCCATGCCTTCTCAACTTATAAACAGAAGAAAAAAGCAAGTCTTTCACTTCAATGTCTCT
GGAGAGGCAGGTTAGCGAGGAAGGTGCTTCGGAAATTGAGGATGGATGCAAGAGATACTGGGGCACTTA
AAGAAGCAAAGGACAAACTGGAAAAGCGTGTCGAGGAGTTAACATGGCGATTAGATTTTGAAAAACACTT
GAGGATTGATCTCGAAGAAGCAAAGGGGCAAGAAATTTCAAAATTGCAAAAAGCCTTACAAGAAATGCAA
ACACAACTAGACGAAGCCCATGATGCAATTATTCATGAGAAAGAAGCAGCAAAGATAGCCATTGAACAAGC
TCCTCCAGTTATCAAGGAGGTACCAGTGATTGACAACACCAAAGTGGAGAAGCTGACAGAGGAAAACAAC
AAACTCGAGGAAGAAATCAGAGAGCTCAAGAAGAGAGTAGAAGACTTCGAACAGAGCTACAACGAAGTT

GAAAAAGAATGCCAGGCCACACGTAAAGAGGCAGAAGAATCTCAGCTAAGAGTTTCGGAGTTCCAAGAGT
CCATAGAAAGATTACAACTGAATCTATCCAACCTTGAATCAGAGAATCAGGTTCTCCGTCAGCAGGCTTTAGT
AGCATCAACAAATGAGGCCCTTTCTGATGAAATGGACATACTCAAGAACAAGATTAAAGACTTGGAGTCAG
AAAACGAGCTTCTCCGCACTCAAAGGGTAGTTGTGGAGCAAGTAGTAAGTTCTGATGATCGGGCACCTAAA
GGGCTTGAGACCGTTGATATCACACATCCAGCTGATAATGAACATCAAACAGAGGAAGTGCATGAAGAAAT
GAAAGTAGAACAACAAATACCTAAGCTTCTGCAGGATTCTAGCCCTCCCATCTCTTTAACTAAGCAAAGATCC
TTGACAGATAGGCAGCAGGAAAGCCATGATATACTGATAAAGTGCCTTGCGGAAGACAAGCGATTTGACAA
GGGAAGACCAGTAGCTGCATGCACCCTGTACAAAGCACTTCTGCAGTGGAGATCCTTTGAAGCAGAAAAAA
CGAATATATTTGATAGGATTATTCATACCATCCGATCATCTATAGAGGATCAGGATAACACCGGTGATCTAGCCT
ACTGGCTGTCAACATCTTCAACTCTGTTGTTCCTTTTGCAAAGCACAATTAAAGCTGGTAATGCGCCTACTAG
GTCCCCATATCGTAATCGCAGCTCACCAACCACATTGTTTGGAAGAATGGCACAGGGTTTTCGTTCAACTTCA
TTAAGCATGGCAATTTCCAGTGGATACAGCGGAATTGAGGGAAGCCCAAATGTCCGAACAAGGATTGAAGC
TAAATACCCAGCACTATTGTTTAAGCAACACCTAACTGCTTGCGTTGAGAAAATATATGGAATGATCCGCGAC
AACTTAAAGAAAGAAATAAGTCCATTTCTTAACCAATGCATACATGCACCAAGATCTGCAAGGATAAAACCTT
TAAAAGGATCATCTAGAAGTATACATTCAAACATCATCGCGAAACAACAGGCATCCAGCGTACACTGGCAAA
ACATTGTGAATAGCCTAGACAATACATTAACCATACTCTCAGAAAATAATGTCCCTTCCACCATTACAAGGAAA
ATACTCAGTCAGGTGTTCTCATACATAAATGTCCAGCTTTTCAACAGCTTGTTGCTTCGCCGTGAATGCTGCTC
ATTTAGCAATGGGGAATATCTGAAGGCGGGTCTGCAAGAACTGGAAAGTTGGTGTTCCAAAGCAACAGAA
CAGTATGCTGGATCTTCGTGGGATGAACTTCAACACATAAGGCAAGCTGTAGGATTTCTGGTGTTACATCAA
AAATCTCAAAAGGCATTGGATGAGATCACGAATGACCTCTGCCCGATGTTGAGCATTGCACAAATATATCGCA
TTGGAACTATGTTCTGGGACGACAAATATGGAGCCCATGGTTTATCGCCAGAGGTCATAAGCAAGATGAGAG
CACTAACATTGGAAGACTCAGCCAGCATTCCAAATAATACATTCTTGCTTGATGTGGATTCAAGCATACCATTC
TCTATAGAAGAAATATCGCGATCCTTCCAGAATATCAACTTGTCTGATGTGGAACCACCTCCCCTCCTTTGTCA
AAGATCAGATTTTCAGTTCCTGCTGCAGGCAGCAGCATAA
>PGSC0003DMT400079918
ATGGGAACTCCTGTAAACATCATAGTTGGATCCCAGGTGTGGATTGAGGATCCTGATGATGCCTGGATTGAT
GGAGAAGTCACAGAAATTAAAGGCTCAAATGCCACAGTAGCCACAACTAATGGGAAGACGACAGTTGCTTC
TATTTCTAGCATATACCCAAAGGATACCGAAGCACCACCATCTGGGGTTGATGACATGACCAAGTTAGCTTAT
CTCCATGAGCCTGGCGTTCTGAATAACCTTGCTTGTCGGTATTCTCTAAATGAGATATATACTTACACGGGGAA
TATCCTAATTGCGGTCAATCCATTTCGGAGACTTCCACATCTGTATGATACCCATATGATGCAGCAATACAAGG
GAGCCCCATTTGGAGAGCTAAGTCCACATCTTTTTGCAGTGGCAGATGCCTGTTATAGGGCATTGATAAATG
AGCATGGCAACCAGTCTATCTTGGTCAGTGGAGAGAGTGGTGCTGGTAAAACTGAGACAACGAAAATGTTA
ATGAGATATCTCGCGTTTATGGGTGGAAGGTCTGGCACTGAAGGAAGAACAGTCGAGCAACAAGTTTTGGA
GTCCAACCCAGTTTTAGAAGCATTTGGGAATGCAAAGACTGTGAAGAACAACAATTCCAGTCGCTTTGGTA
AATTTGTTGAAATTCAGTTTGATAAGCACGCGAAGATTTCTGGGGCTGCAGTTAGGACATATCTTCTTGAAA
GGTCACGTGTTTGCCAAGTCTCAGACCCAGAGAGAAACTACCATTGTTTTTACATGCTTTGTGCTGCACCAC
CAGAGGATGTGAAAAGATTCAAACTTGGGAATCCAAAATCATTTCATTATCTAAACCAAAGTAGCTGTTACG
AAGTTGCAAATGTTGACGATGCAAGAGAGTATCTTGAAACCAGAAATGCTATGGATGTTGTTGGAATCGGTC
AGGAGGAGCAGGAAGCTATATTTCGTGTTGTAGCTGCAATACTCCATCTCGGAAACATCAACTTTGTGAAAG
GAAAGGAGGCAGATTCCTCCAAACTAAAAGATGAGAAGTCACTTTTTCATCTGAAGACAGCTGCAGAGCTA
TTCATGTGTGATGAGAAGGCACTAGAAGACTCACTTTGTAAGCGTGTCATTGTAACTCCTGATGGAAACATC
ACAAAACTACTGGATCCAGCAGCTGCAACCACGAGCAGGGATGCCCTGGCAAAGACCGTATACTCCAGATT
GTTTGACTGGCTTGTGGACAAGATAAACAATTCAATTGGACAGGATCCTGAAGCAAAAAGCATAATTGGCG
TCCTTGATATATATGGCTTTGAGAGCTTCAAAATCAACAGTTTTGAGCAATTCTGCATCAACCTAACAAATGA

GAAGTTGCAGCAGCATTTTAACCAGCACGTATTCAAGATGGAACAAGACGACTATACGACAGAAGAAATCA
ACTGGAGTTATGTGGAGTTTGTAGATAACCAAGATGTTTTAGATCTTATTGAGAAGAAACCTGGAGGCATTAT
TGCTCTTCTTGATGAAGCTTGTATGTTCCCAAAAGCAACTCATGAGACATTTGCCCAAAAGATGTACCAGACA
TACAGAGCCCATAAACGGTTTAGCAAGCCAAAACTTGCTCGTACTGACTTTACAATCAATCATTACGCTGGTG
ATGTTACTTACCAAGCAGATCACTTCCTTGACAAAAATAAGGACTATGTGATAGCAGAATTTCAAGCTCTTCT
GATGGACTCAAAGTGCTTTTTCGTTGCAAACCTCTTCCCTCCATTGCCCGAAGAATCGTCCAAGCAGTCCAA
GTTCTCATCCATCGGCACACGGTTTAAGCAACAACTGCAATCTTTAATGGAGACCTTGAGCACTACAGAGCC
ACATTACATCAGATGTGTAAAGCCCAATACAGTTCTAAAGCCAGGAATATTTGAGAACATGAATGTATTAAAC
CAATTAAGATGTGGGGGTGTCTTAGAGGCGATCAGGATAAGTTGTGCAGGATACCCAACGAAAAGAACATT
CGATGAGTTCCTTGACCGCTTTGGAACGTTAGCTCCAGATGTTCTTGACGGATGTGATGAGAAGTCAGCATG
CATTGCAATTTGTGATAGAATGGGCTTAAAGGGTTATCAGATTGGGAAAACCAAAGTTTTTCTCAGAGCCGG
GCAGATGGCTGAATTAGATGCCAGAAGAACAGAAGTTCTAGCTCATGCTGCAAAGCGCATTCAGAGACAAA
TTCGAACATATCTTACCCGAAAGGAGTTCATAGCCCTAAAGAGAGCTACAATTCATTTCCAGAAACTTTGGA
GAGCACAACTTGCCAGAGTGCTGTATGAACAAATGAAAAGGGAAGCTGCTTCAATCCGCATACAGAAACAT
GCGCGTTCTCATTCAGCAAGAAAATCTTACAAGGAGTTACAGGCAGCAGCTGTAGTCATTCAAACAGGGAT
GAGAGCTATGGCAGCACGAAATGAATATAGGCAGAGGAGGAGAAATAAAGCAGCTAAAATAGTTCAGACTC
AATGGAGAGGATTCCATGCCTTCTCAACTTATAAACAGAAGAAAAAAGCAAGTCTTTCACTTCAATGTCTCT
GGAGAGGCAGGTTAGCGAGGAAGGTGCTTCGGAAATTGAGGATGGATGCAAGAGATACTGGGGCACTTA
AAGAAGCAAAGGACAAACTGGAAAAGCGTGTCGAGGAGTTAACATGGCGATTAGATTTTGAAAAACACTT
GAGGATTGATCTCGAAGAAGCAAAGGGGCAAGAAATTTCAAAATTGCAAAAAGCCTTACAAGAAATGCAA
ACACAACTAGACGAAGCCCATGATGCAATTATTCATGAGAAAGAAGCAGCAAAGATAGCCATTGAACAAGC
TCCTCCAGTTATCAAGGAGGTACCAGTGATTGACAACACCAAAGTGGAGAAGCTGACAGAGGAAAACAAC
AAACTCGAGGAAGAAATCAGAGAGCTCAAGAAGAGAGTAGAAGACTTCGAACAGAGCTACAACGAAGTT
GAAAAAGAATGCCAGGCCACACGTAAAGAGGCAGAAGAATCTCAGCTAAGAGTTTCGGAGTTCCAAGAGT
CCATAGAAAGATTACAACTGAATCTATCCAACCTTGAATCAGAGAATCAGGTTCTCCGTCAGCAGGCTTTAGT
AGCATCAACAAATGAGGCCCTTTCTGATGAAATGGACATACTCAAGAACAAGATTAAAGACTTGGAGTCAG
AAAACGAGCTTCTCCGCACTCAAAGGGTAGTTGTGGAGCAAGTAGTAAGTTCTGATGATCGGGCACCTAAA
GGGCTTGAGACCGTTGATATCACACATCCAGCTGATAATGAACATCAAACAGAGGAAGTGCATGAAGAAAT
GAAAGTAGAACAACAAATACCTAAGCTTCTGCAGGATTCTAGCCCTCCCATCTCTTTAACTAAGCAAAGATCC
TTGACAGATAGGCAGCAGGAAAGCCATGATATACTGATAAAGTGCCTTGCGGAAGACAAGCGATTTGACAA
GGGAAGACCAGTAGCTGCATGCACCCTGTACAAAGCACTTCTGCAGTGGAGATCCTTTGAAGCAGAAAAAA
CGAATATATTTGATAGGATTATTCATACCATCCGATCATCTATAGAGGATAACACCGGTGATCTAGCCTACTGGC
TGTCAACATCTTCAACTCTGTTGTTCCTTTTGCAAAGCACAATTAAAGCTGGTAATGCGCCTACTAGGTCCCC
ATATCGTAATCGCAGCTCACCAACCACATTGTTTGGAAGAATGGCACAGGGTTTTCGTTCAACTTCATTAAGC
ATGGCAATTTCCAGTGGATACAGCGGAATTGAGGGAAGCCCAAATGTCCGAACAAGGATTGAAGCTAAATA
CCCAGCACTATTGTTTAAGCAACACCTAACTGCTTGCGTTGAGAAAATATATGGAATGATCCGCGACAACTTA
AAGAAAGAAATAAGTCCATTTCTTAACCAATGCATACATGCACCAAGATCTGCAAGGATAAAACCTTTAAAA
GGATCATCTAGAAGTATACATTCAAACATCATCGCGAAACAACAGGCATCCAGCGTACACTGGCAAAACATT
GTGAATAGCCTAGACAATACATTAACCATACTCTCAGAAAATAATGTCCCTTCCACCATTACAAGGAAAATACT
CAGTCAGGTGTTCTCATACATAAATGTCCAGCTTTTCAACAGCTTGTTGCTTCGCCGTGAATGCTGCTCATTTA
GCAATGGGGAATATCTGAAGGCGGGTCTGCAAGAACTGGAAAGTTGGTGTTCCAAAGCAACAGAACAGTA
TGCTGGATCTTCGTGGGATGAACTTCAACACATAAGGCAAGCTGTAGGATTTCTGGTGTTACATCAAAAATCT
CAAAAGGCATTGGATGAGATCACGAATGACCTCTGCCCGATGTTGAGCATTGCACAAATATATCGCATTGGA
ACTATGTTCTGGGACGACAAATATGGAGCCCATGGTTTATCGCCAGAGGTCATAAGCAAGATGAGAGCACTA

ACATTGGAAGACTCAGCCAGCATTCCAAATAATACATTCTTGCTTGATGTGGATTCAAGCATACCATTCTCTAT
AGAAGAAATATCGCGATCCTTCCAGAATATCAACTTGTCTGATGTGGAACCACCTCCCCTCCTTTGTCAAAGA
TCAGATTTTCAGTTCCTGCTGCAGGCAGCAGCATAA
>PGSC0003DMT400079919
ATGGGAACTCCTGTAAACATCATAGTTGGATCCCAGGTGTGGATTGAGGATCCTGATGATGCCTGGATTGAT
GGAGAAGTCACAGAAATTAAAGGCTCAAATGCCACAGTAGCCACAACTAATGGGAAGACGACAGTTGCTTC
TATTTCTAGCATATACCCAAAGGATACCGAAGCACCACCATCTGGGGTTGATGACATGACCAAGTTAGCTTAT
CTCCATGAGCCTGGCGTTCTGAATAACCTTGCTTGTCGGTATTCTCTAAATGAGATATATACTTACACGGGGAA
TATCCTAATTGCGGTCAATCCATTTCGGAGACTTCCACATCTGTATGATACCCATATGATGCAGCAATACAAGG
GAGCCCCATTTGGAGAGCTAAGTCCACATCTTTTTGCAGTGGCAGATGCCTGTTATAGGGCATTGATAAATG
AGCATGGCAACCAGTCTATCTTGGTCAGTGGAGAGAGTGGTGCTGGTAAAACTGAGACAACGAAAATGTTA
ATGAGATATCTCGCGTTTATGGGTGGAAGGTCTGGCACTGAAGGAAGAACAGTCGAGCAACAAGTTTTGGA
GTCCAACCCAGTTTTAGAAGCATTTGGGAATGCAAAGACTGTGAAGAACAACAATTCCAGTCGCTTTGGTA
AATTTGTTGAAATTCAGTTTGATAAGCACGCGAAGATTTCTGGGGCTGCAGTTAGGACATATCTTCTTGAAA
GGTCACGTGTTTGCCAAGTCTCAGACCCAGAGAGAAACTACCATTGTTTTTACATGCTTTGTGCTGCACCAC
CAGAGGATGTGAAAAGATTCAAACTTGGGAATCCAAAATCATTTCATTATCTAAACCAAAGTAGCTGTTACG
AAGTTGCAAATGTTGACGATGCAAGAGAGTATCTTGAAACCAGAAATGCTATGGATGTTGTTGGAATCGGTC
AGGAGGAGCAGGAAGCTATATTTCGTGTTGTAGCTGCAATACTCCATCTCGGAAACATCAACTTTGTGAAAG
GAAAGGAGGCAGATTCCTCCAAACTAAAAGATGAGAAGTCACTTTTTCATCTGAAGACAGCTGCAGAGCTA
TTCATGTGTGATGAGAAGGCACTAGAAGACTCACTTTGTAAGCGTGTCATTGTAACTCCTGATGGAAACATC
ACAAAACTACTGGATCCAGCAGCTGCAACCACGAGCAGGGATGCCCTGGCAAAGACCGTATACTCCAGATT
GTTTGACTGGCTTGTGGACAAGATAAACAATTCAATTGGACAGGATCCTGAAGCAAAAAGCATAATTGGCG
TCCTTGATATATATGGCTTTGAGAGCTTCAAAATCAACAGTTTTGAGCAATTCTGCATCAACCTAACAAATGA
GAAGTTGCAGCAGCATTTTAACCAGCACGTATTCAAGATGGAACAAGACGACTATACGACAGAAGAAATCA
ACTGGAGTTATGTGGAGTTTGTAGATAACCAAGATGTTTTAGATCTTATTGAGAAGAAACCTGGAGGCATTAT
TGCTCTTCTTGATGAAGCTTGTATGTTCCCAAAAGCAACTCATGAGACATTTGCCCAAAAGATGTACCAGACA
TACAGAGCCCATAAACGGTTTAGCAAGCCAAAACTTGCTCGTACTGACTTTACAATCAATCATTACGCTGGTG
ATGTTACTTACCAAGCAGATCACTTCCTTGACAAAAATAAGGACTATGTGATAGCAGAATTTCAAGCTCTTCT
GATGGACTCAAAGTGCTTTTTCGTTGCAAACCTCTTCCCTCCATTGCCCGAAGAATCGTCCAAGCAGTCCAA
GTTCTCATCCATCGGCACACGGTTTAAGCAACAACTGCAATCTTTAATGGAGACCTTGAGCACTACAGAGCC
ACATTACATCAGATGTGTAAAGCCCAATACAGTTCTAAAGCCAGGAATATTTGAGAACATGAATGTATTAAAC
CAATTAAGATGTGGGGGTGTCTTAGAGGCGATCAGGATAAGTTGTGCAGGATACCCAACGAAAAGAACATT
CGATGAGTTCCTTGACCGCTTTGGAACGTTAGCTCCAGATGTTCTTGACGGATGTGATGAGAAGTCAGCATG
CATTGCAATTTGTGATAGAATGGGCTTAAAGGGTTATCAGATTGGGAAAACCAAAGTTTTTCTCAGAGCCGG
GCAGATGGCTGAATTAGATGCCAGAAGAACAGAAGTTCTAGCTCATGCTGCAAAGCGCATTCAGAGACAAA
TTCGAACATATCTTACCCGAAAGGAGTTCATAGCCCTAAAGAGAGCTACAATTCATTTCCAGAAACTTTGGA
GAGCACAACTTGCCAGAGTGCTGTATGAACAAATGAAAAGGGAAGCTGCTTCAATCCGCATACAGAAACAT
GCGCGTTCTCATTCAGCAAGAAAATCTTACAAGGAGTTACAGGCAGCAGCTGTAGTCATTCAAACAGGGAT
GAGAGCTATGGCAGCACGAAATGAATATAGGCAGAGGAGGAGAAATAAAGCAGCTAAAATAGTTCAGACTC
AATGGAGAGGATTCCATGCCTTCTCAACTTATAAACAGAAGAAAAAAGCAAGTCTTTCACTTCAATGTCTCT
GGAGAGGCAGGTTAGCGAGGAAGGTGCTTCGGAAATTGAGGATGGATGCAAGAGATACTGGGGCACTTA
AAGAAGCAAAGGACAAACTGGAAAAGCGTGTCGAGGAGTTAACATGGCGATTAGATTTTGAAAAACACTT
GAGGATTGATCTCGAAGAAGCAAAGGGGCAAGAAATTTCAAAATTGCAAAAAGCCTTACAAGAAATGCAA
ACACAACTAGACGAAGCCCATGATGCAATTATTCATGAGAAAGAAGCAGCAAAGATAGCCATTGAACAAGC

TCCTCCAGTTATCAAGGAGGTACCAGTGATTGACAACACCAAAGTGGAGAAGCTGACAGAGGAAAACAAC
AAACTCGAGGAAGAAATCAGAGAGCTCAAGAAGAGAGTAGAAGACTTCGAACAGAGCTACAACGAAGTT
GAAAAAGAATGCCAGGCCACACGTAAAGAGGCAGAAGAATCTCAGCTAAGAGTTTCGGAGTTCCAAGAGT
CCATAGAAAGATTACAACTGAATCTATCCAACCTTGAATCAGAGAATCAGGTTCTCCGTCAGCAGGCTTTAGT
AGCATCAACAAATGAGGCCCTTTCTGATGAAATGGACATACTCAAGAACAAGATTAAAGACTTGGAGTCAG
AAAACGAGCTTCTCCGCACTCAAAGGGTAGTTGTGGAGCAAGTAGTAAGTTCTGATGATCGGGCACCTAAA
GGGCTTGAGACCGTTGATATCACACATCCAGCTGATAATGAACATCAAACAGAGGAAGTGCATGAAGAAAT
GAAAGTAGAACAACAAATACCTAAGGATTCTAGCCCTCCCATCTCTTTAACTAAGCAAAGATCCTTGACAGAT
AGGCAGCAGGAAAGCCATGATATACTGATAAAGTGCCTTGCGGAAGACAAGCGATTTGACAAGGGAAGAC
CAGTAGCTGCATGCACCCTGTACAAAGCACTTCTGCAGTGGAGATCCTTTGAAGCAGAAAAAACGAATATAT
TTGATAGGATTATTCATACCATCCGATCATCTATAGAGGATAACACCGGTGATCTAGCCTACTGGCTGTCAACA
TCTTCAACTCTGTTGTTCCTTTTGCAAAGCACAATTAAAGCTGGTAATGCGCCTACTAGGTCCCCATATCGTAA
TCGCAGCTCACCAACCACATTGTTTGGAAGAATGGCACAGGGTTTTCGTTCAACTTCATTAAGCATGGCAAT
TTCCAGTGGATACAGCGGAATTGAGGGAAGCCCAAATGTCCGAACAAGGATTGAAGCTAAATACCCAGCAC
TATTGTTTAAGCAACACCTAACTGCTTGCGTTGAGAAAATATATGGAATGATCCGCGACAACTTAAAGAAAG
AAATAAGTCCATTTCTTAACCAATGCATACATGCACCAAGATCTGCAAGGATAAAACCTTTAAAAGGATCATC
TAGAAGTATACATTCAAACATCATCGCGAAACAACAGGCATCCAGCGTACACTGGCAAAACATTGTGAATAG
CCTAGACAATACATTAACCATACTCTCAGAAAATAATGTCCCTTCCACCATTACAAGGAAAATACTCAGTCAGG
TGTTCTCATACATAAATGTCCAGCTTTTCAACAGCTTGTTGCTTCGCCGTGAATGCTGCTCATTTAGCAATGGG
GAATATCTGAAGGCGGGTCTGCAAGAACTGGAAAGTTGGTGTTCCAAAGCAACAGAACAGTATGCTGGATC
TTCGTGGGATGAACTTCAACACATAAGGCAAGCTGTAGGATTTCTGGTGTTACATCAAAAATCTCAAAAGGC
ATTGGATGAGATCACGAATGACCTCTGCCCGATGTTGAGCATTGCACAAATATATCGCATTGGAACTATGTTCT
GGGACGACAAATATGGAGCCCATGGTTTATCGCCAGAGGTCATAAGCAAGATGAGAGCACTAACATTGGAA
GACTCAGCCAGCATTCCAAATAATACATTCTTGCTTGATGTGGATTCAAGCATACCATTCTCTATAGAAGAAAT
ATCGCGATCCTTCCAGAATATCAACTTGTCTGATGTGGAACCACCTCCCCTCCTTTGTCAAAGATCAGATTTTC
AGTTCCTGCTGCAGGCAGCAGCATAA
>PGSC0003DMT400050070
ATGGGGAAGGCAAGCAAATGGATCAGAAACTTCCTAATGGTGATGGGGAAAAAGGAGGAAAGGGAAAAG
AAAGAATACAAATCAATAGAAAGTATGGGCACCCCAACAACTCCAAAGGCGAAAAGGAGATGGAGTTTCAA
GAAATCATCAAGCATGGAGAGAAAAAGCCACAAGAGTAACAGGTCTTTCGACTTAACTTTTGATCACCAAC
TAAACACACAAGGTTCAATGCTGGAGTTTGACATGCTAGAGAAACACCACAAAGCAAGCCTAACAGCAAAA
GGAGCTATAAAACCAAAGGCATACCTTACCAGACGTGTTAAGGATGCAGCTGCCACCAAAATCCAAGCTGTT
TTCCGTGCTTATTTGGCTAGGAAAGCATTGCGCGCCCTAAGAAGCCTAGTTAGATTACAGGCACTGGTAAGG
GGTCACCTAGTGAGGAAACAGACAGCAGCAATGGTGAGACGCATGCACTCTCTTATGGTCATTCAACTAAG
GGCTCGTGTTCAAAGAGTTCAGATGACCAAAGAAGCACATACCCCAAACTCAAGAAAGAGTCAGAAAGTAT
CACCTGGGAACAATCAACTTACCAGAGCCTGCAGCATTGAAAAGATGGATGTTAGCATTCAAGAAAAAGGG
AGAGTTCAGAAGAACAATAGCACGAAAAACAGATCTTCTGGAATGGAAAATGGATTGAGCACCTCTGAGTC
TCGTTGCCTTTCACTGTCAAGGAGAAGTCATCAAGTACTTCAGACATGTCCAAGTCCCTCTACACTGAGTGAT
ATGAGCACAATAAGTTATGACAGGCATTTAGAGGATTTTTCCTTCAAGACGCCAGAAAAAGGTTTTGAACAT
TGCTCTAATGTGTCAACAACCACATCTTCCAAAACCCCATTTTCCATTCCACACTCAGAAAAACCGAACTCCAT
TTTTTCTAGTGCTACTTTAGCACTAACATACATGTCCAATACAGAATCCTCAAGAGCAAAAGCTAGGTCACATA
GTGAACCCAGACAACGACCCAATTGGAGCATTATAAGAAAAAGCAAGCGCACACCATCAATGGATGGGATA
ACAGGCATACCTGACTCTAGAAGGGAAGAAACACCTACTCACAGCAGGAGACACAATGTCCCAGAAAGCC
ATGAAGCCTGGTTACTTAAGCTCTACAAACAAGCAAAGTCTATCAAGCATGTCAAGGTTGATTCCGCCAGCA

TAGTGTCCGCCGTTTGA
>PGSC0003DMT400045444
ATGGGAAAATCTCCTGGAAAGTGGCTCAGGTCATTGCTGCCAGGGAAAAAGTCTTCCAAATCTGGCACATC
AAAGAAATCTTCAAATGAGAAAGCATCTGTAATTTCCACCAATGCGGCATTGTCCGGTTCGTCTGTCCATCTG
CCATTGATATCTGAACCAGTTGCTGGTAATTCTGGTGGAATAAAAGAAGACTCGAACTTTGAGAAAGGAGA
GGTCACTGATGAGGTGATTCTCCCTTCTATTGAGCGAGATGGAGACGAACAAAATACTTGTCTTACCTTACCC
GAGGATACTGAGAAAATGAGGCTTGAGCAAGCTGCTATGAAGGCACAAGCTATTGTTAGGGGTTATCTGGC
TCGCCGAGCATTTCTCAGGCTCAAGGGGACCATAAGGCTACAAGCTGCAGTACGTGGCCATCTGGTCAGAA
GGCAGGCTGTTGCTACATTATACTGTATACATGGCATTGTCAAACTTCAAGCACATATCCGTGGCCAGATTATT
AGACGTTCCAGTATTGGTTGTGAATTGATAACCAAACAAGGACTTGAAAAACAGGATGCTAAACAATTGGAT
TATCAGAGAACTAATGCATCCAAACTAGCACGGGAGCTATCCAAGAATGAATTCACTACAAAGCTACTTGCTT
CATCACCTACTGTAATGCCTCTACACCTCCATTATGGTCCAGAGGAACCAAATTCTAGTCAGGAATGGCTAGT
CCGTTGGACTATATCACAGATTTGGCAACCACAACCTAAATCGGAAACACTTTCAAGAAAAAAGCATCAAAA
TGTTGAAGCAGACATTGCTATGTCAAAGCACAGTGGGAGGAAACTACATTCTAGAAAAATGCAGAACGGTT
CAAATCATTCCACTTCTTCAGGGTCAGAAAAGAAGAAATCAAGTCATCTGGTAAACTCTGTTCTTCAGAATCC
TGGAAGTGAGATTAAGAAGGTGAAACATAGTGTAAAGAAAATGTCCAGCCCTATATTGGAAAAACCAATTCA
GTCTGAGGTTGATACTGAGCGAAAAAGGCAAAGTCATGACAAATTATCAAGCATGACATCTGATGAACCATT
GCAAAATTCAGAAGGGATAGTTGAAAATTCTACTAATGTGGCCCCATCTCAAGAAACGCTAGGGGTGGATG
ATACTATCTCTCGTTTGGATATCCTTTCTGTATCTGATACTCTTCACAAATCAACTACTGATGCTGCGTATCAAAA
ACCAATCACTGATAACCAAGAGGATGATACTCCTGTTGCAAATGAGGATTCTTGTACTAACCATGATAATAATG
AGGGGAATGAGAGCAACAAGGTTAACAGAAGAGTTTCTTTACCTGCAAAGCATGATGTCGATGCAAGTACG
CCAACTACGCCAACGACAAGAAAGGTGCCCAGCTACATGGCTCCAACCAAGTCTGCTAAAGCTAAGCTGAA
AGAGCAAGCCTCACCAAGGTTTGGGCAAGATGTGGCTGAGAAGAATGCCGTAACCAGACGTCATTCTTTGC
CGTCCCCTATGAATGGAAAGCTGAGTTCATCACCATCACCACGGGTACAGAGGCTGGTCCAAGCTAGTGCCA
AAGAAGGAATCAAGATCGATAGATCTTTATCATCTTCAAGGGATGGTACTGATAAGATGACTCGAGCAGAAT
GGAAGCGGTAA
>PGSC0003DMT400084738
ATGTGGAAGGATATGCTGGATCACTATGGGGTTTCTGCATCTGCTGAATCACAAACCAAATATTTGCACAAGT
TGGATGAGAATGCAATGCTTCAAACCTCATCAGAGAGAAGGGCCATAGAAGCATATGAAAGTTACAAGTGG
TGTGATTTCAGTGACAGGGAGGCTCAGACGGCTCCTGTACCAGCTTTTAAGCAACTCGAAGATTTCAAGTAT
ACTACATATCCTCCTGCTATCACTACTTTTGGATCCAACCCTGACGAATATACAACAATATTTGACCAAGATCAG
ATTGGTACTTCTCTTGAAGATGAGATGAGCTTAACTATTGCTCAGACGCAGAAATTTACTATTCGACATATATC
CCCTGATTGGGGGTATTCATCTGAGGCAACAAAGATTGTTATCATTGGATCTTTTCTTTGTAATCCTTCAGAGT
GCACGTGGACTTGCATGTTCGGTGATATTGAAGTTCCTGTTCAGATCATTCAAGAAGGTGTCATCTGTTGTCA
AGCTCCTCGTCACTTGCCTGGTAAGGTCACCCTCTGTGTTACTAGCGGCAATCGGGAGTCGTGCAGTGAGGT
GAGGGAGTTTGAATACCGGGTAAAGCCTGATGATTGTGCTCGAAATAATCAGCCTGATGTAGAAGGAGCTT
ATGGAAGTACAGAGGAACTGTTGTTACTTGTCAGGTTTGTGCAGCTGCTTTTATCAGACTTATCTGTCCAAAA
GGGAGAAAGCTCTGAATTAGGCAATGATTTCTTGGAAAAATCCAAAGCAAGTGAAGATTCATGGTCCCAGA
TCATTGAATCTCTTTTGTTTGGATCTTCAATGCCAATGGTAACCATTGATTGGCTTCTTCAAGAGCTTTTGAAA
GACAAGTTCCAGCAGTGGCTTTCTTGTAAATTGCAACAAAAAGATAATCAGATAGGCTGTTCTTTATCTAAGA
AAGAGCAAGGAGTAATTCACATGGTTGCTGGATTGGGATTTGAGTGGGCGCTGCACCCAATTCTAAATGCT
GGAGTCAGTGTTAACTTCCGTGATATTAACGGCTGGACGGCCCTGCACTGGGCTGCACGCTTTGGGAGGGA
AAAAATGGTTGCTTCGCTCATTGCATCTGGGGCATCGGCTGGAGCTGTTACAGATCCCTCTTCACGAGATCC
AGTTGGTAAAACTGCTGCATCAATTGCTTCCAGCTGTGATCATAAGGGACTTGCAGGATATCTTTCAGAGGTA

GCTCTCACCAGTCATCTTTCATCCCTCACTTTAGAGGAGAGTGAGCTCTCAAAAGGTACTGCTGATGTGGAA
GCAGAGAGAACTATTAGTAGCATATCAAACACAAGTGCCACCATAAATGAGGATCAGCGTTCTTTAAACGAC
ACGTTAGCTGCAGTCCGTAATGCAGCTCAGGCTGCTGCTCGTATACAGTCTGCATTCCGAGCACATTCGTTCC
GCAAAAGACAAGAGAGAGAATTTGGTGTCTCTGCTAGTGGAGATGAATATGGTATCCTCTCAAACGATATTC
AGGGGCTTTCAGCTGCATCAAAGTTGGCATTCCGCAACCCGCGAGACTACAACTCAGCAGCCTTAGCTATTC
AGAAGAAATATCGAGGATGGAAAGGCCGGAAAGATTTCCTTGCATTCCGCCAGAAAGTAGTGAAGATACAG
GCTCATGTACGAGGCTATCAGGTTAGAAAGCAATACAAGGTATGTTGGGCTGTTGGAATCTTGGAGAAGGT
GGTGCTAAGGTGGCGTCGACGAGGTGTTGGTCTCCGGGGATTTCGACATGACACAGAATCAATTGATGAAA
TTGAGGATGAAGACATTCTAAAGGTGTTCCGCAAACAAAAAGTTGATGCTGCTCTTGACGAGGCTGTCTCG
AGAGTTCTATCAATGGTTGAATCTCCAGGGGCACGTCAGCAATATCATCGGATTCTTGAGAAGTATCGGCAA
GCTAAGGCTGAACTTGAAGGAGCGGACAGTGAAACAGCATCAACTGCTCATGGAGACATGTCTAACATGGA
AAATGATGACATATACCAGTTCCCTAGTTATTAA
>PGSC0003DMT400011979
ATGACCGTCCAAAATCATGAACAGAGACTACTCGAGATTAACACACTTGAATGGGATGATCTGTTGGCACCT
GGAGATCCCAACAAGATAGTTGCTACCCAGCAAGGAAGTAAAACTGCTTATGTACAGCACACGTCGTATGAG
CAACACAATCTATGTGAATTAAATGGTTACAGCCTCAATGGTGTCTCCTCTTCTCTTGAGAGAATTTCTACAGT
CAACAATTCAAATGAGATCATCTTCCAGACAGTGGATGGTCAAATGACTCCAAGTTTTGAGAAGAATGAGTC
TGGAGTAATGACAGTAAGCACAGGTGATTCCTTTGATAGTCTGAACCAGGATAGACTTCAAACTCAGGATAG
CTTTGGAAGGTGGATGAACTACTTTATTACTGATTCTCCAGAATCCACAGACGATCCAACTCTCGAATCTTCA
GTGTCAACAGGTCAATCATATGCAAGGGAGCAGACATTCAACATAACAGAAATATCGCCTGCGTGGGCTTCT
TCAACTGAAGAAACAAAGATCATTGTCATTGGGCAATTTCATGGAGAACAATCACATCTGGAAAGTTCCTGC
TTGCATTGTGTTTGTGGAGATGCTTGTTTTCCTGCAGAAGTTCTGCAACCTGGGGTATATCGTTGCATAGTTT
CTCCTCAAACCCCTGGACTGGTAAACATATATTTAAGTTTCGATGGTAATAAACCTATTAGTCAAGTCATGAGC
TTTGAGTTTCGAGCTCCTTCAGTACAAGTTTGGACAGAACCCCCGGAGAGTAAATCTGATTGGGATGAATTT
AGAAATCAAATGAGGCTTGCTCATCTGCTGTTCTCTACATCTAAGAGCCTTAATATTCTATCCAGTAAAATACAT
CAAGATTTGCTGAAAGATGCAAAAACATTTGCTGGAAAATGTTCTCACATCATTGATGATTGGGCCTGTCTG
ATCAAATCAATTGAAGACAAGAAAGTCTCTGTCCCACGTGCAAAAGACTGCTTGTTTGAGCTCTCTTTGAAA
ACCAGATTACAGGAATGGCTGTtGGAAAGAGTTGTCGAAGGATGTAAAATCTCAGAACATGACGAGCAAGG
TCAAGGAGTTATCCATTTGTGTGCTATCCTAGGTTACACTTGGGCTGTATATCTGTTTTCCTGGTCCGGTTTGT
CATTGGATTATCGAGACAAATATGGATGGACAGCTCTCCATTGGGCTGCATATTATGGAAGGGAAAAAATGG
TTGCAACTCTTTTATCTGCTGGAGCAAAACCAAATTTGGTCACAGATCCCACTTCAGAAAACCTTGGTGGAT
GTACTGCTTCCGATCTTGCATCTAAAAATGGTCATGAGGGTTTGGGGGCTTATCTCGCTGAGAAGGCTTTAG
TTGCACAATTTAATGATATGACATTAGCTGGAAATATCAGTGGTTCACTCCAGACGACTACTGAGTCAATAAA
CCCCGGGAACTTCACAGAGGAAGAGCTAAATCTGAAAGACAGTTTAGCAGCTTATCGTACAGCTGCAGATG
CAGCTGCACGTATCCAGGCTGCTTTCAGGGAGCGTGCACTGAAAGTGCGGACAGAGGCAGTCGAGTCTTC
AAATTCAGAAATGGAAGCACGTAATATAATTGCAGCTATGAAGATTCAGCATGCTTTCCGCAACTATGAGATG
CAGAAACAGCTGGCAGCTGCTGCACGAATACAGTATAGGTTTCGAACTTGGAAGATGAGGAGAGAGTTTCT
TCACATGCGCCGTCAGGCCATCAAAATTCAAGCTGTGTTCCGGGGTTTCCAAGTCCGAAGGCAGTACAGGA
AGATAACTTGGTCAGTAGGAGTGCTTGAAAAGGCAATATTTCGGTGGCGTTTGAAGAGGAAAGGTCTCCGC
GGGCTTAAACTCCAGTCTAGTCAAGTAGTTAAATCAGATGATGCGGAGGAGGATTTCTTCCAAGCCAGTAG
GAAACAAGCTGAAGAGCGTATTGAAAGATCTGTTGTGCGAGTTCAGGCCATGTTTCGTTCAAAGCAAGCAC
AAGAACAATATAGGAGGATGAAGTTGGAACATAATAAAGCAATGCTGGAATACGAAGGGACTCTCAATCCT
GACACTGAGATGGACTAA
>PGSC0003DMT400011981

ATGAGCTTTGAGTTTCGAGCTCCTTCAGTACAAGTTTGGACAGAACCCCCGGAGAGTAAATCTGATTGGGAT
GAATTTAGAAATCAAATGAGGCTTGCTCATCTGCTGTTCTCTACATCTAAGAGCCTTAATATTCTATCCAGTAA
AATACATCAAGATTTGCTGAAAGATGCAAAAACATTTGCTGGAAAATGTTCTCACATCATTGATGATTGGGCC
TGTCTGATCAAATCAATTGAAGACAAGAAAGTCTCTGTCCCACGTGCAAAAGACTGCTTGTTTGAGCTCTCT
TTGAAAACCAGATTACAGGAATGGCTGTtGGAAAGAGTTGTCGAAGGATGTAAAATCTCAGAACATGACGA
GCAAGGTCAAGGAGTTATCCATTTGTGTGCTATCCTAGGTTACACTTGGGCTGTATATCTGTTTTCCTGGTCCG
GTTTGTCATTGGATTATCGAGACAAATATGGATGGACAGCTCTCCATTGGGCTGCATATTATGGAAGGGAAA
AAATGGTTGCAACTCTTTTATCTGCTGGAGCAAAACCAAATTTGGTCACAGATCCCACTTCAGAAAACCTTG
GTGGATGTACTGCTTCCGATCTTGCATCTAAAAATGGTCATGAGGGTTTGGGGGCTTATCTCGCTGAGAAGG
CTTTAGTTGCACAATTTAATGATATGACATTAGCTGGAAATATCAGTGGTTCACTCCAGACGACTACTGAGTC
AATAAACCCCGGGAACTTCACAGAGGAAGAGCTAAATCTGAAAGACAGTTTAGCAGCTTATCGTACAGCTG
CAGATGCAGCTGCACGTATCCAGGCTGCTTTCAGGGAGCGTGCACTGAAAGTGCGGACAGAGGCAGTCGA
GTCTTCAAATTCAGAAATGGAAGCACGTAATATAATTGCAGCTATGAAGATTCAGCATGCTTTCCGCAACTAT
GAGATGCAGAAACAGCTGGCAGCTGCTGCACGAATACAGTATAGGTTTCGAACTTGGAAGATGAGGAGAG
AGTTTCTTCACATGCGCCGTCAGGCCATCAAAATTCAAGCTGTGTTCCGGGGTTTCCAAGTCCGAAGGCAGT
ACAGGAAGATAACTTGGTCAGTAGGAGTGCTTGAAAAGGCAATATTTCGGTGGCGTTTGAAGAGGAAAGG
TCTCCGCGGGCTTAAACTCCAGTCTAGTCAAGTAGTTAAATCAGATGATGCGGAGGAGGATTTCTTCCAAGC
CAGTAGGAAACAAGCTGAAGAGCGTATTGAAAGATCTGTTGTGCGAGTTCAGGCCATGTTTCGTTCAAAGC
AAGCACAAGAACAATATAGGAGGATGAAGTTGGAACATAATAAAGCAATGCTGGAATACGAAGGGACTCTC
AATCCTGACACTGAGATGGACTAA
>PGSC0003DMT400028274
ATGAAAAAAGAAGCTGCTTCTATCAAAATCCAGACAAAATTGCGTGGACACTTGGCCCGAAAGTCCTACAC
AGGACTCAAAATCAATGTCATTGCTCTTCAGACAGGAATTCGAGCGACAGCTGCACGAAAGGAATTCAGAT
ATAAAAGACAAACCAAAGCAGCAATTAATATACAAGCACATTGGCATGGTCACAGAGCCTTTTCGTACTACA
AGAAACTCATAATTGCATCAATTGTAACACAATGCAGATGGAGGGGAAGGGTTGCAAAGAAAGAGCTTCGG
AAACTAAAGATGGCTTCAAGAGAAACAGGTGCACTAAAAGAAGCAAAGGATAAGCTTGAAAAACAGGTTG
AAGAACTTACATGGCGTCTGCAGTTGGAGAAACGTCTAAGGACGGACTTGGAAGAGGCAAAAAGCCAGG
AAATAGCAAAGCTGAAGAATACCTTGGAAGATGTGCATAGCAAAGTGGATCAAACGAATGCACTGCTCATCA
AGGAACGTGAGACTGCTCAGAAAGCAATTGAAGAAGCAACCTCTATTGTGGAAGAGAAGCCGGTTCTTGT
TGAGGATACAGAAAAGATTGATGCTTTAAATGCAGAAGTGGAAAATTTAAAGGTGTTATTGCAATCTGAAAA
GCAACGTGCTGATGATTCTGAAAGGAAATGTGCTGAATCTCAAGAATCAAGTGAAGAAAAGCACAAGAAGT
TGGAAGAAACTGAAAGAAAAGTTCAACAATTTCAGGAATCAATGAGCAGGCTCGAAGAAAAGCTCACCAA
CATAGAGTCAGAAAATAAAGTACTTCGCCAGCAGGCTCTGACAATGGCACAGAACAATAAATTGCTGTCGG
GACGGTCAAGATCAAGTATTCAGAGGAATGAGAGCAGCACAAGGAATAGCGTAGATCTGCATAGCACTTCA
TTTTCAAGGGAGAGTGCTGAGGTGGAGGGAAGACCACAGAAATCACTTAATGATAAACAACAGGAGTATC
AGGACTTGATCATCCGGTGTATTGCACAGCACCTTGGCTTCTCTAAAGGCAGACCTGTTGCTGCCTGCATTAT
CTACAAGTGCCTTAGGCAGTGGCGATCATTTGAAGTAGAGAGGACCAGTATCTTTGATAGGGTAATACAAAC
CATTGGCCAAGCTATTGAGACTCAGGACAACAATGATATGCTAGCCTATTGGCTgTCCAATGCATCCACTCTAT
TGCTGCTGCTGCAACGTACATTAAAAGCTGGTGGTGCTGCTGGGATGACCCCACAGCACAGGCGATCATCAT
CAGCCTCTCTATTTGGGAGAATGACACAGAGCTTTCGTGGAACCCCTCAAGGTGTCAATATTTCTCTCATTGA
TGGTGATTCAGCCGGTGGAGTGGATACCTTACGCCAAGTAGAGGCCAAATATCCTGCTCTGTTGTTTAAACA
GCAGCTAACAGCATATGTAGAAAAGATTTACGGAATGATCCGTGATAATCTTAAGAAAGAGATTTCTCCTCTG
CTGGGGTTGTGCATTCAGGCACCAAGAATATCCAGAGCAAGTTTACTTAAAGGGACAACAGCACGTACACT
TGCAAATGCAGCTGCTCAGGAAATTTTGATCGCTCACTGGCAAGGAATTGTCAAGAGCCTTGCTAACTTTTT

GAACTTATTGAAAGCCAATCATGTGCCTCCATTTCTTGTGCGCAAAGTATTTACACAAGTCTTTTCTTTCATCA
ATGTCCAATTATTTAACAGCCTTTTGCTGAGAAGAGAATGCTGTTCATTTAGCAACGGTGAATATGTCAAAAC
TGGCTTGGCGGAACTGGAGCACTGGTGCTACAAAGCAACTGATGAGTATACAGGATTAGCTTGGGAGGAG
CTCAAGCATATAAGACAGGCAATTGGATTCTTGGTCATACACCAGAAGCCAAAGAAGACGTTGGATGAAATA
AGCCATGATCTATGTCCTGTGCTTAGCATTCAACAACTTTACAGAATCAGCACAATGTATTGGGACGACAAAT
ATGGCACACATAGCCTTTCATCAGATGTTATAGCCAATATGCGTGTATTAATGACTGAAGACTCTAACAATGCA
GTCAGCAATTCTTTTTTGCTTGACGATGATTCGAGCATACCATTCTCAATTGATGACCTGTCTAAATCAATGGA
TCAGATTGATATTGCAGACATTGAACCACCCCCACTTATTCGAGAAAACTCAGGCTTCAGTTTCTTATTGCCA
CGTGCAGACTAA
>PGSC0003DMT400050471
ATGGGGAAGAAAGGAAGTGGTAGTTGGTTTTCCACTGTGAAGAAAGTCTTTATTAAACCTTCTTCTAAGGAT
TATTCACCAGCAGATCATAAAAAGAAGGAAAAATTAGAGAACCAATGGCAAGATGAAGCTCCAGAAGTTGA
ACAATTTCCAGCAGAGAGTTCTTCAGATCTTACCAATAATCGAGAGAGCAATGACAATTCATCATCTTCGTTG
GCTGAAGATCGAAATCATGATATTCATGTCATCGAAGCCAGTGTTGCGGTAGCAACTTATACGGCCCCAAAA
ATTGTGAAATTAGATGGATACAACAAAGAGGAAATTGCTGCCACCCTTATTCAATCATATTACAGGGGATATC
TGGCAAGACGTGCTTTGCGTGCCCTGAGGGGATTGGTGAAGCTGCAAGCGCTAGTGAGGGGGCATAGTGT
GCGGAAACAAGCGCAAATAACAATGAGATGTATGCAAGCATTAGTACGGGTACAGTCAAAAGTACGTGCAC
GAAGACTCCAGTTGCTGCAGAGCAAAGTGGAAGAAGTAAAATTGCACTCGATTATTAGAGAAGACCAGCA
CAAGAACAAGAGCCTGTCTAACAAACAAGAGACAGAAGGCTGGGATAACAGGAACCAAAGCATTGAAAA
GATCCTACAAAACACTAGAAGAAAACAACACGCTTATCAGAAATGGTTGCACTCTGATCCAGATGATGATGA
AGAATGTTTTTGCAATGAGCATGAAAATCCACAACAAAGTTGGAATTGGCTTGATAGATGGATGGCTTCTCA
ACATGCTGTGCGACGAGAAGGCTCCTATGTGTCATTGTCCATCACAGATGACATATCAGAGAAGACAGTAGA
ATTGGACCCATTGGGCTCAGAAGATGTCAACTTAGCCCATCACATTCTCAGTCCAGTTGAAAGAAGCCCATAT
TCATCCACTCAGTCCAACATGGATTCTGTCCCTAGCTACATGGCTCCAACTAAATCTGCAAAAGCAAAAATAA
GAAGCCCAGGCCCAATAAAACCTAAAAGCCCACCAGGAGTGGCCCAATGGAATGCAACACCGAAGAAGGT
GACAGCCCGTAGGTGGAGCTATGATTCATCTGCGAGAAGCCCAAACCCAAAAACTTCTGCTAAATGGATGG
CAACCTATAGCCCAGAAACACGTGTACACGACAGGGCCTCACCTATGGGAGCCCCTGCTTGCAGATATAATT
ATAATTAG
>PGSC0003DMT400038143
ATGGAAGCAATAAGAATAAGCTGTGCTGGATATCCTACTCGGAAACCCTTTTATGAATTTTTAGATCGCTTTG
GCATCCTTTCGCCTGAAGTTTTAGATGGAAGTACGGACGAGGTCACTGCATGCACACGCCTTTTAGAGAAA
GTCGGCCTTCAAGGATATCAGATTGGTAAAACAAAAGTGTTTCTAAGGGCTGGGCAGATGGCAGAGCTAGA
CAGTCGCAGGACAGAGGTGTTAGGGAGATCGGCGAGCATCATTCAGAGGAAAGTTCGTTCTCACATGGCTC
GAAGAAATTTTACATTATTACGTCAGTTGGCAAGAAGGATACAATCGATGTGCAGAGGAGAACTTGCTCGGC
GTGTATATGAGAGCTTGCGGAGAGAAGCAGCTTGTCTGAAGATCCAGACAGACATGCGCATGCATCTTGCTA
GGAAGGGTTACAAAGAATTGTGCTCTGCTGCCATTTCAATTCAGACAGGAATGCGTGGGATGGCTGCACGC
AATGAAGTACGATTCAGGAGGCAGACAAAAGCTGCCATTATCATTCAGAGTCATTCTCGTGCATTCTTGGCC
CGGTTGAAATACAAGAAACTCAAGAAAGCTGCTATTACCACTCAATGTGCTTGGAGAGCTAGAGTCGCTCGT
GGGGAACTACGGAAACTTAAGATGGCTGCAAGGGAGACTGGTGCGCTTCAAGCTGCAAAGAATAAATTAG
AGAAGCAAGTTGAAGAATTAACTTGGAGACTACAACTGGAGAAACGCATGAGGGCTGACATGGAAGAAGC
AAAAACACAAGAAAATGCAAAACTGCAATCAGCTTTGCAAGAAGTTCAACTACAGTTCAAGGAAACACAA
GAAATGTTTGTCAAAGAACGTGAAACTACAAAAAGGGCGGCAGAGGAAGTTCCTATTATGCAGGAAGTTCC
TGTTGTTGATCATGAAATGATGAACAAACTTAGTGTTGAAAATGAGAAACTAAAGTCTTTGGTAAGCTCTCTA
GAACAGAAGATTGATGAGACAGAAAAAAAGTACGAAGAGACAAGCAAACTTAGTGAGGAGAGGTTGAGG

CAGGTGTTGGATGCAGAGTCTATAATTGTTCAGTTGAAGACTACTATGCAAAGGTTCCAGGAAAGGAATTTT
GATTTGGAATCTGAGAACCAGATTCTTCAGCAATCTCTGTTAGCCCCTGCCAAGCAGGTTTCAGATCATTCAC
CTAGTCTGTCTTCCAAGGTGCAGATAGAAGAAAATGGCTACCACCTCAAAGAAGAAACTAGAACCAATGATC
CACCAGGTTCAACACCTGCTAAAAAAGTTGAAACCCCTAATAGCAAGTCAAGGAAACCTCCTATTGACCGAC
AACGTGAAGACATTGGTGCACTCATTGACTGTGTGATGAAGGATGTTGGATTCAGTCAAAGCAAGCCTGTT
GCAGCTTTTACCATCTACAAATGTCTTCTTCACTGGAAATCTTTTGAAGCAGAAAAGACTAGCGTGTTTGATC
GTTTAGTGCAGATGATTGGTTCTGCCATTGAGAATCAAGATAGTGATGACCACATGGCATACTGGCTGTCAA
ATACCTCAACATTGTTATTGTTAATCCAAAAAAGCCTGAAGCCAGACAGTGCAGTTGGTGCGACTCCTACCC
GTAAACcACAGCCTGCAACATCCCTGTTTGGGAGAATGACATTGGGATTTCGCTCGTCCTCTTCTGACATCAA
TCTTGCTGGAGTAGTGCACCAGGTTCAAGCAAAATACCCAGCTCTGCTTTTCAAACAGCAGCTTACAGCATA
TGTTGAGAAAATGTATGGAATTATTCGTGATAACTTGAAGAAGGAGTTAGGGTCACTTCTTTCCTTATGTATC
CAGGCACCAAGGACCTCCAAAGGAAGTGTGCTAAAGTCTGGTCGATCCTTTGGCAAAGATTATTCAATAAAT
CACTGGCGAGGGATAATTGAATGCCTTGATTCTCTTCTCTGTACTTTGAAAGAAAACTTTATGCCTCCGATTCT
TGTTCAGAAGATATTTAGTCAAGCCTTTTCATACATGAATGTACAGCTCTTTAACAGTTTTCTTCTTCGCCGGG
AGTGTTGTACATTCAGTAATGCAGAATATGTTAAATCTGGCTTGGCTGAGCTAGAGCTGTGGTGCTCCCAAGC
AAAGGAAGAGTATGCTGGCTCATCCTGGGATGAACTCAGACATATACGACAAGTTGTTGGATTCTTGGTTAT
ACATCAGAAGTACAGAATTTCGTATGATGATATCACTAATGATTTGTGTCCTGTTCTCAGTGTCCAGCAACTTT
ACAGAGTATGTACTCTTTACTGGGATGACAAATACAATACACGAAGTGTCTCCCCAGATGTCATATCTAACATG
AGGGTACTTATGACAGAGGACTCGAACGATGCCAAGAGCAACTCTTTTTTGTTAGATGATAACCCAAGCATC
CCATTCTCAATTGAAGAAGTTTCAAATTCACTTCAAGTCAAGGATTTCGCAGATGTCAAACCTGCAACCAAA
CTTCTTGAGAATCCAGCCTTCCAATTTTTACATGAGTGA
>PGSC0003DMT400038144
ATGGAAGCAATAAGAATAAGCTGTGCTGGATATCCTACTCGGAAACCCTTTTATGAATTTTTAGATCGCTTTG
GCATCCTTTCGCCTGAAGTTTTAGATGGAAGTACGGACGAGGTCACTGCATGCACACGCCTTTTAGAGAAA
GTCGGCCTTCAAGGATATCAGATTGGTAAAACAAAAGTGTTTCTAAGGGCTGGGCAGATGGCAGAGCTAGA
CAGTCGCAGGACAGAGGTGTTAGGGAGATCGGCGAGCATCATTCAGAGGAAAGTTCGTTCTCACATGGCTC
GAAGAAATTTTACATTATTACGTCAGTTGGCAAGAAGGATACAATCGATGTGCAGAGGAGAACTTGCTCGGC
GTGTATATGAGAGCTTGCGGAGAGAAGCAGCTTGTCTGAAGATCCAGACAGACATGCGCATGCATCTTGCTA
GGAAGGGTTACAAAGAATTGTGCTCTGCTGCCATTTCAATTCAGACAGGAATGCGTGGGATGGCTGCACGC
AATGAAGTACGATTCAGGAGGCAGACAAAAGCTGCCATTATCATTCAGAGTCATTCTCGTGCATTCTTGGCC
CGGTTGAAATACAAGAAACTCAAGAAAGCTGCTATTACCACTCAATGTGCTTGGAGAGCTAGAGTCGCTCGT
GGGGAACTACGGAAACTTAAGATGGCTGCAAGGGAGACTGGTGCGCTTCAAGCTGCAAAGAATAAATTAG
AGAAGCAAGTTGAAGAATTAACTTGGAGACTACAACTGGAGAAACGCATGAGGGCTGACATGGAAGAAGC
AAAAACACAAGAAAATGCAAAACTGCAATCAGCTTTGCAAGAAGTTCAACTACAGTTCAAGGAAACACAA
GAAATGTTTGTCAAAGAACGTGAAACTACAAAAAGGGCGGCAGAGGAAGTTCCTATTATGCAGGAAGTTCC
TGTTGTTGATCATGAAATGATGAACAAACTTAGTGTTGAAAATGAGAAACTAAAGTCTTTGGTAAGCTCTCTA
GAACAGAAGATTGATGAGACAGAAAAAAAGTACGAAGAGACAAGCAAACTTAGTGAGGAGAGGTTGAGG
CAGGTGTTGGATGCAGAGTCTATAATTGTTCAGTTGAAGACTACTATGCAAAGGTTCCAGGAAAGGAATTTT
GATTTGGAATCTGAGAACCAGATTCTTCAGCAATCTCTGTTAGCCCCTGCCAAGCAGGTTTCAGATCATTCAC
CTAGTCTGTCTTCCAAGGTGCAGATAGAAGAAAATGGCTACCACCTCAAAGAAGAAACTAGAACCAATGATC
CACCAGGTTCAACACCTGCTAAAAAAGTTGAAACCCCTAATAGCAAGTCAAGGAAACCTCCTATTGACCGAC
AACGTGAAGACATTGGTGCACTCATTGACTGTGTGATGAAGGATGTTGGATTCAGTCAAAGCAAGCCTGTT
GCAGCTTTTACCATCTACAAATGTCTTCTTCACTGGAAATCTTTTGAAGCAGAAAAGACTAGCGTGTTTGATC
GTTTAGTGCAGATGATTGGTTCTGCCATTGAGAATCAAGATAGTGATGACCACATGGCATACTGGCTGTCAA

ATACCTCAACATTGTTATTGTTAATCCAAAAAAGCCTGAAGCCAGACAGTGCAGTTGGTGCGACTCCTACCC
GTAAACcACAGCCTGCAACATCCCTGTTTGGGAGAATGACATTGGGATTTCGCTCGTCCTCTTCTGACATCAA
TCTTGCTGGAGTAGTGCACCAGGTTCAAGCAAAATACCCAGCTCTGCTTTTCAAACAGCAGCTTACAGCATA
TGTTGAGAAAATGTATGGAATTATTCGTGATAACTTGAAGAAGGAGTTAGGGTCACTTCTTTCCTTATGTATC
CAGGCACCAAGGACCTCCAAAGGAAGTGTGCTAAAGTCTGGTCGATCCTTTGGCAAAGATTATTCAATAAAT
CACTGGCGAGGGATAATTGAATGCCTTGATTCTCTTCTCTGTACTTTGAAAGAAAACTTTATGCCTCCGATTCT
TGTTCAGAAGATATTTAGTCAAGCCTTTTCATACATGAATGTACAGCTCTTTAACAGGTGA
>PGSC0003DMT400056647
ATGGCTGCTTCCATTAGCTTACCGGTTGGTTCTCTTGTTTGGGTGGAGGATCCTGATGTAGCCTGGATAGATG
GGGAAGTTTTGGAGGTTAATGGTTCAGACATAAAGGTTCTTTGCACTTCTGGTAAAACGGTTGCTGTTAAGT
CTTCTAATGTCTACGCCAAAGATGCTGAAGCTCCGCCGTCTGGTGTGGATGATATGACGAAGCTGGCTTATTT
GCATGAACCAGGAGTCCTACATAATTTAAAGGCTAGATATGATATCAATGAAATATATACATATACAGGGAACA
TATTAATCGCTGTCAATCCTTTTAGAAGGCTACCACACCTATATGATACCCATATGATGGCCCAATATAAAGGTG
CAGCTTTTGGGGAGCTGAGTCCACACCCTTATGCTGTTGCAGATGCAGCATACAGACTTATGATCAATGATG
GAGTAAGTCAGTCAATATTGGTTAGTGGGGAGAGTGGGGCTGGTAAAACAGAAAGCACCAAGCAACTCAT
GCGCTATCTTGCTTACATGGGAGGGAGAGCTGCAGCTGAAGGTAGTAGATCAGTTGAGCAGCAAGTCCTGG
AGTCTAATCCTGTTCTGGAGGCATTTGGTAACGCAAAAACTGTCAGAAACAATAACTCAAGTCGTTTTGGTA
AGTTTGTGGAGATTCAGTTTGACCAAAAGGGAAGGATTTCAGGAGCTGCTGTCAGAACATATTTACTCGAA
AGATCGCGTGTTTGCCAGTTGTCTGATCCTGAGAGAAATTATCATTGTTTCTACATGCTTTGTGCTGCACCACC
GGAGGACATTCAAAGGTTCAAATTGGACAATCCTAGGACATTTCACTATCTCAATCAGACGAATTGCTATGA
GTTAGATGGGCTTGATGATGCCAAAGAATACTTAGCTACAAGAAGGGCAATGGATGTTGTTGGAATAAGTTC
TGAGGAGCAGGATGCAATATTTCGAGTAGTGGCTGCAATTCTCCATCTTGGAAACATAGAATTTGCAAAGGG
GAAGGAGATAGACTCCTCAGTGCCCAAAGATGAGAAGTCTTGGTTTCATCTGAGAACTGCTGCAGAGCTAT
TCATGTGTGACGTAAAGTCTCTAGAGGATTCCCTTTGCAAACGCGTTATTGTTACTCGTGATGAAACCATCAC
CAAATGGCTGGATCCAGAAGCTGCACTTACCAGTAGAGATGCTCTTGCAAAAATTGTGTACTCAAGATTGTT
TGACTGGCTGGTAGATACGATTAATAGTTCAATTGGTCAAGATCCAAATTCTAAATCTTTGATTGGTGTGCTG
GATATCTATGGATTTGAGAGTTTCAAGACTAATAGCTTTGAACAATTCTGTATCAATTTAACAAATGAGAAGCT
TCAGCAGCACTTCAATCAGCATGTTTTCAAAATGGAACAAGAAGAGTATACAAAAGAAGAAATTAACTGGA
GCTACATTGAGTTCATTGATAATCAAGATATTCTTGATCTGGTAGAAAAGAAACCAGGCGGTATTATAGCACT
TCTTGATGAAGCTTGCATGTTTCCTAGATCTACTCATGAAACGTTTGCTCAAAAGCTCTATCAAACTTTCAAAA
ACCATAAACGTTTCTGCAAGCCCAAGTTGGCTCGTTCTGATTTCACTATATGCCATTATGCTGGTGATGTCACG
TATCAAACCGAATTATTTCTGGAGAAAAACAAGGATTATGTTATTGCTGAGCACCAGGCACTCCTGAATGCTT
CAACGTGTTCCTTTGTATCTGGGCTGTTTCCAACATCAAATGAGGAATCCTCAAAACAATCAAAGTTCTCATC
AATTGGCTCAAGGTTTAAGCAACAACTGCAATCTTTGCTTGAAACACTGAATGCAACAGAACCCCACTACAT
TCGATGTGTCAAGCCTAATAATCTGCTAAAGCCATCTATCTTTGAGAATCACAACGTTCTGCAGCAGCTGCGT
TGTGGAGGAGTGATGGAAGCGATTAGAATAAGTATGGCTGGATATCCTACTCGGAGACCATTCTATGAATTTC
TAGATCGTTTTGGCATCCTTTCTCCTGAAGTTTTAGATGGAAGTACGGACGAGGTTGCTGCATGCAAAAGGC
TCTTGGAGAAAGTTGGACTTCAAAGCTACCAGATTGGTAAAACAAAGGTGTTTCTGAGAGCTGGTCAAATG
GCGGAACTGGATGCTCGAAGAACAGAGGTGTTAGGGAGATCTGCTAGCATCATCCAGAGAAAAGTTCGTTC
TTACATGGCTCGGAGAAGTTTTACAGTGTTACGTCGGTCAACAATACAGATACAATCTCTGTGCCGAGGGGA
ACTTGCTCGGCGTGTATATGAGAGCTTGCGGAGGGAAGCAGCTTCTCTTAGAATCCAGACAAATGTGCGCAT
GCATATTGCTAGGAAGGCTTACGAAGAGTTGCGGTCCTCTGCTGTTTCAATTCAGACAGGATTGCGTGGGAT
GGCTGCGCGCAATGAGCTTCGATTCAGAAGCCAGACAAAAGCGGCAATTATTATTCAGAGCCATTGTCGCA
AGTTCTTGGCATATTCAAAATTCAAGAAGCTCAAGAAGGCTGCAATTACCACTCAATGTGCTTGGAGAGCTA

AAGTTGCTCGTAAGGAACTGAAGAAGCTTAAGATGGCTGCACGGGAGACTGGGGCTCTGCAAGCTGCAAA
GAATAAATTAGAGAAGCAAGTTGAAGAATTAACTTGGAGATTGCAGCTGGAGAAACGCATGAGGGCGGAT
GTTGAAGAAGCAAAAACACAAGAAAATGCAAAATTACAATCTGCTTTGCAGGAGATGCAAGTTCAGTTCAA
GGAAACCAAGGAAATGCTTGTCAAAGAACGTGAAAATGCAATAAGGGCCGCCGAGCAGATCCCTATTGTTC
AGGAAGTCCCCGTTATTGACCATGAATTGATGAACAAACTTAGCATTGAAAATGAGAACTTAAAGACTATGG
TAAGCTCTCTAGAAAAGAAGATTGGTGAAACAGAAAAAAAATATGAAGAGACAAACAAACTTAGTGAGGA
GCGGTTGAAGCAAGCGATGGAGGCAGAGTCCAAGATTGTTCAGTTGAAGACTTCTATGCAAAGGCTTGAG
GAGAAAATTGTTGACATGGAATCTGAGAACAAGATTCTTCGACAGCAGGGCCTGTTAACCCCTGCCAAGCG
GGTTTCAGATCATTCACCTAGTCTGGCCTCTAAGATTGTTGAAAATGGACACCATCTTGATGATGAAAACTAT
ACTACTGATGCACTAAGTTCCTCCACACCTTCTAAAAATTTTGAAACCCCCGACAGCAAGATGAGGAGACCT
CCTGTTGATCGACAGCAGCACGAGGATGTTGATGCACTCATTGACTGTGTGATGAAGGATGTGGGGTTCAG
CCAAGGAAAGCCTGTTGCAGCTTTTACCATCTACAAATGCCTTCTCCATTGGAAATCTTTTGAAGCTGAGAG
GACCAGTGTGTTCGATCGTTTGATTCAGATGATTGGTTCAGCTATTGAGAATCAAGAAAGCAACGATCACAT
GGCGTATTGGCTGTCGAATACCTCAACGTTGTTGTTCTTAATCCAAAAAAGTCTGAAACCAGGTGGTTCAGT
TGGTGCAACTCCTACCCGCAAACCACAACCTCCGACATCTCTATTTGGGAGAATGACAATGGGATTTCGTTC
GTCGCCTTCTGCTGTCAACCTTGCTGCAGCTGCAGCTGCATTGGTAGTGCGTCAAGTTGAAGCAAAATACCC
TGCTCTGCTTTTCAAGCAGCAGCTTACAGCATATGTTGAAAAGATTTATGGAATTATTAGGGATAACTTGAAG
AAGGAGTTGGGATCACTCATTTCCTTATGCATCCAGGCACCAAGGACTGCCAAAGGAAGTCTGAGAACTGG
ACGATCCTTTGGCAAAGACACTTCGACAAATCATTGGCAGCGGATTATTGAAGGCCTCAACTCTCTTCTCTGT
ACATTGAAAGAAAATTTTGTGCCTCCAATTCTTGTTCAAAAGATATTTACTCAGACATTCTCTTACATTAATGTA
CAACTGTTTAACAGTCTTCTTCTTCGAAGAGAGTGTTGTACATTCAGTAATGGGGAATATGTTAAAGCTGGGT
TAGCTGAGTTAGAGCTATGGTGCTGCCAAGCAAAAGAAGAGTATGCAGGTTCCTCTTGGGATGAACTCAAA
CATATTAGACAAGCTGTTGGGTTCTTGGTTATTCATCAGAAGTATAGAATATCTTATGATGAGATCACCAATGA
CTTGTGTCCTATTCTTAGTGTCCAGCAACTTTACAGAATCTGTACTCTCTATTGGGATGACAACTATAATACACG
AAGTGTTTCCCCAGATGTCATATCAAGCATGAGAGTGTTAATGACAGAGGACTCAAACAATGCTGAGAGCAA
CTCTTTCTTACTGGATGATAATTCAAGCATCCCGTTCTCTATTGATGAAGTCTCAGAATCACTTCAAGTAAAGG
ATTTTGCAGATGTCAAACCTGCAACAGAACTTATCGAGCATCCAGCCTTCCCATTTTTACACGAGTGA
>PGSC0003DMT400023467
ATGGGCAAGAAAGGAAGTGGTTGGTTTTCATCTGTGAAGAAAGTTTTTAAACAGTCTCCTAAAGATTCACC
AGACAAGAAGAAGGAAACATTAGACAGCAAATGGCAGCAACCTGAACCTCCTGAAGTTGTGTCACTTGAA
CATTTTCCAGCAGGGAGTTCTCCAGATCTTATTAGTAATGGAGGGAGCAACGTTGATTCATCGTCTACTGTAG
CTGAGGATCATAACCATGCCATTGCTGTTGCAGCTGCCACTGCTGCAGCCGCTGAGGCAGCTGTTGCAGCTG
CTCACGCGGCTGCCAAAGTCGTCAGATTAGCTGGTGGCTATGGCCTCCTGCAGTCTACAGAGGAAAGGGCT
GCCACGCTTATTCAGTCATACTACAGGGGATATCTGGCAAGACGAGCTTTGCGTGCTCTGAAAGGATTGGTA
AGGCTGCAAGCACTAGTGAGAGGACATAATGTGCGAAAACAAGCACAAATGACCATGAGATGTATGCAAGC
ATTAGTACGTGTTCAGGGCAGAGTACGAGCAAGGAGGCTCCAGTTAGTCCAGGAGAGGCTCGAGAGCAAG
CTAGAAGAAGCACGAAGACACGCTGCTGAAGAACAACAACACAAGCACACCAGCCCCATCAAGAAGTCAG
GGGCAGAAGGTTGGGATAACAGGAACCAAAGCATGGACAAAATCCAAGAAAGCAGTAGGAGAAAATTCG
ATGCTGAAATGAAAAGGGAAAGAGCTCTTGCTTATGCCTTTGCCTATCAGCAAAAGCAACAACAACTATTGC
ACATTGATCCACATGGTGATGACGCTGAATTTTTTGGCAATGAGCGCGAAAAGTCACAATGGGGTTGGAGT
TGGCTGGAGCGATGGATGGCTTCACAACCATACCAATACTCAAGACACATTGTGCCAAGAGACAGCTCCTAT
GTGACACCTTCCACAACAAGTGACATGTCTGAAAAGACAGTTGAAATGGACCTAATCACCGGGCCATTGGA
CTCGGAGACCATCAACTCAGCCCGTCACACTTTTGATCAAGCTGAAACAAGCCCATACTCAGCCCGGAAGG
TTGGTCACTCAAAATTTGATAATGTCCCTAGCTACATGACCCCAACTAAGTCTGCAAAAGCCAAGGTGAGAA

ATCAAGGCCCTGTTAAGCATCGTAGCCCACCATCGGCCCAATGGAATGCATCGAAAAGGAAGGGAGCACAC
TATGGTTTGGGCCTTGAATCAAGTTCAGGGGCAAGTACAACAGCAATCTATCAAGTCTCAAGAAGCCCAAG
CCCAAAAACTACCGCGAATCGCAGTAGTGGACATGCTAATAAATGGATGGCAACTTACAGTCCGGAATCAAG
TGGCGATGATCGGGCTTCACCATTAGGACCACATCCCTGGAGATATAATTTTAGTTGA
>PGSC0003DMT400006376
ATGGGTGGTTCGGGGAAGTGGATTAAATCTTTAATCGGTTTAAAAAAGAATCAATCAAATGATTCTGAGAAG
GGTAGTGGAAAGAATAGAAAATGGAAGCTGTGGAGGAGTGCATCGGGTGGAATTGCTATGGCGTTTTCGA
AAGGTGTAAAAGGGGGTGGGAATTTAGGAGATTCGGATGAATCTGAATCTTCATTCCTGTCTGACAGTGCTT
TGGCTGCTGCTATGGCTACTGTGATCAGAGCTCCGCACAAGGATTTTGTGGTTGTGAAACAAGAATGGGCT
GCTCTTCGAATTCAGGCTGCGTTTCGTGGTTTTCTGGCAAGGCGGGCGTTAAGGGCACTCAAAGCAGTGGT
TAGGCTACAAGCTATATTTCGTGGGCGACAGGTGAGAAAGCAAGCTGATGTAACCCTCAAGTGTATGCAGAC
TCTTGTCAAGCTGCAGTCTCGAGTTAGAGCCCGATGCCATCAAACATCCGTTGATGCTACACAAGGGTCTCTT
GTTGATAGCCAAGCTGATCCAATTAAGCAAGCTGAGGGTGGATGGTGTGATAGCCCTGGCACAGTGGATGA
AGTGAGGTGTAAGTTAAAAATGAGACAAGTTGGAGCAATTAAGAGGGAGAGGGCCATTGCATATGCCCAA
CAGAAACTGAGAACAAACCCCAGCCCGAATTCAAGAACAAGGAAAGTTGAAACACCGAATAAGTTCAAGG
CTAATGGGGATTCAGTTTGGTTAGAACGTTGGATGGCGAGTAAGCCTTGGGAAAACAGACTGGTGGAAGA
TTTCCATACTGATGCATCAGGGATGACTCCGAGTTCTAGGAAGTATGAAGATTATGATGCTGGATCTTTCACT
GACCGTAGTTCAGTGAACATTAGGCGGAATAACATGTCTACCAGGATATCTACAAGAGGACCAATGAGTTGT
CAAATTGCTAATTCATCTTCTGAGCCTTATACTGATTATTATCAATATGATGATAGTACGACTTCTCATTCTTCCA
TATCGACTTCTGAAACTCTTGGATCGAATCAGACACCTCCGGAAGAGGGTCATAGCAAGAAACCAAACTACA
TGAATCTCACAAAGTCTATTAAGGCGAAGGTTAAGCAGAGGAATTCAAATTACTTGTCTCACAGTATGCAGA
GGAATTCAACTGATAACTTACATGTCCATAGGAAGTCAAGTCCACTTTCGAGGACGATAGCAAGGAGAAGT
GCTGATTGTGATCTTTATTCAGTTGATCTGTGTAAGGATCTCTACCCTCCCTCAAATGCATTTTGA
>PGSC0003DMT400031576
ATGGGAAAGAAAAAGGGAGGTACTTCATGGTTGAGTGCTGTTAAGAGGGCTTTCAGGTCACCAACTAAAG
ATAACAGCTGCGACAAAAAGGCAAAAATAGAGCATGAACTAGACGAAGATGAAGAAAAGAAAAGAGAGA
AGAGAAGGTGGTTATTCAGAAAACAGTCACAAAATGAAGGGAAAGTAATTGTTGATCCAAAGCATGCAACA
GCAGCAGCAGCTGTGGCCACTGCTCAAGCAGCAGTTGAGATCATAAGGTTGACACGATCTTCCAATAATCCG
TCTTCTAACAGGCAACACAATGCTGCTGTACTCATCCAAACAGCATTCAGAGGCTATCTGGCAAGGAGGGCA
CTAATTGCATTGAAGGGGATAGTGAAGCTTCAAGCATTAATAAGGGGTCAAAATGTACGAAAGCAAGCTAA
GATGACACTGAAATGTATGCAAGCTCTGCTGAGGGTGCAGGCTCGGGTTCGTGAACAACGCGCTCGCCTTT
CACATGATGGAGGCCGCAGGTCCATGTTTGCTGAAACAACCAATTTATGGGACTCTAAATATCTCCGTGACAT
CCGAGATAGGAAGTCCAGATCTAGAGATGGAAGCTCAATTGCAGACGATTGTCCGAGATCACTTCTAGAGCT
GGAATCAATGTTACAAGCCAGAAAAGAAGCCTCCTTCAAACGGGAAAAATCCCTTGCTCATGCTTTTACTCA
ACAGGAATTGGATGAGATGGATGTTTGTAGTGAAGAAAGAAATGAAAGGGAACTAGAAGAGACAGCGAAT
TGGCTAGACGAGTGGATGTCATCAAAGCAATGGAACACCAGCAACAGAGGTTCATTTGACAGAAGAGACTC
TATAAAGACTGTTGAGATGGACACGGCTAAGCCATATTCTAACATGGTTCCAAATGCTCGAAGATCACAACAC
TCGAGCCCACTTCACAGACAGGCTAGTAGTCCTCATTATATTGCTAATTCTCCCCATCACCAGAGATCATCACA
TTACAATTACTCGGCAATTCAACCACCAGCCACCCCGCCCCCTTGTCAACCAAAACCTCTTCAAATGCGCCCA
ACAAGCCCACGTAAAAGCCAATCAACTGCAAACACTCCATGCCTACGCTCTACGAGCCGTTCAAACAGTATA
ATGTCCCGGTATAGCACGTCAGGAAACGATGCATCAGTTCCTAACTATATGGCTGCCACTGAGTCTGCAAAAG
CTCGGATTCGTTCACAAAGCACACCTAAACAAAGGCCTTCCACACCAGAAAGAGAAAGAGTTGGATCGGTG
AAAAAACGCCTCTCTTACCCTATTCCTGAGCCATACTCGCTAAACGCTGCGTATGGCTACAGTCAGAACTTGA
GAAGTCCTAGCTTCAAAAGTCTTCAAGCTGCCTATGTTGGGATGGAACAACAATCATGTTACACCGACAGCC

TTGGTGGAGAAATTTCTCCTTGTTCAACCACAGATTTAAGGAGATGGTTAAGATGA
>PGSC0003DMT400031575
ATGGGAAAGAAAAAGGGAGGTACTTCATGGTTGAGTGCTGTTAAGAGGGCTTTCAGGTCACCAACTAAAG
ATAACAGCTGCGACAAAAAGGCAAAAATAGAGCATGAACTAGACGAAGATGAAGAAAAGAAAAGAGAGA
AGAGAAGGTGGTTATTCAGAAAACAGTCACAAAATGAAGGGAAAGTAATTGTTGATCCAAAGCATGCAACA
GCAGCAGCAGCTGTGGCCACTGCTCAAGCAGCAGTTGAGATCATAAGGTTGACACGATCTTCCAATAATCCG
TCTTCTAACAGGCAACACAATGCTGCTGTACTCATCCAAACAGCATTCAGAGGCTATCTGGCAAGGAGGGCA
CTAATTGCATTGAAGGGGATAGTGAAGCTTCAAGCATTAATAAGGGGTCAAAATGTACGAAAGCAAGCTAA
GATGACACTGAAATGTATGCAAGCTCTGCTGAGGGTGCAGGCTCGGGTTCGTGAACAACGCGCTCGCCTTT
CACATGATGGAGGCCGCAGGTCCATGTTTGCTGAAACAACCAATTTATGGGACTCTAAATATCTCCGTGACAT
CCGAGATAGGAAGTCCAGAGTGAGTTCTACTTTCCGCGCCTTAGTTACTGTGTGA
>PGSC0003DMT400014796
ATGGGCAAAGCATCCAAATGGTTCAAAGCACTTCTTGGCTTCAAGAAAAATGACTCTTCTTCTTCCTCTACTT
CTTCCACTAATAAGAAGAAGTGGAGTGATGTTAAATCCTACAAAGATAAAGACTTTCAACACCACCACCAAC
ACCATGACAAATCACACTACGTGAATAGTCGTGCTGGAGTGGACCCCACAATCTGTGAGGTCCACTCCAGCT
TGACTAGTAGTGTGATCCGTACAACAACGTCCGTGACGCCGTGGAGTGGTGAGGAGTGGGCTGCCGTTGTT
ATACAATCACATTTTCGAGCTTATTTGTCAAGGAGAGCTTTACGCGCATTGAAGGGACTTGTGAAGCTTCAA
GCCCTAGTAAGAGGACACATTGTAAGGAAACAAACCGCGGATATGCTAAGGCGTATGCAAGCGTTGATAAG
GGCTCAATCGAGAGCTCGTTTAGGACGATCTATGGTTTTTGAATCACCTCCTTTTAGCGCGAAGTCTACTCAA
TTTATTCCTCATGGTCCTACAACTCCTGATAAATTCGAGCAAATTATTCGTGCAAGGAGCATGAAAAATGATCA
AATGTTTATGCTCAAGAGGAATTCTTCAAACCATATAGGCAATGCCAATGCCAAACAGAGGAATTTATTTCAT
TCTTCAGAATTTAGCTTAAATTCAGAGCAATTCTGTTTTAGCTATGATGAAACATGTTACTCCTCTATTGATAAT
AGCCCACAACTTCACTCAACAGCATCATCGAAATGTACTCGTTCGAGGACAGGACCATTTACGCCAACAAAG
AGTAGTACACGAAGCTACACGAGTGATGAATACTCGAATAATCATCCAAATTACATGTCTTACACCGAGGCAG
CTAAGGCAAAAACGCGATCTATGAGTGCACCAAGACTAAGGTCTCAGTATGATAAGAGGTACTCAAGAAGTA
ACATGCAAAAGGGTTCTAATTGTTATGCCAATTTCACTAGCAAAGGTGTTTGTTCGGGTTCTGATAGACTAGA
TAAGATTGGAGTGCCTATTAGTGGCGATCTGGATGAATTTGGACGTGGTTTTTGTCACATATATTGA
>PGSC0003DMT400041427
ATGGGGAAAAAAGGCAGTTGGTTTTCTGCCATCAAAAGGGTGTTTACACCTAGCTCAAAGGAGAAATTACC
TAATGAATCAGAGAAGAAAGGCGCTAAGGAAAAGAAGAGTCGCGGAAAACTGAAGCATGGAGAGACTAA
ATCATTTATCCCTCTCTTTAGAGAGCCAAGTAGTATTGAGAAAATACTTGGAGAAGTAGATGAACAAATGCTA
CTTTCTCCAAGATTCACTTTACCCGCGGAAGTTGTCTCTCCTCGGATCTCTTCTTATAGGTTTGCCACTCCTAG
CGCTACGTCTCCAAGAGTTGCCTCTCCTAAGGCTTCCTCTCGCCGGATTACTTCCCCTAAGGCTCCTTCACAA
AGGGTTACTTCTCCAAGAGCTATATCTCCTAAGGCTCATCCTCCAAGGGTACCTTCTCCTAATGTTAGTCGTAA
CCGCAAAGAAATCAGCTATGCTTATAGACCAGAACCAACTTTGAGAACTCTCAATCTTTCAGCAACAAAGAT
ACAGGCAGCCTATAGAGGTTACATGGCAAGGAGGAGTTTCAGAGCTTTGAGGGGTTTAGTAAGGCTTCAA
GGAGTTGTGAGAAGTAGTAATGTAAAAAAGCAAACAGCAAATGCCATGAAACAGATGCAACTTCTCGTTAG
AGTACAAACTCAAATTCAGTCGAGGAGGATCCAAATGTTGGAAAACCAAGCACTTCAACACCAAGCATATA
GAAACGACAAGGAAGTCGAGAGCACCATCAGTAAATGGACTCAACTGTGTGAGGCAGGTAACAATGATAAT
TGGGATGATAGTTTGCTGACTAAAGAAGAAGTAGAAGGAAGGCTGAGGAAAAAAGTGGAGGCAGTCATCA
AGAGGGAGAGAGCAATGGCATATGCATATTCTCACCGGCTATGGAAAAATGATCCAAAATCGGGTTTGGAC
ATGGGAGCTAATGGTTTTCCATGGTGGTGGAATTGGTTAGAACGTCAACTACCTTCAAGAAATGCTAACAAA
ACTCCATCTGCTGTGAAAGATATCAAACTAACACCACCAAGGGCTATTTCAGAGCACAAACCAAGTCCAACG
CCTCTAAACAACGTTACTTTCAGACGTATACTCTCTGATTACGATAACAATGAATCATCAGTCACACCAATGTC

AACCAAGTCAGCAATTCCAACGAGGGGAAAACAGATGCATACTCCAATTAGAACACCACCAATGAACAACT
CAAGTCTAAAGAAGCACTCGAGAGCTCGAGCTAGTGCTTCTAACTACCCTTTTGATCTTCCATTAAAAGACG
ATGATAGCCTCACGAGTTGTCCTCCGTTTTCAGTACCACATTACATGTCACAAACAGCATCAGCTAAAGCCAG
AGCAAATAGCAATCCTAAGGAGAGAAATCCAGAAAAACAATCCAATGACACAAAGAAAAGATTTTCATTTCC
TTTAACTCCAAATATATGGTCATCCAAATGGAGTAAAGGCTCTGGAAAGGATCCAACTTCTCGAAAAGAAGT
CGATAAACACGAGTCCATGGCTGATCATATAAGTGTGGATTCAACTGTTTCGATGCCCGCGGTTGTTGGTGG
GAGGAGACCATTTAACAGATTTGTGTGA
>PGSC0003DMT400033128
ATGGGAAAGAGAAGAAGCTGGTTTATATTTGTGAAAAGACTACTTTTCATTCCTGAGGCAAAACCAAAAGC
TGAAAAGAAACCAAAGAGATGGAAATGGTTTTTAGGAAGGTTCAAGTTCAAGCAGTGTCCTCCAGCAATTG
AAGCACCTCAAAAAACACTAACTGAGGCAACAGAACAACAGAAGAAACTTGCTGTGGCTGTTGCTTTAGCA
ACAGCAGCTGCAGCTGAGGCTGCTGTAGCTGCTGCCAATGCTGCAGCCGAAGTTGTACGCCTCACTATCGCT
CCGTATGAGCTAGAGAGGAAGCGGAGAAATGCTGCCATCAGAATCCAAACCGCTTATCGTGCACACCTTGC
AAGGAAAGCACTAAGTGCCCTGAAGGGACTCGTGAAGCTTCAAGCAGTGATTCGAGGAGAACTTGTTAGA
CGAAGATTAGTTGCCAAATTGAAGTTTATGTTGCCCTTTCAAATGCCAAAGCCAAGAGTTTATCATATCAGAG
TTCCTACTGTGGAAGAATACTACGAGAGTATCGAAAAGAAACTTGATGATAGCTCGAAGGAAAGTGTGAAAT
CTAATGAATTCAAACTAAAATGCAACAGCCAAAGGACTTGGGATTTCAGTTTGGCTTCAAAGGAAGAAATT
GACTCCTTGTTTTTAAGAAGACGAGAAGCCTTTGCCAAAAGAGAGCGTATGATGAAATACTCGTTTTCACAT
CGGGAGCGGAGAAATGATCATGTTATGCAAGATCCGTTAATCATTAAGGAAAATCGAAGAAGTTCCAGGTTC
GATCAATGGGAAGAAATGGAAGCACAGAGGAAAGCAGAGCTATTTGAGCAATTGAGGTCATTTGCAAACT
CAAGTAGTCCTCTAGTTGGCATGAACCAAATGAGACAAACGCGTAAACTAGATGTCACAGAGGATTTTAATT
CTCCGTCATCGCTACCAAGGAGATCATTTTCTCATGTGAAACAGAAATCAATTGGTGATGATAGCTCTTTACC
AAGTTCTCCCATGTTTCCTACTTACATGGCTGCTACAGAATCTGCAAAAGCAAAGACGAGATCGATGAGCAC
ACCAAAGCAACGACTAATGTTAAACGAAACATATTCAGTTCAACATTCTCCCTTCATGCTCAACCATACTTCTT
GGACTTCATATAATGGTGAAGTGAACAAAAGTACCAAAAAGAGTGAAATCTCTCAGCAAACATTTCTATAA
>PGSC0003DMT400009545
ATGGGAAAATCTACAGCTTCTTCTTGCTTAAAGATCATTGCTTGTGGCAGTGATTCTGTTGACCGTGATGAAC
TTGAAGCTCATCCTGAGAGTAAAAGCTCAAGCGACAAACGTGGATGGAGTTTCCGCAAGAAATCTGCCAGG
CATCGTGTATTAAGCAATACAGTAGTTTCAGAAACACCATCTGGGAACAAGGACTGGCCAGAAGCTGCTAAT
GCCAATCTGCAAACACAATCTAACTCCACCATTCCAGAGAAGGCATCTGTTGTCCAATGGGCAGATGAGAAA
CCCCAGTTTCCAACGGTCGAGAAGTCTCAGGTGTCGGCTGACGAGAAGCCCCAGGTCTTGGCAAATGAGA
ACCCCCAGATCTCAGAGGATGAGAAGCCACAGGTTCTGGAAGATGAGAAGCTCCAGGTCTCAGTGGATGA
GAAGCCCCAAGTCTCCACGGACGCGAAGCCACAGCTCTTGGTGGAAGTCTCAGTGGATGAAAAGCCCTTG
ATCTCCGAAAAAGTGAATCTCGAGGTCTCCGAAGATGAGAAGCCCAGCGTCTCTTCAGATGAAAAGGCCCC
CATCTCATCAGAAGAGAACTCCCTGCTCTCAGACTTGGTGGATGCAAAACAATCAGAGCCAGTAACAGCTAG
AGTCAATGATGGTAAAGCTGATGTCATCCTGGATGAACACGCTCTTGTTATCCAGACTGCAGTCAGAGCATTT
CTGGCACGAAGAGCTCAATTGAAGCAAAAGCATATAACTAAATTGCAAGCTGCTGTACGTGGACATTTAGTT
CGCAGGCATGCTGTAGGAACTCTGCGATGTGTTCAAGCTATTGTCAAAATGCAAACTCTTGTTCGAGCACAT
CACACTAATCGCATTGCAGAAGGATCTAGTATCAAGGAAAAGCTAAAAGGAAAAGAGAACTCAGGAACAA
AATCAGAGTTCACATACATTTCTATTTCAAAGCTACTGAGCAATAGCTTCGCTCGACAGCTCCTTGAATCAACT
CCAAGGACCAAAAGTATAAATATTAAGTGTGACCCTTCCAAATCTGATTCTGCCTGGAAATGGTTAGAGAGAT
GGATGTCTGTTGCATCACCAGGAAATCAACTGTCACCACAGTCAGAATTATCTGCCGAGCAGCAGGAGAATG
AACCTACCGAGCACCACAGTAACCTTATGGAAAGTAAAGTTCAGCTTGATTCCGAGTCAATGGACTTCAGAG
AGGGTGAGGAGGCATCGCTGTCTGCAGTGCCATCTGAAAGTGATGATAATTTGATCACTTATGATGCAGACA

GCTTAGATTTTCAAGCCGACATACCGACTTTACCTCCTCAGCCTCTGAATGTTGATGAAAAAACTTCAAGAGA
TGACTGTTCTATTCCTACTCAACTTAAGGAGGCCAGGGCTCTTCCTGAGATGGAGCCCAATTCTTTCCCTGCA
AACACTGAAGTTGAGAGAGAGGATACACATTCCCTTGAACTTTCAGAGACCGAGAGCAAAAAGATTTTACA
TGGATCAAGAAAGGCAAGTAATCCTGCATTTATTGCTGCTCAGTCAAAGTTTGAGGAACTCACTTTGGCAGC
TAAATCAACCAAAGTGACTAGTTTGCCCAATCATAAAACTGAAGATGAATCTAGTGAAGATACGTTTTCAACT
ATCACTGATCATTCATTTGGGGCAAGGGAAGCTGCTCCGTCAGAAAATTCTGTTCCTCATAGTACAAGAGCT
CAAGTTGGTGGTTCAGAATGTGGCACGGAGCTTTCTATTTCTTCTACCCTGGATTCACCAGATAGGTCTGAC
GTCGGAGGTCATGTATTTGAGCAGGAACTTCCTTCCAATGGTGGAACTGACCATCGTAAGAGCAATGGATAC
CCTCACATTGAAGATGATAGTACAAATGACTTATCACACTCCGACTATGTTCAGGCAGGGAGAGAGGATCCT
ACTGATGATGCTAAGCATGTAGATGTTATGGTCAGTTCAGATCTATCACCCGAAGAACAGAAGCCAGAAAAC
AATTCAGTTAATGTTCAAATAGAGCACGAAGCTAAGACGGATCGACTATACAAGTCATCACCAGACGCATCTC
CAAGGAGCCATATAACCGTCCCTGAATCCCAAGGGACACCTTCTAGTCAAGTGTCAGTGAATCCTAAGAAGC
TAAAAAGTGAAAACAGTGGATCAATTCCCAAGCCCCGTTCTGCACCTGCTAGCAAAAAGTCTCCTTCAAAGC
TAAACCATGCTCCAGGCACAACTAGTTCGGAACAATTATCTAAGGATCATAATAAAAATGAGAAGCGACGAA
ACTCGTTTGGTTCAACAAAAGCTGGACAAGCTGATCAGGAGGCTAGAGATAACAGTACTAGCAGTTCTCTCC
CAAGTTACATGCAAGCAACAGAATCTGCAAGAGCCAAAGTTATCCCAAATAGCTCCCCAAGATCTAGTCCAG
ATGTCCACAATAAAGATGAATATATCAAAAAGAGACACTCTCTCCCTGGTTCAAATGGTAGGCAAGGTTCACC
TCGTATCCAGCGGTCTCTGTCTAATGCACAGCAGGGTGCAAAGGGAAATGGAACTCAATCTCCACAGGAGA
GGAAGTGGCAGAGATGA

>PGSC0003DMT400009544

ATGGGAAAATCTACAGCTTCTTCTTGCTTAAAGATCATTGCTTGTGGCAGTGATTCTGTTGACCGTGATGAAC
TTGAAGCTCATCCTGAGAGTAAAAGCTCAAGCGACAAACGTGGATGGAGTTTCCGCAAGAAATCTGCCAGG
CATCGTGTATTAAGCAATACAGTAGTTTCAGAAACACCATCTGGGAACAAGGACTGGCCAGAAGCTGCTAAT
GCCAATCTGCAAACACAATCTAACTCCACCATTCCAGAGAAGGCATCTGTTGTCCAATGGGCAGATGAGAAA
CCCCAGTTTCCAACGGTCGAGAAGTCTCAGGTGTCGGCTGACGAGAAGCCCCAGGTCTTGGCAAATGAGA
ACCCCCAGATCTCAGAGGATGAGAAGCCACAGGTTCTGGAAGATGAGAAGCTCCAGGTCTCAGTGGATGA
GAAGCCCCAAGTCTCCACGGACGCGAAGCCACAGCTCTTGGTGGAAGTCTCAGTGGATGAAAAGCCCTTG
ATCTCCGAAAAAGTGAATCTCGAGGTCTCCGAAGATGAGAAGCCCAGCGTCTCTTCAGATGAAAAGGCCCC
CATCTCATCAGAAGAGAACTCCCTGCTCTCAGACTTGGTGGATGCAAAACAATCAGAGCCAGTAACAGCTAG
AGTCAATGATGGTAAAGCTGATGTCATCCTGGATGAACACGCTCTTGTTATCCAGACTGCAGTCAGAGCATTT
CTGGCACGAAGAGCTCAATTGAAGCAAAAGCATATAACTAAATTGCAAGCTGCTGTACGTGGACATTTAGTT
CGCAGGCATGCTGTAGGAACTCTGCGATGTGTTCAAGCTATTGTCAAAATGCAAACTCTTGTTCGAGCACAT
CACACTAATCGCATTGCAGAAGGATCTAGTATCAAGGAAAAGCTAAAAGGAAAAGAGAACTCAGGAACAA
AATCAGAGTTCACATACATTTCTATTTCAAAGCTACTGAGCAATAGCTTCGCTCGACAGCTCCTTGAATCAACT
CCAAGGACCAAAAGTATAAATATTAAGTGTGACCCTTCCAAATCTGATTCTGCCTGGAAATGGTTAGAGAGAT
GGATGTCTGTTGCATCACCAGGAAATCAACTGTCACCACAGTCAGAATTATCTGCCGAGCAGCAGGAGAATG
AACCTACCGAGCACCACAGTAACCTTATGGAAAGTAAAGTTCAGCTTGATTCCGAGTCAATGGACTTCAGAG
AGGGTGAGGAGGCATCGCTGTCTGCAGTGCCATCTGAAAGTGATGATAATTTGATCACTTATGATGCAGACA
GCTTAGATTTTCAAGCCGACATACCGACTTTACCTCCTCAGCCTCTGAATGTTGATGAAAAAACTTCAAGAGA
TGACTGTTCTATTCCTACTCAACTTAAGGAGGCCAGGGCTCTTCCTGAGATGGAGCCCAATTCTTTCCCTGCA
AACACTGAAGTTGAGAGAGAGGATACACATTCCCTTGAACTTTCAGAGACCGAGAGCAAAAAGATTTTACA
TGGATCAAGAAAGGCAAGTAATCCTGCATTTATTGCTGCTCAGTCAAAGTTTGAGGAACTCACTTTGGCAGC
TAAATCAACCAAAGTGACTAGTTTGCCCAATCATAAAACTGAAGATGAATCTAGTGAAGATACGTTTTCAACT
ATCACTGATCATTCATTTGGGGCAAGGGAAGCTGCTCCGTCAGAAAATTCTGTTCCTCATAGTACAAGAGCT

CAAGTTGGTGGTTCAGAATGTGGCACGGAGCTTTCTATTTCTTCTACCCTGGATTCACCAGATAGGTCTGAC
GTCGGAGGTCATGTATTTGAGCAGGAACTTCCTTCCAATGGTGGAACTGACCATCGTAAGAGCAATGGATAC
CCTCACATTGAAGATGATAGTACAAATGACTTATCACACTCCGACTATGTTCAGGCAGGGAGAGAGGATCCT
ACTGATGATGCTAAGCATGTAGATGTTATGGTCAGTTCAGATCTATCACCCGAAGAACAGAAGCCAGAAAAC
AATTCAGTTAATGTTCAAATAGAGCACGAAGCTAAGACGGATCGACTATACAAGTCATCACCAGACGCATCTC
CAAGGAGCCATATAACCGTCCCTGAATCCCAAGGGACACCTTCTAGTCAAGTGTCAGTGAATCCTAAGAAGC
TAAAAAGTGAAAACAGTGGATCAATTCCCAAGCCCCGTTCTGCACCTGCTAGCAAAAAGTCTCCTTCAAAGC
TAAACCATGCTCCAGGCACAACTAGTTCGGAACAATTATCTAAGGATCATAATAAAAATGAGAAGCGACGAA
ACTCGTTTGGTTCAACAAAAGCTGGACAAGCTGATCAGGAGGCTAGAGATAACAGTACTAGCAGTTCTCTCC
CAAGTTACATGCAAGCAACAGAATCTGCAAGAGCCAAAGTTATCCCAAATAGCTCCCCAAGATCTAGTCCAG
ATGTCCACAATAAAGATGAATATATCAAAAAGAGACACTCTCTCCCTGGTTCAAATGGTAGGCAAGGTTCACC
TCGTATCCAGCGGTCTCTGTCTAATGCACAGCAGGGTGCAAAGGGAAATGGAACTCAATCTCCACAGGGTAT
TTTCTAA
####Protein
>PGSC0003DMP400002357
MGKKSPAKWIKAVLFGKKSSKSHLSKDASGEKISSAKAPVGDLSLDSPSLDLPVQNFDNGGDQAGLEKGTSTDF
ACETASLSSATHDIEPHVNGTISTDDAELKRQEHAATIAQAAFRGYLARRAFRALKGIIRLQALIRGHLVRRQAVST
LRCMQAIVRIQALARGRRIRLLDPGHQLLGKYNFEELKDPEQRPAKLTAYAFPRKLLVAVPTAMPLSLQYDECEPN
SAWQWLERWSLSRFWEPLPQPKKVVGAKSLKKQGNKPSVETEAVRPKRSVKKVLTASNGDAYAVSSSEPEKAK
RNPRKFSNHHIEPVQDQPQNELEKVKRNLRKVSAALATSSERSETEIEKAQQTPNLAQAQAQAIASKSSAPDVV
EQMMVNSYEKTSDSVPEIEKLAESEAPLPVPVDEPSDVLHDHPTTEQQQSEDVNNTANSPVVNEELSSMEDQ
TTKERIRRRKSLPTKQDNSENISQNTPSVPSYMAATQSAKAKLKAQGSPKVSDDGAENGFVRRHSLPSSANGKF
NSLSPRIQKPGQANGKGGNKTRPMPSSKDEKVLPGWRR*
>PGSC0003DMP400002358
MGKKSPAKWIKAVLFGKKSSKSHLSKDASGEKISSAKAPVGDLSLDSPSLDLPVQNFDNGGDQAGLEKGTSTDF
ACETASLSSATHDIEPHVNGTISTDDAELKRQEHAATIAQAAFRGYLARRAFRALKGIIRLQALIRGHLVRRQAVST
LRCMQAIVRIQALARGRRIRLLDPGHQLLGKYNFEELKDPEQRPAKLTAYAFPRKLLVAVPTAMPLSLQYDECEPN
SAWQWLERWSLSRFWEPLPQPKKVVGAKSLKKQGNKPSVETEAVRPKRSVKKVLTASNGDAYAVSSSEPEKAK
RNPRKFSNHHIEPVQDQPQNELEKVKRNLRKVSAALATSSERSETEIEKAQQTPNLAQAQAQAIASKSSAPDVV
EQMMVNSYEKTSDSVPEIEKLAESEAPLPVPVDEPSDVLHDHPTTEQQQSEDVNNTANSPVVNEELSSMEDQ
TTKERIRRRKSLPTKQDNSENISQNTPSVPSYMAATQSAKAKLKAQGSPKVSDDGAENGFVRRHSLPSSANGKF
NSLSPRIQKPGQANGKGGNKTRPMPSSKDGKKK*
>PGSC0003DMP400028833
MHGIPGASCYRNVSKLGYQALELKILLQAVVKLQRWWRCKLLHAQRTKAAVVIQSHALGWIARQRASRNKERL
LQAVLKLQRWWRSKLLHEQRTKAAVVIQSHILGWLARQSASRNKDQLLQAILKLQRWWRGKLLHKQRTKAAV
VIQSHAQGWKARQRASRKKYLTLLAVLKLQRWWRGKLLHKRRTKSAVVIQSYVRGWIACQSVSRNKHRIVVIQ
AYMKGYLARKDLRGQLLDLRHKIQKSAANVDDGMRIINRLVAALSELLNMRSVSDILHICATLNMATQHSQKCC
EELVAAGAVGTLFKLIRSLSRSIPDQEVLKPALSTLRNLSRYPHLIDVLIESYGSLETIVSEFLRNKEEGYFIASDLLKKI
FTEKKGVEAVCKSPALLKRLHNHVEELSRRAKADKRTKPHAMKEPVDKRLREAVEILELIKVSMGNPSKRLSMKV
*
>PGSC0003DMP400038858
MVTTYQMKSLSTLELMLEELQQEDEGTNDLPPPLPVRPVIKARLPKGRRKLTFGKEKSNLEDIRVQENVFVDQ
WSTSAERDSAAMTDKICLMVDQREGAELSRVLQIQRCFRGYQARQYYHELKTGAVALQSFVRGEIERKYYQGL
TRRLAAIIFIQKHIKKHHHKRTERQRTAAICLQSVIRGWLTRKKSNLSGDEKRSCVQNIREKNDLDNKEPETKVPR

SVLLDLQRHILKTEAALERKKGENAALRLHIQHYEIKWNQYESKMKAMEKMWQDQLTSIQISLAAEREKHGDE
KTKGKLRLLILQDQDENVHNGFPRTISLRTSALNHPDEQPSGRSNPKNKCNNHHVMDMVNHYQNFVVHDQC
NSGEEGSALRPNDELQKLKIRFEAWKKDYKNKLREAKATMKQLGHSERGKGSKIWCGR*
>PGSC0003DMP400006554
MRMSKKNWFGNIRKKLFRSSPPHKNIIVLHNNTITNRTSSANGRSSTKKNNGHIYFMSKEDMAAITIQSHFRG
HLARRAFKALKSLVRLQAVVRGAYVRRQARIALHCMHALARLQVTVRARQLLSKCNDH*
>PGSC0003DMP400036363
MASVNIIVGSHVWVEDPKLAWKDGEVIKIHGQDLHVKTSDGKEVVAKIAKVFPKDTETPPGGVDDMTKLSYL
HEPGVLQNLATRYELNEIYTYTGNILIAVNPFQRLPHLYDTHMMEQYKGAAFGELSPHVFAVADVAYRAMINEG
KSNSILVSGESGAGKTETTKMLMRYLAHLGGRSGVEGRTVEQQVLESNPVLEAFGNAKTVRNNNSSRFGKFVEI
QFDKSGRISGAAIRTYLLERSRVCQISNPERNYHCFYLLCAAPAEEVERYKLQNPKSFHYLNQSKYYELDGVNDAE
EYLATRRAMDIVGISEEEQDAIFRVVAAILHLGNVEFAKGEEIDSSVIKDEQSRFHLNMTAELLKCDAKSLEDALIT
RVMITPEEVITRTLDPEAALGSRDALAKTIYSRLFDWIVEKINISIGQDPNSKSIIGVLDIYGFESFKTNSFEQFCINF
TNEKLQQHFNQHVFKMEQEEYEKEEINWSYIEFVDNQDVLDLIEKKPGGIIALLDEACMFPKSTHETFAQKLYQ
TYPKNKRFIKPKLSRTSFTISHYAGEVTYQADLFLDKNKDYVVAEHQVLLTASKCTFVAGLFPPLPEESSKSSKFSSI
GSRFKLQLQSLMETLSSTEPHYIRCVKPNNALKPCIFENLNVIQQLRCGGVLEAIRISCAGYPTRRTFYEFLLRFGV
LAPEVLAGSYDDKVACQMILDKKGLKGYQMGKTKVFLRAGQMAELDARRAEVLGNAAKIIQRQIRTYIMRKEF
VSLRQAAIQLQSCWRAMLSCKLYEQLRREAAALKIQKNFRCHVAHITYTTLHSSAIMLQTGMRAMVARNDFRF
RKHTKAAIKIQAHARGHAAYSYYRSLQRAAIITQCGWRRRVARKELRNLKMAARETGALKEAKDKLEKKVEELT
WRLQFEKRLRAELEETKAQEVTKLQEALHTMQKQVEEANAKVVQEREAARRAIEEAPPVIKETPVIVQDTEKIN
ALSAEVDNLKALLASEKKATEEARDSSRDAEAKNTELASKLETAERKVDQLQDSVQRLEEKLSNMESENQVLRQ
QALTMSPTGKALSARPKTTIIQRTPENGNVINGESKPNSDMSLVVASPKEPSSEEKPQKSLNEKQQENQDMLIK
CISQDLGFSGGKPIAACLIYKCLLHWRSFEVERTSVFDRIIQTIASAIEVPDNNDVLAYWLCNTSTLLMLLQQTLKA
SGAANLTPQRRRSSSASLFGRMSQGLRGSPQSAGLSVLNGRMLGRLDDLRHVEAKYPALLFKQQLTAFLEKIYG
MIRDNLKKEISPLLGLCIQAPRTSRASLIKGRSQANAAAQQALFAHWQSIVKSLNNYLMMMKSNHVPPFLVRK
VFTQIFSFINVQLFNSLLLRRECCSFSNGEFVKAGLAELEQWCCYATEEFVGSAWDELKHIRQAVGFLVIHQKPKK
SLNEITNELCPVLSIQQLYRISTMYWDDKYGTHTVSSDVISSMRVMMTEDSNNAVSSSFLLDDDSSIPFSVDDIS
KTMQQIDIGDVEPPPLIRENSGFVFLHQRSS*
>PGSC0003DMP400036362
MASVNIIVGSHVWVEDPKLAWKDGEVIKIHGQDLHVKTSDGKEVVAKIAKVFPKDTETPPGGVDDMTKLSYL
HEPGVLQNLATRYELNEIYTYTGNILIAVNPFQRLPHLYDTHMMEQYKGAAFGELSPHVFAVADVAYRAMINEG
KSNSILVSGESGAGKTETTKMLMRYLAHLGGRSGVEGRTVEQQVLESNPVLEAFGNAKTVRNNNSSRFGKFVEI
QFDKSGRISGAAIRTYLLERSRVCQISNPERNYHCFYLLCAAPAEEVERYKLQNPKSFHYLNQSKYYELDGVNDAE
EYLATRRAMDIVGISEEEQDAIFRVVAAILHLGNVEFAKGEEIDSSVIKDEQSRFHLNMTAELLKCDAKSLEDALIT
RVMITPEEVITRTLDPEAALGSRDALAKTIYSRLFDWIVEKINISIGQDPNSKSIIGVLDIYGFESFKTNSFEQFCINF
TNEKLQQHFNQHVFKMEQEEYEKEEINWSYIEFVDNQDVLDLIEKKPGGIIALLDEACMFPKSTHETFAQKLYQ
TYPKNKRFIKPKLSRTSFTISHYAGEVTYQADLFLDKNKDYVVAEHQVLLTASKCTFVAGLFPPLPEESSKSSKFSSI
GSRFKLQLQSLMETLSSTEPHYIRCVKPNNALKPCIFENLNVIQQLRCGGVLEAIRISCAGYPTRRTFYEFLLRFGV
LAPEVLAGSYDDKVACQMILDKKGLKGYQMGKTKVFLRAGQMAELDARRAEVLGNAAKIIQRQIRTYIMRKEF
VSLRQAAIQLQSCWRAMLSCKLYEQLRREAAALKIQKNFRCHVAHITYTTLHSSAIMLQTGMRAMVARNDFRF
RKHTKAAIKIQEFISLLLLQCL*
>PGSC0003DMP400033506
MIQEYELDGIDWSKVDFEDNQECLNLFEKKPIGLISLLNEESNSLKATDLTFACKLKQHIKSSPCFKSEREEFCIRHY
AGEVTYDATGFLAKNRDVLHPDIIQLLSSSDSHLPEDKKFSIPSTDTGVLDFKKQSVATKFKDNLFKLMQQLENTI

PHFICCIKPNNKQLPGMSDKDLVIEQLRCCGVLEVVRISRSGYPTRLTHQEFTSRYGFLLPKDNACQDPLSMSVAI
LHQFGILPELYQVGYTKLYFRSGQIASLEDVRNQVLQGTLEVQKCFRRHRARRHFHELKGGVIILQSFIRGEIERRL
YNTKVMSKGKVAREGSDEQLVAVVQIQSAIRGWLARRDLRKLQNSKMLNVDKRRSGRKTEVKELPREILPSVV
EDLERRVAKAEATIEQKEKENAALKEQANQFEARCLEYEVKMRSMEEMWQKQMTSLQANLAAAKNTLAAGD
TTGQPGKLEGSPSPHYYDSDDATSMDTPAGRTPINFSNNSLGVVTNREVNGGLSLISHLTMEFEQRKQNFDNE
AMAIVHLKPGLLHSTNNPADEYRRLKHRFEEWKKEYKVRLKETKSKVHKLVHSKAGKSHRKWWGKKSK*
>PGSC0003DMP400025941
MKKKHIPSYSKQTQGQGTTTTTNDHQLSLVTQDESLLTKCFIGKSIIGGPSWLGLMKKAFRSPIKENDIKSIRRRE
EAHDQEEEEKKRGKRRWIFRKPTVHETTLIHHNQDQENAATTSKGNLATIFLPKNADLKQKGAIGVAITRGGSSL
SAATTSTIATDVAIAKTQATIDITQLTQPPILVKQQCAILVIQTTFRGYLARKALGALKGVVKLQALIRGHNVRKRAQ
ITLQCMQSLVRVQNQVCDQRRRLSCEGISCDSMFKEPKSILELHLNDKESNSINQASIPDNDGYDHLHALEKIEA
LLHKAKVAAKKRENTLAHAFSRKMWTSNKDEDSSSNTELDEDLRVFDVIDEKNRKSTSRASCDQPRDRIKNIEI
DTACSYSNSDTEFWRLHHQYYHDHQQKFYSSYVVPSPLSHRANINALPMTPPLKMKNIQVHSASPRYRREEN
NHHRMALLSHRANVNSSPTAVDQPSYMAATASARARERSQSTPRQIPMTPEREKTSSTKKRLSFPIQ*
>PGSC0003DMP400054220
MGTPVNIIVGSQVWIEDPDDAWIDGEVTEIKGSNATVATTNGKTTVASISSIYPKDTEAPPSGVDDMTKLAYLH
EPGVLNNLACRYSLNEIYTYTGNILIAVNPFRRLPHLYDTHMMQQYKGAPFGELSPHLFAVADACYRALINEHGN
QSILVSGESGAGKTETTKMLMRYLAFMGGRSGTEGRTVEQQVLESNPVLEAFGNAKTVKNNNSSRFGKFVEIQ
FDKHAKISGAAVRTYLLERSRVCQVSDPERNYHCFYMLCAAPPEDVKRFKLGNPKSFHYLNQSSCYEVANVDDA
REYLETRNAMDVVGIGQEEQEAIFRVVAAILHLGNINFVKGKEADSSKLKDEKSLFHLKTAAELFMCDEKALEDS
LCKRVIVTPDGNITKLLDPAAATTSRDALAKTVYSRLFDWLVDKINNSIGQDPEAKSIIGVLDIYGFESFKINSFEQF
CINLTNEKLQQHFNQHVFKMEQDDYTTEEINWSYVEFVDNQDVLDLIEKKPGGIIALLDEACMFPKATHETFAQ
KMYQTYRAHKRFSKPKLARTDFTINHYAGDVTYQADHFLDKNKDYVIAEFQALLMDSKCFFVANLFPPLPEESS
KQSKFSSIGTRFKQQLQSLMETLSTTEPHYIRCVKPNTVLKPGIFENMNVLNQLRCGGVLEAIRISCAGYPTKRTF
DEFLDRFGTLAPDVLDGCDEKSACIAICDRMGLKGYQIGKTKVFLRAGQMAELDARRTEVLAHAAKRIQRQIRT
YLTRKEFIALKRATIHFQKLWRAQLARVLYEQMKREAASIRIQKHARSHSARKSYKELQAAAVVIQTGMRAMAA
RNEYRQRRRNKAAKIVQTQWRGFHAFSTYKQKKKASLSLQCLWRGRLARKVLRKLRMDARDTGALKEAKDKL
EKRVEELTWRLDFEKHLRIDLEEAKGQEISKLQKALQEMQTQLDEAHDAIIHEKEAAKIAIEQAPPVIKEVPVIDN
TKVEKLTEENNKLEEEIRELKKRVEDFEQSYNEVEKECQATRKEAEESQLRVSEFQESIERLQLNLSNLESENQVLR
QQALVASTNEALSDEMDILKNKIKDLESENELLRTQRVVVEQVVSSDDRAPKGLETVDITHPADNEHQTEEVHE
EMKVEQQIPKLLQDSSPPISLTKQRSLTDRQQESHDILIKCLAEDKRFDKGRPVAACTLYKALLQWRSFEAEKTNIF
DRIIHTIRSSIEDQDNTGDLAYWLSTSSTLLFLLQSTIKAGNAPTRSPYRNRSSPTTLFGRMAQQGFRSTSLSMAIS
SGYSGIEGSPNVRTRIEAKYPALLFKQHLTACVEKIYGMIRDNLKKEISPFLNQCIHAPRSARIKPLKGSSRSIHSNII
AKQQASSVHWQNIVNSLDNTLTILSENNVPSTITRKILSQVFSYINVQLFNSLLLRRECCSFSNGEYLKAGLQELES
WCSKATEQYAGSSWDELQHIRQAVGFLVLHQKSQKALDEITNDLCPMLSIAQIYRIGTMFWDDKYGAHGLSPE
VISKMRALTLEDSASIPNNTFLLDVDSSIPFSIEEISRSFQNINLSDVEPPPLLCQRSDFQFLLQAAA*
>PGSC0003DMP400054216
MGTPVNIIVGSQVWIEDPDDAWIDGEVTEIKGSNATVATTNGKTTVASISSIYPKDTEAPPSGVDDMTKLAYLH
EPGVLNNLACRYSLNEIYTYTGNILIAVNPFRRLPHLYDTHMMQQYKGAPFGELSPHLFAVADACYRALINEHGN
QSILVSGESGAGKTETTKMLMRYLAFMGGRSGTEGRTVEQQVLESNPVLEAFGNAKTVKNNNSSRFGKFVEIQ
FDKHAKISGAAVRTYLLERSRVCQVSDPERNYHCFYMLCAAPPEDVKRFKLGNPKSFHYLNQSSCYEVANVDDA
REYLETRNAMDVVGIGQEEQEAIFRVVAAILHLGNINFVKGKEADSSKLKDEKSLFHLKTAAELFMCDEKALEDS
LCKRVIVTPDGNITKLLDPAAATTSRDALAKTVYSRLFDWLVDKINNSIGQDPEAKSIIGVLDIYGFESFKINSFEQF
CINLTNEKLQQHFNQHVFKMEQDDYTTEEINWSYVEFVDNQDVLDLIEKKPGGIIALLDEACMFPKATHETFAQ

KMYQTYRAHKRFSKPKLARTDFTINHYAGDVTYQADHFLDKNKDYVIAEFQALLMDSKCFFVANLFPPLPEESS
KQSKFSSIGTRFKQQLQSLMETLSTTEPHYIRCVKPNTVLKPGIFENMNVLNQLRCGGVLEAIRISCAGYPTKRTF
DEFLDRFGTLAPDVLDGCDEKSACIAICDRMGLKGYQIGKTKVFLRAGQMAELDARRTEVLAHAAKRIQRQIRT
YLTRKEFIALKRATIHFQKLWRAQLARVLYEQMKREAASIRIQKHARSHSARKSYKELQAAAVVIQTGMRAMAA
RNEYRQRRRNKAAKIVQTQWRGFHAFSTYKQKKKASLSLQCLWRGRLARKVLRKLRMDARDTGALKEAKDKL
EKRVEELTWRLDFEKHLRIDLEEAKGQEISKLQKALQEMQTQLDEAHDAIIHEKEAAKIAIEQAPPVIKEVPVIDN
TKVEKLTEENNKLEEEIRELKKRVEDFEQSYNEVEKECQATRKEAEESQLRVSEFQESIERLQLNLSNLESENQVLR
QQALVASTNEALSDEMDILKNKIKDLESENELLRTQRVVVEQVVSSDDRAPKGLETVDITHPADNEHQTEEVHE
EMKVEQQIPKLLQDSSPPISLTKQRSLTDRQQESHDILIKCLAEDKRFDKGRPVAACTLYKALLQWRSFEAEKTNIF
DRIIHTIRSSIEDQDNTGDLAYWLSTSSTLLFLLQSTIKAGNAPTRSPYRNRSSPTTLFGRMAQGFRSTSLSMAISS
GYSGIEGSPNVRTRIEAKYPALLFKQHLTACVEKIYGMIRDNLKKEISPFLNQCIHAPRSARIKPLKGSSRSIHSNIIA
KQQASSVHWQNIVNSLDNTLTILSENNVPSTITRKILSQVFSYINVQLFNSLLLRRECCSFSNGEYLKAGLQELES
WCSKATEQYAGSSWDELQHIRQAVGFLVLHQKSQKALDEITNDLCPMLSIAQIYRIGTMFWDDKYGAHGLSPE
VISKMRALTLEDSASIPNNTFLLDVDSSIPFSIEEISRSFQNINLSDVEPPPLLCQRSDFQFLLQAAA*
>PGSC0003DMP400054217
MGTPVNIIVGSQVWIEDPDDAWIDGEVTEIKGSNATVATTNGKTTVASISSIYPKDTEAPPSGVDDMTKLAYLH
EPGVLNNLACRYSLNEIYTYTGNILIAVNPFRRLPHLYDTHMMQQYKGAPFGELSPHLFAVADACYRALINEHGN
QSILVSGESGAGKTETTKMLMRYLAFMGGRSGTEGRTVEQQVLESNPVLEAFGNAKTVKNNNSSRFGKFVEIQ
FDKHAKISGAAVRTYLLERSRVCQVSDPERNYHCFYMLCAAPPEDVKRFKLGNPKSFHYLNQSSCYEVANVDDA
REYLETRNAMDVVGIGQEEQEAIFRVVAAILHLGNINFVKGKEADSSKLKDEKSLFHLKTAAELFMCDEKALEDS
LCKRVIVTPDGNITKLLDPAAATTSRDALAKTVYSRLFDWLVDKINNSIGQDPEAKSIIGVLDIYGFESFKINSFEQF
CINLTNEKLQQHFNQHVFKMEQDDYTTEEINWSYVEFVDNQDVLDLIEKKPGGIIALLDEACMFPKATHETFAQ
KMYQTYRAHKRFSKPKLARTDFTINHYAGDVTYQADHFLDKNKDYVIAEFQALLMDSKCFFVANLFPPLPEESS
KQSKFSSIGTRFKQQLQSLMETLSTTEPHYIRCVKPNTVLKPGIFENMNVLNQLRCGGVLEAIRISCAGYPTKRTF
DEFLDRFGTLAPDVLDGCDEKSACIAICDRMGLKGYQIGKTKVFLRAGQMAELDARRTEVLAHAAKRIQRQIRT
YLTRKEFIALKRATIHFQKLWRAQLARVLYEQMKREAASIRIQKHARSHSARKSYKELQAAAVVIQTGMRAMAA
RNEYRQRRRNKAAKIVQTQWRGFHAFSTYKQKKKASLSLQCLWRGRLARKVLRKLRMDARDTGALKEAKDKL
EKRVEELTWRLDFEKHLRIDLEEAKGQEISKLQKALQEMQTQLDEAHDAIIHEKEAAKIAIEQAPPVIKEVPVIDN
TKVEKLTEENNKLEEEIRELKKRVEDFEQSYNEVEKECQATRKEAEESQLRVSEFQESIERLQLNLSNLESENQVLR
QQALVASTNEALSDEMDILKNKIKDLESENELLRTQRVVVEQVVSSDDRAPKGLETVDITHPADNEHQTEEVHE
EMKVEQQIPKLLQDSSPPISLTKQRSLTDRQQESHDILIKCLAEDKRFDKGRPVAACTLYKALLQWRSFEAEKTNIF
DRIIHTIRSSIEDNTGDLAYWLSTSSTLLFLLQSTIKAGNAPTRSPYRNRSSPTTLFGRMAQGFRSTSLSMAISSGYS
GIEGSPNVRTRIEAKYPALLFKQHLTACVEKIYGMIRDNLKKEISPFLNQCIHAPRSARIKPLKGSSRSIHSNIIAKQQ
ASSVHWQNIVNSLDNTLTILSENNVPSTITRKILSQVFSYINVQLFNSLLLRRECCSFSNGEYLKAGLQELESWCSK
ATEQYAGSSWDELQHIRQAVGFLVLHQKSQKALDEITNDLCPMLSIAQIYRIGTMFWDDKYGAHGLSPEVISK
MRALTLEDSASIPNNTFLLDVDSSIPFSIEEISRSFQNINLSDVEPPPLLCQRSDFQFLLQAAA*
>PGSC0003DMP400054218
MGTPVNIIVGSQVWIEDPDDAWIDGEVTEIKGSNATVATTNGKTTVASISSIYPKDTEAPPSGVDDMTKLAYLH
EPGVLNNLACRYSLNEIYTYTGNILIAVNPFRRLPHLYDTHMMQQYKGAPFGELSPHLFAVADACYRALINEHGN
QSILVSGESGAGKTETTKMLMRYLAFMGGRSGTEGRTVEQQVLESNPVLEAFGNAKTVKNNNSSRFGKFVEIQ
FDKHAKISGAAVRTYLLERSRVCQVSDPERNYHCFYMLCAAPPEDVKRFKLGNPKSFHYLNQSSCYEVANVDDA
REYLETRNAMDVVGIGQEEQEAIFRVVAAILHLGNINFVKGKEADSSKLKDEKSLFHLKTAAELFMCDEKALEDS
LCKRVIVTPDGNITKLLDPAAATTSRDALAKTVYSRLFDWLVDKINNSIGQDPEAKSIIGVLDIYGFESFKINSFEQF
CINLTNEKLQQHFNQHVFKMEQDDYTTEEINWSYVEFVDNQDVLDLIEKKPGGIIALLDEACMFPKATHETFAQ

KMYQTYRAHKRFSKPKLARTDFTINHYAGDVTYQADHFLDKNKDYVIAEFQALLMDSKCFFVANLFPPLPEESS
KQSKFSSIGTRFKQQLQSLMETLSTTEPHYIRCVKPNTVLKPGIFENMNVLNQLRCGGVLEAIRISCAGYPTKRTF
DEFLDRFGTLAPDVLDGCDEKSACIAICDRMGLKGYQIGKTKVFLRAGQMAELDARRTEVLAHAAKRIQRQIRT
YLTRKEFIALKRATIHFQKLWRAQLARVLYEQMKREAASIRIQKHARSHSARKSYKELQAAAVVIQTGMRAMAA
RNEYRQRRRNKAAKIVQTQWRGFHAFSTYKQKKKASLSLQCLWRGRLARKVLRKLRMDARDTGALKEAKDKL
EKRVEELTWRLDFEKHLRIDLEEAKGQEISKLQKALQEMQTQLDEAHDAIIHEKEAAKIAIEQAPPVIKEVPVIDN
TKVEKLTEENNKLEEEIRELKKRVEDFEQSYNEVEKECQATRKEAEESQLRVSEFQESIERLQLNLSNLESENQVLR
QQALVASTNEALSDEMDILKNKIKDLESENELLRTQRVVVEQVVSSDDRAPKGLETVDITHPADNEHQTEEVHE
EMKVEQQIPKDSSPPISLTKQRSLTDRQQESHDILIKCLAEDKRFDKGRPVAACTLYKALLQWRSFEAEKTNIFDRI
IHTIRSSIEDNTGDLAYWLSTSSTLLFLLQSTIKAGNAPTRSPYRNRSSPTTLFGRMAQGFRSTSLSMAISSGYSGIE
GSPNVRTRIEAKYPALLFKQHLTACVEKIYGMIRDNLKKEISPFLNQCIHAPRSARIKPLKGSSRSIHSNIIAKQQAS
SVHWQNIVNSLDNTLTILSENNVPSTITRKILSQVFSYINVQLFNSLLLRRECCSFSNGEYLKAGLQELESWCSKAT
EQYAGSSWDELQHIRQAVGFLVLHQKSQKALDEITNDLCPMLSIAQIYRIGTMFWDDKYGAHGLSPEVISKMR
ALTLEDSASIPNNTFLLDVDSSIPFSIEEISRSFQNINLSDVEPPPLLCQRSDFQFLLQAAA*
>PGSC0003DMP400033806
MGKASKWIRNFLMVMGKKEEREKKEYKSIESMGTPTTPKAKRRWSFKKSSSMERKSHKSNRSFDLTFDHQLN
TQGSMLEFDMLEKHHKASLTAKGAIKPKAYLTRRVKDAAATKIQAVFRAYLARKALRALRSLVRLQALVRGHLVR
KQTAAMVRRMHSLMVIQLRARVQRVQMTKEAHTPNSRKSQKVSPGNNQLTRACSIEKMDVSIQEKGRVQK
NNSTKNRSSGMENGLSTSESRCLSLSRRSHQVLQTCPSPSTLSDMSTISYDRHLEDFSFKTPEKGFEHCSNVSTTT
SSKTPFSIPHSEKPNSIFSSATLALTYMSNTESSRAKARSHSEPRQRPNWSIIRKSKRTPSMDGITGIPDSRREETPT
HSRRHNVPESHEAWLLKLYKQAKSIKHVKVDSASIVSAV*
>PGSC0003DMP400030791
MGKSPGKWLRSLLPGKKSSKSGTSKKSSNEKASVISTNAALSGSSVHLPLISEPVAGNSGGIKEDSNFEKGEVTDE
VILPSIERDGDEQNTCLTLPEDTEKMRLEQAAMKAQAIVRGYLARRAFLRLKGTIRLQAAVRGHLVRRQAVATLY
CIHGIVKLQAHIRGQIIRRSSIGCELITKQGLEKQDAKQLDYQRTNASKLARELSKNEFTTKLLASSPTVMPLHLHY
GPEEPNSSQEWLVRWTISQIWQPQPKSETLSRKKHQNVEADIAMSKHSGRKLHSRKMQNGSNHSTSSGSEK
KKSSHLVNSVLQNPGSEIKKVKHSVKKMSSPILEKPIQSEVDTERKRQSHDKLSSMTSDEPLQNSEGIVENSTNV
APSQETLGVDDTISRLDILSVSDTLHKSTTDAAYQKPITDNQEDDTPVANEDSCTNHDNNEGNESNKVNRRVSL
PAKHDVDASTPTTPTTRKVPSYMAPTKSAKAKLKEQASPRFGQDVAEKNAVTRRHSLPSPMNGKLSSSPSPRV
QRLVQASAKEGIKIDRSLSSSRDGTDKMTRAEWKR*
>PGSC0003DMP400056415
MWKDMLDHYGVSASAESQTKYLHKLDENAMLQTSSERRAIEAYESYKWCDFSDREAQTAPVPAFKQLEDFKYT
TYPPAITTFGSNPDEYTTIFDQDQIGTSLEDEMSLTIAQTQKFTIRHISPDWGYSSEATKIVIIGSFLCNPSECTWTC
MFGDIEVPVQIIQEGVICCQAPRHLPGKVTLCVTSGNRESCSEVREFEYRVKPDDCARNNQPDVEGAYGSTEEL
LLLVRFVQLLLSDLSVQKGESSELGNDFLEKSKASEDSWSQIIESLLFGSSMPMVTIDWLLQELLKDKFQQWLSC
KLQQKDNQIGCSLSKKEQGVIHMVAGLGFEWALHPILNAGVSVNFRDINGWTALHWAARFGREKMVASLIAS
GASAGAVTDPSSRDPVGKTAASIASSCDHKGLAGYLSEVALTSHLSSLTLEESELSKGTADVEAERTISSISNTSATI
NEDQRSLNDTLAAVRNAAQAAARIQSAFRAHSFRKRQEREFGVSASGDEYGILSNDIQGLSAASKLAFRNPRDY
NSAALAIQKKYRGWKGRKDFLAFRQKVVKIQAHVRGYQVRKQYKVCWAVGILEKVVLRWRRRGVGLRGFRH
DTESIDEIEDEDILKVFRKQKVDAALDEAVSRVLSMVESPGARQQYHRILEKYRQAKAELEGADSETASTAHGD
MSNMENDDIYQFPSY*
>PGSC0003DMP400008334
MTVQNHEQRLLEINTLEWDDLLAPGDPNKIVATQQGSKTAYVQHTSYEQHNLCELNGYSLNGVSSSLERISTVN
NSNEIIFQTVDGQMTPSFEKNESGVMTVSTGDSFDSLNQDRLQTQDSFGRWMNYFITDSPESTDDPTLESSVS

TGQSYAREQTFNITEISPAWASSTEETKIIVIGQFHGEQSHLESSCLHCVCGDACFPAEVLQPGVYRCIVSPQTPGL
VNIYLSFDGNKPISQVMSFEFRAPSVQVWTEPPESKSDWDEFRNQMRLAHLLFSTSKSLNILSSKIHQDLLKDAK
TFAGKCSHIIDDWACLIKSIEDKKVSVPRAKDCLFELSLKTRLQEWLLERVVEGCKISEHDEQGQGVIHLCAILGYT
WAVYLFSWSGLSLDYRDKYGWTALHWAAYYGREKMVATLLSAGAKPNLVTDPTSENLGGCTASDLASKNGHE
GLGAYLAEKALVAQFNDMTLAGNISGSLQTTTESINPGNFTEEELNLKDSLAAYRTAADAAARIQAAFRERALKV
RTEAVESSNSEMEARNIIAAMKIQHAFRNYEMQKQLAAAARIQYRFRTWKMRREFLHMRRQAIKIQAVFRGF
QVRRQYRKITWSVGVLEKAIFRWRLKRKGLRGLKLQSSQVVKSDDAEEDFFQASRKQAEERIERSVVRVQAMF
RSKQAQEQYRRMKLEHNKAMLEYEGTLNPDTEMD*
>PGSC0003DMP400008336
MSFEFRAPSVQVWTEPPESKSDWDEFRNQMRLAHLLFSTSKSLNILSSKIHQDLLKDAKTFAGKCSHIIDDWAC
LIKSIEDKKVSVPRAKDCLFELSLKTRLQEWLLERVVEGCKISEHDEQGQGVIHLCAILGYTWAVYLFSWSGLSLD
YRDKYGWTALHWAAYYGREKMVATLLSAGAKPNLVTDPTSENLGGCTASDLASKNGHEGLGAYLAEKALVAQF
NDMTLAGNISGSLQTTTESINPGNFTEEELNLKDSLAAYRTAADAAARIQAAFRERALKVRTEAVESSNSEMEAR
NIIAAMKIQHAFRNYEMQKQLAAAARIQYRFRTWKMRREFLHMRRQAIKIQAVFRGFQVRRQYRKITWSVGV
LEKAIFRWRLKRKGLRGLKLQSSQVVKSDDAEEDFFQASRKQAEERIERSVVRVQAMFRSKQAQEQYRRMKLE
HNKAMLEYEGTLNPDTEMD*
>PGSC0003DMP400019257
MKKEAASIKIQTKLRGHLARKSYTGLKINVIALQTGIRATAARKEFRYKRQTKAAINIQAHWHGHRAFSYYKKLIIA
SIVTQCRWRGRVAKKELRKLKMASRETGALKEAKDKLEKQVEELTWRLQLEKRLRTDLEEAKSQEIAKLKNTLED
VHSKVDQTNALLIKERETAQKAIEEATSIVEEKPVLVEDTEKIDALNAEVENLKVLLQSEKQRADDSERKCAESQES
SEEKHKKLEETERKVQQFQESMSRLEEKLTNIESENKVLRQQALTMAQNNKLLSGRSRSSIQRNESSTRNSVDLH
STSFSRESAEVEGRPQKSLNDKQQEYQDLIIRCIAQHLGFSKGRPVAACIIYKCLRQWRSFEVERTSIFDRVIQTIG
QAIETQDNNDMLAYWLSNASTLLLLLQRTLKAGGAAGMTPQHRRSSSASLFGRMTQSFRGTPQGVNISLIDG
DSAGGVDTLRQVEAKYPALLFKQQLTAYVEKIYGMIRDNLKKEISPLLGLCIQAPRISRASLLKGTTARTLANAAAQ
EILIAHWQGIVKSLANFLNLLKANHVPPFLVRKVFTQVFSFINVQLFNSLLLRRECCSFSNGEYVKTGLAELEHWC
YKATDEYTGLAWEELKHIRQAIGFLVIHQKPKKTLDEISHDLCPVLSIQQLYRISTMYWDDKYGTHSLSSDVIANM
RVLMTEDSNNAVSNSFLLDDDSSIPFSIDDLSKSMDQIDIADIEPPPLIRENSGFSFLLPRAD*
>PGSC0003DMP400034070
MGKKGSGSWFSTVKKVFIKPSSKDYSPADHKKKEKLENQWQDEAPEVEQFPAESSSDLTNNRESNDNSSSSLA
EDRNHDIHVIEASVAVATYTAPKIVKLDGYNKEEIAATLIQSYYRGYLARRALRALRGLVKLQALVRGHSVRKQAQI
TMRCMQALVRVQSKVRARRLQLLQSKVEEVKLHSIIREDQHKNKSLSNKQETEGWDNRNQSIEKILQNTRRKQ
HAYQKWLHSDPDDDEECFCNEHENPQQSWNWLDRWMASQHAVRREGSYVSLSITDDISEKTVELDPLGSED
VNLAHHILSPVERSPYSSTQSNMDSVPSYMAPTKSAKAKIRSPGPIKPKSPPGVAQWNATPKKVTARRWSYDSS
ARSPNPKTSAKWMATYSPETRVHDRASPMGAPACRYNYN*
>PGSC0003DMP400025918
MEAIRISCAGYPTRKPFYEFLDRFGILSPEVLDGSTDEVTACTRLLEKVGLQGYQIGKTKVFLRAGQMAELDSRRT
EVLGRSASIIQRKVRSHMARRNFTLLRQLARRIQSMCRGELARRVYESLRREAACLKIQTDMRMHLARKGYKEL
CSAAISIQTGMRGMAARNEVRFRRQTKAAIIIQSHSRAFLARLKYKKLKKAAITTQCAWRARVARGELRKLKMA
ARETGALQAAKNKLEKQVEELTWRLQLEKRMRADMEEAKTQENAKLQSALQEVQLQFKETQEMFVKERETTK
RAAEEVPIMQEVPVVDHEMMNKLSVENEKLKSLVSSLEQKIDETEKKYEETSKLSEERLRQVLDAESIIVQLKTT
MQRFQERNFDLESENQILQQSLLAPAKQVSDHSPSLSSKVQIEENGYHLKEETRTNDPPGSTPAKKVETPNSKSR
KPPIDRQREDIGALIDCVMKDVGFSQSKPVAAFTIYKCLLHWKSFEAEKTSVFDRLVQMIGSAIENQDSDDHMA
YWLSNTSTLLLLIQKSLKPDSAVGATPTRKPQPATSLFGRMTLGFRSSSSDINLAGVVHQVQAKYPALLFKQQLTA
YVEKMYGIIRDNLKKELGSLLSLCIQAPRTSKGSVLKSGRSFGKDYSINHWRGIIECLDSLLCTLKENFMPPILVQKI

FSQAFSYMNVQLFNSFLLRRECCTFSNAEYVKSGLAELELWCSQAKEEYAGSSWDELRHIRQVVGFLVIHQKYRI
SYDDITNDLCPVLSVQQLYRVCTLYWDDKYNTRSVSPDVISNMRVLMTEDSNDAKSNSFLLDDNPSIPFSIEEVS
NSLQVKDFADVKPATKLLENPAFQFLHE*
>PGSC0003DMP400025919
MEAIRISCAGYPTRKPFYEFLDRFGILSPEVLDGSTDEVTACTRLLEKVGLQGYQIGKTKVFLRAGQMAELDSRRT
EVLGRSASIIQRKVRSHMARRNFTLLRQLARRIQSMCRGELARRVYESLRREAACLKIQTDMRMHLARKGYKEL
CSAAISIQTGMRGMAARNEVRFRRQTKAAIIQSHSRAFLARLKYKKLKKAAITTQCAWRARVARGELRKLKMA
ARETGALQAAKNKLEKQVEELTWRLQLEKRMRADMEEAKTQENAKLQSALQEVQLQFKETQEMFVKERETTK
RAAEEVPIMQEVPVVDHEMMNKLSVENEKLKSLVSSLEQKIDETEKKYEETSKLSEERLRQVLDAESIIVQLKTT
MQRFQERNFDLESENQILQQSLLAPAKQVSDHSPSLSSKVQIEENGYHLKEETRTNDPPGSTPAKKVETPNSKSR
KPPIDRQREDIGALIDCVMKDVGFSQSKPVAAFTIYKCLLHWKSFEAEKTSVFDRLVQMIGSAIENQDSDDHMA
YWLSNTSTLLLLIQKSLKPDSAVGATPTRKPQPATSLFGRMTLGFRSSSSDINLAGVVHQVQAKYPALLFKQQLTA
YVEKMYGIIRDNLKKELGSLLSLCIQAPRTSKGSVLKSGRSFGKDYSINHWRGIIECLDSLLCTLKENFMPPILVQKI
FSQAFSYMNVQLFNR*
>PGSC0003DMP400038095
MAASISLPVGSLVWVEDPDVAWIDGEVLEVNGSDIKVLCTSGKTVAVKSSNVYAKDAEAPPSGVDDMTKLAYL
HEPGVLHNLKARYDINEIYTYTGNILIAVNPFRRLPHLYDTHMMAQYKGAAFGELSPHPYAVADAAYRLMINDG
VSQSILVSGESGAGKTESTKQLMRYLAYMGGRAAAEGSRSVEQQVLESNPVLEAFGNAKTVRNNNSSRFGKFV
EIQFDQKGRISGAAVRTYLLERSRVCQLSDPERNYHCFYMLCAAPPEDIQRFKLDNPRTFHYLNQTNCYELDGL
DDAKEYLATRRAMDVVGISSEEQDAIFRVVAAILHLGNIEFAKGKEIDSSVPKDEKSWFHLRTAAELFMCDVKSL
EDSLCKRVIVTRDETITKWLDPEAALTSRDALAKIVYSRLFDWLVDTINSSIGQDPNSKSLIGVLDIYGFESFKTNSF
EQFCINLTNEKLQQHFNQHVFKMEQEEYTKEEINWSYIEFIDNQDILDLVEKKPGGIIALLDEACMFPRSTHETFA
QKLYQTFKNHKRFCKPKLARSDFTICHYAGDVTYQTELFLEKNKDYVIAEHQALLNASTCSFVSGLFPTSNEESSK
QSKFSSIGSRFKQQLQSLLETLNATEPHYIRCVKPNNLLKPSIFENHNVLQQLRCGGVMEAIRISMAGYPTRRPFY
EFLDRFGILSPEVLDGSTDEVAACKRLLEKVGLQSYQIGKTKVFLRAGQMAELDARRTEVLGRSASIIQRKVRSY
MARRSFTVLRRSTIQIQSLCRGELARRVYESLRREAASLRIQTNVRMHIARKAYEELRSSAVSIQTGLRGMAARN
ELRFRSQTKAAIIIQSHCRKFLAYSKFKKLKKAAITTQCAWRAKVARKELKKLKMAARETGALQAAKNKLEKQVEE
LTWRLQLEKRMRADVEEAKTQENAKLQSALQEMQVQFKETKEMLVKERENAIRAAEQIPIVQEVPVIDHELM
NKLSIENENLKTMVSSLEKKIGETEKKYEETNKLSEERLKQAMEAESKIVQLKTSMQRLEEKIVDMESENKILRQQ
GLLTPAKRVSDHSPSLASKIVENGHHLDDENYTTDALSSSTPSKNFETPDSKMRRPPVDRQQHEDVDALIDCV
MKDVGFSQGKPVAAFTIYKCLLHWKSFEAERTSVFDRLIQMIGSAIENQESNDHMAYWLSNTSTLLFLIQKSLKP
GGSVGATPTRKPQPPTSLFGRMTMGFRSSPSAVNLAAAAAALVVRQVEAKYPALLFKQQLTAYVEKIYGIIRDNL
KKELGSLISLCIQAPRTAKGSLRTGRSFGKDTSTNHWQRIIEGLNSLLCTLKENFVPPILVQKIFTQTFSYINVQLFNS
LLLRRECCTFSNGEYVKAGLAELELWCCQAKEEYAGSSWDELKHIRQAVGFLVIHQKYRISYDEITNDLCPILSVQ
QLYRICTLYWDDNYNTRSVSPDVISSMRVLMTEDSNNAESNSFLLDDNSSIPFSIDEVSESLQVKDFADVKPATEL
IEHPAFPFLHE*
>PGSC0003DMP400016001
MGKKGSGWFSSVKKVFKQSPKDSPDKKKETLDSKWQQPEPPEVVSLEHFPAGSSPDLISNGGSNVDSSSTVAE
DHNHAIAVAAATAAAAEAAVAAAHAAAKVVRLAGGYGLLQSTEERAATLIQSYYRGYLARRALRALKGLVRLQA
LVRGHNVRKQAQMTMRCMQALVRVQGRVRARRLQLVQERLESKLEEARRHAAEEQQHKHTSPIKKSGAEG
WDNRNQSMDKIQESSRRKFDAEMKRERALAYAFAYQQKQQQLLHIDPHGDDAEFFGNEREKSQWGWSWLE
RWMASQPYQYSRHIVPRDSSYVTPSTTSDMSEKTVEMDLITGPLDSETINSARHTFDQAETSPYSARKVGHSKF
DNVPSYMTPTKSAKAKVRNQGPVKHRSPPSAQWNASKRKGAHYGLGLESSSGASTTAIYQVSRSPSPKTTANR
SSGHANKWMATYSPESSGDDRASPLGPHPWRYNFS*

>PGSC0003DMP400004403
MGGSGKWIKSLIGLKKNQSNDSEKGSGKNRKWKLWRSASGGIAMAFSKGVKGGGNLGDSDESESSFLSDSAL
AAAMATVIRAPHKDFVVVKQEWAALRIQAAFRGFLARRALRALKAVVRLQAIFRGRQVRKQADVTLKCMQTL
VKLQSRVRARCHQTSVDATQGSLVDSQADPIKQAEGGWCDSPGTVDEVRCKLKMRQVGAIKRERAIAYAQQK
LRTNPSPNSRTRKVETPNKFKANGDSVWLERWMASKPWENRLVEDFHTDASGMTPSSRKYEDYDAGSFTDR
SSVNIRRNNMSTRISTRGPMSCQIANSSSEPYTDYYQYDDSTTSHSSISTSETLGSNQTPPEEGHSKKPNYMNLT
KSIKAKVKQRNSNYLSHSMQRNSTDNLHVHRKSSPLSRTIARRSADCDLYSVDLCKDLYPPSNAF*
>PGSC0003DMP400021405
MGKKKGGTSWLSAVKRAFRSPTKDNSCDKKAKIEHELDEDEEKKREKRRWLFRKQSQNEGKVIVDPKHATAAA
AVATAQAAVEIIRLTRSSNNPSSNRQHNAAVLIQTAFRGYLARRALIALKGIVKLQALIRGQNVRKQAKMTLKCM
QALLRVQARVREQRARLSHDGGRRSMFAETTNLWDSKYLRDIRDRKSRSRDGSSIADDCPRSLLELESMLQAR
KEASFKREKSLAHAFTQQELDEMDVCSEERNERELEETANWLDEWMSSKQWNTSNRGSFDRRDSIKTVEMD
TAKPYSNMVPNARRSQHSSPLHRQASSPHYIANSPHHQRSSHYNYSAIQPPATPPPCQPKPLQMRPTSPRKSQ
STANTPCLRSTSRSNSIMSRYSTSGNDASVPNYMAATESAKARIRSQSTPKQRPSTPERERVGSVKKRLSYPIPEP
YSLNAAYGYSQNLRSPSFKSLQAAYVGMEQQSCYTDSLGGEISPCSTTDLRRWLR*
>PGSC0003DMP400021404
MGKKKGGTSWLSAVKRAFRSPTKDNSCDKKAKIEHELDEDEEKKREKRRWLFRKQSQNEGKVIVDPKHATAAA
AVATAQAAVEIIRLTRSSNNPSSNRQHNAAVLIQTAFRGYLARRALIALKGIVKLQALIRGQNVRKQAKMTLKCM
QALLRVQARVREQRARLSHDGGRRSMFAETTNLWDSKYLRDIRDRKSRVSSTFRALVTV*
>PGSC0003DMP400010241
MGKASKWFKALLGFKKNDSSSSSTSSTNKKKWSDVKSYKDKDFQHHHQHHDKSHYVNSRAGVDPTICEVHSS
LTSSVIRTTTSVTPWSGEEWAAVVIQSHFRAYLSRRALRALKGLVKLQALVRGHIVRKQTADMLRRMQALIRAQS
RARLGRSMVFESPPFSAKSTQFIPHGPTTPDKFEQIIRARSMKNDQMFMLKRNSSNHIGNANAKQRNLFHSSE
FSLNSEQFCFSYDETCYSSIDNSPQLHSTASSKCTRSRTGPFTPTKSSTRSYTSDEYSNNHPNYMSYTEAAKAKTRS
MSAPRLRSQYDKRYSRSNMQKGSNCYANFTSKGVCSGSDRLDKIGVPISGDLDEFGRGFCHIY*
>PGSC0003DMP400028070
MGKKGSWFSAIKRVFTPSSKEKLPNESEKKGAKEKKSRGKLKHGETKSFIPLFREPSSIEKILGEVDEQMLLSPRFT
LPAEVVSPRISSYRFATPSATSPRVASPKASSRRITSPKAPSQRVTSPRAISPKAHPPRVPSPNVSRNRKEISYAYRP
EPTLRTLNLSATKIQAAYRGYMARRSFRALRGLVRLQGVVRSSNVKKQTANAMKQMQLLVRVQTQIQSRRIQM
LENQALQHQAYRNDKEVESTISKWTQLCEAGNNDNWDDSLLTKEEVEGRLRKKVEAVIKRERAMAYAYSHRL
WKNDPKSGLDMGANGFPWWWNWLERQLPSRNANKTPSAVKDIKLTPPRAISEHKPSPTPLNNVTFRRILSD
YDNNESSVTPMSTKSAIPTRGKQMHTPIRTPPMNNSSLKKHSRARASASNYPFDLPLKDDDSLTSCPPFSVPHY
MSQTASAKARANSNPKERNPEKQSNDTKKRFSFPLTPNIWSSKWSKGSGKDPTSRKEVDKHESMADHISVDST
VSMPAVVGGRRPFNRFV*
>PGSC0003DMP400022535
MGKRRSWFIFVKRLLFIPEAKPKAEKKPKRWKWFLGRFKFKQCPPAIEAPQKTLTEATEQQKKLAVAVALATAAA
AEAAVAAANAAAEVVRLTIAPYELERKRRNAAIRIQTAYRAHLARKALSALKGLVKLQAVIRGELVRRRLVAKLKF
MLPFQMPKPRVYHIRVPTVEEYYESIEKKLDDSSKESVKSNEFKLKCNSQRTWDFSLASKEEIDSLFLRRREAFAK
RERMMKYSFSHRERRNDHVMQDPLIIKENRRSSRFDQWEEMEAQRKAELFEQLRSFANSSSPLVGMNQMR
QTRKLDVTEDFNSPSSLPRRSFSHVKQKSIGDDSSLPSSPMFPTYMAATESAKAKTRSMSTPKQRLMLNETYSV
QHSPFMLNHTSWTSYNGEVNKSTKKSEISQQTFL*
>PGSC0003DMP400006641
MGKSTASSCLKIIACGSDSVDRDELEAHPESKSSSDKRGWSFRKKSARHRVLSNTVVSETPSGNKDWPEAANA
NLQTQSNSTIPEKASVVQWADEKPQFPTVEKSQVSADEKPQVLANENPQISEDEKPQVLEDEKLQVSVDEKPQ

VSTDAKPQLLVEVSVDEKPLISEKVNLEVSEDEKPSVSSDEKAPISSEENSLLSDLVDAKQSEPVTARVNDGKADVI
LDEHALVIQTAVRAFLARRAQLKQKHITKLQAAVRGHLVRRHAVGTLRCVQAIVKMQTLVRAHHTNRIAEGSSIK
EKLKGKENSGTKSEFTYISISKLLSNSFARQLLESTPRTKSINIKCDPSKSDSAWKWLERWMSVASPGNQLSPQSE
LSAEQQENEPTEHHSNLMESKVQLDSESMDFREGEEASLSAVPSESDDNLITYDADSLDFQADIPTLPPQPLNV
DEKTSRDDCSIPTQLKEARALPEMEPNSFPANTEVEREDTHSLELSETESKKILHGSRKASNPAFIAAQSKFEELTL
AAKSTKVTSLPNHKTEDESSEDTFSTITDHSFGAREAAPSENSVPHSTRAQVGGSECGTELSISSTLDSPDRSDVG
GHVFEQELPSNGGTDHRKSNGYPHIEDDSTNDLSHSDYVQAGREDPTDDAKHVDVMVSSDLSPEEQKPENN
SVNVQIEHEAKTDRLYKSSPDASPRSHITVPESQGTPSSQVSVNPKKLKSENSGSIPKPRSAPASKKSPSKLNHAP
GTTSSEQLSKDHNKNEKRRNSFGSTKAGQADQEARDNSTSSSLPSYMQATESARAKVIPNSSPRSSPDVHNKD
EYIKKRHSLPGSNGRQGSPRIQRSLSNAQQGAKGNGTQSPQERKWQR*
>PGSC0003DMP400006640
MGKSTASSCLKIIACGSDSVDRDELEAHPESKSSSDKRGWSFRKKSARHRVLSNTVVSETPSGNKDWPEAANA
NLQTQSNSTIPEKASVVQWADEKPQFPTVEKSQVSADEKPQVLANENPQISEDEKPQVLEDEKLQVSVDEKPQ
VSTDAKPQLLVEVSVDEKPLISEKVNLEVSEDEKPSVSSDEKAPISSEENSLLSDLVDAKQSEPVTARVNDGKADVI
LDEHALVIQTAVRAFLARRAQLKQKHITKLQAAVRGHLVRRHAVGTLRCVQAIVKMQTLVRAHHTNRIAEGSSIK
EKLKGKENSGTKSEFTYISISKLLSNSFARQLLESTPRTKSINIKCDPSKSDSAWKWLERWMSVASPGNQLSPQSE
LSAEQQENEPTEHHSNLMESKVQLDSESMDFREGEEASLSAVPSESDDNLITYDADSLDFQADIPTLPPQPLNV
DEKTSRDDCSIPTQLKEARALPEMEPNSFPANTEVEREDTHSLELSETESKKILHGSRKASNPAFIAAQSKFEELTL
AAKSTKVTSLPNHKTEDESSEDTFSTITDHSFGAREAAPSENSVPHSTRAQVGGSECGTELSISSTLDSPDRSDVG
GHVFEQELPSNGGTDHRKSNGYPHIEDDSTNDLSHSDYVQAGREDPTDDAKHVDVMVSSDLSPEEQKPENN
SVNVQIEHEAKTDRLYKSSPDASPRSHITVPESQGTPSSQVSVNPKKLKSENSGSIPKPRSAPASKKSPSKLNHAP
GTTSSEQLSKDHNKNEKRRNSFGSTKAGQADQEARDNSTSSSLPSYMQATESARAKVIPNSSPRSSPDVHNKD
EYIKKRHSLPGSNGRQGSPRIQRSLSNAQQGAKGNGTQSPQGIF*
